# Functional evolution of ADAMTS genes: Evidence from analyses of phylogeny and gene organization

**DOI:** 10.1186/1471-2148-5-11

**Published:** 2005-02-04

**Authors:** Ainsley C Nicholson, Shehre-Banoo Malik, John M Logsdon, Erwin G Van Meir

**Affiliations:** 1 Laboratory of Molecular Neuro-Oncology, Neurosurgery Department and Winship Cancer Institute, 1365-C Clifton Road, Room C5078, Emory University, Atlanta GA 30322 USA; 2 Roy J. Carver Center for Comparative Genomics, Department of Biological Sciences, 300 Old Biology Building, University of Iowa, Iowa City IA 52242-1324 USA

## Abstract

**Background:**

The ADAMTS (A Disintegrin-like and Metalloprotease with Thrombospondin motifs) proteins are a family of metalloproteases with sequence similarity to the ADAM proteases, that contain the thrombospondin type 1 sequence repeat motifs (TSRs) common to extracellular matrix proteins. ADAMTS proteins have recently gained attention with the discovery of their role in a variety of diseases, including tissue and blood disorders, cancer, osteoarthritis, Alzheimer's and the genetic syndromes Weill-Marchesani syndrome (ADAMTS10), thrombotic thrombocytopenic purpura (ADAMTS13), and Ehlers-Danlos syndrome type VIIC (ADAMTS2) in humans and belted white-spotting mutation in mice (*ADAMTS20*).

**Results:**

Phylogenetic analysis and comparison of the exon/intron organization of vertebrate (*Homo*, *Mus*, *Fugu*), chordate (*Ciona*) and invertebrate (*Drosophila *and *Caenorhabditis*) ADAMTS homologs has elucidated the evolutionary relationships of this important gene family, which comprises 19 members in humans.

**Conclusions:**

The evolutionary history of *ADAMTS *genes in vertebrate genomes has been marked by rampant gene duplication, including a retrotransposition that gave rise to a distinct *ADAMTS *subfamily (*ADAMTS1*, *-4*, *-5*, *-8*, *-15*) that may have distinct aggrecanase and angiogenesis functions.

## Background

ADAMTS (A Disintegrin-like and Metalloprotease with Thrombospondin motifs) proteins have homology with the metalloprotease region of the ADAM proteases, but also have at least one of the Thrombospondin type 1 Sequence Repeat (TSR) motifs that are common in extracellular matrix proteins. Since the discovery of a gene encoding ADAMTS1 in 1997 [1], a total of 19 similar genes have been found in the human genome [2], numbered *ADAMTS1-20*; there is no *ADAMTS11 *because early reports of an *ADAMTS11 *[3] were later found to describe *ADAMTS5*. Many of these genes have been implicated in a variety of diseases, including connective tissue disorders [4], cancer [5-7], osteoarthritis [3,8], and possibly Alzheimer's disease [6,9]. Recently, an autosomal recessive form of Weill-Marchesani syndrome (WMS) has been attributed to null mutations of the *ADAMTS10 *gene [10]. The symptoms characteristic of this syndrome (short stature, brachydactyly, joint stiffness, and anomalies of the eye lenses), together with its expression patterns, suggest a role for the gene encoded by this protein in normal growth and in skin, eye, and heart development.

ADAMTS proteins are characterized by a pro-domain, a metalloprotease domain, the so-called disintegrin-like and spacer domains, and a tail of TSR repeats. The pro-domain of ADAMTS1 and -4 is cleaved at the RX(K/R)R furin cleavage site [11] in the Golgi [12,13], releasing an active protein [14]. There are clearly conserved furin cleavage sites for most human ADAMTS proteins (positions 578–581 of the alignment) [Additional File 2]. While this site was less well conserved in ADAMTS10 and ADAMTS12, the pro-domain of ADAMTS12 was also shown to be removed by a furin-mediated process [7]. On the basis of this combined evidence, it is commonly believed that furin cleavage of the pro-domain might occur for all ADAMTS proteins.

The metalloprotease domain of ADAMTS proteins is shared with the related ADAM proteins, and the catalytic Zn2+-binding motif HEXGHXXXXXHD [15] is well conserved, shown at amino acid positions 761–772 [Additional File 2]. While the metalloprotease domain of ADAM proteins is followed by a disintegrin domain which binds integrins at a conserved X(D/E)ECD site [16,17], the corresponding amino acids in the disintegrin-like domain of ADAMTS proteins are not well conserved. We also note that the so-called spacer domain following this disintegrin-like domain (amino acids 1060–1400) [Additional File 2] in fact has many highly conserved residues, despite its comparatively reduced overall conservation of amino acid sequence.

There are four matrix metalloprotease (MMP) cleavage sites in the spacer domain of ADAMTS1 [14,18], including the highly conserved IPAGA site at amino acid positions 1229–1233 [Additional File 2] (*L. Iruela-Arispe*, *personal communication*). Further proteolytic processing within this domain has been demonstrated for ADAMTS1, -2, -5, and -12 [3,6,7,19]. For ADAMTS1, this second proteolytic step is mediated by several MMPs, and results in removal of the C-terminal TSRs that interact with the extracellular matrix (ECM). This leads to release of the protein from the endothelial cell membrane, reducing its ability to inhibit endothelial cell proliferation and probably reducing its anti-angiogenic potential [14]. Release of ECM-bound proteins *via *proteolytic removal of their TSR domains may be a common theme, as we see similar proteolytic removal of the C-terminal TSRs of the unrelated neuronal guidance protein F-spondin by plasmin, releasing it from ECM binding [20]. While the exact mechanism of the proteolytic processing of ADAMTS proteins remains somewhat controversial, there is an intriguing possibility that regulation of the ratio of ECM-bound *vs*. free ADAMTS protein could be mediated by MMPs. The region containing these sites is conserved to varying degrees in the newly discovered ADAMTS proteins, suggesting variable (perhaps tissue-specific) MMP processing of these proteins. ADAMTS4, which lacks a TSR tail, may not have an ECM-bound form.

The region between the metalloprotease domain and the TSR repeat tail was demonstrated to be necessary for *gon-1*, a *Caenorhabditis ADAMTS *homolog, to mediate cell migration during gonadogenesis [21]. A variant of this region that lacks the conserved amino acids upstream of the classic TSR but maintains the highly conserved spacer residues is found in papilin, where it has been implicated in influencing cell rearrangements during organogenesis [22] and in the *Manduca sexta *lacunin protein which plays a role in basal lamina remodeling during morphogenesis [23]. It will be interesting to investigate whether there is to be a common theme of organogenesis function among proteins that contain this configuration of domains.

There is evidence that several mammalian ADAMTS proteins are expressed organogenesis. For example, mutations in the mouse *ADAMTS20 *gene have been found to cause the belted white-spotting mutation, resulting from a defect in melanocyte development or migration during embryogenesis [29], the ADAMTS1 protein is necessary for mouse gonadogenesis [30], ADAMTS12 is specifically expressed in fetal lung [7], and several of the newly described ADAMTS proteins [28] are expressed solely or primarily in fetal tissue.

ADAMTS proteins contain a single "classic" TSR motif (WXXWXXW) in the disintegrin-like domain, and a variable number of variant TSRs within the C-terminal tail of the protein, which contain 4 amino acids between tryptophan residues (W4XW) rather than two. TSRs can be divided in several structural groups, based on the presence and spacing of cysteines [24]. The precise function of each type of TSR has not yet been determined, although it is known that the sequence CSVTCG in one of the thrombospondin-1 TSR's mediates endothelial cell apoptosis through binding to CD36 [25,26]. About 70 proteins in the human genome contain TSRs [27] and many of them are matrix binding proteins.

ADAMTS1 and -8 inhibit angiogenesis [31], and gene expression profiling suggests that ADAMTS4 also has a role in angiogenesis [32]. Several of these proteins (ADAMTS1, -4, and -5) have also been shown to cleave aggrecan, the proteoglycan that makes cartilage elastic and compressible [19,33,34], and ADAMTS4 was recently shown to cleave cartilage oligomeric matrix protein (TSP5) [35]. The ADAMTS2, -3, and -14 proteins appear functionally related. ADAMTS3 is a procollagen II N-propeptidase, and ADAMTS14 appears also to be an aminoprocollagen peptidase [36]. ADAMTS2 is an aminoprocollagen peptidase of procollagen I and II, and deficiency of this protein causes Ehlers-Danlos syndrome type VIIC [4]. ADAMTS13 is a von Willebrand factor-cleaving protease. Mutations in the *ADAMTS13 *gene result in inappropriate platelet activation, leading to the blood disorder thrombotic thrombocytopenic purpura (TTP) [37-40].

Recently, an intriguing link has been discovered between ADAMTS metalloproteases. The proinflammatory cytokines IL17 [41], IL1β [42] and TGFβ [43] induce expression of *ADAMTS4*. TGFβ also induces *ADAMTS2 *[44] and TNFα was found to up-regulate *ADAMTS1*, *ADAMTS6*, and *ADAMTS9 *in ocular cells [45], suggesting a role for these proteases in inflammatory eye disease. Similarly, TNFα produced a marked induction of *ADAMTS1 *in endothelial cells [46]. As several ADAMTS proteins, ADAMTS4 in particular, are implicated in rheumatoid arthritis, and TNFα inhibitors have been recently been used with great success in its treatment [47], we speculate that part of the effect of the TNFα inhibitors is an indirect downregulation of the ADAMTS proteins that break down connective tissues. As TNFα inhibitors are not without inherent risks [48,49], transcriptional inhibition of specific *ADAMTS *genes may ultimately provide similar benefits with fewer risks.

To better understand the multiple functions of the ADAMTS proteins, we carried out the most detailed and comprehensive analysis to date of the phylogenetic relatedness and intron/exon organization of all human *ADAMTS *genes, including their comparison with invertebrate and chordate *ADAMTS *homologs. Prior analyses included fewer species and did not address the sequence of genomic events that resulted in the current *ADAMTS *genomic structure [2,28,50]. Our analysis reveals distinct sub-families with unique functions and reveals a history of gene duplications, retrotransposition, and the loss and gain of introns during animal evolution. For example, *ADAMTS1*, *-4*, *-5*, *-8*, and *-15 *genes all derive from a retrotransposition event that occurred prior to the divergence of vertebrates and the urochordate *Ciona intestinalis*, and from subsequent gene duplications that occurred prior to the divergence of mammals and the pufferfish, *Fugu rubripes*. This ADAMTS protein subfamily encompasses proteins that share aggrecanase and angiogenesis-related activities.

## Results & Discussion

### Gene discovery

To perform a phylogenetic analysis of the ADAMTS gene family we first examined several genome databases to have a comprehensive survey of all ADAMTS genes in humans and other species. When we started this analysis in 2002 only 12 ADAMTS proteins were known. We predicted coding sequences of the human *ADAMTS *genes based on several sequence databases (see Material and Methods) and found them to be identical to those subsequently published by Cal, *et al *[28] based on cloned cDNAs. This work confirmed that a total of 19 ADAMTS genes exist in the human genome, with various isoforms. Since there were several cases in which alternative splicing or different exon predictions resulted in ADAMTS proteins of varying lengths, the most complete (longest) translations were considered in our analyses, and their Genbank accession numbers are indicated [Additional File 2]. *ADAMTS9 *has three known splice variants, of which the long variant that we used for analysis was NM_182920. *ADAMTS20 *has two known splice variants, of which we used NM_025003. *ADAMTS18 *has two known variants, of which we used NM_199355, minus the final exon. *ADAMTS13 *has four known variants, of which we used NM_139025.

*ADAMTS10 *and *ADAMTS6 *each have a single known coding sequence, and we found evidence of others. The variant of *ADAMTS6 *that is published (NM_014273) is a short form, and contains a non-consensus exon immediately following the metalloprotease catalytic site, while the variant of *ADAMTS10 *that is published (NM_030957) has a consensus exon at this location, and is in the long form. We predict a long form of *ADAMTS6*, as well as a consensus exon for *ADAMTS6 *and a non-consensus exon for *ADAMTS10*. We used the consensus exons of both genes in our analyses.

### Phylogenetic analyses

**Figure 1:**
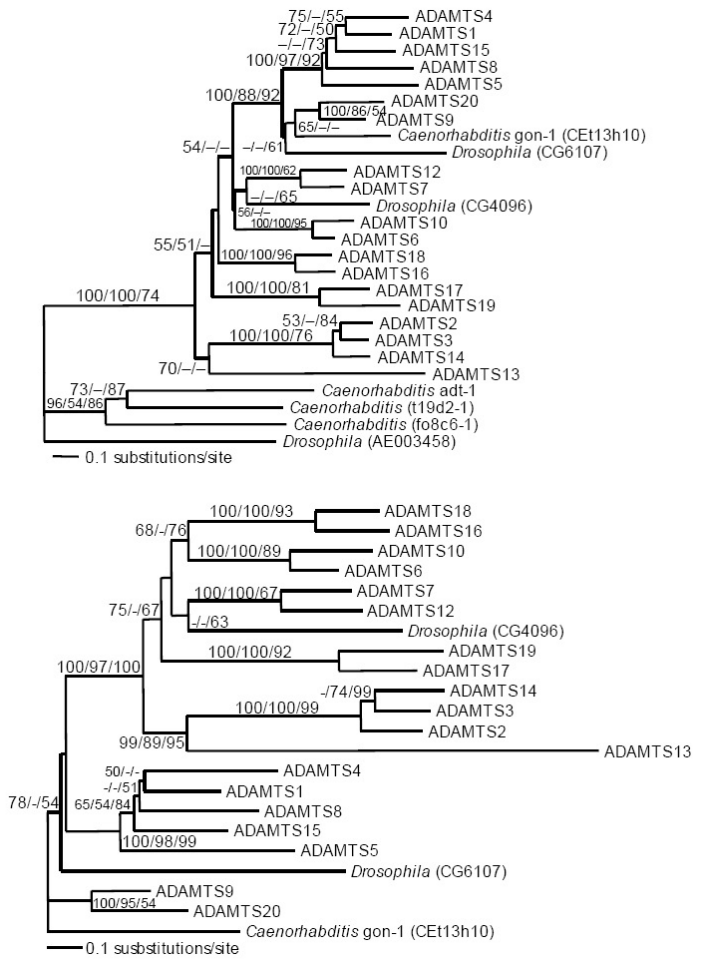
**Phylogenetic analysis of human ADAMTS proteins and invertebrate homologs**. Unambiguously aligned amino acids were analyzed by distance (Protdist+NJ), maximum parsimony (MP) and maximum likelihood (ML) methods. The trees shown are the ML distance topologies. Numbers at the nodes represent the percent of bootstrap replicates of distance (NJ) and parsimony (MP), and the percent of quartet puzzling steps (QP) in support of each group. **(A) Phylogenetic tree of human and distantly related invertebrate ADAMTS homologs **inferred from a 359-amino acid alignment, with α = 1.42 and proportion of invariable sites (pI) = 0.09. **(B) Phylogeny of human and invertebrate ADAMTS homologs with long branches removed, **inferred from 543 aligned amino acids, with α = 1.48 and proportion of invariable sites (pI) = 0.10. For reference, Genbank GI numbers for the sequences are provided [Additional File 2].

**Figure 2:**
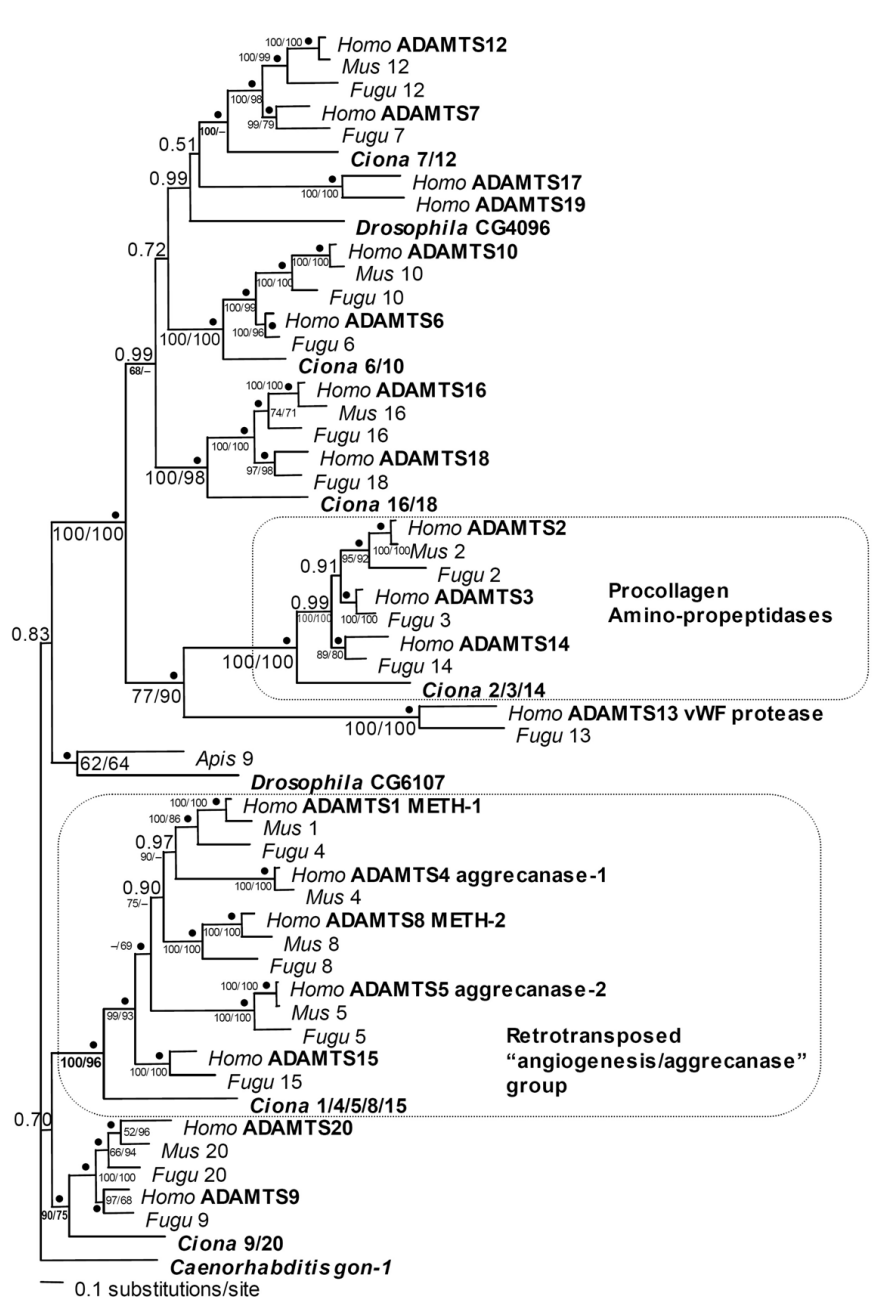
**Phylogenetic analysis of animal ADAMTS homologs. **This is the consensus maximum likelihood tree topology determined from 900 trees with the highest posterior probabilities inferred by Bayesian analysis of protein sequences. 571 aligned amino-acid sites were analyzed, with mean α = 1.59 (1.38 < α < 1.83), pI = 0.10 (0.07 < pI < 0.13) and lnL = -37875.26. Numbers at nodes represent Bayesian posterior probabilities for that relationship, with the best-supported posterior probabilities (1.00) represented by bullets (•). The percent of 1000 bootstrap replicates in support of the nodes, as found by distance and parsimony analyses, are also reported. Accession numbers and scaffold numbers for sequences are provided [Additional File 2].

We compared the 19 known human ADAMTS protein sequences with ADAMTS homologs from invertebrates (*Drosophila *and *Caenorhabditis*) from which entire genome sequences were recently determined, to elucidate their evolutionary relationships (Figure 1A and 1B) [Additional File 1]. This revealed a series of gene duplications among human *ADAMTS *genes, of uncertain affinity to these invertebrate relatives. With the goal to further elucidate this gene duplication history, the human and invertebrate *ADAMTS *orthologs were compared with *ADAMTS *orthologs from *Mus*, *Fugu *and *Ciona*, which diverged between humans and invertebrates, and with an additional invertebrate, the honeybee, *Apis mellifera *(Figure 2) [Additional File 2].

To perform this analysis we inferred the coding sequence of sixteen ADAMTS proteins in the *Fugu rubripes *genome [52], and drew from Genbank nine representative mouse orthologs, three orthologs from the *Drosophila *genome and four from *Caenorhabditis *(see Figures 1A and 2). Other *Mus *(and *Rattus*) *ADAMTS *orthologs that were unannotated at the time of our initial work have since been identified by others [51,53], and some are annotated in the MEROPS database[82]. However, they were not included for our final analyses presented here since they are so similar to the human sequences (69–99% identical, [53]) that they offered no help in elucidating the gene duplication history. The invertebrate genomes were surveyed extensively for additional *ADAMTS *genes, and those most closely related to the vertebrate *ADAMTS *orthologs were retained in the analyses presented herein. The most divergent *Drosophila *and *Caenorhabditis *ADAMTS homologs represented in Figure 1A were removed from further analyses in attempt to avoid systematic bias known as "long branch attraction" where divergent but putatively unrelated sequences group together because of their divergence rather than due to shared characters [54]. All ADAMTS proteins introduced here contain the same basic domain structure as previously described ADAMTS proteins. A complete alignment of all human and invertebrate ADAMTS protein sequences, and representative *Ciona*, *Fugu *and *Mus *orthologs, annotated with intron positions and phases, is available [Additional File 2].

### Intron position and phase

**Figure 3:**
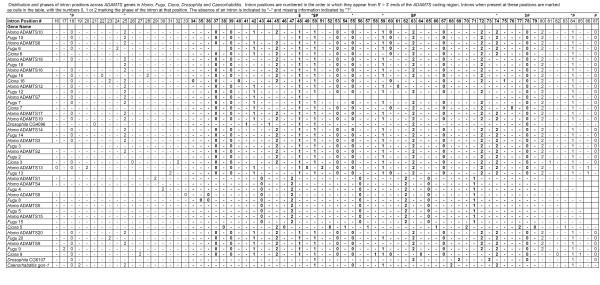
**The phase and position of introns in ADAMTS genes support the phylogeny of ADAMTS proteins. **Intron positions 16 – 87, found in the conserved region of the multiple alignment, are numbered consecutively from the 5'>3' locations in the ADAMTS-coding regions of genes. The presence of an intron is indicated according to its phase (0, 1, 2), absence of an intron indicated by "-" and missing data is blank. Unambiguously aligned intron positions are highlighted in bold, and conserved intron positions shared between chordates and *Drosophila *CG4096 or CG6107 or chordates and *Caenorhabditis *are indicated by $, # or * symbols respectively. The phases and positions of introns summarized here are also individually highlighted in the multiple sequence alignment [Additional File 2].

We compared the positions of introns and their phases between *Homo*, *Fugu*, *Ciona*, *Drosophila *and *Caenorhabditis *homologs of ADAMTS genes, in attempt to corroborate and further elucidate their evolutionary relationships, as shown in Figure 3. The term intron phase refers to the position of the splice site with respect to the codon, where phase 0 describes a splice site 5' of the codon, phase 1 describes a splice site between the first and second base of a codon, and phase 2 describes a splice site between the second and third base of a codon. Introns at the same position that have the same phase in homologous genes are considered to be shared characters that were conserved during evolution. The lack of an intron at a conserved position may either suggest that the gene lost an intron at that position during its evolution, or that it never had that intron, and the intron conserved in the other homologs was gained after those homologs diverged from the intron-lacking homolog. In combination with the phylogenetic analysis of the ADAMTS protein sequences, a parsimonious interpretation of the data summarized in Figure 3 that invokes the fewest changes should help to distinguish between older and more recent gene duplication events in this gene family. Three of our most striking observations of the intron distribution are that (i) some intron positions are shared between worm, fly, chordate and vertebrates, (ii) recently duplicated genes share similar patterns of introns, and that (iii) the complete absence of ancient introns and the presence of introns at new positions in *ADAMTS1, 4, 5, 8 *and *15 *of vertebrates and *Ciona *reveals that this subgroup of genes evolved by retrotransposition early during chordate evolution, was repopulated by new introns (in some cases, separately in vertebrates and *Ciona*), and subsequently underwent gene duplication during the evolution of vertebrates.

**Figure 4:**
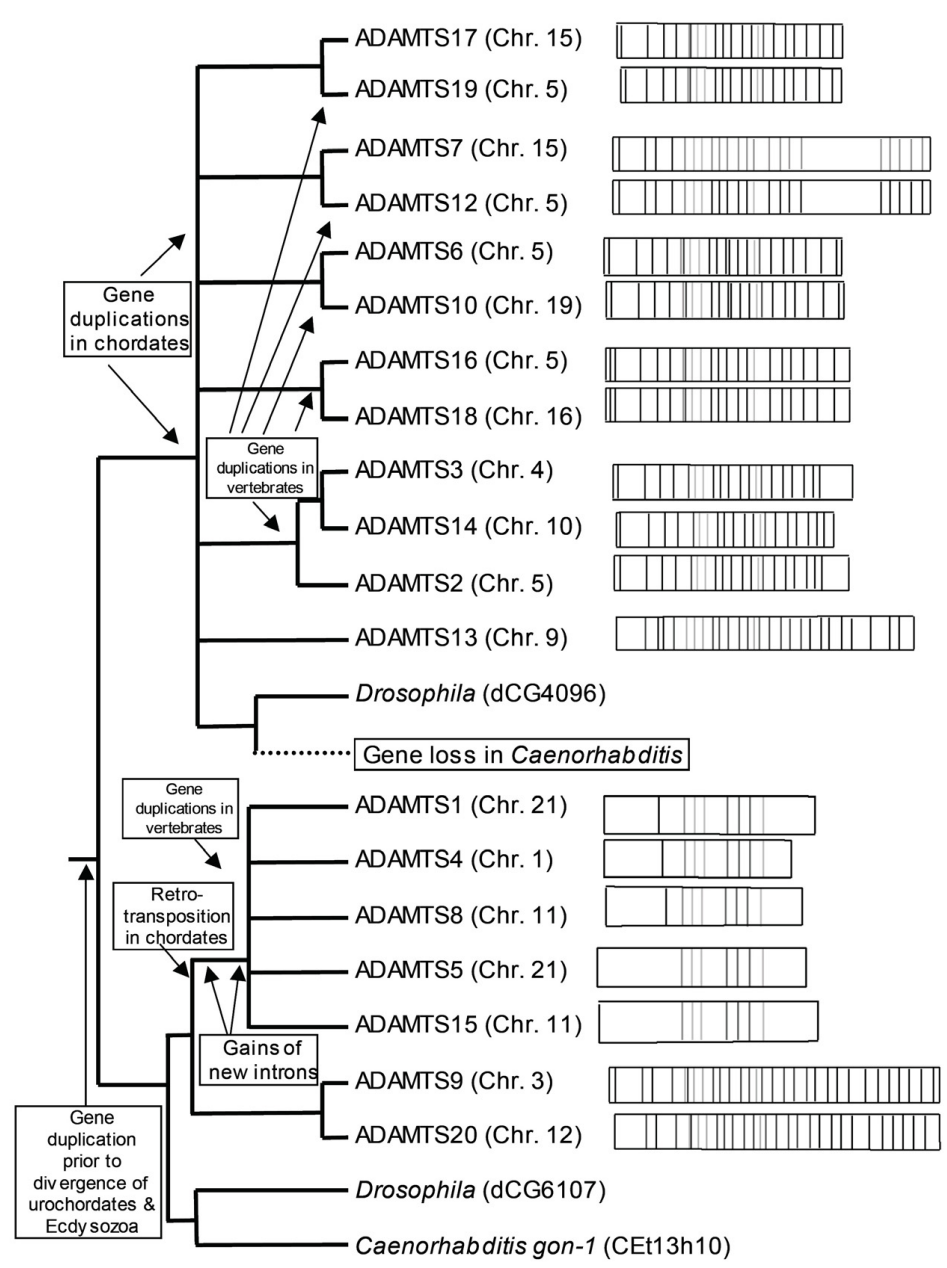
**Proposed scenario for the evolutionary history of ADAMTS proteins. **During chordate evolution a series of gene duplications resulted in six ADAMTS proteins present in the *Ciona *genome, while an early retrotransposition event gave rise to the "angiogenesis clade" of ADAMTS proteins. This proliferation of ADAMTS proteins did not occur in invertebrates, and there is evidence of the loss of one ADAMTS ortholog from *Caenorhabditis*. More recent duplications that occurred early during vertebrate evolution resulted in the paired sets of ADAMTS proteins present in the human genome. The chromosomal locations of the human ADAMTS genes are indicated in parentheses and the exon structure of each human gene is diagrammed to the right of its position in the schematic phylogenetic tree, and shown in more detail in the Additional Files.

The phylogenetic analysis of animal ADAMTS homologs reveals that proteins that are known to have similar biological activities are closely related, and that they have evolved by a series of gene duplication events (Figure 2). Since the functions of only some ADAMTS proteins have been empirically tested, estimates of evolutionary relatedness amongst the entire family may imply closer functional relatedness, and thus guide the future design of more specific empirical tests of protein functions. An interesting property of the vertebrate ADAMTS proteins are the extensive sequence similarity between many pairs of sequences, as indicated in Figures 1, 2, 3, 4 [and Additional file 2]. Although in many cases little is known about the functions of these proteins, we can speculate that the two proteins in each pair may share similar biological activities due to their shared primary sequence. It is also possible that these ADAMTS proteins act as heterodimers, in a manner similar to the ADAM proteins fertilin α and β [55].

As shown in Figures 1 and 2 and summarized in Figure 4, animal *ADAMTS *homologs have undergone several gene duplications. Assuming that the Ecdysozoa hypothesis is true and arthropods and nematodes are united as a group [56-62], our results indicate that a single *ADAMTS *gene duplication preceded the divergences of Ecdysozoa and chordates. At least 3–4 *ADAMTS *gene duplications occurred in chordates prior to the divergence of *Ciona *and vertebrates, followed by additional gene duplications in vertebrates prior to the divergence of *Fugu *and mammals (Figures 2 and 4).

*Ciona intestinalis*, the urochordate sea squirt, was found to have at least six *ADAMTS *genes (Figure 2), which correspond to six of the seven major groups of vertebrate ADAMTS homologs. *Ciona *ADAMTS6 is the sister of the group comprised of vertebrate ADAMTS6 and -10, indicating that ADAMTS6 and -10 evolved by gene duplication early during vertebrate evolution, preceding the divergence of pufferfish and mammals, but after their divergence from urochordates. Similarly, *Ciona *ADAMTS16 is a sister to the group comprised of vertebrate ADAMTS16 and -18, *Ciona *ADAMTS7 is a sister to the group comprised of vertebrate ADAMTS7 and -12, *Ciona *ADAMTS3 is a sister to the group comprised of vertebrate ADAMTS2, -3 and -14, *Ciona *ADAMTS9 is a sister to the group comprised of vertebrate ADAMTS9 and -20 and *Ciona *ADAMTS15 is the sister to the group comprised of vertebrate ADAMTS1, -4, -5, -8 and -15. This reveals that both gene duplications early in chordate evolution as well as subsequent gene duplications early during vertebrate evolution have contributed to the proliferation of ADAMTS genes studied in growing depth in mammalian model systems.

*ADAMTS2*, *-3*, and *-14 *have been recognized as evolutionarily closely related genes, encoding proteins with a common functionality as procollagen aminopeptidases. They are as a group most closely related to a single gene in *Ciona*, and appear to have evolved by gene duplications that occurred prior to the divergence of pufferfish and mammals but after the divergence of urochordates and vertebrates. They are most closely related to *ADAMTS13*, suggesting a gene duplication from a common ancestor (Figures 1 and 2). *ADAMTS13 *appears to have originated early in vertebrate evolution as it has a closely related homolog in the pufferfish *Fugu rubripes *but is apparently absent in *Ciona*, fly and worm genomes. The pufferfish *ADAMTS13 *homolog is not only closely related at the amino acid sequence level, but also has the same *ADAMTS13*-specific intron/exon structure in its tail, which is unique among the *ADAMTS *gene family. This is in agreement with a unique function for ADAMTS13 as a protease cleaving von Willebrand factor, leading to abnormal blood clotting. Although the mouse *ADAMTS13 *gene was not included in this analysis, it has been identified (Genbank accession number NM_001001322).

A second evolutionarily related group is comprised of *ADAMTS1*, *-4*, *-5*, *-8 and -15 *and their single sister in *Ciona*. Vertebrate members of this group share unique intron positions and lack all of the intron positions held by other *ADAMTS *genes and their invertebrate homologs (Figure 3). Three members of this group (*ADAMTS1*, *-4*, *and -8*) encode proteins with aggrecanase and angiogenesis-related functions, which suggests the examination of ADAMTS5 and -15 for similar biological activities. This putative "angiogenesis/aggrecanase group" appears most closely related to *ADAMTS20 *and *-9*. Further, the unique intron positions shared by *ADAMTS1*, *-4*, *-5*, *-8*, and *-15*, and lack of invertebrate orthologs in this putative "angiogenesis/aggrecanase group" suggest that this group's progenitor arose within chordates *via *a retrotransposition event from the common ancestor of the group comprised by *ADAMTS20 *and *-9 *(Figures 2 and 4). The intron/exon structures of *ADAMTS1*, *-4*, *-5*, *-8*, and *-15 *are similar to that of the mouse *ADAMTS1 *gene [63], and our analysis shows four *ADAMTS *genes with this characteristic gene structure in the genome of *F. rubripes*. Therefore, retrotransposition of an ancestor of the angiogenesis/aggrecanase subfamily of genes, its acquisition of new introns, and subsequent gene duplications that produced five related genes occurred prior to the divergence of human, mouse and pufferfish lineages. In at least one case (intron 17 in *ADAMTS8*) we see evidence of acquisition of a new intron following the process of duplication, but prior to the divergence of mammals and pufferfish.

*ADAMTS20 *and *-9 *are most closely related and, along with the members of the angiogenesis/aggrecanase clade, are most closely related to invertebrate *gon-1 *(*Caenorhabditis*) and CG6107 (*Drosophila*). The finding that *ADAMTS9 *and *-20 *together as a group have a single sister gene in *Ciona *indicates that they evolved by gene duplication in the vertebrate lineage, after their divergence from urochordates. However, the results of our phylogenetic analyses demonstrate that neither *ADAMTS9 *nor *ADAMTS20 *can alone be rightfully dubbed as being orthologous to *gon-1*, as has been recently proposed [64,65]. In fact, our analyses suggest that while *gon-1 *and CG6107 are likely orthologs, the chordate ortholog of these genes was the common ancestor of *Ciona ADAMTS9 *and *-15*, *i.e. *also the common ancestor of the later-duplicated vertebrate ADAMTS genes *ADAMTS9*, *-20*, *-15*, *-5*, *-8*, *-4*, and *-1 *(Figures 1 and 2).

Only one invertebrate sequence (*Drosophila *CG4096) was found that grouped with the remainder of the human *ADAMTS *homologs. The placement of this gene with or within a group of these remaining *ADAMTS *genes would suggest the number of gene duplication events in this gene family that occurred prior to the divergence of vertebrates and invertebrates from a common ancestor. The Kishino-Hasegawa test revealed that the likelihood of *Drosophila *CG4096 being most closely related to an ancestor of the group of all remaining mammalian ADAMTS proteins, or of the various groups nested within it, was not significantly different from the likelihood that *Drosophila *CG4096 is most closely related to *ADAMTS 7 *and *-12 *(data not shown). The most parsimonious explanation of this result is that a single gene duplication of an ancient ADAMTS homolog occurred early during the evolution of animals, prior to the divergence of chordates from invertebrates, followed by lineage-specific gene loss and gene duplications (Figure 4). If this scenario is correct, the *Caenorhabditis *ortholog of *Drosophila *CG4096 has been subsequently lost, but the vertebrate ortholog has been retained and underwent several gene duplications within and among chromosomes in the vertebrate lineage (*ADAMTS2*, *-3*, *-6*, *-7*, *-10*, *12–14*, *16–19*).

The exons comprising the TSR tail each contain a single variant TSR, with the C(S/T)XCG motif 5' and the W4XW motif at the 3' end. This exon structure may facilitate the formation of alternately spliced isoforms, such as we describe here, but it would also lend itself to duplication or loss of individual repeats. However, the number of variant TSRs in the tails of these proteins has been conserved for the gene pairs *ADAMTS17 *and *-19*, *ADAMTS6 *and *-10*, and *ADAMTS18 *and *-16 *(Figure 4). While this may suggest a series of relatively recent gene duplications, a more likely explanation is that each TSR has an important and non-redundant role, or that the presence of a specific number of TSRs is critical for each protein's function.

The apparent absence of any ortholog of *ADAMTS17 *or *-19 *in the pufferfish genome (Figure 2), but their presence in *Mus *and *Rattus *[51] suggests that this gene duplication either occurred in mammals after they diverged from fish, or that *ADAMTS17 *or *-19 *evolved earlier than the mammal/fish divergence but were lost in *Fugu*.

Gene duplication is a common way by which new genes with similar functions may evolve. In fact, duplication of large segments of chromosomes has been a common occurrence during animal evolution [66,67]. Both the phylogenetic trees and the intron/exon structures of *ADAMTS *genes show a history of such duplications. In addition to the similarity in intron positions of *ADAMTS-1*, *-4*, *-5*, *-8*, and *-15 *in both mouse and pufferfish, human *ADAMTS1 *and *-5 *are located in tandem on chromosome 21, and human *ADAMTS8 *and *-15*, on chromosome 11 (Figure 3). The remaining genes have additional introns at conserved locations, and both proteins in each set of pairs have the same intron/exon structure.

Thus, by combining the phylogenetic analysis and intron/exon structure determinations, we are able to propose the following series of events leading to the ADAMTS protein family (Figure 4): (i) The ancestral *ADAMTS *gene duplicated prior to the divergence of the ecdysozoan and chordate lineages, approximately 673 million years ago [68]. (ii) In the following approximately 250 million years prior to divergence of fish and mammals [69], multiple gene duplications occurred. (iii) A retrotransposition of the common ancestor of the *ADAMTS9 *and *-20 *gene pair resulted in an intronless gene that proceeded to gain multiple introns, giving rise to the angiogenesis/aggrecanase clade. (iv) This gene was involved in a duplicative chromosomal inversion, and later a duplication of the chromosomal segment containing both *ADAMTS *genes. (v) Another intron was gained, in *ADAMTS8*, prior to the divergence of the pufferfish and mammalian lineages. In the other branch of the *ADAMTS *family, we see a remarkable frequency of genes located on chromosome 5, suggesting it as the location of the ancestor of these genes. We can speculate that a similar scenario of within-chromosome duplication followed by duplication of chromosomal segments took place, although none are in as close physical proximity as the *ADAMTS1*-subfamily genes.

## Conclusions

This comprehensive bioinformatic survey of the human genome affirms the widely held belief, derived from experimental work, that the nineteen known human ADAMTS proteins constitute the complete gene family. By examining both the amino acid sequences using rigorous phylogenetic methods and comparing the exon structure of these proteins, we were able to draw conclusions about the evolutionary history of this family of proteins which, in turn, provides a framework for further analysis of the functions of these clinically-relevant genes.

## Methods

### Gene discovery

Sequences of *ADAMTS *homolog sequences presented here were identified from 2001 to 2004 using genomic sequences cataloged in the NCBI, JGI (*Fugu *and *Ciona *[52,70]) and Celera genome databases using BLASTp, BLASTn, BLASTx, tBLASTn and tBLASTx searches [71]. We searched databases of Expressed Sequence Tags (ESTs) for all human genes and, with the exception of *ADAMTS15*, found ESTs that corresponded to those genes, confirming their transcription. Splice site predictions were made by neural network *via *the Berkeley Drosophila Genome Project and also by eye using Sequencher 4.2 (Genecodes), with reference to protein multiple sequence alignments. We used the same method to identify *Caenorhabditis elegans*, *Drosophila melanogaster*, and *Fugu rubripes *homologs of *ADAMTS *genes, and their genomic structure. Multiple sequence alignments of the inferred amino acid sequences were prepared using Multalin [72] and ClustalX1.81 [73] and manually refined and annotated within BioEdit [74] and MacClade4.06 [75].

### Phylogenetic analysis

Initial phylogenetic analyses were conducted including all of the human ADAMTS proteins along with seven invertebrate homologs, three from *Drosophila melanogaster *and four from *Caenorhabditis elegans *(Figure 1A), and then these analyses were repeated with the most divergent invertebrate sequences removed (Figure 1B). The datasets for these analyses were comprised of 359 and 543 unambiguously aligned amino acid sites, respectively. The alignments were analyzed using parsimony and distance methods. Parsimony analyses used a heuristic search with random stepwise addition of data and tree-bisection-reconnection in PAUP*4.0b10 for 1000 bootstrap replicates [76]. Distance matrices were inferred using the Jones, Taylor, Thornton (JTT) substitution model with PROTDIST in PHYLIP 3.6a3 [77,78]. Neighbor-joining trees were constructed with the input order jumbled for 1000 bootstrap replicates using NEIGHBOR, SEQBOOT and CONSENSE in PHYLIP. Using Tree-Puzzle 5.0 [79] we generated maximum-likelihood distance matrices in which among-site substitution rate heterogeneity was corrected using an invariable and eight gamma-distributed substitution rate categories and the JTT model. 10,000 quartet-puzzling steps were also used (Tree-Puzzle) to assess branch support.

To better resolve the evolutionary relationships of the vertebrate *ADAMTS *subfamily members and to provide information on their presence in other chordate lineages, sequences from *Mus*, *Fugu *and *Ciona *were identified and added to the alignment and phylogenetic analysis. An annotated ADAMTS homolog from the honeybee *Apis mellifera *was also included in the analysis as another representative invertebrate. 571 unambiguously aligned amino acid sites of *ADAMTS*-homologous sequences encoded by *Homo*, *Mus*, *Fugu*, *Ciona*, *Apis*, *Drosophila *and *Caenorhabditis *were analyzed (Figure 2). MrBayes3.0 [80] was used to construct a maximum likelihood (ML) phylogenetic tree from this protein alignment. MrBayes was run for 1000000 generations, with four incrementally heated Markov chains, and a sampling frequency of 1000 generations. The temperature setting was increased to 0.5. Among-site substitution rate heterogeneity was corrected using an invariable and eight gamma-distributed substitution rate categories and the WAG model for amino acid substitutions [81], abbreviated herein as WAG+I+8Γ. The consensus ML tree topology, the arithmetic mean log-likelihood (lnL) for this topology, and branch support were estimated from the set of sampled trees with the best posterior probabilities. Means and 95% confidence intervals for the gamma distribution shape parameter α and the proportion of invariable sites (pI) were also estimated.

## List of abbreviations

ADAMTS (**A D**isintegrin-like and **M**etalloprotease with **T**hrombo**s**pondin motifs)

ADAM (**A D**isintegrin-like and **M**etalloprotease)

TSR (**T**hrombospondin type 1 **S**equence **R**epeat)

MMP (**m**atrix **m**etallo**p**rotease)

ECM (**e**xtra**c**ellular **m**atrix)

SAGE (**s**erial **a**nalysis of **g**ene **e**xpression)

TTP (**t**hrombotic **t**hrombocytopenic **purpura**)

IL (**i**nter**l**eukin)

TGF (**t**ransforming **g**rowth **f**actor)

TNF (**t**umor **n**ecrosis **f**actor)

BLAST (**B**asic **l**ocal **a**lignment **s**earch **t**ool)

## Authors' contributions

ACN and EGVM initiated the project and all authors were involved in the design phases. ACN, SBM and JML inferred sequences of previously un-annotated ADAMTS genes from Genbank, Celera, and JGI, and determined intron positions. SBM performed the phylogenetic analyses. ACN and SBM drafted the manuscript and JML and EGVM contributed to writing the paper and advised throughout.

## Supplementary Material

Additional File 2 **Alignment used for phylogenetic analyses of animal ADAMTS homologs. **Unambiguously aligned amino acid sites, indicated by the "mask" line in the alignment, were used for phylogenetic analyses (Figure 2). Accession (GI) numbers for sequences are provided. Intron positions in the corresponding genomic sequence are indicated, with color-coding for intron phases (Figure 3). Click here for file

Additional File 1 **Alignment used for phylogenetic analyses of human ADAMTS proteins and invertebrate homologs. **Unambiguously aligned amino acid sites, indicated by the "mask" line in the alignment, were used for phylogenetic analyses (figure 1). Intron positions in the corresponding genomic sequence are indicated, with color-coding for intron phases (Figures 3 and 4). Click here for file
