# Supplementary material for: Functional evolution of ADAMTS genes: Evidence from analyses of phylogeny and gene organization
Source: BMC Evol Biol. 2005 Feb 4;5:11. doi: 10.1186/1471-2148-5-11 (PMC549037; doi:10.1186/1471-2148-5-11)
Supplement: Additional File 2 — Alignment used for phylogenetic analyses of animal ADAMTS homologs. Unambiguously aligned amino acid sites, indicated by the "mask" line in the alignment, were used for phylogenetic analyses (Figure 2). Accession (GI) numbers for sequences are provided. Intron positions in the corresponding genomic sequence are indicated, with color-coding for intron phases (Figure 3). [file 1471-2148-5-11-S2.pdf]

Additional file #2 for:

Research Article

Functional evolution of ADAMTS genes: Evidence from analyses of phylogeny and gene organization

Ainsley C. Nicholson[1,3*], Shehre-Banoo Malik[2*], John M. Logsdon Jr.[2] and Erwin G. Van Meir[1§]

[1]Laboratory of Molecular Neuro-Oncology, Winship Cancer Institute, 1365-C Clifton Road, Room C5078, Emory University, Atlanta GA 30322 USA. Phone: 404-778-5563, Fax: 404-778-5240.
[2]Roy J. Carver Center for Comparative Genomics, Department of Biological Sciences, 300 Old Biology Building, University of Iowa, Iowa City IA 52242-1324 USA. Phone: 319-335-1083, Fax: 319-335-1069
[3]Present address: Viral Exanthems and Herpesvirus Branch, Centers for Disease Control and Prevention, 1600 Clifton Rd, Mailstop A14, Atlanta, GA 30333 USA.

* These authors contributed equally to this work

§ Corresponding author

Email addresses:

ACN: agn0@cdc.gov
SBM: banoo-malik@uiowa.edu
JML: john-logsdon@uiowa.edu
EGVM: evanmei@emory.edu

Additonal File 2 – **(NMLVadamtsalign2.pdf) Alignment used for phylogenetic analyses of animal ADAMTS homologs.** Unambiguously aligned amino acid sites, indicated by the "mask" line in the alignment, were used for phylogenetic analyses (**Figure 2**). Accession (GI) numbers for sequences are provided. Intron positions in the corresponding genomic sequence are indicated, with color-coding for intron phases (**Table 1**).

```
#NEXUS
[AC Nicholson, SB Malik, JM Logsdon Jr, E van Meir, 2004. Functional evolution of ADAMTS genes: evidence from]
[analyses of phylogeny and gene organization. Contact: john-logsdon@uiowa.edu]


BEGIN DATA;
        DIMENSIONS  NTAX=56 NCHAR=2875;
        FORMAT DATATYPE=PROTEIN  SYMBOLS = " 1 2 3 4"  MISSING=? GAP=-  INTERLEAVE ;
MATRIX

[                                                 10        20        30        40        50        60]
[                                                 .         .         .         .         .         .]

mask                                         ------------------------------------------------------------   [0]
Homo ADAMTS6 (11131380)                      ------------------------------------------------------------   [0]
Fugu 6                                       ------------------------------------------------------------   [0]
Ciona 6 (0100146470)                         ------------------------------------------------------------   [0]
Homo ADAMTS10 (17432918)                     ------------------------------------------------------------   [0]
Mus 10 (27502095)                            ------------------------------------------------------------   [0]
Fugu 10                                      ------------------------------------------------------------   [0]
Homo ADAMTS16 (21265061)                     ------------------------------------------------------------   [0]
Mus 16 (21961374)                            ------------------------------------------------------------   [0]
Fugu 16                                      ------------------------------------------------------------   [0]
Ciona 16 (0100138085)                        ------------------------------------------------------------   [0]
Homo ADAMTS18 (21265067)                     ------------------------------------------------------------   [0]
Fugu 18                                      ------------------------------------------------------------   [0]
Homo ADAMTS12 (13569928)                     ------------------------------------------------------------   [0]
Mus 12 (27817773)                            ------------------------------------------------------------   [0]
Fugu 12                                      ------------------------------------------------------------   [0]
Homo ADAMTS7 (11131377)                      ------------------------------------------------------------   [0]
Fugu 7                                       ------------------------------------------------------------   [0]
Ciona 7 (genewise.34.12.1)                   ------------------------------------------------------------   [0]
Homo ADAMTS17 (21265064)                     ------------------------------------------------------------   [0]
Homo ADAMTS19 (19525737)                     ------------------------------------------------------------   [0]
Drosophila dCG4096 (22831757)                ------------------------------------------------------------   [0]
Homo ADAMTS14 (29337086)                     ------------------------------------------------------------   [0]
Fugu 14                                      ------------------------------------------------------------   [0]
Homo ADAMTS3 (2224673)                       ------------------------------------------------------------   [0]
Fugu 3                                       ------------------------------------------------------------   [0]
Ciona 3 (0100146117)                         ------------------------------------------------------------   [0]
Homo ADAMTS2 (7656867)                       ------------------------------------------------------------   [0]
Mus 2 (28204840)                             ------------------------------------------------------------   [0]
Fugu 2                                       ------------------------------------------------------------   [0]
Homo ADAMTS13 (16306598)                     ------------------------------------------------------------   [0]
Fugu 13                                      ------------------------------------------------------------   [0]
Homo ADAMTS1 (6525075)                       ------------------------------------------------------------   [0]
Mus 1 (1813340)                              ------------------------------------------------------------   [0]
Homo ADAMTS4 (12643637)                      ------------------------------------------------------------   [0]
Mus 4 (26350615)                             ------------------------------------------------------------   [0]
Fugu 4                                       ------------------------------------------------------------   [0]
Homo ADAMTS8 (21536392)                      ------------------------------------------------------------   [0]
Mus 8 (6708110)                              ------------------------------------------------------------   [0]
Fugu 8                                       ------------------------------------------------------------   [0]
Homo ADAMTS5 (7768707)                       ------------------------------------------------------------   [0]
Mus 5 (5923784)                              ------------------------------------------------------------   [0]
Fugu 5                                       ------------------------------------------------------------   [0]
Ciona 15 (0100137065)                        ------------------------------------------------------------   [0]
Homo ADAMTS15 (19171175)                     ------------------------------------------------------------   [0]
Fugu 15                                      ------------------------------------------------------------   [0]
Homo ADAMTS9 (27463365)                      ------------------------------------------------------------   [0]
Fugu 9                                       ------------------------------------------------------------   [0]
Homo ADAMTS20 (28460690)                     ------------------------------------------------------------   [0]
Mus 20 (29500513)                            ------------------------------------------------------------   [0]
Fugu 20                                      ------------------------------------------------------------   [0]
Ciona 9 (0100132719)                         ------------------------------------------------------------   [0]
Apis 9                                       ------------------------------------------------------------   [0]
Drosophila CG6107 (20151419)                 ---------------------------MSMPDAGSLKSPAGGQEVECHRLLSDLPAFAYP   [33]
Caenorhabditis gon-1 CEt13h10 (3879882)      ------------------------------------------------------------   [0]
introns                                      ------------------------------------------------------------   [0]
```

[	70 80 90 100 110 120]	
[	. . . . . .]	
mask	--	[0]
Homo ADAMTS6 (11131380)	--	[0]
Fugu 6	--	[0]
Ciona 6 (0100146470)	--	[0]
Homo ADAMTS10 (17432918)	--	[0]
Mus 10 (27502095)	--	[0]
Fugu 10	--	[0]
Homo ADAMTS16 (21265061)	---MKPRA	[5]
Mus 16 (21961374)	--	[0]
Fugu 16	--	[0]
Ciona 16 (0100138085)	--	[0]
Homo ADAMTS18 (21265067)	--MECALLLACA	[10]
Fugu 18	--	[0]
Homo ADAMTS12 (13569928)	--	[0]
Mus 12 (27817773)	--	[0]
Fugu 12	--	[0]
Homo ADAMTS7 (11131377)	--	[0]
Fugu 7	--	[0]
Ciona 7 (genewise.34.12.1)	--	[0]
Homo ADAMTS17 (21265064)	--	[0]
Homo ADAMTS19 (19525737)	--MRLTHICCCCLLYQLGFL	[18]
Drosophila dCG4096 (22831757)	--	[0]
Homo ADAMTS14 (29337086)	--	[0]
Fugu 14	--	[0]
Homo ADAMTS3 (2224673)	--	[0]
Fugu 3	--	[0]
Ciona 3 (0100146117)	--	[0]
Homo ADAMTS2 (7656867)	--MDPPAG	[6]
Mus 2 (28204840)	--MDPPAG	[6]
Fugu 2	--	[0]
Homo ADAMTS13 (16306598)	--	[0]
Fugu 13	--	[0]
Homo ADAMTS1 (6525075)	--	[0]
Mus 1 (1813340)	--	[0]
Homo ADAMTS4 (12643637)	--	[0]
Mus 4 (26350615)	--	[0]
Fugu 4	--	[0]
Homo ADAMTS8 (21536392)	--	[0]
Mus 8 (6708110)	--	[0]
Fugu 8	--	[0]
Homo ADAMTS5 (7768707)	--ML	[2]
Mus 5 (5923784)	--	[0]
Fugu 5	--	[0]
Ciona 15 (0100137065)	--	[0]
Homo ADAMTS15 (19171175)	--	[0]
Fugu 15	--	[0]
Homo ADAMTS9 (27463365)	---MQF	[3]
Fugu 9	--	[0]
Homo ADAMTS20 (28460690)	--	[0]
Mus 20 (29500513)	--	[0]
Fugu 20	--	[0]
Ciona 9 (0100132719)	MKTTTSSSGLTVILFAILYLWNFVGCTFPSNSDTSRFSFNHRTSEEPLTNKAYFDVDPSK	[60]
Apis 9	--	[0]
Drosophila CG6107 (20151419)	STTLRSTTSGHSTGTNSTTVCESPGARDRTSFACSSSCVSSACSATASDDDEDERALERC	[93]
Caenorhabditis gon-1 CEt13h10 (3879882)	--	[0]
introns	--	[0]

```
[                                                     130       140       150       160       170       180]
[                                                     .         .         .         .         .         .]

mask                                          ------------------------------------------------------------   [0]
Homo ADAMTS6 (11131380)                       --MEILWKTL-TWILSLI-MAS----------------------SEFHSDHRLSYSSQEE   [34]
Fugu 6                                        --MEILWKTL-TWILSLVVMAA----------------------SEFQSFNRLSHNSQEE   [35]
Ciona 6 (0100146470)                          --MYLRRV------------------------------------SINKTELLLRNFEELS   [22]
Homo ADAMTS10 (17432918)                      --MAPACQIL-RWALALGLG------------------------LMFEVTH--AFRSQDE   [31]
Mus 10 (27502095)                             ------------------------------------------------------------   [0]
Fugu 10                                       -----------------------------------------------------------A   [1]
Homo ADAMTS16 (21265061)                      RGWRGLAALW-MLLAQVAEQAPACAM---------GPA---AAAPGSPSVPRPPPPAERP   [52]
Mus 16 (21961374)                             ------------------------------------------------------------   [0]
Fugu 16                                       ------------------------------------------------------------   [0]
Ciona 16 (0100138085)                         ------------------------------------------------------------   [0]
Homo ADAMTS18 (21265067)                      FPAAGSGPPRGLAGLGRVAKA--------------LQL---CCLCCASVAAALASDSSSG   [53]
Fugu 18                                       ------------------------------------------------------------   [0]
Homo ADAMTS12 (13569928)                      --MPCAQRSW-LANLSVVAQLLNFGA-LCYG---------------------RQPQPGPV   [35]
Mus 12 (27817773)                             ------------------------------------------------------------   [0]
Fugu 12                                       -------------------------------MVGLFLH---SSGSTCLHHIRSLRFHPAG   [26]
Homo ADAMTS7 (11131377)                        --MPGGPSPR-SPAPLLRPLLLLLCA-LAPG---------------------APGPAPGR   [35]
Fugu 7                                        -----------------------------------------------------------R   [1]
Ciona 7 (genewise.34.12.1)                    ------------------------------------------------------------   [0]
Homo ADAMTS17 (21265064)                      -----------------------------MCDGALLPP---LVLPVLLLLVWGLDPGTAV   [28]
Homo ADAMTS19 (19525737)                      SNGIVSELQFAPDREEWEVVFPALWRREPVDPAGGSGG---SADPGWVRGVGGGGSARAQ   [75]
Drosophila dCG4096 (22831757)                 ------MWIR-SRWLGLLLHYWLVVV-TQGGGVRPLYG---LHSDELVAGEGQLVVPRRV   [49]
Homo ADAMTS14 (29337086)                      -------MAP-LRALLSYLLPLHCAL-CA-------------------AAGSRTPE---L   [29]
Fugu 14                                       ------------------------------------------KLSGKLSEYGLIVPFSTD   [18]
Homo ADAMTS3 (2224673)                        -----MVLLS-LWLIAAALVEVRTSA-DG----------------QAGNEEMVQID---L   [34]
Fugu 3                                        ------------------------------------------------------------   [0]
Ciona 3 (0100146117)                          ------------------------------------------------------------   [0]
Homo ADAMTS2 (7656867)                        AARRLLCPAL-LLLLLLLPPPLLPPP-PP-------------PANARLAAAADPPG---G   [48]
Mus 2 (28204840)                              AARRLLCPAL-LLLLLPPPPLLLLPP-P--------------PASVRLVAATEPPG---G   [47]
Fugu 2                                        ------------------------------------------------------------   [0]
Homo ADAMTS13 (16306598)                      ------------------------------------------------------------   [0]
Fugu 13                                       ------------------------------------------------------------   [0]
Homo ADAMTS1 (6525075)                        MQRAVPEGFG-RRKLGSDMGNAERAP-GS----------------RSFGPVPTLLLLAAA   [42]
Mus 1 (1813340)                               ------------------------------------------------------------   [0]
Homo ADAMTS4 (12643637)                       -MSQTGSHPG-RGLAGRWLWGAQPCL-LLP---------------IVPLSWLVWLLLLLA   [43]
Mus 4 (26350615)                              ------------------------------------------------------------   [0]
Fugu 4                                        ------------------------------------------------------------   [0]
Homo ADAMTS8 (21536392)                       ------------------MFPAPAAP--------------------RWLPFLLLLLLLLP   [23]
Mus 8 (6708110)                               ------------------------------------------------------------   [0]
Fugu 8                                        -----------------------------------------------------MRSWPLV   [7]
Homo ADAMTS5 (7768707)                        LGWASLLLCA-FRLPLAAVGPAATPA-QDK--------------AGQPPTAAAAAQPRRR   [46]
Mus 5 (5923784)                               ------------------------------------------------------------   [0]
Fugu 5                                        -----------------------------------------------------------F   [1]
Ciona 15 (0100137065)                         ------------------------------------------------------------   [0]
Homo ADAMTS15 (19171175)                      -----------------------------------------------------MLLLGIL   [7]
Fugu 15                                       -------------------------------------------------------QPAAG   [5]
Homo ADAMTS9 (27463365)                       VSWATLLTLL-VRDLAEMGSPDAAAA-VRKDR---------------------LHPRQVK   [40]
Fugu 9                                        ------------------------------------------------------------   [0]
Homo ADAMTS20 (28460690)                      -----------MRNAWELTGPVAVVA-EA--------------------LVRTLTSYEVV   [28]
Mus 20 (29500513)                             ------------------------------------------------------------   [0]
Fugu 20                                       -----------------------------------------------------------S   [1]
Ciona 9 (0100132719)                          LFTKNQHREDQNRNTRKITSKVEENL--AHGFDEN----------------------EVV   [96]
Apis 9                                        ------------------------------------------------------------   [0]
Drosophila CG6107 (20151419)                  LRGTTDDLGESGLRYSLKGPPPELYSAKKDF-ISKGPL---LGRQLEVKKRCEWWCHKYI   [149]
Caenorhabditis gon-1 CEt13h10 (3879882)       -----------------------MRS-IGGSFHLLQPV---VAALILLVVCLVYALQSGS   [33]
introns                                       -------1------------2---3----------------4-----------------5   [2]
```

```
[                                                        190       200       210       220       230       240]
[                                                          .         .         .         .         .         .]

mask                                          ------------------------------------------------------------   [0]
Homo ADAMTS6 (11131380)                       FLTYLEHYQLTIPIRVDQNGAFLSFTVKN---DKHSRRRRSMDPI---------------   [76]
Fugu 6                                        FLSYLQHYQLTVPVRVDENGEFLSYTVKHHRPGRRRRGAGDPVVGP--------------   [81]
Ciona 6 (0100146470)                          KASSEATIHTIRPQRIDRNGGFIEFATLDPISSRSGGKLRKRRSVEATNVLDTLDAAA--   [80]
Homo ADAMTS10 (17432918)                      FLSSLESYEIAFPTRVDHNGALLAFSPPP---PRRQRRG---------------------   [67]
Mus 10 (27502095)                             ------------------------------------------------------------   [0]
Fugu 10                                       FLSSLGQYEIAIPVRIGPHGETLDAE----------------------------------   [27]
Homo ADAMTS16 (21265061)                      GWMEKGEYDLVSAYEVDHRGDYVSHEIMH---HQRRRRAV--------------------   [89]
Mus 16 (21961374)                             ------------------------------------------------------------   [0]
Fugu 16                                       -YEIVSPYEVTHQGHYISHEVGHHHRRRRRSVTPGAFSTIN-------------------   [40]
Ciona 16 (0100138085)                         ------------------------------------------------------------   [0]
Homo ADAMTS18 (21265067)                      ASGLNDDYVFVTPVEVDSAGSYISHDILH-NGRKKRS-----------------------   [89]
Fugu 18                                       ------------------------------------------------------------   [0]
Homo ADAMTS12 (13569928)                      RFPDRRQEHFIKGLPEYHVVGPVRVDASGHFLSYGLHYPITSSRRKRD------------   [83]
Mus 12 (27817773)                             ------------------------------------------------------------   [0]
Fugu 12                                       HFQPGSDLSIVHPVKTTADGSFISHSVSHRFKGGRFRRDLQ-------------------   [67]
Homo ADAMTS7 (11131377)                       ATEGRAALDIVHPVRVDAGGSFLSYELWP---RALRKRD---------------------   [71]
Fugu 7                                        LLLDLPQYDVVHPTRVDARGHFLSNFLSHHARRVQRREAPE-------------------   [42]
Ciona 7 (genewise.34.12.1)                    -----------------------------------------------------PVQVDTH   [7]
Homo ADAMTS17 (21265064)                      GDAAADVEVVLPWRVRPDDVHLPPLPAAPGPRRRRRPRTPPAAPRA-RPG----------   [77]
Homo ADAMTS19 (19525737)                      AAGSSREVRSVAPVPLEEPVEGRSESRLRPPPPPSEGEEDEELESQELPRGSSGAAALSP   [135]
Drosophila dCG4096 (22831757)                 HPDGAFMTHQLEYAHE---------LDHRRHRQRRSLNSEHDTQAA-DLHLL--------   [91]
Homo ADAMTS14 (29337086)                      HLSGKLSDYGVTVPCSTDFRGRFLSHVVSGPAAASAGSMVVDTPPT-LPRHS--------   [80]
Fugu 14                                       SRGRYLSHVVSAGSGSKGDDAAAAESDGATAPGSRRRVARSAPEMP--------------   [64]
Homo ADAMTS3 (2224673)                        PIKRYREYELVTPVSTNLEGRYLSHTLSASHKKRSARDVSSNP-----------------   [77]
Fugu 3                                        ------------------------------------------------------------   [0]
Ciona 3 (0100146117)                          -----------RFVEFEIIEPTVVTATGQYISHDVTNARRNTT-----------------   [32]
Homo ADAMTS2 (7656867)                        PLGHGAERILAVPVRTDAQGRLVSHVVSAATSRAGVRARRAAP-----------------   [91]
Mus 2 (28204840)                              PPGQGAERILAVPVRTDAQGRLVSHVVSLETAGAGVRARRAAL-----------------   [90]
Fugu 2                                        ------------------------------------------------------------   [0]
Homo ADAMTS13 (16306598)                      ------------------------------------------------------------   [0]
Fugu 13                                       ------------------------------------------------------------   [0]
Homo ADAMTS1 (6525075)                        LLAVSDALGRPSEEDEELVVPELER-----------APG---------------------   [70]
Mus 1 (1813340)                               ------------------------------------------------------------   [0]
Homo ADAMTS4 (12643637)                       SLLPSARLASPLPREEEIVFPEKLNGSV--------LPG---------------------   [74]
Mus 4 (26350615)                              ------------------------------------------------------------   [0]
Fugu 4                                        ------------------------------------------------------------   [0]
Homo ADAMTS8 (21536392)                       LARGAPARPAAGGQASELVVPTRLP-----------------------------------   [48]
Mus 8 (6708110)                               ------------------------------------------------------------   [0]
Fugu 8                                        LLLCVTDAALCAPFDSEDVLPVRTRKGGGGRLWK--------------------------   [41]
Homo ADAMTS5 (7768707)                        QGEEVQERAEPPGHPHPLAQRRRSKGLVQN-----IDQL---------------------   [80]
Mus 5 (5923784)                               -MRLEWASLLLLLLLLSASCLSLAADSPAAAPAQDKTRQPQAAAAAAEPDQPQGEETRER   [59]
Fugu 5                                        FLPPANGSLLAPARRTDGVVRTIDRIYHG-------------------------------   [30]
Ciona 15 (0100137065)                         ------------------------------------------------------------   [0]
Homo ADAMTS15 (19171175)                      TLAFAGRTAGGSEPEREVVVPIRLDPDINGRRYYWRGPE---------------------   [46]
Fugu 15                                       RRASAGARGAAG------------------------------------------------   [17]
Homo ADAMTS9 (27463365)                       LLETLGEYEIVSPIRVNALGEPFPTNVHFKRTRRSINSATDPWPAF-ASSS---------   [90]
Fugu 9                                        ------------------------------------------------------------   [0]
Homo ADAMTS20 (28460690)                      IPERVNEFGEVFPQSHHFSRQKRSS-EALEPMP---------------------------   [60]
Mus 20 (29500513)                             ------------------------------------------------------------   [0]
Fugu 20                                       LVKQLSSYEIITPLRVNDFGESFPLQLHYRRRRRSLSDHLS-------------------   [42]
Ciona 9 (0100132719)                          VPIRVHHVTGH-PVHHGRHFRTRKKEERDSPKDRRRRDVEEDERETILRDRLEHGIAN--   [153]
Apis 9                                        ------------------------------------------------------------   [0]
Drosophila CG6107 (20151419)                  QKMSTHWRQNACLYACCIAFLLGMLIMFHLGLRSAHKGQEELPQSTHPLANSPPATPATL   [209]
Caenorhabditis gon-1 CEt13h10 (3879882)       GTISEFSSDVLFSRAKYSG---VPVHHSRWRQDAGIHVIDSHHIVRRDSYGR--------   [82]
introns                                       -6-----7------------8-9----------------10---------11--------   [2]
```

[250 260 270 280 290 300]
[. . . . . .]

mask	--	[0]
Homo ADAMTS6 (11131380)	---------------------DPQQAVSKLFFKLSAYGKHFHLNLTLNTDFVSKHFTVEY	[115]
Fugu 6	-------------------SPSLDPLESRLFYSLSAYGKHFHLNLTLNPHLVSRHFAVEY	[122]
Ciona 6 (0100146470)	---------------------ATADGNHEVFYDLGEAPGSPLSGAKLRLTRNYKLVSKNF	[119]
Homo ADAMTS10 (17432918)	---------------------TGATAESRLFYKVASPSTHFLLNLTRSSRLLAGHVSVEY	[106]
Mus 10 (27502095)	--	[0]
Fugu 10	--------------------------KSQHHQRRRRSAEDRLPDSVLSSPFISSSCWPSA	[61]
Homo ADAMTS16 (21265061)	----------------------AVSEVESLHLRLKGPRHDFHMDLRTSSSLVAPGFIVQT	[127]
Mus 16 (21961374)	-----------MESRGCAALWVLLLAQVSEQQTPACALGLAAAASGSPEDPQPPPFSGSS	[49]
Fugu 16	------------------------GGGETVHFRLSGLGQDFHLELQETSDGLIAPGFTIQ	[76]
Ciona 16 (0100138085)	--	[0]
Homo ADAMTS18 (21265067)	----------------------AQNARSSLHYRFSAFGQELHLELKP-SAILSSHFIVQV	[126]
Fugu 18	--	[0]
Homo ADAMTS12 (13569928)	---------------------LDGS-EDWVYYRISHEEKDLFFNLTVNQGFLSNSYIMEK	[121]
Mus 12 (27817773)	--MPCARGSWLAKLSIVAQLINFGAFCHGRQTQPWPVRFPDPRQEHFIKSLPEYHIVSPV	[58]
Fugu 12	----------------------PSGLEELVYYKVNYKGRSLTFNLTVNHHLVSSGYVLER	[105]
Homo ADAMTS7 (11131377)	---------------------VSVRRDAPAFYELQYRGRELRFNLTANQHLLAPGFVSET	[110]
Fugu 7	----------------------GSANMDRVFYQLWHSNHSLRFNLTLNTHLLAPGFLTER	[80]
Ciona 7 (genewise.34.12.1)	GRFITNNVKFSYPKFKSRSRKKRSISEDDHIYFKMMIENKEVLLNLSRNHNLVHSNFIVE	[67]
Homo ADAMTS17 (21265064)	--------------------------ERALLLHLPAFGRDLYLQLRRDLRFLSRGFEVEE	[111]
Homo ADAMTS19 (19525737)	GAPASWQPPPPPQPPSPPPAQHAEPDGDEVLLRIPAFSRDLYLLLRRDGRFLAPRFAVEQ	[195]
Drosophila dCG4096 (22831757)	---------------------------------LPLANETLHLELMAHSYFLAPNLVVER	[118]
Homo ADAMTS14 (29337086)	----SHLRVARSPLHPGGTLWPGRVGRHSLYFNVTVFGKELHLRLRPNRRLVVPGSSVEW	[136]
Fugu 14	----------------------KVAPSQHLFFNVTVFGKELHLRLRANRRLVAPGAFVEW	[102]
Homo ADAMTS3 (2224673)	---------------------------EQLFFNITAFGKDFHLRLKPNTQLVAPGAVVEW	[110]
Fugu 3	--	[0]
Ciona 3 (0100146117)	-------------------------SQEHLHFNLTVFGESLKLRVRSKSHLISPRAKSVL	[67]
Homo ADAMTS2 (7656867)	---------VRTPSFPGGN--E-EEPGSHLFYNVTVFGRDLHLRLRPNARLVAPGATMEW	[139]
Mus 2 (28204840)	---------DQTSGLPGGA--A-QDPGGRLFYNLTVFGRDLHLRLRPNARLVAPGATVEW	[138]
Fugu 2	--	[0]
Homo ADAMTS13 (16306598)	-------------------------------------MHQRHPRARCPPLCVAGILACGF	[23]
Fugu 13	--	[0]
Homo ADAMTS1 (6525075)	----------------------HGTTR----LRLHAFDQQLDLELRPDSSFLAPGFTLQN	[104]
Mus 1 (1813340)	---MGDVQRAARSRGSLSAHML	[19]
Homo ADAMTS4 (12643637)	----------------------SGAPAR-LLCRLQAFGETLLLELEQDSGVQVEGLTVQY	[111]
Mus 4 (26350615)	--	[0]
Fugu 4	--	[0]
Homo ADAMTS8 (21536392)	-----------------------GSAGE-LALHLSAFGKGFVLRLAPDDSFLAPEFKIER	[84]
Mus 8 (6708110)	--	[0]
Fugu 8	------------------------RSEEQPSFLLSAFGKDMTLNLVPDTSFLAPSFSIQR	[77]
Homo ADAMTS5 (7768707)	----------------------YSGGGK-VGYLVYAGGRRFLLDLERDGSVGIAGF-VPA	[116]
Mus 5 (5923784)	GHLQPLAGQRRSGGLVHNIDQLYSGGGK-VGYLVYAGGRRFLLDLERDDTVGAAGSIVTA	[118]
Fugu 5	--------------------------GGKVGYLLYLDGSRFLLDMERDESALSHPFSPQY	[64]
Ciona 15 (0100137065)	--	[0]
Homo ADAMTS15 (19171175)	----------------------DSGDQG-LIFQITAFQEDFYLHLTPDAQFLAPAFSTEH	[83]
Fugu 15	--------------------------DGGVVFKLRAFTRDLYLRLTPDSGFLAPPESRAA	[51]
Homo ADAMTS9 (27463365)	----------------------SSSTSSQAHYRLSAFGQQFLFNLTANAGFIAPLFTVTL	[128]
Fugu 9	-----------------------------LGTFRSPEGEYFVEPLHNYQGEHYEEEHTKP	[31]
Homo ADAMTS20 (28460690)	-------------------------FR--THYRFTAYGQLFQLNLTADASFLAAGYTEVH	[93]
Mus 20 (29500513)	---MRVAK	[5]
Fugu 20	--------------------------SLRLLYRIDAFGERFHLNLTAHSDFIAPSYTVVH	[76]
Ciona 9 (0100132719)	-------------------------------YSLDAFGERFLLHLMPFDGFLAPNYTLHY	[182]
Apis 9	--	[0]
Drosophila CG6107 (20151419)	HPRRLDNDTSTDHEPPDGLDDLDEEEHSAFVMPTKVYNYSLSEADLIYESKRNSDINSFL	[269]
Caenorhabditis gon-1 CEt13h10 (3879882)	---------------RGKRDVTSTDRRRRLQGVARDCGHACHLRLRSDDAVYIVHLHRWN	[127]
introns	--	[2]

```
[                                                    310       320       330       340       350       360]
[                                                      .         .         .         .         .         .]

mask                                           ------------------------------------------------------------   [0]
Homo ADAMTS6 (11131380)                        W-----------------------------------------------------------   [116]
Fugu 6                                         W-----------------------------------------------------------   [123]
Ciona 6 (0100146470)                           VVEER-------------------------------------------------------   [124]
Homo ADAMTS10 (17432918)                       W-----------------------------------------------------------   [107]
Mus 10 (27502095)                              ----------------------------------------MSSCPVWRAMRLPSQLEWTT   [20]
Fugu 10                                        GCDN--------------------------------------------------------   [65]
Homo ADAMTS16 (21265061)                       L-----------------------------------------------------------   [128]
Mus 16 (21961374)                              WLETGEYDLVSAYEVDHRGDYVSHDIMHYQRRRRRRAVTQPGGDALHLRLKGPRHDLHLD   [109]
Fugu 16                                        VLGTN-------------------------------------------------------   [81]
Ciona 16 (0100138085)                          ------------------------------------------------------------   [0]
Homo ADAMTS18 (21265067)                       L-----------------------------------------------------------   [127]
Fugu 18                                        ------------------------------------------------------------   [0]
Homo ADAMTS12 (13569928)                       R-----------------------------------------------------------   [122]
Mus 12 (27817773)                              QVDAGGHVLSYGLHHPVTSSRKKRAAGGSGDQLYYRISHEEKDLFFNLTVNWEFLSNGYV   [118]
Fugu 12                                        RNGG--------------------------------------------------------   [109]
Homo ADAMTS7 (11131377)                         R-----------------------------------------------------------   [111]
Fugu 7                                         RYGG--------------------------------------------------------   [84]
Ciona 7 (genewise.34.12.1)                     RLRDDASNAKRNPGVKNK------------------------------------------   [85]
Homo ADAMTS17 (21265064)                       ------------------------------------------------------------   [111]
Homo ADAMTS19 (19525737)                       RPNPGPGPT---------------------------------------------------   [204]
Drosophila dCG4096 (22831757)                  HRRDLRTRSPLTTRHL--------------------------------------------   [134]
Homo ADAMTS14 (29337086)                       QE---------D------------------------------------------------   [139]
Fugu 14                                        QED---------------------------------------------------------   [105]
Homo ADAMTS3 (2224673)                         HETSLVPGNITDPINNHQ------------------------------------------   [128]
Fugu 3                                         ------------------------------------------------------------   [0]
Ciona 3 (0100146117)                           FTSG--------------------------------------------------------   [71]
Homo ADAMTS2 (7656867)                         QG---------E------------------------------------------------   [142]
Mus 2 (28204840)                               QG---------E------------------------------------------------   [141]
Fugu 2                                         ------------------------------------------------------------   [0]
Homo ADAMTS13 (16306598)                       LLGCW-------------------------------------------------------   [28]
Fugu 13                                        ------------------------------------------------------------   [0]
Homo ADAMTS1 (6525075)                         VGRK--------------------------------------------------------   [108]
Mus 1 (1813340)                                LLLLASITMLLCARGAHGRPTEEDEELVLPSLERAPGHDSTTTRLRLDAFGQQLHLKLQP   [79]
Homo ADAMTS4 (12643637)                        LG----------------------------------------------------------   [113]
Mus 4 (26350615)                               ------------MASIHPSCSPGTMSQMGLHPRRGLTGHWLQRFQPCLPLHTVQWRRLLL   [48]
Fugu 4                                         ------------------------------------------------------------   [0]
Homo ADAMTS8 (21536392)                         LG----------------------------------------------------------   [86]
Mus 8 (6708110)                                ----MLRDPTTTGWPPLLLLLLQLPPPPLVCGAPAGPGTGAQASELVVPTRLPGSASELA   [56]
Fugu 8                                         LRASGAAVP---------------------------------------------------   [86]
Homo ADAMTS5 (7768707)                          GGG---------------------------------------------------------   [119]
Mus 5 (5923784)                                GGG---------------------------------------------------------   [121]
Fugu 5                                         VFAMMG------------------------------------------------------   [70]
Ciona 15 (0100137065)                          ----MSMLKIVFLFVFLSTV----------------------------------------   [16]
Homo ADAMTS15 (19171175)                        LG-V--------------------------------------------------------   [86]
Fugu 15                                        AS----------------------------------------------------------   [53]
Homo ADAMTS9 (27463365)                         LGTPGV------------------------------------------------------   [134]
Fugu 9                                         HVVYR-------------------------------------------------------   [36]
Homo ADAMTS20 (28460690)                       LGTP--------------------------------------------------------   [97]
Mus 20 (29500513)                              WLTGLLCPISLLLTGSWEVRFHPRQEALVKTLASYEVVTPTRVNEFGDVFPQNRHFSRKK   [65]
Fugu 20                                        LG----------------------------------------------------------   [78]
Ciona 9 (0100132719)                           MGDAATD-----------------------------------------------------   [189]
Apis 9                                         ------------------------------------------------------------   [0]
Drosophila CG6107 (20151419)                   KESASAFAMTGTYRNMSNEIWDPHPQYNLNVFGRQLHLVLRQDASF-VHNHSMTHIRILK   [328]
Caenorhabditis gon-1 CEt13h10 (3879882)        QIPDSHNKSVPHFSNSNFAPMVLYLDSE--------------------------------   [155]
introns                                        --12----13---------------------------------------------------   [2]
```

```
[                                                       370       380       390       400       410       420]
[                                                         .         .         .         .         .         .]

mask                                          ------------------111111111-----------1111----------------11   [15]
Homo ADAMTS6 (11131380)                       ----GKDGPQWKH--DFLDNCHYTGYLQDQRSTT---KVALSNCVG------LHGVIATE   [161]
Fugu 6                                        ----GKDGLQWRH--NTADNCHYVGVLQNQHGTTR---VALSNCKG------LHGVITTE   [168]
Ciona 6 (0100146470)                          -SRAGK--IRSRH--TKLEECHYVGVIHNHNSFS---KVALSLCNG------MQGLITKD   [170]
Homo ADAMTS10 (17432918)                       ----TREGLAWQR--AARPHCLYAGHLQGQASSS---HVAISTCGG------LHGLIVAD   [152]
Mus 10 (27502095)                             TGQCWPSLHLPSGGSVGVQGLQLSPAYSTSTHFLL--NLTRSPRLLAG---HVSVEYWTR   [75]
Fugu 10                                       ------SLPLVFVLVALLPAVHITQQLPAQPHVAG--RPAVETVQGGILEERAQGVIVAG   [117]
Homo ADAMTS16 (21265061)                       ----GKTGTKSVQTLPPEDFCFYQGSLRSHRNSS----VALSTCQG------LSGMIRTE   [174]
Mus 16 (21961374)                             LKAASNLMAPGFMVQTLGKGGTKSVQMFPPEENCF--YQGSLRSQGNS---SVALSTCQG   [164]
Fugu 16                                       -------GTKSLRAYQKHDLCFYQGSLRSRVNSS----VALSTCTG------MVSLIRTP   [124]
Ciona 16 (0100138085)                         --------------------------MTSHGKRR----VAMSTCEG------LTGLLRTS   [24]
Homo ADAMTS18 (21265067)                       ----GKDGASETQ-KPEVQQCFYQGFIRNDSSSS----VAVSTCAG------LSGLIRTR   [172]
Fugu 18                                       ------------------------------------------------------------   [0]
Homo ADAMTS12 (13569928)                       ----YGNLSHVKMMASSAPLCHLSGTVLQQGTRVG--TAALSACHG------LTGFFQLP   [170]
Mus 12 (27817773)                             VEKRYGNLSHVKMVASSGQPCHLRGTVLQQGTTVGIGTAALSACQG------LTGFFHLP   [172]
Fugu 12                                       -------SNRTEHPLSEGNSCHLLGTVEGSDGRG---TAAVSTCRG------LRGFFSLP   [153]
Homo ADAMTS7 (11131377)                        ----RRGGLGRAHIRAHTPACHLLGEVQDPELEGG--LAAISACDG------LKGVFQLS   [159]
Fugu 7                                        -------LEGAKMQTAASSQCHFLGEVWDEDSVKG--SAAISTCDG------LTGLFRLS   [129]
Ciona 7 (genewise.34.12.1)                    ---FESAPLRSSIKLGNGRQCYFRGHVTGHPNS----QVAISTCNG------LTGFVRLN   [132]
Homo ADAMTS17 (21265064)                       -------AGAARRRGRPAELCFYSGRVLGHPGSL----VSLSACGAAG---GLVGLIQLG   [157]
Homo ADAMTS19 (19525737)                       -----GAASAPQPPAPPDAGCFYTGAVLRHPGSL----ASFSTCGG-----GLMGFIQLN   [250]
Drosophila dCG4096 (22831757)                 --NCHFHGKVRGQPATNVAISTCAGLVSCPNFC-V-LPELITLCQ--------VGHIRTA   [182]
Homo ADAMTS14 (29337086)                       --------FRELFRQPLRQECVYTGGVTGMPGAA----VAISNCDG------LAGLIRTD   [181]
Fugu 14                                       --------FVEKAKERIYGDCVFTGDVSDMPEAS----VAISNCDG------LSGLIRTD   [147]
Homo ADAMTS3 (2224673)                         ---PGSATYRIRKTEPLQTNCAYVGDIVDIPGTS----VAISNCDG------LAGMIKSD   [175]
Fugu 3                                        -------VNDTVKRR---------------------------------------------   [8]
Ciona 3 (0100146117)                          ---------GGRVERKLDPGCVFTGEIPQLPHSN----VAISNCDG------LAGLIRTP   [112]
Homo ADAMTS2 (7656867)                         --------KGTTRVEPLLGSCLYVGDVAGLAEASS---VALSNCDG------LAGLIRME   [185]
Mus 2 (28204840)                              --------TGDTRVEPLLGSCLYVGDVADLPKASS---VALSNCDG------LAGLIRME   [184]
Fugu 2                                        ----------ADLGGLMDLESLYRGVEQSINHTR--------------------------   [24]
Homo ADAMTS13 (16306598)                       ------------GPSHFQQSCLQALEPQ----------AVSS------------------   [48]
Fugu 13                                       ------------------------------------------------------------   [0]
Homo ADAMTS1 (6525075)                         ------SGSETPLPETDLAHCFYSGTVNGDPSSA----AALSLCEG------VRGAFYLL   [152]
Mus 1 (1813340)                               DSGFLAPGFTLQTVGRSPGSEAQHLDPTGDLAHCF-YSGTVNGDPGS----AAALSLCEG   [134]
Homo ADAMTS4 (12643637)                        --------QAPELLGGAEPGTYLTGTINGDPESV----ASLHWDGGA-----LLGVLQYR   [156]
Mus 4 (26350615)                              LAFLLSLAWPASPLPREEEIVFPEKLNGSSILPGS--GVPARLLYRLP---AFGEMLLLE   [103]
Fugu 4                                        ------------------------------------------------------------   [0]
Homo ADAMTS8 (21536392)                        ------GSGRATGGERGLRGCFFSGTVNGEPESL----AAVSLCRG------LSGSFLLD   [130]
Mus 8 (6708110)                               FHLSAFGQGFVLRLAPDASFLAPEFKIERLGGSSAAAGGEPGLRGC-----FFSGTVNGE   [111]
Fugu 8                                        ----RSSVNRTEESAEQLRGCFYSGTVDEEQDSL----VSVSLCSG------IHGSFITG   [132]
Homo ADAMTS5 (7768707)                         ----------TSAPWRHRSHCFYRGTVDASPRSL----AVFDLCGGLD---GFFAVKHAR   [162]
Mus 5 (5923784)                               ----------LSASSGHRGHCFYRGTVDGSPRSL----AVFDLCGGLD---GFFAVKHAR   [164]
Fugu 5                                        -----------ESPAPLQRECVYRGTVDSNPESL----AVFNLCGGG-----LQGFFALN   [110]
Ciona 15 (0100137065)                         ------G-----AGDVGKDVDFVIP-VRMEEDLSRV--AGSKHFKSRHTVTHETTSKHTV   [62]
Homo ADAMTS15 (19171175)                       ------PLQGLTGGSSDLRRCFYSGDVNAEPDSF----AAVSLCGG------LRGAFGYR   [130]
Fugu 15                                       -------------AASDLRDCFYSGDVNADPDSF----AALSLCGG------LSGGFSYD   [90]
Homo ADAMTS9 (27463365)                        ----NQTKFYSEE-EAELKHCFYKGYVNTNSEHT----AVISLCSG------MLGTFRSH   [179]
Fugu 9                                        ------KKAPKQQTSGEGSACDTSGHQRRHKRHKP--RRRLMPAAS------LLPHAPPL   [82]
Homo ADAMTS20 (28460690)                       ----ERGAWESDAGPSDLRHCFYRGQVNSQEDYK----AVVSLCGG------LTGTFKGQ   [143]
Mus 20 (29500513)                             RSSGVPEPPPFRTHYRISAYGQLFQLNLSADAAFL--AAGYTEVHLGT---PVPGPGGRS   [120]
Fugu 20                                       ------AEQGNGTERSDMRHCFFRGHVNGRSDFP----AVFSLCTG------LVGTFTTQ   [122]
Ciona 9 (0100132719)                          ------GFAGASLGVP--RHCFYSGYVNERPEHT----AVLSVCSG------LFGAFRTD   [231]
Apis 9                                        ------------MAVKRPPMCRNTLTDQRNATP--------DIETG--------TRKQAA   [32]
Drosophila CG6107 (20151419)                  EGEEHPGPETEAEAEQRHLGCFYSGYVEDDPHSM----VSVSLCGG------MTGYIKTS   [378]
Caenorhabditis gon-1 CEt13h10 (3879882)       -------EEVRGGMSRTDPDCIYRAHVKGVHQHSI--VNLCDSEDG------LYGMLALP   [200]
introns                                       ------14-15-------16----17--------------------------18------   [2]
```

```
[                                                      430       440       450       460       470       480]
[                                                       .         .         .         .         .         .]

mask                                        11-1111---------------------------11111111------------------   [30]
Homo ADAMTS6 (11131380)                     DE-EYFIEPLKN--------TTEDSKHFSYENG-HPHVIYKKSALQQRHLYDHSHCGVSD   [211]
Fugu 6                                      EE-QYLIEPLKN--------TSSTTSSEWNLEEAQQHVIYKMSAIPSPQEPSQEFSCG--   [217]
Ciona 6 (0100146470)                        DD-TFFIEPKWNHTSNSTSSEFSTTASPQEVEEGHPHVIVKRSAIRRSHHHKKQFEDL--   [227]
Homo ADAMTS10 (17432918)                    EE-EYLIEPLHG---------GPKGSR-SPEE-SGPHVVYKRSSLRHPHLDTACGVRD--   [198]
Mus 10 (27502095)                           EG-LAWQRAARAH-------CLYAGHLQGQAGSSHVAVSTCGGLHGLIVADDEEYLIEPL   [127]
Fugu 10                                     GE-EYLIEPLVSP-------DNQTRMEKGERAEGRPHVVYKRSSLRHQYKGQSCGVID--   [167]
Homo ADAMTS16 (21265061)                     EA-DYFLRPLPSH--------LSWKLGRAAQGSSPSHVLYKRSTEPHAPGASEVLVTSR-   [224]
Mus 16 (21961374)                           LL-GMIRTKDTDY-------FLKPLPPHLTSKLNRSAQGDSPSHVLYKRSTERQAPRENE   [216]
Fugu 16                                     DA-DYFLRPVSRD--------LAQRENFTAPSSHQPHILYRRTEPRGPDPRARDGEDQEY   [175]
Ciona 16 (0100138085)                       ED-DFLVEPLPHH------------INHTFPDRQHPHIIYKRRHLYKHRLHYCGKKKRYT   [71]
Homo ADAMTS18 (21265067)                    KN-EFLISPLPQL--------LAQEHNYSSPAGHHPHVLYKRTAEEKIQRYRGYPGSGRN   [223]
Fugu 18                                     ------------------------------------------------------------   [0]
Homo ADAMTS12 (13569928)                    HG-DFFIEPVKK---------HPLVEG-----GYHPHIVYRRQKVPETKEPTCGLKD---   [212]
Mus 12 (27817773)                           HG-DFFIEPVKK---------HPLTEE-----GSYPHVVYRRQSIRAPETKEPICGLKD-   [216]
Fugu 12                                     EG-QYFIEPIQSS-------------PHDATDAPEPHVVYPRVTETHRKKRNVDPGATPG   [199]
Homo ADAMTS7 (11131377)                     NE-DYFIEPLDS---------APARPG-----HAQPHVVYKRQAPERLAQRGDSSAPSTC   [204]
Fugu 7                                      KE-EFFIQPLQKS--------TLETSAPQAHAIYKRHVTSPSWNPVLQPLPGKQTLNGTC   [180]
Ciona 7 (genewise.34.12.1)                  NTAEYLIEPTAI--------------HSLSKNSRMPHRLYKRGAERRQAADFLRARRTDE   [178]
Homo ADAMTS17 (21265064)                     QE-QVLIQPLNN---------SQGPFS-----G-REHLIRRKWSLTPSPSAEAQRPEQLC   [201]
Homo ADAMTS19 (19525737)                     ED-FIFIEPLND---------------TMAITG-HPHRVYRQKRSMEEKVTEKSALHSHY   [293]
Drosophila dCG4096 (22831757)               GN-EYFIEPSKEH-------EPHP------VNG-HPHVVFQRSSVKPKHSLRKRNKRKRG   [227]
Homo ADAMTS14 (29337086)                    ST-DFFIEPLER---------GQQEKEA-S--G-RTHVVYRREAVQQEWAEPDGDLHNEA   [227]
Fugu 14                                     NG-EFFIEPLEKG-------QQDVEVKG------RVHVVYRRSAIKKETGQQRQDLRN--   [191]
Homo ADAMTS3 (2224673)                      NE-EYFIEPLER---------GKQMEEE-K--G-RIHVVYKRSAVEQAPI----------   [211]
Fugu 3                                      ------------------------------------------------------------   [8]
Ciona 3 (0100146117)                        DD-EIFIEPIED---------RHVT-------GPQKHVIYRRSAT-KNSANHGQRKLRHS   [154]
Homo ADAMTS2 (7656867)                      EE-EFFIEPLEK---------GLAAQEAEQ--G-RVHVVYRRPPT--SPPLGGPQALDTG   [230]
Mus 2 (28204840)                            EE-EFFIEPLEK---------GQTDQEAEQ--G-RVHVVYRRPPTPKPPPVSEPQALDTG   [231]
Fugu 2                                      ------------------------------------------------------------   [24]
Homo ADAMTS13 (16306598)                     -Y-LSPGAPLK---------------------GRPPSPGFQRQRQ---------------   [70]
Fugu 13                                     ------------------------------------------------------------   [0]
Homo ADAMTS1 (6525075)                      GE-AYFIQPLPAA-------SERLATAAPGEKPPAPLQFHLLRRNRQGDVGG--TCGVVD   [202]
Mus 1 (1813340)                             VR-GAFYLQGEEF-------FIQPAPGVATERLAPAVPEEESSARPQFHILRRRRRGSGG   [186]
Homo ADAMTS4 (12643637)                      GA-ELHLQPLEGG-------TPNSAGGP------GAHILRRKSP----------------   [186]
Mus 4 (26350615)                            LE-QDPGVQVEGL-------TVQYLGQAPEMLGGAEPGTYLTGTINGDPESVASLHWDGG   [155]
Fugu 4                                      ------------------------------------------------------------   [0]
Homo ADAMTS8 (21536392)                      GE-EFTIQPQGA---------GGSLA--------QPHRLQRWGPAGARPLPR--------   [164]
Mus 8 (6708110)                             RE-SLAAMSCVAG-------WSGSFLLAGEEFTIQPQGAGDSLDQPHRLQRWGPGQRRED   [163]
Fugu 8                                      GT-EYLIEP---------------------------------------------------   [140]
Homo ADAMTS5 (7768707)                      YT-LKPLLRGPWA-------EEEKGRVYGDGSARILHVYTREGFSFEALPPRASCE---T   [211]
Mus 5 (5923784)                             YT-LKPLLRGSWA-------EYER--IYGDGSSRILHVYNREGFSFEALPPRASCE---T   [211]
Fugu 5                                      HS-RYSITPIVRA-------KGHEHDVRTLQDKDAESALHAFTRESFSFEATREGRESCG   [162]
Ciona 15 (0100137065)                       HE-DEFTRPVYKI-------PAFGKDLLVR-------LEKDNSLVAPSFTTSYSYSNETV   [107]
Homo ADAMTS15 (19171175)                     GA-EYVISPLPN---------ASA--PAAQRNSQGAHLLQRRGVPG--------------   [164]
Fugu 15                                     GM-EYFISPGGSA-------RAAPGDPAERTHVISRRGRVDPGGSATRRCGVAPGGN---   [139]
Homo ADAMTS9 (27463365)                      DG-DYFIEPLQS---------MDEQEDEEEQN--KPHIIYRRSAPQREPSTGR-------   [220]
Fugu 9                                      VG-ARIFSDLESL-------GAGVVSESLRRTASSPGGRPSNDSSSDSGPHR--------   [126]
Homo ADAMTS20 (28460690)                     NG-EYFLEPIMK---------ADGNEYED--GHNKPHLIYRQDLNNSFLQT---------   [182]
Mus 20 (29500513)                           TE-SPDLRHCFYR-------GQVNAREDHTAVFSLCGGLMGTFKANDGEYFLEPVLRADG   [172]
Fugu 20                                     HG-EYFLEPLLNA-------AGEEYDEQHNKPHLVYRHERHKNTSKSANATVPCAASEDS   [174]
Ciona 9 (0100132719)                        TS-DYLIQPEHKS--------SGSEHDEAEEEGAKRHRIFRRSTRAAGSTGKSLCGYTGE   [282]
Apis 9                                      KL-KYDNTPTPI------------------VENSSKGKIYFDGRRRKRR-----------   [62]
Drosophila CG6107 (20151419)                FG-ALLIQPVNR---------TSSDE--------VLHRVFRKSQRNARHAVSKFEL----   [416]
Caenorhabditis gon-1 CEt13h10 (3879882)     SG-IHTVEPIIS---------GNGTEHD----GASRHRQHLVRKFDPMHFKSF-------   [239]
introns                                     -------------------------19-----20-------------21-22------23   [2]
```

AC Nicholson, SB Malik, JM Logsdon Jr, E van Meir, 2004. Functional evolution of ADAMTS genes: evidence]
[from analyses of phylogeny and gene organization. Contact: john-logsdon@uiowa.edu]
[Intron phases marked 0, 1, 2]

```
[                                                                      490       500       510       520       530       540]
[                                                                        .         .         .         .         .         .]

mask                                        ------------------------------------------------------------   [30]
Homo ADAMTS6 (11131380)                     -------------------------------------FTR-SGKPWWLNDTSTVSYSL-P   [232]
Fugu 6                                      ----------------------------ISDLIKTSSPCQ-LSTPSPLHPSAEPKNGS-E   [247]
Ciona 6 (0100146470)                        -----------------------------------DFESL-SNHRTSAGRRRGLEEDSTE   [251]
Homo ADAMTS10 (17432918)                    -------------------------------------EKP-WKGRPWWLRTLKPPPAR-P   [219]
Mus 10 (27502095)                           QGGPKGHRGPEESGPHVVYKRSSLRHPHLDTACGVRDEKP-WKGRPWWLRTLKPPPAR-P   [185]
Fugu 10                                     -------------------------------------EKP-MKSASWWQRTLKTPPHH-V   [188]
Homo ADAMTS16 (21265061)                    ------TWELAHQPLHSSDLRLGLPQKQHFCGRRKKYMPQ-PPKEDLFILPDEYKS----   [273]
Mus 16 (21961374)                           VLMITRKRDLARPHLHHDNFHLGPSQKQHFCGRRKKYMPQ-PPNDDLYILPDEYKP----   [271]
Fugu 16                                     RQQGRGDILNDRTEHRGDGHHHGNHHSDYRHGGRQRQHFC-GRRKKYMPKPPEDDTFILP   [234]
Ciona 16 (0100138085)                       YKDQREVLSQIVLRNVTRVHCYAVKGYINVSLGNERYPVT-IPDTENHQII-KLLSNGEI   [129]
Homo ADAMTS18 (21265067)                    YPGYSPSHIPHASQSRETEYHHRRLQKQHFCGRRKKYAPK-PPTEDTYLRFDEYGS----   [278]
Fugu 18                                     -------------------------------------APK-PPTDDRFIMPDEFATPA-T   [21]
Homo ADAMTS12 (13569928)                    -------------------------------------SVN-ISQKQELWREKWERHNL-P   [233]
Mus 12 (27817773)                           -------------------------------------SLD-NSVKQELQREKWERKTL-R   [237]
Fugu 12                                     PCGVQDAP---------------------------IDSVH-VEREREEWEREQQRDGG-Q   [230]
Homo ADAMTS7 (11131377)                      GVQV---------------------------------YPE-LESRR----ERWEQRQQ-W   [225]
Fugu 7                                      G---------------------------------NKNSHQ-SLADFERHRERWE------   [200]
Ciona 7 (genewise.34.12.1)                  ANARQK------------------------------------------WED---------   [187]
Homo ADAMTS17 (21265064)                     KVLT-----E---------------------------KKK-PTW--------GRPSRD-W   [219]
Homo ADAMTS19 (19525737)                     CGIISD-------------------------------KGR-PRSRKIA------------   [309]
Drosophila dCG4096 (22831757)               GKSGSGAEVSNCGTREPRRRMETRLEWQARGKVKVQGGRQ-IRRHHHHHHHHHHKHKY-R   [285]
Homo ADAMTS14 (29337086)                     -------------------------------------FGL-GDLPNLLGLVGDQLG----   [245]
Fugu 14                                     -----------------------------------EVADF-GIANLPVALDIVEHKL--S   [213]
Homo ADAMTS3 (2224673)                       DMSKDFHYRE---------------------------SDL-EGLDDLGTVYGNIHQQL-N   [242]
Fugu 3                                      ------------------------------------------------------------   [8]
Ciona 3 (0100146117)                        TTR--------------------------------RTFRD-DTRDSMQGSLEVILEQTK-   [180]
Homo ADAMTS2 (7656867)                       -------------------------------------ASL-DSLDSLSRALGVLEEHA-N   [251]
Mus 2 (28204840)                            -------------------------------------VSQ-GNLDSLSRALGVLEERI-N   [252]
Fugu 2                                      ------------------------------------------------------------   [24]
Homo ADAMTS13 (16306598)                    ------------------------------------------------------------   [70]
Fugu 13                                     ------------------------------------------------------------   [0]
Homo ADAMTS1 (6525075)                      DEPRP-TGKAETEDEDEGTE--------GEDEGPQWS------PQDPALQGVGQPTG--T   [245]
Mus 1 (1813340)                             AKCGVMDDETLPTSDSRPE---------SQNTRNQW-------PVRDPTPQDAGKPSG-P   [229]
Homo ADAMTS4 (12643637)                      ----------------------------ASGQGPMCN------VKAP----LGSPS---P   [205]
Mus 4 (26350615)                             ALLGVLQYRGAELHLQPLEGGALNSAGGPGAHILRRKSPA-SSQGPMCTVKAPSGSPS-P   [213]
Fugu 4                                      ------------------------------------------------------------   [0]
Homo ADAMTS8 (21536392)                      ---GP-EWEVETGEGQRQER-------GDHQEDSEEE-----SQEEEAEGASEPPPPL-G   [207]
Mus 8 (6708110)                             PGLAAAEVFPLPQGLEWEVEMGNGQGQERSDNEEDRKQDK-EGLLKETEDSRKVPPPF-G   [221]
Fugu 8                                      ------------------------------------------------------------   [140]
Homo ADAMTS5 (7768707)                      PAS-TPEAHEHAPAHSNP-------SGRAALASQL------LDQSALSPAGGSGP-QT-W   [255]
Mus 5 (5923784)                             PAS-PSGPQESPSVHSRS-------RRRSALAPQL------LDHSAFSPSGNAGP-QT-W   [255]
Fugu 5                                      TRDGRRGRRLRRHRKG--------------ARDGEGLAGE-SGRHGSRGRRWRSRPAA-A   [206]
Ciona 15 (0100137065)                       NDMNTFKQPLLRHCFYKGTVLGEPESQVALSICDSLTGSI-YTDSAHYMIKPTKNAHLPN   [166]
Homo ADAMTS15 (19171175)                    ---GP-SGDPTSRCGVASGW--------NPAILRALD------PYKPRRAGFGESRSR-R   [205]
Fugu 15                                     ------------------------------------------FSASLERYRHLSETVL-G   [156]
Homo ADAMTS9 (27463365)                      ------------------------------HACDTSEHKN-RHSKDKKKTRARKWGER-I   [248]
Fugu 9                                      ------------------------------------------------------------   [126]
Homo ADAMTS20 (28460690)                    -----------------------------LKYCSVSESQI-KETSLPFHTYSNMNEDL-N   [211]
Mus 20 (29500513)                           SAHDDDHNKPHLIYRQELKRNSFARSHKPCEVSENQMEKT-ALPSQSSRNTTGDVDIE-E   [230]
Fugu 20                                     KMSDVFESGKNMG--------------------DELESLR-ATIEERHISAHDADGAQ-P   [212]
Ciona 9 (0100132719)                        KMSCQLYTNQSYRP-------------------SVTLNNL-TAQSSLKPTIEKQQEES--   [320]
Apis 9                                      --------------------------------SLTEKKELERSEEDEMYKQFVGQAEGHR   [90]
Drosophila CG6107 (20151419)                -GLDDFMSKLEQVQEEEQKSKSRKLN----------------------------------   [441]
Caenorhabditis gon-1 CEt13h10 (3879882)     ----------DHLNSTSVNETETTVATWQDQWEDVIERKA--------------------   [269]
introns                                     -------------------------------24---25,26---27-28----------29   [2]
```

AC Nicholson, SB Malik, JM Logsdon Jr, E van Meir, 2004. Functional evolution of ADAMTS genes: evidence]
[from analyses of phylogeny and gene organization. Contact: john-logsdon@uiowa.edu]
[Intron phases marked 0, 1, 2]

```
[                                                     550       560       570       580       590       600]
[                                                      .         .         .         .         .         .]
[Modal, ≥80%, full maj.                                                          R  R                E  ]
mask                                            -------------------------------------1111--------11111111111   [45]
Homo ADAMTS6 (11131380)                         INNTHI--HH---------------------------RQKR--------SVSIERFVETL   [255]
Fugu 6                                          QSGDG-------------------------------HRRRR--------SVSSERFVETL   [268]
Ciona 6 (0100146470)                            EVTNEEDIAKS--------------------------RGKR--------SISTERNVETM   [277]
Homo ADAMTS10 (17432918)                        LGNETE--RGQP-------------------------GLKR--------SVSRERYVETL   [244]
Mus 10 (27502095)                               LGNESE--RGQL-------------------------GLKR--------SVSRERYVETL   [210]
Fugu 10                                         GRGQ--------------------------------WPLKR--------SVNRERYVETL   [208]
Homo ADAMTS16 (21265061)                        -CL----------------------------------RHKRSLLRS---HRNEELNVETL   [295]
Mus 16 (21961374)                               -SS----------------------------------RHKRSLLKS---HRNEELNVETL   [293]
Fugu 16                                         DEYKFIP------------------------------RNKRAVLMKT--QHSRKLNVEML   [262]
Ciona 16 (0100138085)                           ELESDE-----------------------------------------Q--TPVT?KNVETL   [147]
Homo ADAMTS18 (21265067)                        -SG----------------------------------RPRRSAG-----KSQKGLNVETL   [298]
Fugu 18                                         DESG---------------------------------RAKRSPINS---NRVGGLNVETL   [45]
Homo ADAMTS12 (13569928)                        SRS----------------------------------LSRR--------SISKERWVETL   [251]
Mus 12 (27817773)                               SRS----------------------------------LSRR--------SISKERWVETL   [255]
Fugu 12                                         ARP----------------------------------RSQR--------SVSKERWVESM   [248]
Homo ADAMTS7 (11131377)                         RRP----------------------------------RLRRLHQR----SVSKEKWVETL   [247]
Fugu 7                                          RRR---------------------------------LRKRRRIRQR---SVSKEKWVETL   [224]
Ciona 7 (genewise.34.12.1)                      ------------------------------------RRIKRNPFQRSRRSISLEHWIELM   [211]
Homo ADAMTS17 (21265064)                        -------------------------------------RERRNAI-----RLTSEHTVETL   [237]
Homo ADAMTS19 (19525737)                        ----------------------------------ESGRGKRYSY-----KLPQEYNIETV   [330]
Drosophila dCG4096 (22831757)                   HHQQKISRVPHT-KFKYETQF-QTE-----PDHAEIPRRRR--------SISSPRHVETL   [330]
Homo ADAMTS14 (29337086)                        DTE----------------------------------RKRRH-------AKPGSYSIEVL   [264]
Fugu 14                                         ESE----------------------------------RKRR-----H--AKTEDYNIEVL   [232]
Homo ADAMTS3 (2224673)                          ETM----------------------------------RRRRH-------AGENDYNIEVL   [261]
Fugu 3                                          -------------------------------------RRRRD-------AGEDDYNIEIL   [24]
Ciona 3 (0100146117)                            -----------------------------------RERSKRH-------AKRNDYNMEIL   [198]
Homo ADAMTS2 (7656867)                          SS---------------------------------RRRARRH-------AADDDYNIEVL   [271]
Mus 2 (28204840)                                SS---------------------------------RRRVRRH-------ATDDDYNIEVL   [272]
Fugu 2                                          -----------------------------------AGRMRRQ-------SLERSYNIEVL   [42]
Homo ADAMTS13 (16306598)                        -------------------------------------RQRR--------AAGGILHLELL   [85]
Fugu 13                                         ------------------------------------GRARRS----V--LMPDVTHLELL   [18]
Homo ADAMTS1 (6525075)                          GSI----------------------------------RKKR--------FVSSHRYVETM   [263]
Mus 1 (1813340)                                 GSI----------------------------------RKKR--------FVSSPRYVETM   [247]
Homo ADAMTS4 (12643637)                         RPR----------------------------------RAKR--------FASLSRFVETL   [223]
Mus 4 (26350615)                                ISR----------------------------------RTKR--------FASLSRFVETL   [231]
Fugu 4                                          -----------------------------------HHRTRR--------FVSTPRYLEIM   [17]
Homo ADAMTS8 (21536392)                         ATS----------------------------------RTKR--------FVSEARFVETL   [225]
Mus 8 (6708110)                                 SKT----------------------------------RSKR--------FVSEARFVETL   [239]
Fugu 8                                          ------------------------------------------------------KVVETL   [146]
Homo ADAMTS5 (7768707)                          WR-----------------------------------RRRR--------SISRARQVELL   [272]
Mus 5 (5923784)                                 WR-----------------------------------RRRR--------SISRARQVELL   [272]
Fugu 5                                          PVR----------------------------------RRER--------SVSRARYVELL   [224]
Ciona 15 (0100137065)                           KAYHSVSKITNRVRRSISTSE-NFKEPVVAEGVRAKSRSKRA-------FDSQQHYIETL   [218]
Homo ADAMTS15 (19171175)                        RSG----------------------------------RAKR--------FVSIPRYVETL   [223]
Fugu 15                                         GAG----------------------------------RSRR--------FASIPRFVEVL   [174]
Homo ADAMTS9 (27463365)                         NLAGDVAALNSG-LATEAFSA-YGNKTDNTREKRTHRRTKR--------FLSYPRFVEVL   [298]
Fugu 9                                          -------------------------------------RSKR--------FLSYPRFVEVM   [141]
Homo ADAMTS20 (28460690)                        VMKERVLGHTSK-NV-------------PLKDERRHSRKKR--------LISYPRYIEIM   [249]
Mus 20 (29500513)                               EAVFRLEGERSQ-L---------------------HSRNKR--------FLSYPRYVEVM   [260]
Fugu 20                                         RSP---------------------------------HRRKR--------FLSYPRYVELM   [231]
Ciona 9 (0100132719)                            -------------NT-------------P-----R-SRKKR--------YLSYPRNVEVM   [340]
Apis 9                                          NFLRNDARYYSVERRRNDLSEEYGQDSEMESDPSVTWRPRR--------ALSSEYFIEIM   [142]
Drosophila CG6107 (20151419)                    -------------------------------------RKKR--HYAD--VDNQVYTLEVL   [460]
Caenorhabditis gon-1 CEt13h10 (3879882)         -------------------------------------RSRR-----A--ANSWDHYVEVL   [285]
introns                                         ----------------------------------30,31,32,33--------34,35--   [2]
[                                                                     furin prodomain cleavage motif            ]
```

AC Nicholson, SB Malik, JM Logsdon Jr, E van Meir, 2004. Functional evolution of ADAMTS genes: evidence]
[from analyses of phylogeny and gene organization. Contact: john-logsdon@uiowa.edu]
[Intron phases marked 0, 1, 2]

```
[                                                               610       620       630       640       650       660]
[                                                                .         .         .         .         .         .]
[Modal, ≥80%, full maj.                                     D      HG         Y LT            S                    ]
mask                                          111111111111--1111--1111111-1111111111111111111111111111111   [100]
Homo ADAMTS6 (11131380)                       VVADKMMVGYHGRKDIEH--YILSVMN-IVAKLYRDSSLGNVVNIIVARLIVLTEDQPNL   [312]
Fugu 6                                        VVADKMMVGYHGRKDIEH--YILSVMN-I??????????---------?????????PNL   [316]
Ciona 6 (0100146470)                          VVADKMMMVTHGKQELEK--YILTIMN-IVASFYHDASIGNAINIIITRVIILTEDQPNL   [334]
Homo ADAMTS10 (17432918)                       VVADKMMVAYHGRRDVEQ--YVLAIMN-IVAKLFQDSSLGSTVNILVTRLILLTEDQPTL   [301]
Mus 10 (27502095)                             VVADKMMVAYHGRRDVEQ--YVLAIMN-IVAKLFQDSSLGNIVNILVTRLILLTEDQPTL   [267]
Fugu 10                                       VVADKMMVGYHGRRDIEQ--YILAVMNIQVAKLFQDSSLGNAVNIVVTRLILLMEDQPTL   [266]
Homo ADAMTS16 (21265061)                      VVVDKKMMQNHGHENITT--YVLTILN-MVSALFKDGTIGGNINIAIVGLILLEDEQPGL   [352]
Mus 16 (21961374)                             VVVDRKMMQSHGHENITT--YVLTILN-MVSALFKDGTIGGNINIVIVGLILLEDEQPGL   [350]
Fugu 16                                       VVVDRKMMDNHGHENITT--YVLTVLN-MVSSLFKDGTIGGNINIVIVGLVLLDEEQEGL   [319]
Ciona 16 (0100138085)                         VVADAQMIRNHERDHRDITTYILTVMN-MVSGLFSDRSMDGTIN--VV-LVGIMLLEDNG   [203]
Homo ADAMTS18 (21265067)                      VVADKKMVEKHGKGNVTT--YILTVMN-MVSGLFKDGTIGSDINVVVVSLILLEQEPGGL   [355]
Fugu 18                                       VVADRKMLEKHGRDNVTT--YVLTVMN-MVSSLFKDGTIGSDINIVVVSLLLLEEDPLGL   [102]
Homo ADAMTS12 (13569928)                      VVADTKMIEYHGSENVES--YILTIMN-MVTGLFHNPSIGNAIHIVVVRLILLEEEEQGL   [308]
Mus 12 (27817773)                             VVADTKTVEYHGSENVES--YILTIMN-MVTGLFHSPSIGNLVHIVVVRLILLEEEEQGL   [312]
Fugu 12                                       VVADSKLIDYHGSDNVES--YIFTIMN-MVAGIFHDASIGNAIHVILVRLILLHGEEKGL   [305]
Homo ADAMTS7 (11131377)                       VVADAKMVEYHGQPQVES--YVLTIMN-MVAGLFHDPSIGNPIHITIVRLVLLEDEEEDL   [304]
Fugu 7                                        VVADPKMVEYHGSTAVEN--YILAVMN-IVAGLFGDASIGNAINVVVVRLILLEQDEDDL   [281]
Ciona 7 (genewise.34.12.1)                    LVVDNKMVHYHGK-NIET--YVLTIVN-MVSELFHDVSIGNSLHVTLIRLVLLEGDEKGL   [267]
Homo ADAMTS17 (21265064)                      VVADADMVQYHGAEAAQR--FILTVMN-MVYNMFQHQSLGIKINIQVTKLVLLRQRPAKL   [294]
Homo ADAMTS19 (19525737)                      VVADPAMVSYHGADAARR--FILTILN-MVFNLFQHKSLGVQVNLRVIKLILLHETPPEL   [387]
Drosophila dCG4096 (22831757)                 IVADATMSAFHR--DLNG--YLLTIMN-MVSALYKDPSIGNSIEIVVVRIIQLDEEESQL   [385]
Homo ADAMTS14 (29337086)                      LVVDDSVVRFHGKEHVQN--YVLTLMN-IVDEIYHDESLGVHINIALVRLIMVGYRQSLS   [321]
Fugu 14                                       LAVDDSVVRFHGKEHVQN--YVLTLMN-IVDEIYHDESLGTNINIVLVRMIMVGYRQSIS   [289]
Homo ADAMTS3 (2224673)                        LGVDDSVVRFHGKEHVQN--YLLTLMN-IVNEIYHDESLGVHINVVLVRMIMLGYAKSIS   [318]
Fugu 3                                        LGVDDSVVRFHGKEHVQN--YLLTLMN-IVNEIYHEESLGVHINVVLVRMIMLGYAKSIS   [81]
Ciona 3 (0100146117)                          LTVGHEIVRVHGRHHVEN--YLVTLMN-IVDEIYHDDSLGEHINIILTRIYQLDAVRTNN   [255]
Homo ADAMTS2 (7656867)                        LGVDDSVVQFHGKEHVQK--YLLTLMN-IVNEIYHDESLGAHINVVLVRIILLSYGKSMS   [328]
Mus 2 (28204840)                              LGVDDSVVQFHGKEHVQK--YLLTLMN-IVNEIYHDESLGAHINVVLVRIILLSHAKSMS   [329]
Fugu 2                                        LGVDDSVVQFHRKENVQK--YLLTLMN-IVNEIYHDKSLGAKINVVLVRIIMLGQGKSTS   [99]
Homo ADAMTS13 (16306598)                      VAVGPDVFQAHQE-DTER--YVLTNLN-IGAELLRDPSLGAQFRVHLVKMVILTEPEGAP   [141]
Fugu 13                                       VVVGPDVHQVHKQ-DTER--YILTNLN-IASELLRDISLGGNMRVHLVRMIILSEPEPEI   [74]
Homo ADAMTS1 (6525075)                        LVADQSMAEFHGS-GLKH--YLLTLFS-VAARLYKHPSIRNSVSLVVVKILVIHDEQKGP   [319]
Mus 1 (1813340)                               LVADQSMADFHGS-GLKH--YLLTLFS-VAARFYKHPSIRNSISLVVVKILVIYEEQKGP   [303]
Homo ADAMTS4 (12643637)                       VVADDKMAAFHGA-GLKR--YLLTVMA-AAAKAFKHPSIRNPVSLVVTRLVILGSGEEGP   [279]
Mus 4 (26350615)                              VVADDKMAAFHGT-GLKR--YLLTVMA-AAAKAFKHPSIRNPVNLVVTRLVILGSGQEGP   [287]
Fugu 4                                        IVADQSMAEFHGA-GLKP--YLLTIMA-VASRLYRHPSIHNSISLAVVKLLVVYEEEYGP   [73]
Homo ADAMTS8 (21536392)                       LVADASMAAFYGA-DLQN--HILTLMS-VAARIYKHPSIKNSINLMVVKVLIVEDEKWGP   [281]
Mus 8 (6708110)                               LVADASMAAFYGT-DLQN--HILTVMS-MAARIYKHPSIRNSVNLVVVKVLIVEKERWGP   [295]
Fugu 8                                        VVADPSMTHFYGD-EIKH--YILTLMS-MAAQLYKHPSIKNSVNLVLVKMLVVEDEEVGP   [202]
Homo ADAMTS5 (7768707)                        LVADASMARLYGR-GLQH--YLLTLAS-IANRLYSHASIENHIRLAVVKVVVLGDKDKSL   [328]
Mus 5 (5923784)                               LVADSSMARMYGR-GLQH--YLLTLAS-IANRLYSHASIENHIRLAVVKVVVLTDKDTSL   [328]
Fugu 5                                        LVADDTMTKKYGK-DLNH--YLLTLAS-IASKLYGHASIENPIRLSVVKVTTVGEKEKGL   [280]
Ciona 15 (0100137065)                         VVADASMVEEHGD-DLEH--YILTLMM-VANRVFSHPSLGSAISISVVKVRIMVLHEDSD   [274]
Homo ADAMTS15 (19171175)                      VVADESMVKFHGA-DLEH--YLLTLLA-TAARLYRHPSILNPINIVVVKVLLLRDRDSGP   [279]
Fugu 15                                       VVADESMASAHGE-DLQH--YLLTLMS-VVARLYKHPSILNPISVVVVGVMVLGGADKGP   [230]
Homo ADAMTS9 (27463365)                       VVADNRMVSYHGE-NLQH--YILTLMS-IVASIYKDPSIGNLINIVIVNLIVIHNEQDGP   [354]
Fugu 9                                        LVADSKMVEHHGS-NLQH--YILTLMS-IVSSIYKDPSIGNLINIVVVKLVIINNELEGP   [197]
Homo ADAMTS20 (28460690)                      VTADAKVVSAHGS-NLQN--YILTLMS-IVATIYKDPSIGNLIHIVVVKLVMIHREEEGP   [305]
Mus 20 (29500513)                             VTADAKMVHHHGQ-NLQH--YVLTLMS-IVAAIYKDSSIGNLINIVIVKLVVIHSEQEGP   [316]
Fugu 20                                       VTADAKMARHHGP-NLEH--YILTIMS-VVAAVYRDPSVGNLINIMIVKLIVVHNEQEGP   [287]
Ciona 9 (0100132719)                          VTADNAMLRSHDD--VEH--YVLTLMA-IVNHVYRDPSIENLVNIVLVKLIVIGDSQNGP   [395]
Apis 9                                        VAADAEMVRYHGK-GLLG--YILGLMS-TVSRIYKDPSIGNPISISVMSIVPIDKI-FGV   [197]
Drosophila CG6107 (20151419)                  IAVDNSMKQFHGE-DLQP--YILILMS-IVSSIFADASIGNSIRILLVRLISLPNIN---   [513]
Caenorhabditis gon-1 CEt13h10 (3879882)       VVADTKMYEYHGR-SLED--YVLTLFS-TVASIYRHQSLRASINVVVVKLIVLKTENAGP   [341]
introns                                       -------------------36-------37---------------------38---39--   [2]
```

```
[                                                                    670       680       690       700       710       720]
[                                                                     .         .         .         .         .         .]
[Modal, ≥80%, full maj.                                                   L   C W                       D A   T        ]
mask                                          1--111-1-11111111111111111111---------------1111111111--1111   [139]
Homo ADAMTS6 (11131380)                       E--INH-H-ADKSLDSFCKWQKSILSHQSDGNTIPENG----IAHHDNAVLITR--YDIC   [362]
Fugu 6                                        E--INH-H-ADKSLDSFCKWQRTVLSHHSGGNSIPENG----MAHHDNAVLITR--YDIC   [366]
Ciona 6 (0100146470)                          E--IST-H-AGNTLNSFCEWQAGVNINSTGDSSDSG------LAHHDNAVLLTG--NNIC   [382]
Homo ADAMTS10 (17432918)                      E--ITH-H-AGKSLDSFCKWQKSIVNHSGHGNAIPENG----VANHDTAVLITR--YDIC   [351]
Mus 10 (27502095)                             E--ITH-H-AGKSLDSFCKWQKSIVSHSGHGNAIPENG----VANHDTAVLITR--YDIC   [317]
Fugu 10                                       E--INH-H-AGKSLDSFCKWQKTIQHRNSYGNAIPDNG----IAHHDTAVLIT?------   [312]
Homo ADAMTS16 (21265061)                      V--ISH-H-ADHTLSSFCQWQSGLMGKDG-------------TRHDHAILLTG--LDIC   [392]
Mus 16 (21961374)                             A--ISH-H-ADHTLTSFCQWQSGLMGKDG-------------TRHDHAILLTG--LDIC   [390]
Fugu 16                                       V--INH-H-ADHTLNSFCHWQSTLRGREG-------------RHHDHAILLTG--LDIC   [359]
Ciona 16 (0100138085)                         AL-HLDHK-ADEALSSFCHWQANLNNTNGGKP--------------DHSILLTG--IDIC   [245]
Homo ADAMTS18 (21265067)                      L--INH-H-ADQSLNSFCQWQSALIGKNG-------------KRHDHAILLTG--FDIC   [395]
Fugu 18                                       T--ISH-H-ADQSLNSFCQWQSGLLGKGG-------------KRHDHAVLLTG--LDIC   [142]
Homo ADAMTS12 (13569928)                      K--IVH-H-AEKTLSSFCKWQKSINPKSDLNP-----------VHHDVAVLLTR--KDIC   [351]
Mus 12 (27817773)                             K--IVH-H-AEKTLSSFCKWQKSINPKSDLNP-----------VHHDVAVLITR--KDIC   [355]
Fugu 12                                       K--IAH-H-ADTTLSSFCAWQKNLNPQSDTHP-----------AHHDVAVLITR--KDIC   [348]
Homo ADAMTS7 (11131377)                       K--ITH-H-ADNTLKSFCKWQKSINMKGDAHP-----------LHHDTAILLTR--KDLC   [347]
Fugu 7                                        K--ITH-H-ADNSLHSFCKWQKKLNMKGDNHP-----------LHHDVSVLLTR--KDIC   [324]
Ciona 7 (genewise.34.12.1)                    K--ITQ-H-AERTLDSFCRWAKRMNPKSDNHA-----------NHHDVAVLLTR--RNIC   [310]
Homo ADAMTS17 (21265064)                      S--IGH-H-GERSLESFCHWQNEEYGGARYLGNNQVPGGKDDPPLVDAAVFVTR--TDFC   [348]
Homo ADAMTS19 (19525737)                      Y--IGH-H-GEKMLESFCKWQHEEFGKKNDIHLEMSTNWGEDMTSVDAAILITR--KDFC   [441]
Drosophila dCG4096 (22831757)                 QL-NLTQN-AQKNLDRFCSWQHKLNKGSEKDP-----------HHHDVAILITR--KNIC   [430]
Homo ADAMTS14 (29337086)                      L--IERGN-PSRSLEQVCRWAHSQRQDPSHA-----------EHHDHVVFLTR--QDFG   [365]
Fugu 14                                       L--IERGN-PSRSLEQVCRWANTQQRRDPEHS-----------EYHDHAIFLTR--QDFG   [333]
Homo ADAMTS3 (2224673)                        L--IERGN-PSRSLENVCRWASQQQRSDLNHS-----------EHHDHAIFLTR--QDFG   [362]
Fugu 3                                        L--IERGN-PSRSLENVCRWAFVQQKSDPDHA-----------EHHDHAIFLTR--QGFG   [125]
Ciona 3 (0100146117)                          L--IVENE-PKTSLNNVCHWAKDIQVTDPAPNG----------RTYDAAIFLTR--IRFG   [300]
Homo ADAMTS2 (7656867)                        L--IEIGN-PSQSLENVCRWAYLQQKPDTGHD-----------EYHDHAIFLTR--QDFG   [372]
Mus 2 (28204840)                              L--IEIGN-PSQSLENVCRWAYLQQKPDTDHD-----------EYHDHAIFLTR--QDFG   [373]
Fugu 2                                        L--IEIGN-PSQSLENVCRWAFLQQKQDTGDA-----------EYHDHAIFLTR--QEFG   [143]
Homo ADAMTS13 (16306598)                      N--ITA-N-LTSSLLSVCGWSQTINPEDDTDP-----------GHADLVLYITR--FDLE   [184]
Fugu 13                                       Q--LST-N-ITSSLTSVCEWGRKINPANDSDP-----------LHADLLLYITR--YDLV   [117]
Homo ADAMTS1 (6525075)                        E--VTS-N-AALTLRNFCNWQKQHNPPSDRDA-----------EHYDTAILFTR--QDLC   [362]
Mus 1 (1813340)                               E--VTS-N-AALTLRNFCSWQKQHNSPSDRDP-----------EHYDTAILFTR--QDLC   [346]
Homo ADAMTS4 (12643637)                       Q--VGP-S-AAQTLRSFCAWQRGLNTPEDSDP-----------DHFDTAILFTR--QDLC   [322]
Mus 4 (26350615)                              Q--VGP-S-AAQTLRSFCTWQRGLNTPNDSDP-----------DHFDTAILFTR--QDLC   [330]
Fugu 4                                        Q--VSS-N-AAMTLRNFCQWQRQHNPTSDRHA-----------EHYDTAVLFTR--TDLC   [116]
Homo ADAMTS8 (21536392)                       E--VSD-N-GGLTLRNFCNWQRRFNQPSDRHP-----------EHYDTAILLTR--QNFC   [324]
Mus 8 (6708110)                               E--VSD-N-GGLTLRNFCSWQRRFNKPSDRHP-----------EHYDTAILFTR--QNFC   [338]
Fugu 8                                        E--VSS-N-GGVALRNFCSWQQLFNSPSQRHP-----------EHYDTAMLFTR--EDIC   [245]
Homo ADAMTS5 (7768707)                        E--VSK-N-AATTLKNFCKWQHQHNQLGDDHE-----------EHYDAAILFTR--EDLC   [371]
Mus 5 (5923784)                               E--VSK-N-AATTLKNFCKWQHQHNQLGDDHE-----------EHYDAAILFTR--EDLC   [371]
Fugu 5                                        E--VSKN--AAATLKSFCKWQNQQNSLDDDHQ-----------HHHDAAILFTR--QDLC   [323]
Ciona 15 (0100137065)                         GP-RITQH-AQHTLREFCSWQKRHNTPDDSDP-----------QHYDLAILLTR--TNLC   [319]
Homo ADAMTS15 (19171175)                      K--VTG-N-AALTLRNFCAWQKKLNKVSDKHP-----------EYWDTAILFTR--QDLC   [322]
Fugu 15                                       K--VSS-N-AALTLRNFCSWQKKLNKHSDKHP-----------DYWDTAVLFTK--QDLC   [273]
Homo ADAMTS9 (27463365)                       S--ISF-N-AQTTLKNLCQWQHSKNSPGGI--------------HHDTAVLLTR--QDIC   [394]
Fugu 9                                        V--ISF-N-AQATLKNFCIWQQSQNILDDNHH-----------SHHDTAILITR--QDIC   [240]
Homo ADAMTS20 (28460690)                      V--INF-D-GATTLKNFCSWQQTQNDLDDVHP-----------SHHDTAVLITR--EDIC   [348]
Mus 20 (29500513)                             V--ISF-N-AATTLRNFCLWQQSQNVPDDAHP-----------SHHDTAVLITR--EDIC   [359]
Fugu 20                                       A--VSF-N-AATTLHNFCVWQQGQNVQDDSHP-----------SHHDTALLITR--EDIC   [330]
Ciona 9 (0100132719)                          R--VAR-D-AHTCLRNFCEWQFTQNDPDDSHPD-----------HHDTSVLITG--QDIC   [438]
Apis 9                                        RRTKGEGIAAADMLNKFCRWQQSNNPNEYSPG------------HYDTALLLTR--ENLC   [243]
Drosophila CG6107 (20151419)                  ---DQTHS-SNEMLKHFCQFINQ----SG--------------YERDTAMLITR--EPIC   [549]
Caenorhabditis gon-1 CEt13h10 (3879882)       R--ITQ-N-AQQTLQDFCRWQQYYNDPDDSSV-----------QHHDVAILLTR--KDIC   [384]
introns                                       40----------------41----------------------------------42,43-   [2]
```

[	730 740 750 760 770 780]	
[	. . . . . .]	
[Modal, ≥80%, full maj.	G A C SC G AFT A**HE** **GH** **HD** C]	
mask	-------1111111111111111111111111111111111111111111111111--1	[190]
Homo ADAMTS6 (11131380)	TYKNKP-CGTLGLASVAGMCEPERSCSINEDIGLGSAFTIA**HE**I**GH**NFGMN**HD**GIGNS-C	[420]
Fugu 6	TYKNKP-CGTLGLASVAGMCEPERSCSINEDIGLGSAFTIA**HE**I**GH**NFGMN**HD**GIGNS-C	[424]
Ciona 6 (0100146470)	ANDFAP-CATLGMANVGGICENERSCNINQDTGLASAFTIA**HE**I**GH**NFDME**HD**GTNND-C	[440]
Homo ADAMTS10 (17432918)	IYKNKP-CGTLGLAPVGGMCERERSCSVNEDIGLPQAFTIA**HE**I**GH**TFGMN**HD**GVGNS-C	[409]
Mus 10 (27502095)	IYKNKP-CGTLGLAPVGGMCERERSCSINEDIGLATAFTIA**HE**I**GH**TFGMN**HD**GVGNG-C	[375]
Fugu 10	---------??????VGGMCVSERSCSINEDIGLGTAFTIA**HE**I**GH**TFGMN**HD**GMGNA-C	[362]
Homo ADAMTS16 (21265061)	SWKNEP-CDTLGF*APIS*GMCSKYRSCTINEDTGLGLAFTIA**HE**S**GH**NFGMI**HD**GEGNM-C	[450]
Mus 16 (21961374)	SWKNEP-CDTLGF*APIS*GMCSKYRSCTVNEDSGLGLAFTIA**HE**S**GH**NFGMV**HD**GEGNM-C	[448]
Fugu 16	SWKNEP-CDTLGF*APIS*GMCSRYRSCTINEDTGLGLAFTIA**HE**S**GH**NFGMV**HD**GEGNV-C	[417]
Ciona 16 (0100138085)	VDKNKP-CETLGLAQIGGMCSERNSCTINEDMGLGLAFTVA**HE**S**GH**SFGMK**HD**GEGNL-C	[303]
Homo ADAMTS18 (21265067)	SWKNEP-CDTLGF*APIS*GMCSKYRSCTINEDTGLGLAFTIA**HE**S**GH**NFGMI**HD**GEGNP-C	[453]
Fugu 18	SWKNEP-CDTLGF*APIS*GMCSKYRSCTINEDTGLGLAFTIA**HE**S**GH**NFGMI**HD**GEGNP-C	[200]
Homo ADAMTS12 (13569928)	AGFNRP-CETLGLSHLSGMCQPHRSCNINEDSGLPLAFTIA**HE**L**GH**SFGIQ**HD**GKEND-C	[409]
Mus 12 (27817773)	AGVNRP-CETLGLSQLSGMCQPHRSCNINEDSGLPLAFTIA**HE**L**GH**SFGIQ**HD**GKEND-C	[413]
Fugu 12	AGMNQP-CETLGLSHLSGMCQPHRSCNINEDSGLPVAFTVA**HE**M**GH**SFGIH**HD**GQGND-C	[406]
Homo ADAMTS7 (11131377)	AAMNRP-CETLGLSHVAGMCQPHRSCSINEDTGLPLAFTVA**HE**L**GH**SFGIQ**HD**GSGND-C	[405]
Fugu 7	AAINMP-CETLGLSHVAGMCQAHRSCSISEDTGLPVAFTVA**HE**L**GH**NFGIQ**HD**GNGND-C	[382]
Ciona 7 (genewise.34.12.1)	SRSNKS-CETLGLAHVAGMCQSHRSCNVNQDTGLSLAFTVA**HE**L**GH**NFAMH**HD**GQLNH-C	[368]
Homo ADAMTS17 (21265064)	VHKDEP-CDTVGIAYLGGVCSAKRKCVLAEDNGLNLAFTIA**HE**L**GH**NLGMN**HD**DDHSS-C	[406]
Homo ADAMTS19 (19525737)	VHKDEP-CDTVGIAYLSGMCSEKRKCIIAEDNGLNLAFTIA**HE**M**GH**NMGIN**HD**NDHPS-C	[499]
Drosophila dCG4096 (22831757)	ANN----CMTLGLANVGGMCKPKQSCSVNEDNGIMLSHTIT**HE**L**GH**NFGMF**HD**TAKIG-C	[485]
Homo ADAMTS14 (29337086)	P------SGMQGYAPVTGMCHPLRSCALNHEDGFSSAFVIA**HE**T**GH**VLGME**HD**GQGNG-C	[418]
Fugu 14	P------AGMQGYAPVTGMCHPLRSCTLNHEDGFSSAFVVA**HE**T**GH**VLGME**HD**GQGNR-C	[386]
Homo ADAMTS3 (2224673)	P------AGMQGYAPVTGMCHPVRSCTLNHEDGFSSAFVVA**HE**T**GH**VLGME**HD**GQGNR-C	[415]
Fugu 3	P------TGMQGYAPVTGMCHPVRSCTLNHEDGFSSAFVVA**HE**T**GH**VLGME**HD**GQGNR-C	[178]
Ciona 3 (0100146117)	TAGM---GIKEGYAHVDGLCQPLRSCSLTYEDGFSSAFVVA**HE**T**GH**ILGMK**HD**GDFNR-C	[356]
Homo ADAMTS2 (7656867)	P------SGMQGYAPVTGMCHPVRSCTLNHEDGFSSAFVVA**HE**T**GH**VLGME**HD**GQGNR-C	[425]
Mus 2 (28204840)	P------SGMQGYAPVTGMCHPVRSCTLNHEDGFSSAFVVA**HE**T**GH**VLGME**HD**GQGNR-C	[426]
Fugu 2	P------TGMQGYAPVTGMCHPVRSCTLNHEDGFSSAFVVA**HE**T**GH**VLGME**HD**GQGNR-C	[196]
Homo ADAMTS13 (16306598)	LPDGN--RQVRGVTQLGGACSPTWSCLITEDTGFDLGVTIA**HE**I**GH**SFGLE**HD**GAPGSGC	[242]
Fugu 13	LPDGN--NQVRGVAQLGGACSSEWSCVITEDTGFDLGITIT**HE**I**GH**SFGIN**HD**GVRNT-C	[174]
Homo ADAMTS1 (6525075)	-GSQT--CDTLGMADVGTVCDPSRSCSVIEDDGLQAAFTTA**HE**L**GH**VFNMP**HD**DAKQ--C	[417]
Mus 1 (1813340)	-GSHT--CDTLGMADVGTVCDPSRSCSVIEDDGLQAAFTTA**HE**L**GH**VFNMP**HD**DAKH--C	[401]
Homo ADAMTS4 (12643637)	-GVST--CDTLGMADVGTVCDPARSCAIVEDDGLQSAFTAA**HE**L**GH**VFNML**HD**NSKP--C	[377]
Mus 4 (26350615)	-GVST--CDTLGMADVGTVCDPARSCAIVEDDGLQSAFTAA**HE**L**GH**VFNML**HD**NSKP--C	[385]
Fugu 4	-GAHS--CDTLGMADVGTVCDPDRSCSIIEDDGLQAAFTVA**HE**L**GH**VFNMP**HD**DAQL--C	[171]
Homo ADAMTS8 (21536392)	-GQEGL-CDTLGVADIGTICDPNKSCSVIEDEGLQAAHTLA**HE**L**GH**VLSMP**HD**DSKP--C	[380]
Mus 8 (6708110)	-GKGEQ-CDTLGMADVGTICDPDKSCSVIKDEGLQAAYTLA**HE**L**GH**VLSMP**HD**DSKP--C	[394]
Fugu 8	-GQKS--CDTLGVADVGTMCDPKRSCSVIEDNGLQAAFTAA**HE**L**GH**VLSMP**HD**DSKT--C	[300]
Homo ADAMTS5 (7768707)	-GHHS--CDTLGMADVGTICSPERSCAVIEDDGLHAAFTVA**HE**I**GH**LLGLS**HD**DSKF--C	[426]
Mus 5 (5923784)	-GHHS--CDTLGMADVGTICSPERSCAVIEDDGLHAAFTVA**HE**I**GH**LLGLS**HD**DSKF--C	[426]
Fugu 5	-GHHS--CDTLGMADVGTICSPERSCAVIEDDGLHAAFTVA**HE**I**GH**LLGLS**HD**DSKF--C	[378]
Ciona 15 (0100137065)	-GDT---CATLGLAEVGTMCSRRHSCAVIEDDGLSASYTIA**HE**V**GH**VLNMM**HD**DNRL--C	[373]
Homo ADAMTS15 (19171175)	-GATT--CDTLGMADVGTMCDPKRSCSVIEDDGLPSAFTTA**HE**L**GH**VFNMP**HD**NVKV--C	[377]
Fugu 15	-GATT--CDTLGMADVGTMCDPKRSCSVIEDDGLPTAFTAA**HE**L**GH**VFNMP**HD**NVKA--C	[328]
Homo ADAMTS9 (27463365)	-RAHDK-CDTLGLAELGTICDPYRSCSISEDSGLSTAFTIA**HE**L**GH**VFNMP**HD**DNNK--C	[450]
Fugu 9	-RARDK-CDTLGLAELGTVCDPYRSCSIIEDNGLSTAFTIA**HE**L**GH**VFNMP**HD**DSNK--C	[296]
Homo ADAMTS20 (28460690)	-SSKEK-CNMLGLSYLGTICDPLQSCFINEEKGLISAFTIA**HE**L**GH**TLGVQ**HD**DNPR--C	[404]
Mus 20 (29500513)	-GAKEK-CDTLGLAELGTLCDPSRSCSISEENGLSAAFTIA**HE**L**GH**VFNVP**HD**DSFK--C	[415]
Fugu 20	-RAKDK-CDTLGLAELGTMCDQYRSCSISEENGISASFTIA**HE**L**GH**VFNMP**HD**DNPK--C	[386]
Ciona 9 (0100132719)	-RSNSV-CDTLGLAELGTMCDLRKSCSINEDNGLSTAFTIA**HE**I**GH**EFNAP**HD**NNDK--C	[494]
Apis 9	HNSDQRNCDTLGLAELGRMCSPRSSCAIVQDNGLAAAFTIA**HE**I**GH**VLDMP**HD**DDVK--C	[301]
Drosophila CG6107 (20151419)	GSVPGKICHMLGLAELGTVCS-SSSCSIVQDTGLPTAFTMA**HE**L**GH**ILNMN**HD**DDDK--C	[606]
Caenorhabditis gon-1 CEt13h10 (3879882)	-RSQGK-CDTLGLAELGTMCDMQKSCAIIEDNGLSAAFTIA**HE**L**GH**VFSIP**HD**DERK--C	[440]
introns	--------44,45----------------------------46,47,48----------	[2]

```
[                                                      790       800       810       820       830       840]
[                                                        .         .         .         .         .         .]
[Modal, ≥80%, full maj.                                                     M                WS CS       L    ]
mask                                          --------------------------111111111--111111111111111111111--   [220]
Homo ADAMTS6 (11131380)                       -GTKG-----------------HEAAKLMAAHITA--NTNPFSWSACSRDYITSFLDSGR   [460]
Fugu 6                                        -GTKG-----------------HETAKLMAAHITA--NTNPFSWSACSKDYITSFLDSGR   [464]
Ciona 6 (0100146470)                          -GPRN-----------------GEPSQIMSPQLTK--YTSPFSWSSCSKKYITDYLDSGS   [480]
Homo ADAMTS10 (17432918)                      -GARG-----------------QDPAKLMAAHITM--KTNPFVWSSCNRDYITSFLDSGL   [449]
Mus 10 (27502095)                             -GARG-----------------QDPAKLMAAHITM--KTNPFVWSSCSRDYITSFLDSGL   [415]
Fugu 10                                       -GPHS-----------------QETAKLMADHITM--KTNPFIWSACSRDYITSFLDSGM   [402]
Homo ADAMTS16 (21265061)                      KKSEG---------------------NIMSPTLAG--RNGVFSWSPCSRQYLHKFLSTAQ   [487]
Mus 16 (21961374)                             KKSEG---------------------NIMSPTLAG--RNGVFSWSSCSRQYLHKFLSTAQ   [485]
Fugu 16                                       KKSEG---------------------NIMSPTLAG--HNGIFSWSACSRQYLSRFLNSAQ   [454]
Ciona 16 (0100138085)                         SKRDG---------------------HIMSPTLNG--LNGVFTWSTCSRNAIGDFLQNVE   [340]
Homo ADAMTS18 (21265067)                      RKAEG---------------------NIMSPTLTG--NNGVFSWSSCSRQYLKKFLSTPQ   [490]
Fugu 18                                       RKTEG---------------------NIMSPTLAG--NNGVFFWSTCSRQYLSRFLGTTQ   [237]
Homo ADAMTS12 (13569928)                      -EPVG------------------RHPYIMSRQLQY--DPTPLTWSKCSEEYITRFLDRGW   [448]
Mus 12 (27817773)                             -EPVG------------------RHPYIMSQQIQY--DPTPLTWSKCSKEYITRFLDRGR   [452]
Fugu 12                                       -ELEG------------------RHPFIMSRQLMY--DSSPLTWSSCSKEYITRFLDRGW   [445]
Homo ADAMTS7 (11131377)                       -EPVG------------------KRPFIMSPQLLY--DAAPLTWSRCSRQYITRFLDRGW   [444]
Fugu 7                                        -EPVG------------------KRPFVMSPQLLY--GTSLPQWSRCSREYITRFLDRGW   [421]
Ciona 7 (genewise.34.12.1)                    -TPDE------------------ANPRVMTPHLTS--NNLPMRWSTCSRKYITRFLDRNW   [407]
Homo ADAMTS17 (21265064)                      ----------------------AGRSHIMSGEWVKGRNPSDLSWSSCSRDDLENFLKSKV   [444]
Homo ADAMTS19 (19525737)                      ----------------------ADGLHIMSGEWIKGQNLGDVSWSRCSKEDLERFLRSKA   [537]
Drosophila dCG4096 (22831757)                 HPRVGP------------------IVHIMTPTFGA--DTLQVCWSNCSRKYITHFLDQGL   [525]
Homo ADAMTS14 (29337086)                      -----D---------------ETSLGSVMAPLVQA--AFHRFHWSRCSKLELSRYLPS--   [454]
Fugu 14                                       ----AD---------------ETSMGSIMAPLVQA--AFHRYHWSRCSKQELNRYIHS--   [423]
Homo ADAMTS3 (2224673)                        ----GD---------------ETAMGSVMAPLVQA--AFHRYHWSRCSGQELKRYIHS--   [452]
Fugu 3                                        ----GD---------------ETAMGSVMAPLVQA--AFHRYHWSMCSGQELKRYIHT--   [215]
Ciona 3 (0100146117)                          ----GD---------------ETTMGSIMAPLVQA--AFHRYHWSRCSQEELRSSLGS--   [393]
Homo ADAMTS2 (7656867)                        ----GD---------------EVRLGSIMAPLVQA--AFHRFHWSRCSQQELSRYLHS--   [462]
Mus 2 (28204840)                              ----GD---------------EVRLGSIMAPLVQA--AFHRFHWSRCSQQELSRYLHS--   [463]
Fugu 2                                        ----GD---------------EVHMGSIMAPLVQA--AFHRFHWSRCSMQELGRYLHS--   [233]
Homo ADAMTS13 (16306598)                      ----------------------GPSGHVMASDGAA--PRAGLAWSPCSRRQLLSLLSAGR   [278]
Fugu 13                                       ----------------------SRSGFIMASDGGY--NSVDLTWSPCSRQQLLAFFSDGK   [210]
Homo ADAMTS1 (6525075)                        ASLNG----------------VNQDSHMMASMLSN--LDHSQPWSPCSAYMITSFLDNGH   [459]
Mus 1 (1813340)                               ASLNG----------------VSGDSHLMASMLSS--LDHSQPWSPCSAYMVTSFLDNGH   [443]
Homo ADAMTS4 (12643637)                       ISLNGP---------------LSTSRHVMAPVMAH--VDPEEPWSPCSARFITDFLDNGY   [420]
Mus 4 (26350615)                              TNLNGQ---------------GGSSRHVMAPVMAH--VDPEEPWSPCSARFITDFLDNGY   [428]
Fugu 4                                        SEVNG----------------AHWGSHMMASTLSN--LDQQQPWSPCSALMVTTFLDNGH   [213]
Homo ADAMTS8 (21536392)                       TRLFG----------------PMGKHHVMAPLFVH--LNQTLPWSPCSAMYLTELLDGGH   [422]
Mus 8 (6708110)                               VRLFG----------------PMGKYHMMAPFFIH--VNKTLPWSPCSAVYLTELLDDGH   [436]
Fugu 8                                        QRLFG----------------DLGGHFLMAPVLVS--LNKTAPWSPCSALYVTEFFDNGH   [342]
Homo ADAMTS5 (7768707)                        EETFG----------------STEDKRLMSSILTS--IDASKPWSKCTSATITEFLDDGH   [468]
Mus 5 (5923784)                               EENFG----------------TTEDKRLMSSILTS--IDASKPWSKCTSATITEFLDDGH   [468]
Fugu 5                                        EERFG----------------VNSDKRLMSSILTS--IDASKPWSRCTSATITDFFDDGN   [420]
Ciona 15 (0100137065)                         RANFA---------------NIDSTSHVMSPTMDR--VDSEQPWSLCSKQALTDFLEDGG   [416]
Homo ADAMTS15 (19171175)                      EEVFG----------------KLRANHMMSPTLIQ--IDRANPWSACSAAIITDFLDSGH   [419]
Fugu 15                                       EEVFG----------------KLKDNHMMSPTLIQ--IDRSRPWSVCSAAIITEFLDRGH   [370]
Homo ADAMTS9 (27463365)                       -KEEG----------------VKSPQHVMAPTLNF--YTNPWMWSKCSRKYITEFLDTGY   [491]
Fugu 9                                        -KEDG----------------VKLQQHVMAPTLNY--NTNPWMWSKCSRKYITEFLDTGY   [337]
Homo ADAMTS20 (28460690)                      -KEM-----------------KVTKYHVMAPALSF--HMSPWSWSNCSRKYVTEFLDTGY   [444]
Mus 20 (29500513)                             -KEAG----------------IKHQYHVMAPTLNY--HTSPWTWSACSQKHITEFLDTGH   [456]
Fugu 20                                       -WEGG----------------VKRQYHVMASTLNY--DTNPWTWSKCSRKYITEFLDTGY   [427]
Ciona 9 (0100132719)                          LDARGAT---------------VGL-NVMSPTLDNN--AHPWSWSKCSAKYITSFLDNGN  [536]
Apis 9                                        AAYKNRS----------------GMHNIMSRMLDDN--TFPWEWSKCSRHYVTEFLEAGK  [343]
Drosophila CG6107 (20151419)                  MPYVTRQN-------------NNKVLHIMSSVMGI--HMHPWSWSKCSRHFVSEFLEKTD   [651]
Caenorhabditis gon-1 CEt13h10 (3879882)       STYMPVNKVCKFQSTKFDKTQFQNNFHIMAPTLEY--NTHPWSWSPCSAGMLERFLENNR   [498]
introns                                       --------------------------------49---------------------50,51   [2]
```

AC Nicholson, SB Malik, JM Logsdon Jr, E van Meir, 2004. Functional evolution of ADAMTS genes: evidence]
[from analyses of phylogeny and gene organization. Contact: john-logsdon@uiowa.edu]
[Intron phases marked 0, 1, 2]

```
[                                                   850       860       870       880       890       900]
[                                                     .         .         .         .         .         .]
[Modal, ≥80%, full maj.                        CL    P          P   PG    Y    QC    G     C           ]
mask                                       11--11111-1----1111111-1111--11111111111111111111-------1---   [261]
Homo ADAMTS6 (11131380)                    GT--CLDNE-P---PKRDFLYPAVAPG--QVYDADEQCRFQYGATSRQCK----YG-E---   [505]
Fugu 6                                     GT--CLDNE-P---LKRDFLYPTVAPG--QVYEADEQCRFQYGASSRQCK----YG-E---   [509]
Ciona 6 (0100146470)                       GD--CLLNT-P---PQRDFDFPSELPG--QLHDADEQCRLQYGASSRQCK----YG-D---   [525]
Homo ADAMTS10 (17432918)                   GL--CLNNR-P---PRQDFVYPTVAPG--QAYDADEQCRFQHGVKSRQCK----YG-E---   [494]
Mus 10 (27502095)                          GL--CLNNR-P---PRQDFVYPTVAPG--QAYDADEQCRFQHGVKSRQCK----YG-E---   [460]
Fugu 10                                    GS--CLNNI-P---PKQEFVYPTTAPG--QAYDADEQCRFQYGVRSRQCK----YA-E---   [447]
Homo ADAMTS16 (21265061)                   AI--CLADQ-P--KPVKEYKYPEKLPG--ELYDANTQCKWQFGEKAKLCMLDFKKA-D---   [537]
Mus 16 (21961374)                          AI--CLADQ-P--KPVKEYKYPEKLPG--QLYDANTQCKWQFGEKAKLCMLDFRK--D---   [534]
Fugu 16                                    SL--CLSDE-P--RAAKEYRYPEKLPG--ELYDADTQCKWQFGEKAKLCTLDFKK--D---   [503]
Ciona 16 (0100138085)                      SQ--CLDDH-P--SPMANLNFPDQLPG--EIYDAASQCKWQFGADAITCIFRYEHKQA---   [391]
Homo ADAMTS18 (21265067)                   AG--CLVDE-P--KQAGQYKYPDKLPG--QIYDADTQCKWQFGAKAKLCSLGFVK--D---   [539]
Fugu 18                                    AS--CLVDE-P--KQIGQYKYPEKLPG--QLYDADTQCKWQFGSKAKLCSLDFVK--D---   [286]
Homo ADAMTS12 (13569928)                   GF--CLDDI-P---KKKGLKSKVIAPG--VIYDVHHQCQLQYGPNATFCQ---EVE-N---   [494]
Mus 12 (27817773)                          GF--CLDDI-P---SKKGLKSNVIAPG--VIYDVHHQCQLQYGPNATFCQ---EVE-N---   [498]
Fugu 12                                    GF--CLDDR-P---SKRDLTTPLARLG--VRYTTHHQCQLQYGPNATFCN---EVD-N---   [491]
Homo ADAMTS7 (11131377)                    GL--CLDDP-P---AKDIIDFPSVPPG--VLYDVSHQCRLQYGAYSAFCE---DMD-N---   [490]
Fugu 7                                     GW--CLDDA-P---VKDKHSWNSVLPG--VVYTAVHQCRLQYGPSSRLCD---DMD-N---   [467]
Ciona 7 (genewise.34.12.1)                 GF--CLMDT-P-SSELTPFPYPSELPG--VLYDADHQCRLMFGAKSRHCK---GLD-D---   [454]
Homo ADAMTS17 (21265064)                   ST--CLLVTDP--RSQHTVRLPHKLPG--MHYSANEQCQILFGMNATFCR---NME-HL--   [493]
Homo ADAMTS19 (19525737)                   SN--CLLQTNP--QSVNSVMVPSKLPG--MTYTADEQCQILFGPLASFCQ---EMQ-HV--   [586]
Drosophila dCG4096 (22831757)              GE--CLDDP-P--TPLDEYNYTGELPG--MRYNARGQCRLQFNLTTDSEV---GAC-SAPH   [575]
Homo ADAMTS14 (29337086)                   YD--CLLDD-P--FDPAWPQPP-ELPG--INYSMDEQCRFDFGSGYQTCL-----A-FRTF   [501]
Fugu 14                                    YD--CLLDD-P--FEHKWPKLP-ELPG--INYSMDEQCRFDFGIGYKMCT-----A-FRTY   [470]
Homo ADAMTS3 (2224673)                     YD--CLLDD-P--FDHDWPKLP-ELPG--INYSMDEQCRFDFGVGYKMCT-----A-FRTF   [499]
Fugu 3                                     YD--CLLDD-P--FKHDWPQLP-ELPG--INYSMDEQCRFDFGVGYKICT-----S-FRTF   [262]
Ciona 3 (0100146117)                       YT--CLSDD-P--HLQDWGPTP-LYPG--VAYSVDDQCRFDFGRGYKRCT-----S-LQNI   [440]
Homo ADAMTS2 (7656867)                     YD--CLLDD-P--FAHDWPALP-QLPG--LHYSMNEQCRFDFGLGYMMCT-----A-FRTF   [509]
Mus 2 (28204840)                           YD--CLRDD-P--FAHDWPALP-QLPG--LHYSMNEQCRFDFGLGYMMCT-----A-FRTF   [510]
Fugu 2                                     YD--CLRDD-P--FDHNWPSLP-QLPG--LHYSMNEQCRFDFGTGYTMCT-----A-YRTF   [280]
Homo ADAMTS13 (16306598)                   AR--CVWDP-PRPQPGSAGHPPDAQPG--LYYSANEQCRVAFGPKAVACTFAREHL-D---   [329]
Fugu 13                                    AE--CVKDL-P----VLEGSLQDWKPG--LYYGVDDQCRIAFGSAARACSFTNSDL-P---   [257]
Homo ADAMTS1 (6525075)                     GE--CLMDK-P----HNPIQLPGDLPG--TSYDANRQCQFTFGEDSKHCP----DA-AS--   [504]
Mus 1 (1813340)                            GE--CLMDK-P----QNPIKLPSDLPG--TLYDANRQCQFTFGEESKHCP----DA-AS--   [488]
Homo ADAMTS4 (12643637)                     GH--CLLDK-P----EAPLHLPVTFPG--KDYDADRQCQLTFGPDSRHCP----QL-PP--   [465]
Mus 4 (26350615)                           GH--CLLDK-P----EAPLHLPATFPG--KDYDADRQCQLTFGPDSSHCP----QL-PP--   [473]
Fugu 4                                     GQ--CLLDK-P----VRPQALPQPLPG--TVYDADHQCRLTFGEDSQHCP----DL-ST--   [258]
Homo ADAMTS8 (21536392)                     GD--CLLDA-P----GAALPLPTGLPGRMALYQLDQQCRQIFGPDFRHCP--NTSA-QD--   [471]
Mus 8 (6708110)                            GD--CLLDA-P----TSVLPLPTGLPGHSTLYELDQQCKQIFGPDFRHCP--NTSV-ED--   [485]
Fugu 8                                     GD--CLLDV-P----DNTMSLPRELPG--ATYSLDQQCQQVFGEDFLHCP--NNSD-ND--   [389]
Homo ADAMTS5 (7768707)                     GN--CLLDL-P----RKQILGPEELPG--QTYDATQQCNLTFGPEYSVCP-----G-MD--   [512]
Mus 5 (5923784)                            GN--CLLDL-P----RKQILGPEELPG--QTYDATQQCNLTFGPEYSVCP-----G-MD--   [512]
Fugu 5                                     AE--CLLDS-P----RQPLLGSEELPG--QSYDAVRQCRLAFGPEYTVCP-----G-MD--   [464]
Ciona 15 (0100137065)                      GA--CLLDR-P----QEPRVYPSQYPG--AMYDVNAQCKMAFGEGAVECP-----Y-MR--   [460]
Homo ADAMTS15 (19171175)                   GD--CLLDQ-P----SKPISLPEDLPG--ASYTLSQQCELAFGVGSKPCP-----Y-MQ--   [463]
Fugu 15                                    GE--CLLDP-P----QKQLSLPNNLPG--FSYNLHRQCQLAFGPGSKPCP-----F-MQ--   [414]
Homo ADAMTS9 (27463365)                     GE--CLLNE-P---ESRPYPLPVQLPG--ILYNVNKQCELIFGPGSQVCP-----Y-MM--   [536]
Fugu 9                                     GE--CLLDE-P---ISRPYTLPQQLPG--QIYNVNKQCELIFGPETQVCP-----Y-MM--   [382]
Homo ADAMTS20 (28460690)                   GE--CLLDK-P---DEEIYNLPSELPG--SRYDGNKQCELAFGPGSQMCP----HI-EN--   [490]
Mus 20 (29500513)                          GE--CLLDK-P---NGRTYDLSPQLPG--SVYDGNRQCELMFGPGSQVCP----YL-K---   [501]
Fugu 20                                    GE--CLLDE-P---VGRTYELPTLLPG--QIYNANRQCELMFGPGSQICP----YL-K---   [472]
Ciona 9 (0100132719)                       GQ--CLLDE-P---PQRVLSLPTTEPG--QVFDANAQCSFVYGSGVRHCD----FM-FQN-   [583]
Apis 9                                     GD--CLLDT-P--DKIMERTDPRRLPG--EDYSVNKQCELVFGNGSRICN----HM-VGDG   [392]
Drosophila CG6107 (20151419)               KS--CLETS-V---GAHIPYGTERLPG--EIYSLDAQCQLSFGNDFGYCP----TD-E---   [696]
Caenorhabditis gon-1 CEt13h10 (3879882)    GQTQCLFDQ-PVERRYYEDVFVRDEPG--KKYDAHQQCKFVFGPASELCP----YM-P---   [548]
introns                                    -----------------52-------------------------------------53--   [2]
```

```
[                                                     910       920       930       940       950       960]
[                                                     .         .         .         .         .         .]
[Modal, ≥80%, full maj.                           C  LWC          C T     P   GT C         C  G C         ]
mask                                         ---111111111--------11111--11111111111----111111111111------   [298]
Homo ADAMTS6 (11131380)                      ---VCRELWCLSK--SNR--CVTNS--IPAAEGTLCQTGNIEKGWCYQGDCVPFGTW-PQ   [555]
Fugu 6                                       ---VCRELWCLSK--SNR--CVTNS--IPAAEGTLCQTGSIDKGWCYQGECVAFGTF-PQ   [559]
Ciona 6 (0100146470)                         ---VCRELWCISK--LGY--CVTNS--MPAATGTNCSEVN--RGWCYRGHCVPYGYR-AD   [573]
Homo ADAMTS10 (17432918)                     ---VCSELWCLSK--SNR--CITNS--IPAAEGTLCQTHTIDKGWCYKRVCVPFGSR-PE   [544]
Mus 10 (27502095)                            ---VCSELWCLSK--SNR--CITNS--IPAAEGTLCQTHTIDKGWCYKRVCVPFGSR-PE   [510]
Fugu 10                                      ---VCSELWCMSK--SNR--CITSS--IPAAEGTICQTNTIEKGWCYKRMCVAYGTR-PE   [497]
Homo ADAMTS16 (21265061)                     ---ICKALWCHRI--GRK--CETKF--MPAAEGTICGH----DMWCRGGQCVKYGDEGPK   [584]
Mus 16 (21961374)                            ---ICKALWCHRI--GRK--CETKF--MPAAEGTLCGQ----DMWCRGGQCVKYGDEGPK   [581]
Fugu 16                                      ---ICKALWCHRV--GRK--CETKF--MPAAEGSACGP----DMWCRRGQCVKRGDEGPR   [550]
Ciona 16 (0100138085)                        --SVCKVLWCQRS-ESSR--CETKY--MPAAEGTNCGN----NMWCRRGVCVTYGDPGPD   [440]
Homo ADAMTS18 (21265067)                     ---ICKSLWCHRV--GHR--CETKF--MPAAEGTVCGL----SMWCRQGQCVKFGELGPR   [586]
Fugu 18                                      ---ICKSLWCHRT--GHR--CETKF--MPAAEGTTCGP----DMWCRRGQCVKFGEHGPK   [333]
Homo ADAMTS12 (13569928)                     ---VCQTLWCSV---KGF--CRSKL--DAAADGTQCGE----KKWCMAGKCITVGKK-PE   [539]
Mus 12 (27817773)                            ---VCQTLWCSV---KGF--CRSKL--DAAADGTRCGE----KKWCMAGKCITVGKK-PE   [543]
Fugu 12                                      ---VCQILWCSV---NGS--CRSKL--DSPIDGTRCGP----EKWCISGECVIVGKL-PE   [536]
Homo ADAMTS7 (11131377)                      ---VCHTLWCSV---GTT--CHSKL--DAAVDGTRCGE----NKWCLSGECVPVGFR-PE   [535]
Fugu 7                                       ---VCSTLWCTV---GTT--CHSKL--DGAVDGTSCGE----DKWCFRGECLHVGHQ-PA   [512]
Ciona 7 (genewise.34.12.1)                   ---MCNRLWCLY---RGS--CRSRL--HSAVQGTKCGR----GRWCYGGKCVRVGKR-AA   [500]
Homo ADAMTS17 (21265064)                     ---MCAGLWCLVE--GDTS-CKTKL--DPPLDGTECGA----DKWCRAGECVSKTPI-PE   [540]
Homo ADAMTS19 (19525737)                     ---ICTGLWCKVE--GEKE-CRTKL--DPPMDGTDCDL----GKWCKAGECTSRTSA-P-   [632]
Drosophila dCG4096 (22831757)                --EFCSTLWCKVN--GE---CVTHM--RPTAPGTLCGR----NKWCQNGKCVRREEL---   [619]
Homo ADAMTS14 (29337086)                     --EPCKQLWCSHPD-NPYF-CKTKK--GPPLDGTECAP----GKWCFKGHCIWKS--PEQ   [549]
Fugu 14                                      --DPCKQLWCSHPD-NQYF-CKTKK--GPPVDGTECAP----GKWCFKGHCIWRSSQEPQ   [520]
Homo ADAMTS3 (2224673)                       --DPCKQLWCSHPD-NPYF-CKTKK--GPPLDGTECAA----GKWCYKGHCMWK---NAN   [546]
Fugu 3                                       --DPCKQLWCSHPD-NPYF-CKTKK--GPPLDGTECAP----GKWCYKGHCMWK---NPN   [309]
Ciona 3 (0100146117)                         --DVCKTLWCSNDE-NPDI-CKTKK--GPAVDGTECGD----NMWCFKGHCVWRD---QR   [487]
Homo ADAMTS2 (7656867)                       --DPCKQLWCSHPD-NPYF-CKTKK--GPPLDGTMCAP----GKHCFKGHCIWLT---PD   [556]
Mus 2 (28204840)                             --DPCKQLWCSHPD-NPYF-CKTKK--GPPLDGTMCAP----GKHCFKGHCIWLT---PD   [557]
Fugu 2                                       --DPCKQLWCSHPE-NPFF-CKTKK--GPPIDGTTCGN----GKHCFKGHCVWLT---AD   [327]
Homo ADAMTS13 (16306598)                     ---MCQALSCHTDPLDQSS-CSRLL--VPLLDGTECGV----EKWCSKGRCRSLVELTP-   [379]
Fugu 13                                      ---ACRTLSCHVNPGDDSS-CKRLL--VPLLDGTECAP----HRWCLRGHCVSTEELSS-   [307]
Homo ADAMTS1 (6525075)                       ---TCSTLWCTGTSGGVLV-CQTKH--FPWADGTSCGE----GKWCINGKCVNKTDRKHF   [554]
Mus 1 (1813340)                              ---TCTTLWCTGTSGGLLV-CQTKH--FPWADGTSCGE----GKWCVSGKCVNKTDMKHF   [538]
Homo ADAMTS4 (12643637)                      ---PCAALWCSGHLNGHAM-CQTKH--SPWADGTPCGP----AQACMGGRCLHMDQLQDF   [515]
Mus 4 (26350615)                             ---PCAALWCSGHLNGHAM-CQTKH--SPWADGTPCGS----SQACMGGRCLHVDQLKDF   [523]
Fugu 4                                       ---TCAALWCTVTTSNGLLVCQTKN--FPWADGTPCGH----DSYCLAGQCLTKSQAAKH   [309]
Homo ADAMTS8 (21536392)                      ---VCAQLWCHTDGAEPL--CHTKNGSLPWADGTPCGP----GHLCSEGSCLPEEEVERP   [522]
Mus 8 (6708110)                              ---ICVQL-CARHRDSDEPICHTKNGSLLWADGTPCGP----GHLCLDGSCVLKEDVENP   [537]
Fugu 8                                       ---ACGQLWCQEDGTLQ---CLTRNGSLPWADGTPCGP----NRTCLHGACTPTQEVMQP   [439]
Homo ADAMTS5 (7768707)                       ---VCARLWCAVVRQGQMV-CLTKK--LPAVEGTPCGK----GRICLQGKCVDKTKKKYY   [562]
Mus 5 (5923784)                              ---VCARLWCAVVRQGQMV-CLTKK--LPAVEGTPCGK----GRVCLQGKCVDKTKKKYY   [562]
Fugu 5                                       ---VCSRLWCAVIRQGQMV-CLTKK--LPAVEGTPCGK----GRICLQGKCVDKTRKRHY   [514]
Ciona 15 (0100137065)                        ---ECGRLWCQFPTGSRANRCHTRS--FPRADGSDCAA----GMHCMQGRCVAKVAAV--   [509]
Homo ADAMTS15 (19171175)                     ---YCTKLWCTGKAKGQMV-CQTRH--FPWADGTSCGE----GKLCLKGACVERHNLNKH   [512]
Fugu 15                                      ---PCSKLWCTGKAQGQLV-CQTRH--FPWADGTGCGD----GRVCHRGSCTAKNSTAQA   [463]
Homo ADAMTS9 (27463365)                      ---QCRRLWCNNVNGVHKG-CRTQH--TPWADGTECEP----GKHCKYGFCVPKEMDV--   [584]
Fugu 9                                       ---QCRRLWCTSPEGAQRG-CRTQH--MPWADGTDCSP----GKHCKHGLCITKESD---   [429]
Homo ADAMTS20 (28460690)                     ---ICMHLWCTSTEKLHKG-CFTQH--VPPADGTDCGP---GMVHCRHGLCVNKETE---   [538]
Mus 20 (29500513)                            ---HCRRLWCTSAEGVHKG-CRTQH--MPLADGTSCGP----GMHCHRGLCVTRDME---   [548]
Fugu 20                                      ---QCKRLWCTSAEGDHKG-CRTQH--MPLADGTDCGH----GMYCKHGMCVNKELD---   [519]
Ciona 9 (0100132719)                         ---TCQRLWCINQNRSH-G-CRTPH--VPWADGTSCGDGK-QKMWCQRGHCVPEDRHRV-   [634]
Apis 9                                       KSAVCRRLWCTTPNEDHYDHCRTQH--MPWADGTSCGRD----KWCYRGECVSRSLNLQ-   [445]
Drosophila CG6107 (20151419)                 ---ECKRLWCNRTSGNSNEQCASSN--LPWADGTPCGSS---GHWCQRGKCVSNKHGYG-   [747]
Caenorhabditis gon-1 CEt13h10 (3879882)      ---TCRRLWCATFYGSQMG-CRTQH--MPWADGTPCDESR--SMFCHHGACVRLAPESL-   [599]
introns                                      ---------------54--------------------------55---------------   [2]
```

```
[                                                                     970       980       990       1000      1010      1020]
[                                                                      .         .         .         .         .         .]
[Modal, ≥80%, full maj.                                             G W  W      CSR CG G      R C  P                      ]
mask                                                            ----1111111111111111111111111111111-------------------------   [329]
Homo ADAMTS6 (11131380)                                         SI--DGGWGPWSLWGECSRTCGGGVSSSLRHCDSP-------------------------   [588]
Fugu 6                                                          SL--NGNWGPWSMWGECSRTCGGGVSSSMRHCDSP-------------------------   [592]
Ciona 6 (0100146470)                                            SV--DGQWGEWTSWGECSRTCGTGVSSSHRSCDSP-------------------------   [606]
Homo ADAMTS10 (17432918)                                        GV--DGAWGPWTPWGDCSRTCGGGVSSSSRHCDSP-------------------------   [577]
Mus 10 (27502095)                                               GV--DGAWGPWTPWGDCSRSCGGGVSSSSRHCDSP-------------------------   [543]
Fugu 10                                                         GV--DGGWGLWSTWGECSRTCGGGVSSSIRHCDSP-------------------------   [530]
Homo ADAMTS16 (21265061)                                         PT--HGHWSDWSSWSPCSRTCGGGVSHRSRLCTNP-------------------------   [617]
Mus 16 (21961374)                                               PT--HGHWSDWSPWSPCSRTCGGGISHRDRLCTNP-------------------------   [614]
Fugu 16                                                         PQ--HGQWSDWSSWSACSRSCESGVTYRERHCNNP-------------------------   [583]
Ciona 16 (0100138085)                                           AI--DGGWGDYASWSPCTRTCGGGVQHRMRACNNP-------------------------   [473]
Homo ADAMTS18 (21265067)                                        PI--HGQWSAWSKWSECSRTCGGGVKFQERHCNNP-------------------------   [619]
Fugu 18                                                         AV--HGQWSAWSQWSDCSRTCGGGVMYRERSCTSP-------------------------   [366]
Homo ADAMTS12 (13569928)                                        SI--PGGWGRWSPWSHCSRTCGAGVQSAERLCNNP-------------------------   [572]
Mus 12 (27817773)                                               SI--PGGWGRWSPWSHCSRTCGAGAQSAERLCNNP-------------------------   [576]
Fugu 12                                                         TV--NGGWGQWTTWSHCSRTCGTGVQSAERECNNP-------------------------   [569]
Homo ADAMTS7 (11131377)                                          AV--DGGWSGWSAWSICSRSCGMGVQSAERQCTQP-------------------------   [568]
Fugu 7                                                          MI--NGGWGSWSEWSACSRTCGAGVQNAHRDCDNP-------------------------   [545]
Ciona 7 (genewise.34.12.1)                                      AF--DGAWSAWTTWSRCSRSCGVGITFSERRCDQP-------------------------   [533]
Homo ADAMTS17 (21265064)                                         HV--DGDWSPWGAWSMCSRTCGTGARFRQRKCDNP-------------------------   [573]
Homo ADAMTS19 (19525737)                                         ----EHLAGEWSLWSPCSRTCSAGISSRERKC--P-------------------------   [661]
Drosophila dCG4096 (22831757)                                   -AAVNGGWGDWSEWSECSRSCGGGVSTQQRECDNP-------------------------   [653]
Homo ADAMTS14 (29337086)                                         TYGQDGGWSSWTKFGSCSRSCGGGVRSRSRSCNNP-------------------------   [584]
Fugu 14                                                         GH--DGSWGSWSKFGSCSRTCGGGIRSRSRQCNNP-------------------------   [553]
Homo ADAMTS3 (2224673)                                           QQKQDGNWGSWTKFGSCSRTCGTGVRFRTRQCNNP-------------------------   [581]
Fugu 3                                                          QVKQDGAWGSWSKYGSCSRSCGTGIRFRTRQCNNP-------------------------   [344]
Ciona 3 (0100146117)                                            QTAVDGNWGSWNTYGQCSRSCGTGVWYRTRMCNNP-------------------------   [522]
Homo ADAMTS2 (7656867)                                           ILKRDGSWGAWSPFGSCSRTCGTGVKFRTRQCDNP-------------------------   [591]
Mus 2 (28204840)                                                 ILKRDGNWGAWTPFGSCSRTCGTGVKFRTRQCDNP-------------------------   [592]
Fugu 2                                                          IKKQDGNWGAWSKFGQCSRTCGGGVKFRTRECDSP-------------------------   [362]
Homo ADAMTS13 (16306598)                                        IAAVHGRWSSWGPRSPCSRSCGGGVVTRRRQCNNP-------------------------   [414]
Fugu 13                                                         SVVVHGAWSSWSGFSPCSRTCGGGISHRTRKCTNP-------------------------   [342]
Homo ADAMTS1 (6525075)                                           DTPFHGNWGMWGPWGDCSRTCGGGVQYTMRECDNP-------------------------   [589]
Mus 1 (1813340)                                                 ATPVHGSWGPWGPWGDCSRTCGGGVQYTMRECDNP-------------------------   [573]
Homo ADAMTS4 (12643637)                                          NIPQAGGWGPWGPWGDCSRTCGGGVQFSSRDCTRP-------------------------   [550]
Mus 4 (26350615)                                                NVPQAGGWGPWGPWGDCSRTCGGGVQFSSRDCTRP-------------------------   [558]
Fugu 4                                                          QIPVNGGWGTWGPWGDCSRTCGGGVQYSFRSCDNP-------------------------   [344]
Homo ADAMTS8 (21536392)                                          KPVVDGGWAPWGPWGECSRTCGGGVQFSHRECKDP-------------------------   [557]
Mus 8 (6708110)                                                 KAVVDGDWGPWRPWGQCSRTCGGGIQFSNRECDNP-------------------------   [572]
Fugu 8                                                          LVVVDGGWSLWGPWRRCSRTCGGGVEFSYRECTDP-------------------------   [474]
Homo ADAMTS5 (7768707)                                           STSSHGNWGSWGSWGQCSRSCGGGVQFAYRHCNNP-------------------------   [597]
Mus 5 (5923784)                                                 STSSHGNWGSWGPWGQCSRSCGGGVQFAYRHCNNP-------------------------   [597]
Fugu 5                                                          SASNHGSWSSWGPWGSCSRSCGGGVQFAQRLCNNP-------------------------   [549]
Ciona 15 (0100137065)                                           -RPVDGHWGRWGHFGACSRSCGGGVQMSSRQCNNP-------------------------   [543]
Homo ADAMTS15 (19171175)                                         RV--DGSWAKWDPYGPCSRTCGGGVQLARRQCTNP-------------------------   [546]
Fugu 15                                                         KV--NGRWGAWSPYAGCSRSCGGGVELAKRECNSP-------------------------   [497]
Homo ADAMTS9 (27463365)                                          -PVTDGSWGSWSPFGTCSRTCGGGIKTAIRECNRP-------------------------   [618]
Fugu 9                                                          ATPVDGAWGVWSPFGTCSRTCGGGIKIAMRECNRP-------------------------   [464]
Homo ADAMTS20 (28460690)                                         TRPVNGEWGPWEPYSSCSRTCGGGIESATRRCNRP-------------------------   [573]
Mus 20 (29500513)                                                TRPVDGEWGPWGPYSSCSRTCGGGIKSTARLCDRP-------------------------   [583]
Fugu 20                                                         LQPVHGEWGPWGPYSVCSRSCGGGTRSTSRDCNKP-------------------------   [554]
Ciona 9 (0100132719)                                            --PVDGNWGEWSRFGECSRRCGGGIKSATRKCDSPNNAANSGGFSNLLLKDLSFIEIRVR   [692]
Apis 9                                                          --PVDGQWGEWGRYGKCSRTCGGGVKKKYRECDNP-------------------------   [478]
Drosophila CG6107 (20151419)                                    -RQVNGGWGPWTPFTPCSLTCGGGVQESRRECNQP-------------------------   [781]
Caenorhabditis gon-1 CEt13h10 (3879882)                         -TKIDGQWGDWRSWGECSRTCGGGVQKGLRDCDSP-------------------------   [633]
introns                                                         56----------------57---------------58-----------------------   [2]
[                                                                      thrombospondin                                      ]
```

```
[                                                     1030      1040      1050      1060      1070      1080]
[                                                     .         .         .         .         .         .]
[Modal, ≥80%, full maj.                                          P   GG  C G        C    C          FR  QC ]
mask                                        ----------1---------11111111111111111111111-11-1111--1111111   [365]
Homo ADAMTS6 (11131380)                     ----------A---------PSGGGKYCLGERKRYRSCNTDPC-PL-GSRD--FREKQCA   [625]
Fugu 6                                      ----------A---------PSGGGKYCLGERKRYRSCNIDAC-PA-GSRN--FREKQCA   [629]
Ciona 6 (0100146470)                        ----------A---------PEHGGKYCLGGRKRYRSCNTQEC-PN-PKSD--FRQEQCA   [643]
Homo ADAMTS10 (17432918)                     ----------R---------PTIGGKYCLGERRRHRSCNTDDC-PP-GSQD--FREVQCS   [614]
Mus 10 (27502095)                           ----------R---------PTIGGKYCLGERRRHRSCNTNDC-PP-GSQD--FREMQCS   [580]
Fugu 10                                     ----------R---------PTIGGKYCLGERKRFRSCNIDDC-PA-GSRD--FREIQCS   [567]
Homo ADAMTS16 (21265061)                     ----------K---------PSHGGKFCEGSTRTLKLCNSQKC-PR-DSVD--FRAAQCA   [654]
Mus 16 (21961374)                           ----------R---------PSHGGKFCQGSTRTLKLCNSQRC-PL-DSVD--FRAAQCA   [651]
Fugu 16                                     ----------R---------PAYGGKFCEGSARSYKLCNTVDC-PP-NTTD--YRAQQCA   [620]
Ciona 16 (0100138085)                       ----------T---------PRYGGRHCEGEEKIYKLCNTEPC-AP-NQVS--FREQQCA   [510]
Homo ADAMTS18 (21265067)                     ----------K---------PQYGGLFCPGSSRIYQLCNINPC-NE-NSLD--FRAQQCA   [656]
Fugu 18                                     ----------R---------PQNNGKFCPGSSRFNQLCNTRPC-PP-NTVD--FRAQQCA   [403]
Homo ADAMTS12 (13569928)                     ----------E---------PKFGGKYCTGERKRYRLCNVHPC-RS-EAPT--FRQMQCS   [609]
Mus 12 (27817773)                           ----------E---------PKFGGKYCTGERKRYRLCNVHPC-RS-DTPT--FRQMQCS   [613]
Fugu 12                                     ----------K---------PEFGGKYCTGERKRYRTCNTKPC-QK-NKPT--FREMQCS   [606]
Homo ADAMTS7 (11131377)                      ----------T---------PKYKGRYCVGERKRFRLCNLQAC-PA-GRPS--FRHVQCS   [605]
Fugu 7                                      ----------V---------PKYRGTYCLGERRRYKICNITPC-PQ-EVPT--FRDVQCG   [582]
Ciona 7 (genewise.34.12.1)                  ----------E---------PRHGGRYCLGERKRYRTCNKWAC-EA-TNTTSTFREQQCA   [572]
Homo ADAMTS17 (21265064)                     ----------P---------PGPGGTHCPGASVEHAVCENLPC-PK-GLPS--FRDQQCQ   [610]
Homo ADAMTS19 (19525737)                     ----------G---------LDSEARDCNGPRKQYRICENPPC-PA-GLPG--FRDWQCQ   [698]
Drosophila dCG4096 (22831757)               ----------V---------PANGGVFCIGERKRYKICRKRPC-PA-EEPS--FRAQQCA   [690]
Homo ADAMTS14 (29337086)                     ----------S---------PAYGGRLCLGPMFEYQVCNSEEC-PG-TYED--FRAQQCA   [621]
Fugu 14                                     ----------P---------PAYGGRDCPGSSFDYQMCNTEEC-AG-PYED--FRAQQCV   [590]
Homo ADAMTS3 (2224673)                      ----------M---------PINGGQDCPGVNFEYQLCNTEEC-QK-HFED--FRAQQCQ   [618]
Fugu 3                                      ----------A---------PSNGGQDCPGVNFEYQLCNTDDC-PK-HFED--FRAQQCQ   [381]
Ciona 3 (0100146117)                        ----------M---------PSHGGVDCIGQNIDFDLCSKRPC-P--NGRD--FREEQCQ   [558]
Homo ADAMTS2 (7656867)                       ----------H---------PANGGRTCSGLAYDFQLCSRQDC-PD-SLAD--FREEQCR   [628]
Mus 2 (28204840)                            ----------H---------PANGGRTCSGLAYDFQLCNPQDC-PN-SLAD--FREEQCQ   [629]
Fugu 2                                      ----------R---------PLNGGRTCLGATYQFQMCNTNEC-ED-IYND--PREEQCR   [399]
Homo ADAMTS13 (16306598)                     ----------R---------PAFGGRACVGADLQAEMCNTQAC-EK-TQLE--FMSQQCA   [451]
Fugu 13                                     ----------R---------PAFGGSDCEGADVEAELCHQQRC-ENRTQLD--FIAEQCS   [380]
Homo ADAMTS1 (6525075)                       ----------V---------PKNGGKYCEGKRVRYRSCNLEDC-PDNNGKT--FREEQCE   [627]
Mus 1 (1813340)                             ----------V---------PKNGGKYCEGKRVRYRSCNIEDC-PDNNGKT--FREEQCE   [611]
Homo ADAMTS4 (12643637)                      ----------V---------PRNGGKYCEGRRTRFRSCNTEDC-PTGSALT--FREEQCA   [588]
Mus 4 (26350615)                            ----------V---------PRNGGKYCEGRRTRFRSCNTENC-PHGSALT--FREEQCA   [596]
Fugu 4                                      ----------L---------PRNGGKYCEGKRIQYHSCNTETC-PDTNGLT--FREEQCL   [382]
Homo ADAMTS8 (21536392)                      ----------E---------PQNGGRYCLGRRAKYQSCHTEEC-PP-DGKS--FREQQCE   [594]
Mus 8 (6708110)                             ----------M---------PQNGGRFCLGERVKYQSCNTEEC-PP-NGKS--FREQQCE   [609]
Fugu 8                                      ----------E---------PRNGGKYCEGQRVQYQSCNTQAC-PDHSAKS--FREEQCE   [512]
Homo ADAMTS5 (7768707)                       ----------A---------PRNNGRYCTGKRAIYRSCSLMPC-PP-NGKS--FRHEQCE   [634]
Mus 5 (5923784)                             ----------A---------PRNSGRYCTGKRAIYRSCSVTPC-PP-NGKS--FRHEQCE   [634]
Fugu 5                                      ----------P---------PRNNGRYCTGKRAIYRSCNVSPC-PP-STKS--FRAEQCE   [586]
Ciona 15 (0100137065)                       ----------E---------PRHGGKYCIGSRLKFSSCNTNPCQPVAGRPVRDYRSEQCA   [584]
Homo ADAMTS15 (19171175)                     ----------T---------PANGGKYCEGVRVKYRSCNLEPC-PS-SASGKSFREEQCE   [585]
Fugu 15                                     ----------T---------PENGGKYCYGLRVKYRSCNLNPC-PD-AGKS--FREQQCE   [534]
Homo ADAMTS9 (27463365)                      ----------E---------PKNGGKYCVGRRMKFKSCNTEPC-LK-QKRD--FRDEQCA   [655]
Fugu 9                                      ----------V---------PRNGGKYCVGRRMKFRSCNSEPC-SK-QKKD--FREEQCA   [501]
Homo ADAMTS20 (28460690)                     ----------E---------PRNGGNYCVGRRMKFRSCNTDSC-PK-GTQD--FREKQCS   [610]
Mus 20 (29500513)                           ----------E---------PRNGGRYCVGRRMKFRSCNTDSC-PK-GKRD--FREKQCS   [620]
Fugu 20                                     ----------E---------PRNGGRFCVGRRMKFRSCNTEPC-PR-GRKD--FREEQCS   [591]
Ciona 9 (0100132719)                        SQVNVHLQVFEIKRGIKPDMPRYGGRYCTGKRKKFRPCNFQEC-SV--DSD--FRAEQCA   [747]
Apis 9                                      ----------L---------PQNGGRYCIGEQVKYRSCGTREC-PP-GTPD--FRDQQCS   [515]
Drosophila CG6107 (20151419)                ----------V---------PENGGKYCTGSRKKYRSCNTHQC-PP-GSMD--PREQQCY   [818]
Caenorhabditis gon-1 CEt13h10 (3879882)     ----------K---------PRNGGKYCVGQRERYRSCNTQEC-PW-DTQP--YREVQCS   [670]
introns                                     -------------------59-------------------60---61,62---------   [2]
```

AC Nicholson, SB Malik, JM Logsdon Jr, E van Meir, 2004. Functional evolution of ADAMTS genes: evidence]
[from analyses of phylogeny and gene organization. Contact: john-logsdon@uiowa.edu]
[Intron phases marked 0, 1, 2]

[	1090 1100 1110 1120 1130 1140]	
[	. . . . . .]	
[Modal, ≥80%, full maj.	W P C L C	]
mask	11111----11-11111---11111111----------1111111111111--11----1	[401]
Homo ADAMTS6 (11131380)	DFDNM----PF-RGKYY---NWKPYTGG-----GV---KPCALNCLAEGYN--FY----T	[663]
Fugu 6	DFDNM----PF-RGKYY---NWKPYAGG-----GV---KPCALNCLAEGYN--FY----T	[667]
Ciona 6 (0100146470)	RFDTE----PF-QGRYL---NWIAFDGG-----YQV--KPCALNCKPEGYS--FY----A	[682]
Homo ADAMTS10 (17432918)	EFDSI----PF-RGKFY---KWKTYRGG-----GV---KACSLTSLAEGFN--FY----T	[652]
Mus 10 (27502095)	EFDSV----PF-RGKFY---TWKTYRGG-----GV---KACSLTCLAEGFN--FY----T	[618]
Fugu 10	DFDGV----PF-RGKFY---TWKPYRGG-----GV---KPCSLNCLAEGYN--FY----T	[605]
Homo ADAMTS16 (21265061)	EHNSR----RF-RGRHY---KWKPYTQVED---QD----LCKLYCIAEGFD--FF----F	[693]
Mus 16 (21961374)	EYNSK----RF-RGWLY---KWKPYTQLED---QD----LCKLYCIAEGFD--FF----F	[690]
Fugu 16	EFNSR----QF-RGWHY---TWRPYTKVDD---QD----ICKLYCFAEGYD--FF----F	[659]
Ciona 16 (0100138085)	QYNRR----AY-RRQYF---HWTPFTKYSS---FD----QCELLCKAEGYD--FF----D	[549]
Homo ADAMTS18 (21265067)	EYNSK----PF-RGWFY---QWKPYTKVEE---ED----RCKLYCKAENFE--FF----F	[695]
Fugu 18	EYNSK----LF-RGWYY---KWKPYTKVDD---ED----ICKLYCIAEDFD--FF----F	[442]
Homo ADAMTS12 (13569928)	EFDTV----PY-KNELY---HWFPIFNP-----AH----PCELYCRPIDGQ--FS----E	[646]
Mus 12 (27817773)	EFDTV----PY-KNQFY---RWFPVFNA-----AH----PCELYCRPIDEQ--FS----E	[650]
Fugu 12	EFDTV----PY-NNELY---QWIPVSNPRKYSKIKSMVHPCELHCRPSTEY--FS----E	[652]
Homo ADAMTS7 (11131377)	HFDAM----LY-KGQLH---TWVPVVND-----VN----PCELHCRPANEY--FA----K	[642]
Fugu 7	HFNVL----PY-KGTFY---KWETVFNKA----S-----PCELHCRPLNEN--FS----E	[619]
Ciona 7 (genewise.34.12.1)	DFNNV----LY-KNKTY---TWLPVNSAV----Y-----PCELHCYTRAGKTPFA----D	[611]
Homo ADAMTS17 (21265064)	AHDR-----LS-PKKKG---LLTAVVVDD----K-----PCELYCSPLGKE--SP----L	[646]
Homo ADAMTS19 (19525737)	AYSVR---TSS-PKHIL---QWQAVLDEE----K-----PCALFCSPVGKE--QP----I	[736]
Drosophila dCG4096 (22831757)	RFDNV----SY-QGATYK---WLPFFDKN----N-----PCKLFCSDVDDT--II----A	[727]
Homo ADAMTS14 (29337086)	KRNSY---YVH-QNAKH---SWVPYEPDDD---AQ----KCELICQSADTG--DV----V	[661]
Fugu 14	QRSNK---Y-H-NNIKH---TWIPYEHPDE---AR----KCELSCRSKETG--EV----V	[629]
Homo ADAMTS3 (2224673)	QRNSH---FEY-QNTKH---HWLPYEHPDP---KK----RCHLYCQSKETG--DV----A	[658]
Fugu 3	LRNSH---FEF-QNTKH---HWLPHEHPDA---NK----RCHLYCQSKETG--DV----T	[421]
Ciona 3 (0100146117)	YQKE----VLY-KGQKH---KWFPYEPRDA---SL----KCELYCVSKRTH--DI----V	[597]
Homo ADAMTS2 (7656867)	QWDLY---FEH-GDAQH---HWLPHEHRDA---KE----RCHLYCESRETG--EV----V	[668]
Mus 2 (28204840)	QWDLY---FEH-GDVQH---HWLPHEHRDA---KE----RCHLYCESKETG--EV----V	[669]
Fugu 2	TWEPE---FEL-HSNKH---HWLPYEHPDP---NK----RCNLHCQSKETR--SV----A	[439]
Homo ADAMTS13 (16306598)	RTDGQP--LRS-SPGGASFYHWGAAVPHSQG--DA----LCRHMCRAIGES--FI----M	[496]
Fugu 13	RTDSQA--LYL-LPNAASFYTWIPAVGFTQG--EE----QCQYMCQSDGEN--FL----V	[425]
Homo ADAMTS1 (6525075)	AHNEF---SKA-SFGSGPAVEWIPKYAGV----SPKD--RCKLICQAKGIG--YF----F	[671]
Mus 1 (1813340)	AHNEF---SKA-SFGNEPTVEWTPKYAGV----SPKD--RCKLTCEAKGIG--YF----F	[655]
Homo ADAMTS4 (12643637)	AYNHR---TDL-FKSFPGPMDWVPRYTGV----APQD--QCKLTCQARALG--YY----Y	[632]
Mus 4 (26350615)	AYNHR---TDL-FKSFPGPMDWVPRYTGV----APRD--QCKLTCQARALG--YY----Y	[640]
Fugu 4	AHNDMS--AQV-SLGSGEGVEWVPKYAGI----SPKD--RCKLVCRAKGTG--YF----F	[427]
Homo ADAMTS8 (21536392)	KYNAY---NYT-DMDGN-LLQWVPKYAGV----SPRD--RCKLFCRARGRS--EF----K	[637]
Mus 8 (6708110)	KYNAY---NHT-DLDGN-FLQWVPKYSGV----SPRD--RCKLFCRARGRS--EF----K	[652]
Fugu 8	KYNSP----NY-LDHNGHMKEWIPKYAGV----SPRD--RCKLFCRARGSS--EF----K	[555]
Homo ADAMTS5 (7768707)	AKNGY---QSD-AKGVKTFVEWVPKYAGV----LPAD--VCKLTCRAKGTG--YY----V	[678]
Mus 5 (5923784)	AKNGY---QSD-AKGVKTFVEWVPKYAGV----LPAD--VCKLTCRAKGTG--YY----V	[678]
Fugu 5	VRNGP---QTD-PKGVKTFVEWVPKYAGV----LPKD--VCKLTCRAKGTG--YY----V	[630]
Ciona 15 (0100137065)	TYDGQ---RFP-LLGVQTASTWMPKYTDI----HTRD--RCKLTCQSVQTG--AY----I	[628]
Homo ADAMTS15 (19171175)	AFNGY---NHS-TNRLTLAVAWVPKYSGV----SPRD--KCKLICRANGTG--YF----Y	[629]
Fugu 15	EFNGM---NLN-TNRLGSSVVWVPKYSGI--- SHKD--QCKLICRANGTG--YF----Y	[578]
Homo ADAMTS9 (27463365)	HFDGK---HFN-INGLLPNVRWVPKYSGI----LMKD--RCKLFCRVAGNT--AY----Y	[699]
Fugu 9	AFDGR---HFN-INGLPPNVRWVPKYSGI----LMKD--RCKLFCRVAGST--AY----Y	[545]
Homo ADAMTS20 (28460690)	DFNGK---HLD-ISGIPSNVRWLPRYSGI----GTKD--RCKLYCQVAGTN--YF----Y	[654]
Mus 20 (29500513)	DFDGK---HFD-INGLPPNVRWLPKYSGI----AVKD--RCKLYCRVAGTT--SF----Y	[664]
Fugu 20	QFDGK---HFN-INGLPPSVRWIPKYIGI----LMKD--RCKLFCRVAGTT--AY----Y	[635]
Ciona 9 (0100132719)	AYNNWARHHRHRIRGIAPNAEWIPKYDGI----LLQD--RCKLICQVKSDD--QYAVPWY	[799]
Apis 9	YFNNNNL---N-IKNLSRDVKWHAK---I----LPHE--RCKLYCETDSNQ--CY----P	[556]
Drosophila CG6107 (20151419)	AMNGR---NMN-IPGVNPDTKWVPKYEK-----D-----ACKLFCRMDMKV--TY----F	[858]
Caenorhabditis gon-1 CEt13h10 (3879882)	EFNNKD--IGI-QGVASTNTHWVPKYANV----APNE--RCKLYCRLSGSA--AF----Y	[715]
**introns**	**----------------------------------63,64----------------------**	**[2]**

AC Nicholson, SB Malik, JM Logsdon Jr, E van Meir, 2004. Functional evolution of ADAMTS genes: evidence]
[from analyses of phylogeny and gene organization. Contact: john-logsdon@uiowa.edu]
[Intron phases marked 0, 1, 2]

```
[                                                                 1150      1160      1170      1180      1190      1200]
[                                                                    .         .         .         .         .         .]
[Modal, ≥80%, full maj.                                       V DGT C              C  G C   GCD    S    D CGVC G     C ]
mask                                        1111111111111-------111111111111111111111111111111111111111111111   [454]
Homo ADAMTS6 (11131380)                     ERAPAVIDGTQCNA------DSLDICINGECKHVGCDNILGSDAREDRCRVCGGDGSTCD   [717]
Fugu 6                                      ERSPAVVDGTRCQA------DSLDICINGECKHVGCDNILGSDAKEDRCRVCGGDGSTCE   [721]
Ciona 6 (0100146470)                        ERAGAVIDGTRCFS------NSLDMCINGECHHVGCDHVLHSDAVEDKCRVCGGDGSTCR   [736]
Homo ADAMTS10 (17432918)                    ERAAAVVDGTPCRP------DTVDICVSGECKHVGCDRVLGSDLREDKCRVCGGDGSACE   [706]
Mus 10 (27502095)                           ERAAAVVDGTPCRP------DTVDICVSGECKHVGCDRVLGSDLREDKCRVCGGDGSACE   [672]
Fugu 10                                     ERAPAVVDGTVCRD------DSVDVCVNGECKHVGCDRVLGSDVREDRCRTCGGDGSSCV   [659]
Homo ADAMTS16 (21265061)                    SLSNKVKDGTPCSE------DSRNVCIDGICERVGCDNVLGSDAVEDVCGVCNGNNSACT   [747]
Mus 16 (21961374)                           SLSNKVKDGTPCSE------DSRNVCIDGMCERVGCDNVLGSDATEDSCGVCKGNNSDCV   [744]
Fugu 16                                     ALASKVKDGTLCSQ------DSSNVCIDGLCERVGCDRILGSAAAPDACGICRGDNSTCK   [713]
Ciona 16 (0100138085)                       MLADKVIDGTPCDA------SSTDVCIQGKCKHVGCDFVLGSNATFDSCGVCNGDNSTCQ   [603]
Homo ADAMTS18 (21265067)                    AMSGKVKDGTPCSP------NKNDVCIDGVCELVGCDHELGSKAVSDACGVCKGDNSTCK   [749]
Fugu 18                                     AMSSKVKDGTPCSK------TETNVCIEGVCEEVGCDQILGSQASLDACGICKGDNSTCK   [496]
Homo ADAMTS12 (13569928)                    KMLDAVIDGTPCFEGG----NSRNVCINGICKMVGCDYEIDSNATEDRCGVCLGDGSSCQ   [702]
Mus 12 (27817773)                           RMLEAVIDGTPCFEGG----NSRNVCINGICKRVGCDYEIDSNATEDRCGVCLGDGSACQ   [706]
Fugu 12                                     KMLDAVSDGTPCFVNN----NSRSICVNGVCKDVGCDYGIDSNAVEDRCGVCLGDGKSCE   [708]
Homo ADAMTS7 (11131377)                     KLRDAVVDGTPCYQVR----ASRDLCINGICKNVGCDFEIDSGAMEDRCGVCHGNGSTCH   [698]
Fugu 7                                      KLRDAVTDGTLCYEGN----KSRDMCINGICMKVGCDYVVESSAVEDRCGVCNGDGSTCT   [675]
Ciona 7 (genewise.34.12.1)                  RLRDKVIDGTKCYN------RGKDICVDGICKSLGCDLMIDSPAQEDRCGVCRGDGTSCE   [665]
Homo ADAMTS17 (21265064)                    LVADRVLDGTPCGP------YETDLCVHGKCQKIGCDGIIGSAAKEDRCGVCSGDGKTCH   [700]
Homo ADAMTS19 (19525737)                    LLSEKVMDGTSCGY------QGLDICANGRCQKVGCDGLLGSLAREDHCGVCNGNGKSCK   [790]
Drosophila dCG4096 (22831757)               NWGATVLDGTPCTL------GTNNMCIDGICKKVGCDWIVDSEVQDDRCGVCGGSGDQCQ   [781]
Homo ADAMTS14 (29337086)                    FMNQVVHDGTRCSYR-----DPYSVCARGECVPVGCDKEVGSMKADDKCGVCGGDNSHCR   [716]
Fugu 14                                     FMNQVMHDGTRCSYS-----DPFSVCARGECLDVGCDKEVGSYKQEDKCGVCEGDNSHCR   [684]
Homo ADAMTS3 (2224673)                      YMKQLVHDGTHCSYK-----DPYSICVRGECVKVGCDKEIGSNKVEDKCGVCGGDNSHCR   [713]
Fugu 3                                      YMKQLVHDGTRCSYK-----DAYSICVRGECVKVGCDREIGSTKVEDKCGVCGGDNSHCR   [476]
Ciona 3 (0100146117)                        SMGDIVGDGTRCSYE-----DPHGICVRGECVDIGCDKVIGSSMREDVCGVCGGNGQHCR   [652]
Homo ADAMTS2 (7656867)                      SMKRMVHDGTRCSYK-----DAFSLCVRGDCRKVGCDGVIGSSKQEDKCGVCGGDNSHCK   [723]
Mus 2 (28204840)                            SMKRMVHDGTRCSYK-----DAFSLCVRGDCRKVGCDGVIGSRKQEDKCGVCGGDNTHCK   [724]
Fugu 2                                      STDQVVLDGTRCSYK-----DPHSVCVRGECEKVGCDGLFGSSKQEDKCGVCGGDNSSCK   [494]
Homo ADAMTS13 (16306598)                    KRGDSFLDGTRCMPSGPREDGTLSLCVSGSCRTFGCDGRMDSQQVWDRCQVCGGDNSTCS   [556]
Fugu 13                                     RRGSQFLDGTRCESDASPPFGSTAACLRGICQLFGCDGTLHSGKVKDVCGVCGGNASSCS   [485]
Homo ADAMTS1 (6525075)                      VLQPKVVDGTPCSP------DSTSVCVQGQCVKAGCDRIIDSKKKFDKCGVCGGNGSTCK   [725]
Mus 1 (1813340)                             VLQPKVVDGTPCSP------DSTSVCVQGQCVKAGCDRIIDSKKKFDKCGVCGGNGSTCK   [709]
Homo ADAMTS4 (12643637)                      VLEPRVVDGTPCSP------DSSSVCVQGRCIHAGCDRIIGSKKKFDKCMVCGGDGSGCS   [686]
Mus 4 (26350615)                            VLEPRVADGTPCSP------DTSSVCVQGRCIHAGCDRIIGSKKKFDKCMVCGGDGSRCS   [694]
Fugu 4                                      VLKSKVADGTPCSP------DSTTVCVQGQCVKAGCDRVIGSNRRFDKCGVCGGDGSTCK   [481]
Homo ADAMTS8 (21536392)                      VFEAKVIDGTLCGP------ETLAICVRGQCVKAGCDHVVDSPRKLDKCGVCGGKGNSCR   [691]
Mus 8 (6708110)                             VFEAKVIDGTLCGP------DTLSICVRGQCVKAGCDHVVNSPKKLDKCGVCGGKGTACR   [706]
Fugu 8                                      VFESKVIDGTTCGP------DTTSVCVQGQCVKAGCDKVIGSNIRVDKCGECGGSGMSCR   [609]
Homo ADAMTS5 (7768707)                      VFSPKVTDGTECRP------YSNSVCVRGKCVRTGCDGIIGSKLQYDKCGVCGGDNSSCT   [732]
Mus 5 (5923784)                             VFSPKVTDGTECRP------YSNSVCVRGRCVRTGCDGIIGSKLQYDKCGVCGGDNSSCT   [732]
Fugu 5                                      VFSQRVTDGTECRP------YSSSVCVKGKCVRTGCDGIIGSKLQYDKCGVCGGDSTGCI   [684]
Ciona 15 (0100137065)                       TLADRVVDGTRCGP------DTTNVCVHGQCMPAGCDGILGSKANFSKCGVCGGKNSDCK   [682]
Homo ADAMTS15 (19171175)                    VLAPKVVDGTLCSP------DSTSVCVQGKCIKAGCDGNLGSKKRFDKCGVCGGDNKSCK   [683]
Fugu 15                                     VLAPKVVDGTPCSP------DTPSVCVQGKCIKAGCEGKLNSRKKFDKCGVCGGNNQGCT   [632]
Homo ADAMTS9 (27463365)                     QLRDRVIDGTPCGQ------DTNDICVQGLCRQAGCDHVLNSKARRDKCGVCGGDNSSCK   [753]
Fugu 9                                      QLRDRVIDGTPCGP------DTNDICVQGLCRQAGCDHVLNSKARRDKCGVCGGDNSSCK   [599]
Homo ADAMTS20 (28460690)                    LLKDMVEDGTPCGT------ETHDICVQGQCMAAGCDHVLNSSAKIDKCGVCGGDNSSCK   [708]
Mus 20 (29500513)                           QLKDRVADGTPCGT------ETNDICVQGLCRQAGCDHVLNSKAKRDKCGVCGGDNSSCQ   [718]
Fugu 20                                     QLKDRVIDGTPCGT------DTYDICVQGLCRQAGCDHVLNSKARNDKCGVCGGDNSSCK   [689]
Ciona 9 (0100132719)                        VLKPKVINGTPCGP------DTNDICVEGVCKMAGCDHVLNSRTTVDECGICNGDNSACR   [853]
Apis 9                                      -LAEKVIDGTPCDQ------DTFHICVNGFCKPAGCDHVLNSTAELDICGVCKGDNSTCQ   [609]
Drosophila CG6107 (20151419)                MLKSMVTDGTSCAV------DSFDKCVNGICRPAGCDNELNSIAKLDKCGVCEGRNDTCH   [912]
Caenorhabditis gon-1 CEt13h10 (3879882)     LLRDKVVDGTPCDR------NGDDICVAGACMPAGCDHQLHSTLRRDKCGVCGGDDSSCK   [769]
introns                                     ----65-----66------------------67--68---------69------------   [2]
```


AC Nicholson, SB Malik, JM Logsdon Jr, E van Meir, 2004. Functional evolution of ADAMTS genes: evidence]
[from analyses of phylogeny and gene organization. Contact: john-logsdon@uiowa.edu]
[Intron phases marked 0, 1, 2]

```
[                                                       1210      1220      1230      1240      1250      1260]
[                                                          .         .         .         .         .         .]
[Modal, ≥80%, full maj.                           G                   Y         IP G                  A     ]
mask                                           1111111111-----------11111111111111111111111111-----111111--   [496]
Homo ADAMTS6 (11131380)                        AIEGFFNDSLPR---------GGYMEVVQIPRGSVHIEVREVAMSKN-----YIALKSE-   [762]
Fugu 6                                         TTEGLFNQSLPR---------GGYMEVVQIPKGSVHIEIKELAMSKN-----YIALKSE-   [766]
Ciona 6 (0100146470)                           TEAGVFNLPVTR---------TQYQEVITIPKGSVNIFIQELEISVQN----YIALRNT-   [782]
Homo ADAMTS10 (17432918)                       TIEGVFSPASPG---------AGYEDVVWIPKGSVHIFIQDLNLSLS-----HLALKGD-   [751]
Mus 10 (27502095)                              TIEGVFSPALPG---------TGYEDVVWIPKGSVHIFIQDLNLSLS-----HLALKGD-   [717]
Fugu 10                                        SVEGLFNSSLPE---------GGYEEVVRIPKGSVFIHIQELNISLN-----YLVLKNK-   [704]
Homo ADAMTS16 (21265061)                       IHRGLYTKHHHT---------NQYYHMVTIPSGARSIRIYEMNVSTS-----YISVRNA-   [792]
Mus 16 (21961374)                              THRGLYSKHHST---------NQYYHMVTIPSGARSIHIYETNISTS-----YISVRNS-   [789]
Fugu 16                                        IYKGQYTKQHYT---------NQYYGVVTIPAGARGIRVMELNTSSS-----YLAVRDT-   [758]
Ciona 16 (0100138085)                          SFNGSYDDNVRD---------SGYYDVIRIPRGARSVKIWENKCCTH---S-YIAVRSAS   [650]
Homo ADAMTS18 (21265067)                       FYKGLYLNQHKA---------NEYYPVVLIPAGARSIEIQELQVSSS-----YLAVRSL-   [794]
Fugu 18                                        FFKGQYTLQHRA---------NEYYTMVVIPAGARSIHVQEMAVSTS-----YLAVRSI-   [541]
Homo ADAMTS12 (13569928)                       TVRKMFKQKE-G---------SGYVDIGLIPKGARDIRVMEIEGAGN-----FLAIRSED   [747]
Mus 12 (27817773)                              TVKKLFRQKE-G---------SGYVDIGLIPKGARDIRVMEIKAAGN-----FLAIRSED   [751]
Fugu 12                                        MVYKSFDEEE-G---------LGYTDVGVIPVGARDIVVKEVEEAGN-----FLALKSES   [753]
Homo ADAMTS7 (11131377)                        TVSGTFEEAE-G---------LGYVDVGLIPAGAREIRIQEVAEAAN-----FLALRSED   [743]
Fugu 7                                         TVRRTFEESE-G---------LGYVDIGLIPEGARDIRIEEVAEAGN-----FLALRSDN   [720]
Ciona 7 (genewise.34.12.1)                     PVDNIFDVKR-G---------EQYVQVGILPKGARNIEVSEVLPSRN-----FLALKGPG   [710]
Homo ADAMTS17 (21265064)                       LVKGDFSHAR-G---------TGYIEAAVIPAGARRIRVVEDKPAHS-----FLALKDS-   [744]
Homo ADAMTS19 (19525737)                       IIKGDFNHTR-G---------AGYVEVLVIPAGARRIKVVEEKPAHS-----YLALRDA-   [834]
Drosophila dCG4096 (22831757)                 PVRETYTDPFAA-------KDGAYVEIVTIPARARHILIRELANSPH-----FLAIATGD   [829]
Homo ADAMTS14 (29337086)                       TVKGTLGKASKQ---------AGALKLVQIPAGARHIQIEALEKSPH-----RIVVKNQV   [762]
Fugu 14                                        TVKLTLTKTPKK---------NGMFKMFDIPMGARHIVIEENETSPH-----VIAVKNQV   [730]
Homo ADAMTS3 (2224673)                          TVKGTFTRTPRK---------LGYLKMFDIPPGARHVLIQEDEASPH-----ILAIKNQA   [759]
Fugu 3                                         TVKGSFTRTPKK---------PGYLKMFYIPPGARHVVIQEHEASPQ-----ILAIKNQA   [522]
Ciona 3 (0100146117)                           NINGVLNKRANRKV-------NNYMRLYKIPAGARRINIRETSSSSSF---NLLALKSRQ   [702]
Homo ADAMTS2 (7656867)                          VVKGTFTRSPKK---------HGYIKMFEIPAGARHLLIQEVDATSH-----HLAVKNLE   [769]
Mus 2 (28204840)                               VVKGTFTRSPRK---------QDYIKMFEIPAGARHLLIQEADTTSH-----HLSVKNLE   [770]
Fugu 2                                         TFKDTITRTSKK---------QGFLKVLEIPRGARHLLIQELKATSH-----TLAVKNVA   [540]
Homo ADAMTS13 (16306598)                       PRKGSFTAGRA----------REYVTFLTVTPNLTSVYIANHRPLFT-----HLAVRI--   [599]
Fugu 13                                        LRSGSYTAGQA----------REYTTFLSLPVNATQVHVINRAPLFT-----HMAVVL--   [528]
Homo ADAMTS1 (6525075)                          KISGSVTSAK-----------PGYHDIITIPTGATNIEVKQRNQRGSRNNGSFLAIKAA-   [773]
Mus 1 (1813340)                                KMSGIVTSTR-----------PGYHDIVTIPAGATNIEVKHRNQRGSRNNGSFLAIRAA-   [757]
Homo ADAMTS4 (12643637)                         KQSGSFRKFR-----------YGYNNVVTIPAGATHILVRQQGNPGHRS--IYLALKLP-   [732]
Mus 4 (26350615)                               KQSGSFKKFR-----------YGYSDVVTIPAGATHILVRQQGGSGLKS--IYLALKLS-   [740]
Fugu 4                                         KVSGSLDRAR-----------PGYQDVVTIPAGATHVDVKQRATGNGRHDNSYLAVRRQ-   [529]
Homo ADAMTS8 (21536392)                         KVSGSLTPTN-----------YGYNDIVTIPAGATNIDVKQRSHPGVQNDGNYLALKTA-   [739]
Mus 8 (6708110)                                KISGSFTPFS-----------YGYNDIVTIPAGATNIDVKQRSHPGVRNDGSYLALKTA-   [754]
Fugu 8                                         KITGLYNKEIS----------YGYSDIITIPVGATNIDIKQRSHRGLKHDGNYLAIKRE-   [658]
Homo ADAMTS5 (7768707)                          KIVGTFNKKS-----------KGYTDVVRIPEGATHIKVRQFKAKDQTRFTAYLALKKK-   [780]
Mus 5 (5923784)                                KIIGTFNKKS-----------KGYTDVVRIPEGATHIKVRQFKAKDQTRFPAYLALKKK-   [780]
Fugu 5                                         RVVGNFTKKS-----------KGYTDVVKIPAGSTHLKVRQHKAKDQTRYSAYLALRRP-   [732]
Ciona 15 (0100137065)                          RIRGKYNKRV-----------AGYNNVITLPVGAASIKIEQRGFHNKVYDGNYLALKKP-   [730]
Homo ADAMTS15 (19171175)                       KVTGLFTKPM-----------HGYNFVVAIPAGASSIDIRQRGYKGLIGDDNYLALKNS-   [731]
Fugu 15                                        KVSGMFTKPI-----------HGYNFVVTLPVGAANIDVRQRGYQGMMSDENYLAVRNR-   [680]
Homo ADAMTS9 (27463365)                         TVAGTFNTVH-----------YGYNTVVRIPAGATNIDVRQHSFSGETDDDNYLALSSS-   [801]
Fugu 9                                         TVAGTFNIVH-----------YGYNEVVRIPSGATNIDVRQHSYSGRSDDDNYLAISNS-   [647]
Homo ADAMTS20 (28460690)                       TITGVFNSSH-----------YGYNVVVKIPAGATNVDIRQYSYSGQPDDS-YLALSDA-   [755]
Mus 20 (29500513)                              TLAGVFNSAH-----------YGYNVVVKIPAGATNIEILQHSYSGRPEDDNYLALSDT-   [766]
Fugu 20                                        TMAGTFNDAQ-----------YGYNTVVRIPAGATNIDIKQVSYSGKPEDDNYLALSDS-   [737]
Ciona 9 (0100132719)                           TVQGSYNTGYN----------IGYNRVVIIPAGANNIVIMQRSYSGDADDFNYLALRDS-   [902]
Apis 9                                         WITGSYNSTQ-----------YGYTRIAKIPAGSNNIDIRQPGWKDMIDDGNYLALRLGE   [658]
Drosophila CG6107 (20151419)                   EVTGNLLVSNLLGLNDGNEPNKTLYYVTRIPKGASNIIITQRGYPDQ----NFIVLTDD-   [967]
Caenorhabditis gon-1 CEt13h10 (3879882)        VVKGTFNEQGT----------FGYNEVMKIPAGSANIDIRQKGYNNMKEDDNYLSLRAA-   [818]
introns                                        ---70--------------71,72---------73--------------------74---   [2]
[                                                             matrix metalloprotease cleavage site              ]
```

```
[                                                     1270      1280      1290      1300      1310      1320]
[                                                      .         .         .         .         .         .]
[Modal, ≥80%, full maj.                             NG                    G      Y       E               ]
mask                                        --111111111-11111----1---11111---1111111-1111111-----1111111   [537]
Homo ADAMTS6 (11131380)                     -GDDYYINGAWTIDWPR----K---FDVAG--TAFHYKRPTDEPESLE-----ALGPTSE   [807]
Fugu 6                                      -GEEYYINGAWTIDWPR----K---FDIGG--TTFHYKRPSDEPESLE-----AMGPTTE   [811]
Ciona 6 (0100146470)                        -RGDEYINGGWAIDWPR----K---FDIAG--TTFSYKRPAGEPESLQ-----ALGPTTE   [827]
Homo ADAMTS10 (17432918)                    -QESLLLEGLPGTPQPH----R---LPLAG--TTFQLRQGPDQVQSLE-----ALGPINA   [796]
Mus 10 (27502095)                           -QESLLLEGLPGTPQPH----R---LPLAG--TTFHLRQGPDQAQSLE-----ALGPINA   [762]
Fugu 10                                     -GDQYFINGNLSIDTPR----R---FNVAG--TTFHYRRPIDGPETLE-----ALGPTNM   [749]
Homo ADAMTS16 (21265061)                    -LRRYYLNGHWTVDWPG----R---YKFSG--TTFDYRRSYNEPENLI-----ATGPTNE   [837]
Mus 16 (21961374)                           -LKRYYLNGHWSVDWPG----R---YKFSG--ATFNYKRSYKEPENLT-----SPGPTNE   [834]
Fugu 16                                     -QRRYYLNGHWTVDWPG----R---HAIAG--AIFEYKRPYNRPESLI-----SSGPTNE   [803]
Ciona 16 (0100138085)                       NSDRYYLNGNWEIDLYG----E---TTFAG--AKWFYNRKSFQSETLL-----TPGPLGE   [696]
Homo ADAMTS18 (21265067)                    -SQKYYLTGGWSIDWPG----E---FPFAG--TTFEYQRSFNRPERLY-----APGPTNE   [839]
Fugu 18                                     -KKKYYLTGDWTVDWPG----K---FHIGG--TMFDYQRSFNKPESLY-----AAGPTNE   [586]
Homo ADAMTS12 (13569928)                    -PEKYYLNGGFIIQWNG----N---YKLAG--TVFQYDRK-GDLEKLM-----ATGPTNE   [791]
Mus 12 (27817773)                           -PEKYYLNGGFIIQWNG----N---YKLAG--TVFQYDRK-GDLEKLI-----APGPTNE   [795]
Fugu 12                                     -SEEYFLNGNYIIQWNG----E---YVAGG--TTFYYERS-GNMENLT-----APGPIKQ   [797]
Homo ADAMTS7 (11131377)                     -PEKYFLNGGWTIQWNG----D---YQVAG--TTFTYARR-GNWENLT-----SPGPTKE   [787]
Fugu 7                                      -PNKYFLNGGWTIQWNG----D---YKAAG--TIFTYERT-RHLENLT-----SPGPTTE   [764]
Ciona 7 (genewise.34.12.1)                  DSDKFYLNGGYMIQWNS----N---FELAG--STFSYERVNTTQDNIT-----AQGPITH   [756]
Homo ADAMTS17 (21265064)                    --GKGSINSDWKIELPG----E---FQIAG--TTVRYVRR-GLWEKIS-----AKGPTKL   [787]
Homo ADAMTS19 (19525737)                    --GKQSINSDWKIEHSG----A---FNLAG--TTVHYVRR-GLWEKIS-----AKGPTTA   [877]
Drosophila dCG4096 (22831757)               GGDRFYLNGDSLISMPG----E---FEIAG--AESLYDRV-DEQETIT-----IPQPIQH   [874]
Homo ADAMTS14 (29337086)                    -TGSFILNPKG-KEATS----R--TFTAMG--LEWEDAVE-DAKESLK-----TSGPLPE   [806]
Fugu 14                                     -TGNFILNSKS-EDAET----K--TFIENG--LLWEYSND-GDKETLK-----TSGPLHE   [774]
Homo ADAMTS3 (2224673)                      -TGHYILNGKG-EEAKS----R--TFIDLG--VEWDYNIE-DDIESLH-----TDGPLHD   [803]
Fugu 3                                      -TGHYILNGKG-EELKS----R--SFIDLG--VEWHYIMD-RDVETLH-----TDGPLHD   [566]
Ciona 3 (0100146117)                        -GRRYTLNSRSYRIQTR-DTPH--KFVSAG--TLWHYDVI-NGLESIT-----AYGPTNS   [750]
Homo ADAMTS2 (7656867)                      -TGKFILNEENDVDASS----K--TFIAMG--VEWEYRDE-DGRETLQ-----TMGPLHG   [814]
Mus 2 (28204840)                            -TGKFILNEENHLDPNS----R--SFIAMG--VEWEYRNE-DERETLQ-----TIGPLHG   [815]
Fugu 2                                      -SGLFFVNGEN-EYLES----R--SMIEKG--VEWEYEYD-DGKETLQ-----TTGPLRH   [584]
Homo ADAMTS13 (16306598)                    -GGRYVVAGKMSISPNT----TYPSLLEDG---RVEYRVALTEDRLPRLEEIRIWGPLQE   [651]
Fugu 13                                     -RSQYIVAGRGAMSLNV----TYPSVLDED---RLEYRLYLTPDLLPEMEELLLPGPLQE   [580]
Homo ADAMTS1 (6525075)                      -DGTYILNGDYTLSTLE----Q--DIMYKG--VVLRYSGSSAALERIR-----SFSPLKE   [819]
Mus 1 (1813340)                             -DGTYILNGNFTLSTLE----Q--DLTYKG--TVLRYSGSSAALERIR-----SFSPLKE   [803]
Homo ADAMTS4 (12643637)                     -DGSYALNGEYTLMPSP----T--DVVLPGA-VSLRYSGATAASETLS-----GHGPLAQ   [779]
Mus 4 (26350615)                            -DGSYALNGEYTLMPSP----T--DVVLPGA-VSLRYSGATAASETLS-----GHGPLAQ   [787]
Fugu 4                                      -DGTYLLNGDYKLMTME----T--DVALRG--ALLRYSGSSATLERIR-----SFSPLPE   [575]
Homo ADAMTS8 (21536392)                     -DGQYLLNGNLAISAIE----Q--DILVKG--TILKYSGSIATLERLQ-----SFRPLPE   [785]
Mus 8 (6708110)                             -NGQYLLNGNLAISAIE----Q--DILVKG--TILKYSGSMATLERLQ-----SFQALPE   [800]
Fugu 8                                      -GGSYILNGNYSISTVE----Q--DIPVLG--AVLKYSGSSTTLERIQ-----SFRQLKE   [704]
Homo ADAMTS5 (7768707)                      -NGEYLINGKYMISTSE----T--IIDING--TVMNYSGWSHRDDFLHGM---GYSATKE   [828]
Mus 5 (5923784)                             -TGEYLINGKYMISTSE----T--IIDING--TVMNYSGWSHRDDFLHGM---GYSATKE   [828]
Fugu 5                                      -NGDYLLNGKFMISTSE----T--IIPLNG--SVLNYSGWSQRDEWLHSM---GPGALQE   [780]
Ciona 15 (0100137065)                       -NGQYILNGALILSTIK----R--DIEVNG--TVLQYSGVDVPNERIH-----ATMPLKE   [776]
Homo ADAMTS15 (19171175)                    -QGKYLLNGHFVVSAVE----R--DLVVKG--SLLRYSGTGTAVESLQ-----ASRPILE   [777]
Fugu 15                                     -HGEYILNGNYVVSAAE----R--DLLVRG--SLVRYSGTSSSVETLQ-----AVMPLKE   [726]
Homo ADAMTS9 (27463365)                     -KGEFLLNGNFVVTMAK----R--EIRIGN--AVVEYSGSETAVERIN-----STDRIEQ   [847]
Fugu 9                                      -RGEFLFNGEFVVSMFK----R--EINVGN--AVIEYSGSDHVIERIN-----CTDRIEE   [693]
Homo ADAMTS20 (28460690)                    -EGNFLFNGNFLLSTSK----K--EINVQGTRTVIEYSGSNNAVERIN-----STNRQEK   [803]
Mus 20 (29500513)                           -QGNFLLNGNFVVSMAK----K--EINIQG--AVFEYSGSNNSIERIN-----STDRLEA   [812]
Fugu 20                                     -QSNFVINGNLVVAMFK----K--EINFKG--TIIEYSGSGTEVERIN-----CTERIEE   [783]
Ciona 9 (0100132719)                        -RGNYILNGNRSVMKNKHKIHKRNNKNNRGPLYQCDYTGTDTAIEKIN-----CSKAIEI   [956]
Apis 9                                      -GGKYIVNGNFKV-MTQ----RV-IVDIGV---TIEYSGRDSRVERLN-----TSRPIET   [703]
Drosophila CG6107 (20151419)                -RDNELLNGKFLKTYPL----K---FVYAG--VTMQYTGSSSVVEQVN---TTYSWKLSR   [1014]
Caenorhabditis gon-1 CEt13h10 (3879882)     -NGEFLLNGHFQVSLAR----Q--QIAFQD--TVLEYSGSDAIIERIN-----GTGPIRS   [864]
introns                                     -75----------76-----------------77--------------------------   [2]
```

AC Nicholson, SB Malik, JM Logsdon Jr, E van Meir, 2004. Functional evolution of ADAMTS genes: evidence]
[from analyses of phylogeny and gene organization. Contact: john-logsdon@uiowa.edu]
[Intron phases marked 0, 1, 2]

```
[                                                                 1330      1340      1350      1360      1370      1380]
[                                                                    .         .         .         .         .         .]
[Modal, ≥80%, full maj.                                                         Y                                      ]
mask                                          111111111-------111111111111111111-11-----------------------   [566]
Homo ADAMTS6 (11131380)                       NLIVMVLLQ-------EQNLGIRYKFNVPITRTGSGDNEVGFT-----------------   [843]
Fugu 6                                        NLFVMVLLQ-------EQNQGIRYKFNVPIQRTGSGDNEVGFS-----------------   [847]
Ciona 6 (0100146470)                          DLVVMMLLQ-------EPNRGLRYEYNVRVEREGSAGAESAFL-----------------   [863]
Homo ADAMTS10 (17432918)                      SLIVMVLAR-------TELPALRYRFNAPIARDSLPPYS---------------------   [828]
Mus 10 (27502095)                             SLIIMVLAQ-------AELPALHYRFNAPIARDALPPYS---------------------   [794]
Fugu 10                                       SLIIMVLVR-------EENPGVHYRFNPPLNRDPLTGFA---------------------   [781]
Homo ADAMTS16 (21265061)                      TLIVELLFQ-------GRNPGVAWEYSMPRLGTEKQPPAQPSYT----------------   [874]
Mus 16 (21961374)                             TLIVELLFQ-------GRNPGVAWEFSLPRSGAKKTPAAQPSYS----------------   [871]
Fugu 16                                       TLVIEVLLQ-------GWNPGVRWEYTLMKADEKRKHNYT--------------------   [836]
Ciona 16 (0100138085)                         ELLIQVLVV-------AQNPGISYSYTTQVIDNRLPGLRTNSAIQVQPPSYN--------   [741]
Homo ADAMTS18 (21265067)                      TLVFEILMQ-------GKNPGIAWKYALPKVMNGTPPATKRPAYT---------------   [877]
Fugu 18                                       TLVFEILLQ-------GKNPGVVWEYTLPRTEKKPDYS----------------------   [617]
Homo ADAMTS12 (13569928)                      SVWIQLLFQ-------VTNPGIKYEYTIQKDGLDNDVE-QMYF-----------------   [826]
Mus 12 (27817773)                             SVWLQLLFQ-------VTNPGIKYEYTVRKDGLDNDVEKLLYF-----------------   [831]
Fugu 12                                       PVMLQLLFQ-------ETNPGLKYEFSVRKTKVTGNEVMQPIYR----------------   [834]
Homo ADAMTS7 (11131377)                       PVWIQLLFQ-------ESNPGVHYEYTIHREAGGHDEVPPPVFS----------------   [824]
Fugu 7                                        PLWIQLLFQ-------EPNPGVRYEYTINRNTSGNNSLPTSSFS----------------   [801]
Ciona 7 (genewise.34.12.1)                    DIMIMILFV-------EENPGIRVSYMMPFNVTAVFEEERPKFE----------------   [793]
Homo ADAMTS17 (21265064)                      PLHLMVLLFH------DQDYGIHYEYTVPVNRTAENQSEPEKPQDSLFI-----------   [830]
Homo ADAMTS19 (19525737)                      PLHLLVLLFQ------DQNYGLHYEYTIPSDPLPENQSSKAPEPLFM-------------   [918]
Drosophila dCG4096 (22831757)                 SISLYAIVRGN-----ESNAGIFYEFTLPALNVTAGRQFQ--------------------   [909]
Homo ADAMTS14 (29337086)                      AIAILALPPTEG----GPRSSLAYKYVIHEDLLPLIGSNNVLLEEMDTYE----------   [852]
Fugu 14                                       GIVVLVIPQEE-----DTKISLTYKYIIHEDLLPLITNNNVLLAELDTYE----------   [819]
Homo ADAMTS3 (2224673)                        PVIVLIIPQEN-----DTRSSLTYKYIIHEDSVPTINSNNVIQEELDTFE----------   [848]
Fugu 3                                        PVVVLIIPKDN-----ETRTTLMYKYIIHEDSVPVNN-NNVIQE--DTYE----------   [608]
Ciona 3 (0100146117)                          DVILLISVQRR-----ASRVSVAYSYVMHVNSLPRSHE--------TFR-----------   [786]
Homo ADAMTS2 (7656867)                         TITVLVIPVG------DTRVSLTYKYMIHEDSLN-VDDNNVLEEDSVVYE----------   [857]
Mus 2 (28204840)                              TITVLVIPEG------DTRISLTYKYMIHEDSLN-VDDNNVLEDDAVRHE----------   [858]
Fugu 2                                        GILIMMKLHG------DEDVNLSYKYMMNMDSDTAIQ-NNMLVEDSS-YE----------   [626]
Homo ADAMTS13 (16306598)                      DADIQVYRRYGEEYGNLTRPDITFTYFQPKPRQAWV------------------------   [687]
Fugu 13                                       EINVEVYRKYGKEYGEQTNPNISFQFYVPGPPKDVTNTKLKGK-----------------   [623]
Homo ADAMTS1 (6525075)                        PLTIQVLTVGN-----ALRPKIKYTYFVKKKKESFNAIPTFSA-----------------   [857]
Mus 1 (1813340)                               PLTIQVLMVGH-----ALRPKIKFTYFMKKKTESFNAIPTFSE-----------------   [841]
Homo ADAMTS4 (12643637)                       PLTLQVLVAGN-----PQDTRLRYSFFVPRPTPSTPRPTPQDWLHRRAQILEILRRRP--   [832]
Mus 4 (26350615)                              PLTLQVLVAGN-----PQNARLRYSFFVPRPVPSTPRPPPQDWLQRRAEILKILRKRP--   [840]
Fugu 4                                        PLTIQVLSVGE-----APRPRVKYSYFAPRPNNGASTSNNGGRRPSINAIREVG------   [624]
Homo ADAMTS8 (21536392)                       PLTVQLLTVPGE----VFPPKVKYTFFVPNDVDFSMQS-SKERATTNIIQPLLH-AQ---   [836]
Mus 8 (6708110)                               PLTVQLLTVSGE----VFPPKVRYTFFVPNDMDFSVQN-SKERATTNIIQSLPSAE----   [851]
Fugu 8                                        AITIQLLTTAGD----VIPAKVKYTFFIPRDVTFSKSKEQKAPSPSLHVIHPFG------   [754]
Homo ADAMTS5 (7768707)                        ILIVQILATDP-----TKPLDVRYSFFVPKKSTPKVNS-VTSHGSNKVGSHTSQPQ----   [878]
Mus 5 (5923784)                               ILIVQILATDP-----TKALGVRYSFFVPKKTTQKVNS-VISHGSNKVGPHSTQLQ----   [878]
Fugu 5                                        ALVVQILATDA-----KKPLDIRYSFFMPRRTAPQQPS-VP-------------------   [815]
Ciona 15 (0100137065)                         PITVQLLSVGVAGSK-LLPPRVKYSYYVSAPKRRASRKSKAKVPSSRSAVSDGSLVNRGI   [835]
Homo ADAMTS15 (19171175)                      PLTVEVLSVGK-----MTPPRVRYSFYLPKEPREDKSSHPKDPRGPSVLHNSVLSLSNQV   [832]
Fugu 15                                       PLTVEVLSVGK-----MTPPRVRYTFYVSKESKEEKVLRKEERSHNRILEGGNKVEPKMG   [781]
Homo ADAMTS9 (27463365)                       ELLLQVLSVGK-----LYNPDVRYSFNIPIEDKPQQFY----------------------   [880]
Fugu 9                                        EIIIQVLSVGK-----LYNPDVRYSFNIPIEDKPQQFF----------------------   [726]
Homo ADAMTS20 (28460690)                      ELILQVLCVGN-----LYNPDVHYSFNIPLEERSDMFT----------------------   [836]
Mus 20 (29500513)                             ELVLQVLCVGN-----LYNPDVRYSFNIPIEERSNLFS----------------------   [845]
Fugu 20                                       ELILQVLCVGS-----LYNPDVRYSFNIPIEEHKEQFV----------------------   [816]
Ciona 9 (0100132719)                          DIIVEVLSAGD-----LMPPNINYRYNIPVRDRLSYR-----------------------   [988]
Apis 9                                        DLILEVLSVSE-----LNPPQISYKYTVPRKILESYE-----------------------   [735]
Drosophila CG6107 (20151419)                  DLIVQIISLDVSPSKRQDTVLLSYSYTIDKPPDYEAEVEIYR------------------   [1056]
Caenorhabditis gon-1 CEt13h10 (3879882)       DIYVHVLSVG------SHPPDISYEYMTAAVPNAVIRPISSALYL---------------   [903]
introns                                       ----78-------------------------------------79---------------   [2]
```

AC Nicholson, SB Malik, JM Logsdon Jr, E van Meir, 2004. Functional evolution of ADAMTS genes: evidence]
[from analyses of phylogeny and gene organization. Contact: john-logsdon@uiowa.edu]
[Intron phases marked 0, 1, 2]

```
[                                                                     1390      1400      1410      1420      1430      1440]
[                                                                        .         .         .         .         .          .]
[Modal, ≥80%, full maj.                                                            W            C    C  G       C            ]
mask                                                      -------------------11111------------------------------------   [571]
Homo ADAMTS6 (11131380)                                   -------------------WNHQP-W---SECSAT-CAGGVQRQEVVCKRLDDNSI--VQ   [877]
Fugu 6                                                    -------------------WNHLP-W---SECSTT-CSGGSQKQEVVCKRLDDNSV--VQ   [881]
Ciona 6 (0100146470)                                      -------------------WSHGD-W---SPCTAS-CAGGTQRRPIECRRVDDDTV--VQ   [897]
Homo ADAMTS10 (17432918)                                  -------------------WHYAP-W---TKCSAQ-CAGGSQVQAVECRNQLDSSA--VA   [862]
Mus 10 (27502095)                                         -------------------WHYAP-W---TKCSAQ-CAGGSQVQVVECRNQLDSSA--VA   [828]
Fugu 10                                                   -------------------WHYTS-W---SRCSAL-CAGGVQTQQVVCMKHSDRTI--VY   [815]
Homo ADAMTS16 (21265061)                                   -------------------WAIVR-----SECSVS-CGGGQMTVREGCYRDLKFQV--NM   [907]
Mus 16 (21961374)                                         -------------------WAIVR-----SECSVS-CGGDRQCQTGRGHLEISS------   [900]
Fugu 16                                                   -------------------WAIVR-----SQCSAT-CAAGLMNTKTACYKDMRVQV--NT   [869]
Ciona 16 (0100138085)                                     -------------------WSSTS-----DSCSAT-CAGGTQTVHISCVMDGTTVV--DE   [774]
Homo ADAMTS18 (21265067)                                   -------------------WSIVQ-----SECSVS-CGGGYINVKAICLRDQNTQV--NS   [910]
Fugu 18                                                   -------------------WGVVR-----SDCSAP-CAGGRISTKAICLQDQKVQV--NS   [650]
Homo ADAMTS12 (13569928)                                   -------------------WQYGH-W---TECSVT-CGTGIRRQTAHCIKKGRGMV--KA   [860]
Mus 12 (27817773)                                         -------------------WQFGR-W---TECSVT-CGTGIRRQAAHCVKKGHGIV--KT   [865]
Fugu 12                                                   -------------------WRHGA-W---TDCSTS-CGLGEQLQPVRCFEMDVGVV--DE   [868]
Homo ADAMTS7 (11131377)                                    -------------------WHYGP-W---TKCTVT-CGRGVQRQNVYCLERQAGPV--DE   [858]
Fugu 7                                                    -------------------WKFGS-W---TECSAT-CAGVQRQIVHCVERTTGIV--EE   [835]
Ciona 7 (genewise.34.12.1)                                -------------------WMMQK-W---SSCSRT-CGRGIQLSLAICVERKAGFV--EE   [827]
Homo ADAMTS17 (21265064)                                   -------------------WTHSG-W---EGCSVQ-CGGGERRTIVSCTRIVNKTT--TL   [864]
Homo ADAMTS19 (19525737)                                   -------------------WTHTS-W---EDCDAT-CGGGERKTTVSCTKIMSKNI--SI   [952]
Drosophila dCG4096 (22831757)                             -------------------WRLSN-W---TACSAS-CGGGVQHREPICQENGKGES--NE   [943]
Homo ADAMTS14 (29337086)                                   -------------------WALKS-W---APCSKA-CGGGIQFTKYGCRRRRDHHM--VQ   [886]
Fugu 14                                                   -------------------WALKS-W---SQCSKP-CGGGIQYTKYGCRRKSDSRL--VH   [853]
Homo ADAMTS3 (2224673)                                     -------------------WALKS-W---SQVSKP-CGGGFQYTKYGCRRKSDNKM--VH   [882]
Fugu 3                                                    -------------------WALKS-W---SPCSKP-CAGGFQYTKYGCRKKGDTKM--LH   [642]
Ciona 3 (0100146117)                                      -------------------YAVKR-W---TVCSVT-CGAGTKRARYGCRSSRNGGR--WV   [820]
Homo ADAMTS2 (7656867)                                     -------------------WALKK-W---SPCSKP-CGGGSQFTKYGCRRRLDHKM--VH   [891]
Mus 2 (28204840)                                          -------------------WALKK-W---SPCSKP-CGGGSQFTKYGCRRRLDSKM--VH   [892]
Fugu 2                                                    -------------------WAPKK-W---SFCSRP-CGGGKQYLRYGCRRKVDSKM--VH   [660]
Homo ADAMTS13 (16306598)                                   -------------------WAAVR-----GPCSVS-CGAGLRWVNYSCLDQARKEL--VE   [720]
Fugu 13                                                   -------------------WTFVT-----TPCSVS-CGSGVQTHVHVCIDENTKKH--TE   [656]
Homo ADAMTS1 (6525075)                                     -------------------WVIEE-W---GECSKS-CELGWQRRLVECRDINGQPA--SE   [891]
Mus 1 (1813340)                                           -------------------WVIEE-W---GECSKT-CGSGWQRRVVQCRDINGHPA--SE   [875]
Homo ADAMTS4 (12643637)                                    -------------------WAGRK------------------------------------   [837]
Mus 4 (26350615)                                          -------------------WAGRK------------------------------------   [845]
Fugu 4                                                    ----------------GAEWTLRE-W---GPCSQT-CGGGVQQREVVCLDAYGHPS--RD   [661]
Homo ADAMTS8 (21536392)                                    -------------------WVLGD-W---SECSST-CGAGWQRRTVECRDPSGQAS--AT   [870]
Mus 8 (6708110)                                           -------------------WVLGD-W---SECPST-CRGSWQRRTVECRDPSGQAS--DT   [885]
Fugu 8                                                    ----------------VPDWVLGE-W---SECSKS-CGSGWSRRNVECKDSAGFSS--SH   [791]
Homo ADAMTS5 (7768707)                                     -------------------WVTGP-W---LACSRT-CDTGWHTRTVQCQDGNRKLA--KG   [912]
Mus 5 (5923784)                                           -------------------WVTGP-W---LACSRT-CDTGWHTRTVQCQDGNRKLA--KG   [912]
Fugu 5                                                    -------------------------L---LPYPKP-TTAHSTTTLSTMVRTTSSRT--TS   [844]
Ciona 15 (0100137065)                                      GRRIKSPSATPGSNHGPPRWVTGR-WRKCKAYGKRKCGSGWQKRSVTCKQGKRRAR----   [890]
Homo ADAMTS15 (19171175)                                   EQP---------DDRPPARWVAGS-W---GPCSAS-CGSGLQKRAVDCRGSAGQRT--VP   [876]
Fugu 15                                                   -------------KRPVSHWVAGG-W---ESCTVT-CGSGLQRRPVLCQSADKRAA--MD   [821]
Homo ADAMTS9 (27463365)                                     -------------------WNSHGPW---QACSKP-CQ-GERKRKLVCTRESDQLT--VS   [914]
Fugu 9                                                    -------------------WDAYGPW---QECSSL-CQ-GVRKRKIVCSRESDRVV--VS   [760]
Homo ADAMTS20 (28460690)                                   -------------------WDPYGPW---EGCTKM-CQ-GLQRRNITCIHKSDHSV--VS   [870]
Mus 20 (29500513)                                          -------------------WDPYGPW---QDCTKM-CQ-GLHRRKIACVRKSDHAV--VS   [879]
Fugu 20                                                   -------------------WDPSGSW---LECSRI-CQ-GERRRKVVCVRKSDHLE--VS   [850]
Ciona 9 (0100132719)                                       -------------------WDPHGPW---SPCSRL-CD-GKTKGKIMCVR-SDQQI--VS   [1021]
Apis 9                                                    -------------------WKLSG-W---SDCTRT-CQ-GIKYRKAECTNIEYKEV--VA   [768]
Drosophila CG6107 (20151419)                              -------------------WEMQA-P---SNCDSL-CE-GRSHRLPACISTTQGVK--VA   [1089]
Caenorhabditis gon-1 CEt13h10 (3879882)                   -------------------WRVTDTW---TECDRA-CR-GQQSQKLMCLDMSTHRQ--SH   [937]
introns                                                   ----------------------------------------80,81,82-------------   [2]
```


AC Nicholson, SB Malik, JM Logsdon Jr, E van Meir, 2004. Functional evolution of ADAMTS genes: evidence]
[from analyses of phylogeny and gene organization. Contact: john-logsdon@uiowa.edu]
[Intron phases marked 0, 1, 2]

```
[                                                               1450      1460      1470      1480      1490      1500]
[                                                                  .         .         .         .         .         .]
[Modal, ≥80%, full maj.                                       C             C    C    W       C   C                 ]
mask                                            ------------------------------------------------------------  [571]
Homo ADAMTS6 (11131380)                         NNY--CDPDSK-PPENQRACNTEPCPPE-WFIGDWLECSKTCDGGMRT-RAVLCIRK---  [929]
Fugu 6                                          NNY--CDPDGK-PPENHRDCNTEPCPPE-WFIGDWSECGKTCDGGIRT-RSVLCIRK---  [933]
Ciona 6 (0100146470)                            HSY--CNSDTM-PSEQVQTCNAEPCPAR-WITGSWGQCSRSCDGGHHV-RTVVCMRA---  [949]
Homo ADAMTS10 (17432918)                        PHY--CSAHSK-LPKRQRACNTEPCPPD-WVVGNWSLCSRSCDAGVRS-RSVVCQRR---  [914]
Mus 10 (27502095)                               PHY--CSGHSK-LPKRQRACNTEPCPPD-WVVGNWSRCSRSCDAGVRS-RSVVCQRR---  [880]
Fugu 10                                         NHF--CDKKNR-PKEKRRPCNTEPCSPS-WGTGEWSECSRSCNGGLRT-RQVLCKRR---  [867]
Homo ADAMTS16 (21265061)                        SF---CNPKTR-PVTGLVPCKVSACPPS-WSVGNWSACSRTCGGGAQS-RPVQC-TR---  [957]
Mus 16 (21961374)                               ------------------------------------------------------------  [900]
Fugu 16                                         SY---CNPRSR-PVTGLVPCNTQPCPAS-WSVGEWGACSQSCGGGEHT-RLVQCIQR---  [920]
Ciona 16 (0100138085)                           SH---CDRPSR-PSL-TKECNTQACPAR-WHTTEWGQCSRTCGGGLQL-RQVTCKSK---  [824]
Homo ADAMTS18 (21265067)                        SF---CSAKTK-PVTEPKICNAFSCPAY-WMPGEWSTCSKACAGGQQS-RKIQCVQK---  [961]
Fugu 18                                         TM---CNPYTK-PILGYHLCNTQPCPAY-WAPGDWGLCSRSCGGGQQT-RPLRCLRK---  [701]
Homo ADAMTS12 (13569928)                        TF---CDPETQ-PNGRQKKCHEKACPPR-WWAGEWEACSATCGPHGEKKRTVLCIQT---  [912]
Mus 12 (27817773)                               TF---CNPETQ-PSVRQKKCHEKDCPPR-WWAGEWEACSTTCGPYGEKKRTVLCIQT---  [917]
Fugu 12                                         SM---CDPESR-PDDRHRKCKTMDCPAR-WWVGGWQQCSATCGSDGTRKRTVLCVRT---  [920]
Homo ADAMTS7 (11131377)                          EH---CDPLGR-PDDQQRKCSEQPCPAR-WWAGEWQLCSSSCGPGGLSRRAVLCIRS---  [910]
Fugu 7                                          HW---CDPLTR-PDDNQTSCQKDPCPAV-WWIGEWQKCSASCGGLGQTKRTVLCIQA---  [887]
Ciona 7 (genewise.34.12.1)                      DF---CDPNTR-PDDNRRFCNTSACPPE-WLTGPWQSCSVTCGGQRSGRQSRNVLCI---  [879]
Homo ADAMTS17 (21265064)                        VNDSDCPQASR-PEPQVRRCNLHPCQSR-WVAGPWSPCSATCEKGF-QHREVTCVYQ---  [918]
Homo ADAMTS19 (19525737)                        VDNEKCKYLTK-PEPQIRKCNEQPCQTR-WMMTEWTPCSRTCGKGM-QSRQVACTQQ---  [1006]
Drosophila dCG4096 (22831757)                   PFQ--RIVSIK-RNLLIALGDTLPC----WTHAKN-KR-PARQSRG--------------  [980]
Homo ADAMTS14 (29337086)                        RHL--CDHKKR-PKPIRRRCNQHPCSQPVWVTEEWGACSRSCGKLGVQTRGIQCLLP---  [940]
Fugu 14                                         RNF--CETGKK-PKPIRKRCNVQDCSPPTWVAEEWGPCSKTCGKLGYQTRVVQCMQA---  [907]
Homo ADAMTS3 (2224673)                          RSF--CEANKK-PKPIRRMCNIQECTHPLWVAEEWEHCTKTCGSSGYQLRTVRCLQP---  [936]
Fugu 3                                          KGY--CDASKK-PKPIRRMCNLQDCTQPQWITDEWENCPRSCGSLGFQIRIVRCVQF---  [696]
Ciona 3 (0100146117)                            SKRN-CNGRP--PRPEKKICSTTCTVTTRWFATPWSACSKTCGNNGFHTRAVTCRQI---  [874]
Homo ADAMTS2 (7656867)                           RGF--CAALSK-PKAIRRACNPQECSQPVWVTGEWEPCSQTCGRTGMQVRSVRCIQP---  [945]
Mus 2 (28204840)                                RAF--CSALAK-PKAIRRACNPQECSQPVWVTGEWEPCTQSCGRTGMQVRSVRCIQP---  [946]
Fugu 2                                          KSF--CNKSNMKPRGDVRDCNQKSCPPPIWVTGDWQNCSKACGKTGLQIRTVSCVQP---  [715]
Homo ADAMTS13 (16306598)                         TVQ--CQGSQQ-PPAWPEACVLEPCPPY-WAVGDFGPCSASCGGGLRE-RPVRCVEA---  [772]
Fugu 13                                         EDN--CGTPPS-ITTPPTPCQLPPCPPR-WDTGPFGPCSASCGGGERK-RLLKCVQV---  [708]
Homo ADAMTS1 (6525075)                          -----CAKEVK-PASTRP-CADHPCPQ--WQLGEWSSCSKTCGKGYKKRSLK-CLSH---  [938]
Mus 1 (1813340)                                 -----CAKEVK-PASTRP-CADLPCPH--WQVGDWSPCSKTCGKGYKKRTLK-CVSH---  [922]
Homo ADAMTS4 (12643637)                          ------------------------------------------------------------  [837]
Mus 4 (26350615)                                 ------------------------------------------------------------  [845]
Fugu 4                                          -----CPEELR--PLASRSCATQPCPT--WLLGDWSVCSKTCG-----------------  [695]
Homo ADAMTS8 (21536392)                          -----CNKALK-PEDAKP-CESQLCPL---------------------------------  [890]
Mus 8 (6708110)                                 -----CDEALK-PEDAKP-CGSQPCPL---------------------------------  [905]
Fugu 8                                          -----CDRGLK-PTDIKA-CGDLPCPI--WQMGPWSACSRTCGHGERHRSVF-CIDY---  [838]
Homo ADAMTS5 (7768707)                           -----CPLSQR-PSAFKQ-CLLKKC-----------------------------------  [930]
Mus 5 (5923784)                                 -----CLLSQR-PSAFKQ-CLLKKC-----------------------------------  [930]
Fugu 5                                          SSS---PTFLASEPPIGLVMSTSSPGP-QWVTGSWMTCSRTCDTGWQSRTVQ-CKNL---  [896]
Ciona 15 (0100137065)                            G----CTGS---KPFDRLPCFINACPTVSTNGRRNHSGRSRPSPT----------ER---  [930]
Homo ADAMTS15 (19171175)                         A----CDAAHR-PVETQA-CGEP-CPT--WELSAWSPCSKSCGRGFQRRSLK-CVGH---  [923]
Fugu 15                                         -----CDSAER-PQSERA-CGDP-CPM--WTIGTWSHCSKSCGRGFKRRQVR-CETG---  [867]
Homo ADAMTS9 (27463365)                          DQR--CDRLPQ-PGHITEPCGTD-CDLR-WHVASRSECSAQCGLGYRTLDIY-CAKY---  [965]
Fugu 9                                          DQR--CHGIPR-PSVIADRCNTE-CELR-WHVARKSECTAQCGLGYRTLEIY-CAKI---  [811]
Homo ADAMTS20 (28460690)                         DKE--CDHLPL-PSFVTQSCNTD-CELR-WHVIGKSECSSQCGQGYRTLDIH-CMKY---  [921]
Mus 20 (29500513)                               DHN--CGHLPM-PLFVTEKCNMD-CELR-WHIIGKSDCSSQCGQGYRTLDVH-CMKY---  [930]
Fugu 20                                         DRR--CERLPQ-VVAVSEPCNMD-CEVR-WHVAGKSECSAKCGSGYRSLDVQ-CVKY---  [901]
Ciona 9 (0100132719)                            DRK--CNHLPK-PSTIIRPCNTR-CQFT-WHPKP-SECSVRCGRGTTHLEFE-CRKVT--  [1072]
Apis 9                                          DDY--CRESEK-PREESQMCNNH-CVLE-WNITV-SECSNHCGLGTRVVTSR-CIQKL--  [819]
Drosophila CG6107 (20151419)                    PQF--CDKSAM-PKIDDRACNTD-CRLN-LTVTSISECSAACGELGTREKTYACVQT---  [1141]
Caenorhabditis gon-1 CEt13h10 (3879882)         DRN--CQNVLK-PKQATRMCNID-CSTR-WITEDVSSCSAKCGSGQKRQRVS-CVKM---  [988]
introns                                         -----83---------------------84------------------------------  [2]
```

AC Nicholson, SB Malik, JM Logsdon Jr, E van Meir, 2004. Functional evolution of ADAMTS genes: evidence]
[from analyses of phylogeny and gene organization. Contact: john-logsdon@uiowa.edu]
[Intron phases marked 0, 1, 2]

```
[                                                           1510      1520      1530      1540      1550      1560]
[                                                            .         .         .         .         .         .]
[Modal, ≥80%, full maj.                                                                  C                      ]
mask                                        ------------------------------------------------------------   [571]
Homo ADAMTS6 (11131380)                     --I-GPSEEETL--DYSG-CLTHRPVEKEP---CNNQS---CPPQ-WVALDWSECTPKCG   [976]
Fugu 6                                      --I-SAAVEETL--EDVH-CLTHRPIEQES---CNNQS---CPPK-WVTLDWSECTPKCG   [980]
Ciona 6 (0100146470)                        --I-TQTEDEVV--DDRY-CTDRKPWSTGS---CGTEA---CPAR-WHTGDWSRCNPSCG   [996]
Homo ADAMTS10 (17432918)                    --V-SAAEEKAL--DDSA-CPQPRPPVLEA---CHGPT---CPPE-WAALDWSECTPSCG   [961]
Mus 10 (27502095)                           --V-SAAEEKAL--DDSA-CPQPRPPVLEA---CQGPM---CPPE-WATLDWSECTPSCG   [927]
Fugu 10                                     --I-SVTEEKVL--DDSA-CSPLRPSLTEP---CNNHS---CPPE-WLALDWSECTPSCG   [914]
Homo ADAMTS16 (21265061)                     --R-VHYDSEPV--PASL-CPQPAPSSRQA---CNSQS---CPPA-WSAGPWAECSHTCG   [1004]
Mus 16 (21961374)                           ------------------------------------------------------------   [900]
Fugu 16                                     --T-SQTDAHNL--SDAQ-CVQPAPARRQA---CNTHS---CPPV-WSTGPWSQCSRKCG   [967]
Ciona 16 (0100138085)                       ----RMNGDVYV--GESH-CNNKRKPPRTQQ--CMQTE---CPAY-WHTGEWSKCSRACG   [871]
Homo ADAMTS18 (21265067)                    --K-PFQKEEAV--LHSL-CPVSTPTQVQA---CNSHA---CPPQ-WSLGPWSQCSKTCG   [1008]
Fugu 18                                     --V-TYQREEVV--AHSL-CPIISPAQVQP---CQTQT---CPPE-WSTGSWSQ------   [742]
Homo ADAMTS12 (13569928)                     --M-VSDEQ-AL--PPTD-CQHLLKPKTLLS--CNRDIL--CPSD-WTVGNWSECSVSCG   [960]
Mus 12 (27817773)                           --M-GSDEQ-AL--PATD-CQHLLKPKALVS--CNRDIL--CPSD-WTVGNWSECSVSCG   [965]
Fugu 12                                     --V-LGEER-VL--HPVE-CKHLLKPKPLVP--CNRDVA--CGQD-WAVGVWGQCPVTCG   [968]
Homo ADAMTS7 (11131377)                     --V-GLDEQSAL--EPPA-CEHLPRPPTETP--CNRHVP--CPAT-WAVGNWSQCSVTCG   [959]
Fugu 7                                      --V-SAEEQKAL--PSRE-CEHVPKPASLSS--CNTHIP--CPAD-WTAGNWSK------   [930]
Ciona 7 (genewise.34.12.1)                  -QSISDDEQVVL--DSSR-CVASAKPIPIRK--CPPDAQQPCPV--WRTGPWSNCSVNCG   [931]
Homo ADAMTS17 (21265064)                     --L-QNGTHVAT--RPLY-CPGPRPAAVQS---CEGQD---CLSI-WEASEWSQCSASCG   [965]
Homo ADAMTS19 (19525737)                     --L-SNGTLIRA--RERD-CIGPKPASAQR---CEGQD---CMTV-WEAGVWSECSVKCG   [1053]
Drosophila dCG4096 (22831757)               ---------------------------------CGDQP---CPAH-WWPGPWQFCPVTCR   [1003]
Homo ADAMTS14 (29337086)                     --L-SNGTHKVM--PAKA-CAGDRPEARRP---CLRVP---CPAQ-WRLGAWSQCSATCG   [987]
Fugu 14                                     --Q-HNGTNKPV--HSKH-CTEDRPDTRRS---CNHTL---CPAQ-WRTGAWSQCSVTCG   [954]
Homo ADAMTS3 (2224673)                      --L-LDGTNRSV--HSKY-CMGDRPESRRP---CNRVP---CPAQ-WKTGPWSECSVTCG   [983]
Fugu 3                                      --L-HDGTNRSV--HSKY-CSGEKPESRRP---CNRVP---CPAQ-WRTGAWSECSVTCG   [743]
Ciona 3 (0100146117)                        --NPDATTQLVS---AAM-CFGQVKLPTQKT--CTVSP---CPPK-WRTGAWTKCSKTCG   [922]
Homo ADAMTS2 (7656867)                       --L-HDNTTRSV--HAKH-CNDARPESRRA---CSREL---CPGR-WRAGPWSQCSVTCG   [992]
Mus 2 (28204840)                            --L-HNNTTRSV--HTKH-CNDHRPESRRA---CNREL---CPGR-WRAGSWSQCSVTCG   [993]
Fugu 2                                      --S-EDNTTRPI--HNKH-CNDNRPESRRA---CNRQP---CPTQ-WRVGPWSQCSVTCG   [762]
Homo ADAMTS13 (16306598)                    --Q-GSLLKTLP--PAR--CRAGAQQPAVALETCNPQP---CPAR-WEVSEPSSCTSAGG   [821]
Fugu 13                                     --L-GGTVTQVQ--NSD--CPADKAPTSVER--CNLQH---CPAR-WQVSEPGECSAVCG   [755]
Homo ADAMTS1 (6525075)                       --D-GGVLSHES-------CDPLKKPKHFIDF-CTMAE---CS-----------------   [967]
Mus 1 (1813340)                             --D-GGVLSNES-------CDPLKKPKHYIDF-CTLTQ---CS-----------------   [951]
Homo ADAMTS4 (12643637)                      ------------------------------------------------------------   [837]
Mus 4 (26350615)                            ------------------------------------------------------------   [845]
Fugu 4                                      ------------------------------------------------------------   [695]
Homo ADAMTS8 (21536392)                     ------------------------------------------------------------   [890]
Mus 8 (6708110)                             ------------------------------------------------------------   [905]
Fugu 8                                      --T-GKTVEPEK-------CDPNKKPQPVSTE-CHNNE---CL-----------------   [867]
Homo ADAMTS5 (7768707)                      ------------------------------------------------------------   [930]
Mus 5 (5923784)                             ------------------------------------------------------------   [930]
Fugu 5                                      --D-GKLSKG---------CILSFRPSAFRP--CVVKK---C------------------   [921]
Ciona 15 (0100137065)                       --P------------------------------------------IWNSGSWGPCSRSCG   [946]
Homo ADAMTS15 (19171175)                     --G-GRLLARDQ-------CNLHRKPQELDF--CVLRP---C------------------   [950]
Fugu 15                                     --K-GVNLPREH-------CSWKRKPQELDL--CYL------------------------   [891]
Homo ADAMTS9 (27463365)                      --S-RLDGKTEK--VDDGFCSSHPKPSNREK--CSGE----CNTGGWRYSAWTECSKSCD   [1014]
Fugu 9                                      --S-HADGETEK--VDDRFCSGQHKPDNKEG--CHGD----CNPGGWEYSPWSECSKSCG   [860]
Homo ADAMTS20 (28460690)                     --S-IHEGQTVQ--VDDHYCGDQLKPPTQEL--CHGN----CVFTRWHYSEWSQCSRSCG   [970]
Mus 20 (29500513)                           --S-VHKGQAVP--VGDQYCGDQLKPPSREP--CHGS----CVLTRWHYSEWSQCSRSCG   [979]
Fugu 20                                     --S-LLKRQSER--VVASVCANAIKPRTRES--CHGD----CPLKSWQYGAWSECSKTCG   [950]
Ciona 9 (0100132719)                        --NRIHRSNHVVETVSDSLCP-QPKPGGVSS--CEGD----CLSTRWTYGEWGECSRSCG   [1123]
Apis 9                                      ----LNSNHPPRA-IPPHSCAHLERPNETES--CMGP----CEDAHWSYGKWSACNATCG   [868]
Drosophila CG6107 (20151419)                --F-TNMQRSNI--VDMSYCKLKFDVAYHEE--CREG----C----WVLSEWSTCSKSCG   [1186]
Caenorhabditis gon-1 CEt13h10 (3879882)     --E-GDRQTPAS--EHL--CDRNSKPSDIAS--CYID----CSGRKWNYGEWTSCSETCG   [1035]
introns                                     --85------------------------------------------86------87----   [2]
```

AC Nicholson, SB Malik, JM Logsdon Jr, E van Meir, 2004. Functional evolution of ADAMTS genes: evidence]
[from analyses of phylogeny and gene organization. Contact: john-logsdon@uiowa.edu]
[Intron phases marked 0, 1, 2]

```
[                                                        1570      1580      1590      1600      1610      1620]
[                                                        .         .         .         .         .         .]

mask                                         ------------------------------------------------------------   [571]
Homo ADAMTS6 (11131380)                      P-GFKHRI-----VLCKSSDLSKTFPAAQ----CPEESKPPVRIR---CSLGR--CPPPR   [1021]
Fugu 6                                       P-GFKHRI-----ALCKSSDLAKTFPPAQ----CSSHSKPPVRVR---CSLGR--CPPPR   [1025]
Ciona 6 (0100146470)                         A-GWKTRV-----VTCASANGDETYDDSQ----CDVNQKPTVKAH---CMGRA--CRAK-   [1040]
Homo ADAMTS10 (17432918)                     P-GLRHRV-----VLCKSADHRATLPPAH----CSPAAKPPATMR---CNLRR--CPPAR   [1006]
Mus 10 (27502095)                            P-GLRHRV-----VLCKSADQRSTLPPGH----CLPAAKPPSTMR---CNLRR--CPPAR   [972]
Fugu 10                                      P-GYRHRV-----VVCKSGESGDTLPESK----CPKHSRPTSRVR---CNLKR--CPP--   [957]
Homo ADAMTS16 (21265061)                     K-GWRKRA-----VACKSTNPSARAQLLPDAV-CTSEPKPRMHEA---CLLQR--CHKPK   [1052]
Mus 16 (21961374)                            ------------------------------------------------------------   [900]
Fugu 16                                      S-GLRKRT-----VLCTSTKPGVETRTLP-DSLCAGLPKPGSQES---CFIKR--CQKLR   [1015]
Ciona 16 (0100138085)                        R-GVRNRH-----VRCRS--GTRSLRNAR----CDRRVRPKSREP---CLVTY--CQRKY   [914]
Homo ADAMTS18 (21265067)                     R-GVRKRE-----LLCKGSAAETLPESQ-----CTSLPRPELQEG---CVLGR--CPKNS   [1052]
Fugu 18                                      ------------------------------------------------------------   [742]
Homo ADAMTS12 (13569928)                     G-GVRIRS-----VTCAKNHDEP----------CDVTRKPNSRAL---CGLQQ--CPSSR   [999]
Mus 12 (27817773)                            G-GVRIRS-----VTCAKNLNEP----------CDKTRKPNSRAL---CGLQQ--CPFSR   [1004]
Fugu 12                                      G-GVRSRT-----VTCAVAPKKT----------CDPSTKPRSRSL---CALLS--CPNSG   [1007]
Homo ADAMTS7 (11131377)                      E-GTQRRN-----VLCTNDTGVP----------CDEAQQPASEVT---CSLPL--CR---   [995]
Fugu 7                                       ------------------------------------------------------------   [930]
Ciona 7 (genewise.34.12.1)                   N-GLRHRR-----VVCVEKESSIPNDT------CNVKLKPRMHKK---C-----------   [965]
Homo ADAMTS17 (21265064)                     K-GVWKRT-----VACTNSQGK-----------CDASTRPRAEEA---CEDYSG-CYE--   [1002]
Homo ADAMTS19 (19525737)                     K-GIRHRT-----VRCTNPRKK-----------CVLSTRPREAED---CEDYSK-CYV--   [1090]
Drosophila dCG4096 (22831757)                PVGFVAPPQRRRSVVCLDEHDVVVADAE-----CGHLQKPAEMEP---CESSLPICRTK-   [1054]
Homo ADAMTS14 (29337086)                     E-GIQQRQ-----VVCRTNANSLGH--------CEGDRPDTVQV----CSLPA--CG---   [1024]
Fugu 14                                      E-GIQQRQ-----VVCKASDNTAAE--------CEGEKPETVII----CKLIS--CP---   [991]
Homo ADAMTS3 (2224673)                       E-GTEVRQ-----VLCRAGDH------------CDGEKPESVRA----CQLPP--CNDEP   [1019]
Fugu 3                                       E-GVERRL-----VTCRIGDQ------------CSREKPEAVRP----CRLGP--CHDEP   [779]
Ciona 3 (0100146117)                         E-GIQQRR-----VICRSSNHSLTSNHE-----CPQPQPPDVRE----CQLVA--CSVQS   [965]
Homo ADAMTS2 (7656867)                       N-GTQERP-----VPCRTADDSFGI--------CQEERPETART----CRLGP--CP-RN   [1031]
Mus 2 (28204840)                             N-GTQERP-----VLCRTADDNFGV--------CREERPETARI----CRLAP--CP-RN   [1032]
Fugu 2                                       N-GTQQRK-----ALCHTRDNTIGL--------CLDSKPDTIRV----CRLDP--CP---   [799]
Homo ADAMTS13 (16306598)                     A-GLALEN-----ETCVPGADGLEAPVTEGPG-SVDEKLPAPEP----CVGMS--CPPGW   [868]
Fugu 13                                      P-GEAKQV-----VSCVRPENGQDVKVDERF--CQRLKRPADSV---PCVVNV--CPIGW   [802]
Homo ADAMTS1 (6525075)                       ------------------------------------------------------------   [967]
Mus 1 (1813340)                              ------------------------------------------------------------   [951]
Homo ADAMTS4 (12643637)                      ------------------------------------------------------------   [837]
Mus 4 (26350615)                             ------------------------------------------------------------   [845]
Fugu 4                                       ------------------------------------------------------------   [695]
Homo ADAMTS8 (21536392)                      ------------------------------------------------------------   [890]
Mus 8 (6708110)                              ------------------------------------------------------------   [905]
Fugu 8                                       ------------------------------------------------------------   [867]
Homo ADAMTS5 (7768707)                       ------------------------------------------------------------   [930]
Mus 5 (5923784)                              ------------------------------------------------------------   [930]
Fugu 5                                       ------------------------------------------------------------   [921]
Ciona 15 (0100137065)                        L-GTKMRL-----VRCQTRSGHVLSESR-----CEAGTRPSEAHG---CRLRN--CT---   [987]
Homo ADAMTS15 (19171175)                     ------------------------------------------------------------   [950]
Fugu 15                                      ------------------------------------------------------------   [891]
Homo ADAMTS9 (27463365)                      G-GTQRRR-----AICVNTRNDVLDDSK-----CTHQEKVTIQR----CSEFP--CPQ--   [1055]
Fugu 9                                       G-GTRRRG-----AACRKAAEADGDESK-----CSQRDKLTSQP----CNEFL--CPQ--   [901]
Homo ADAMTS20 (28460690)                     G-GERSRE-----SYCMNNFGHRLADNE-----CQELSRVTREN----CNEFS--CPS--   [1011]
Mus 20 (29500513)                            G-GDKTRE-----SYCVNGFGHRLAESE-----CRELPRVVLEN----CNEFP--CPS--   [1020]
Fugu 20                                      R-GSRSRE-----SYCMNDQGRRLADRE-----CSERQRVVAES----CNDQP--CPK--   [991]
Ciona 9 (0100132719)                         G-GSQTRS-----SVCHDSRGIELNPTQ-----CEDNERVVSRT----CGNVA--CPQ--   [1164]
Apis 9                                       G-GIQYRT-----ATCIDSNQRTVSEEN-----CMGQRKVLENA----CANDP--CPK--   [909]
Drosophila CG6107 (20151419)                 T-GSQQRE-----AHCYLHNSRVSDDL------CNPRTKPHLNTLIGICNTES--CPTYT   [1232]
Caenorhabditis gon-1 CEt13h10 (3879882)      SNGKMHRK-----SYCVDDSNRRVDESL-----CGREQKEATERE---CNRIP--CPR--   [1078]
introns                                      ------------------------------------------------------------   [2]
```

```
[                                            1630      1640      1650      1660      1670      1680]
[                                               .         .         .         .         .         .]

mask                                         ------------------------------------------------------------   [571]
Homo ADAMTS6 (11131380)                      -----------------------WVTGDWGQ----CSAQCGLGQQMRTVQCLSYTGQASS   [1054]
Fugu 6                                       -----------------------WTPGEWGQ----CSAQCGLGQQMRTVQCLSYTGQPSN   [1058]
Ciona 6 (0100146470)                         -----------------------WVTYKWGQ----CSATCGRGTKRRRVVCIDPVTNRAS   [1073]
Homo ADAMTS10 (17432918)                     -----------------------WVAGEWGE----CSAQCGVGQRQRSVRCTSHTGQASH   [1039]
Mus 10 (27502095)                            -----------------------WVTSEWGE----CSTQCGLGQQQRTVRCTSHTGQPSR   [1005]
Fugu 10                                      -------------------------PLWVTGPWGECSAKCGLGQEMRSVQCLTHTGQLSN   [992]
Homo ADAMTS16 (21265061)                      KLQ--------------------WLVSAWSQ----CSVTCERGTQKRFLKCAEKYVSGKY   [1088]
Mus 16 (21961374)                            ------------------------------------------------------------   [900]
Fugu 16                                      KVQ--------------------WFVSTWQE-----------------------DTAGKY   [1032]
Ciona 16 (0100138085)                        Q----------------------WFITAWDQ----CSVTCGEGIQRRELKCSFKGRNGRY   [948]
Homo ADAMTS18 (21265067)                     RLQ--------------------WVASSWSE----CSATCGLGVRKREMKCSEKGFQGKL   [1088]
Fugu 18                                      ------------------------------------------------------------   [742]
Homo ADAMTS12 (13569928)                     RVLKPNKGTISNGKNPPTLKPVPPPTSRPRMLTTPTGPESMSTSTPAISSPSPTTASKEG   [1059]
Mus 12 (27817773)                            RVLKPHKDIAPSGKNQSTAEHDPFKPIPAPTSRPTPLSTPTVPESMSTSTPTINSLGSTI   [1064]
Fugu 12                                      -----------------------------------LQRRPGPLPKYRSIYPPRNQPTRQP   [1032]
Homo ADAMTS7 (11131377)                      -----------------------WPLGTLGPEGSGSGSSSHELFNEADFIPHHLAPRPSP   [1032]
Fugu 7                                       ------------------------------------------------------------   [930]
Ciona 7 (genewise.34.12.1)                   ------------------------------------------------------------   [965]
Homo ADAMTS17 (21265064)                     -----------------------WKTGDWST----CSSTCGKGLQSRVVQCMHKVTGRHG   [1035]
Homo ADAMTS19 (19525737)                     -----------------------WRMGDWSK----CSITCGKGMQSRVIQCMHKITGRHG   [1123]
Drosophila dCG4096 (22831757)                ------------------------------------------------------------   [1054]
Homo ADAMTS14 (29337086)                     GNHQNSTVRADV-----------WELVTPEGQWVPQSGPLHPIYKISSTEPCTGDRSVFC   [1073]
Fugu 14                                      ------------------------------------------------------------   [991]
Homo ADAMTS3 (2224673)                       ------------------------------------------CLGDKSIF----------   [1027]
Fugu 3                                       ------------------------------------------CYGDKSIFCQMEVLARYC   [797]
Ciona 3 (0100146117)                         TETCVKER---------------WLFCRYGIQHCGIREYQKLCCLSC-------------   [997]
Homo ADAMTS2 (7656867)                       ISDPSKKSYVVQ-----------WLSRPDPDSPIRKISSKGHCQGDKSIFCRMEVLSRY-   [1079]
Mus 2 (28204840)                             GSDPSKKSYVVQ-----------WLSRPDPDSPIQKISSKDQCQGDKSMFCRMEVLSRY-   [1080]
Fugu 2                                       ------------------------------------------------------------   [799]
Homo ADAMTS13 (16306598)                     GHLDATSAGEKAPSPWGSIRTGAQAAHVWTPAAGSCSVSCGRGLMELRFLCMDSALRVPV   [928]
Fugu 13                                      ESTGQSLPDTKQD-----------PVYVWSPIISKCSKTCGNGTLQLWFSCVDHQTRLGV   [851]
Homo ADAMTS1 (6525075)                       ------------------------------------------------------------   [967]
Mus 1 (1813340)                              ------------------------------------------------------------   [951]
Homo ADAMTS4 (12643637)                      ------------------------------------------------------------   [837]
Mus 4 (26350615)                             ------------------------------------------------------------   [845]
Fugu 4                                       ------------------------------------------------------------   [695]
Homo ADAMTS8 (21536392)                      ------------------------------------------------------------   [890]
Mus 8 (6708110)                              ------------------------------------------------------------   [905]
Fugu 8                                       ------------------------------------------------------------   [867]
Homo ADAMTS5 (7768707)                       ------------------------------------------------------------   [930]
Mus 5 (5923784)                              ------------------------------------------------------------   [930]
Fugu 5                                       ------------------------------------------------------------   [921]
Ciona 15 (0100137065)                        ------------------------------------------------------------   [987]
Homo ADAMTS15 (19171175)                     ------------------------------------------------------------   [950]
Fugu 15                                      ------------------------------------------------------------   [891]
Homo ADAMTS9 (27463365)                      -----------------------WKSGDWSE----CLVTCGKGHKHRQVWCQFGEDRLND   [1088]
Fugu 9                                       -----------------------WKTGDWSE----CLVTCGKGYKHRQTWCQFGEDRLDD   [934]
Homo ADAMTS20 (28460690)                     -----------------------WAASEWSE----CLVTCGKGTKQRQVWCQLNVDHLSD   [1044]
Mus 20 (29500513)                            -----------------------WATSEWSE----CPVTCGKGMKQRQVWCQLSEDPMRD   [1053]
Fugu 20                                      -----------------------WSPGEWSE----CLVTCGKGVRHRQVSCSASTEEKLS   [1024]
Ciona 9 (0100132719)                         -----------------------WVVSAFSP----CMATCGSGVRHRRVWCAIRGVVRPN   [1197]
Apis 9                                       -----------------------WTFKNWSN----CSVTCGIGKQYSRYACHIENRVVPD   [942]
Drosophila CG6107 (20151419)                 KSPNALAVSN-------------WVIGEWGE----CNEWCEKTRSVS---CSHPYGIG--   [1270]
Caenorhabditis gon-1 CEt13h10 (3879882)      -----------------------WVYGHWSE----CSRSCDGGVKMRHAQCLDAADRETH   [1111]
introns                                      ------------------------------------------------------------   [2]
```

```
[                                                    1690      1700      1710      1720      1730      1740]
[                                                       .         .         .         .         .         .]

mask                                         ------------------------------------------------------------   [571]
Homo ADAMTS6 (11131380)                      D-------CLETVRPPSMQQ--CESK-CDS---------TPISNTEECKDVNKVAYCPLV   [1095]
Fugu 6                                       E-------CSDTLRPAMMQQ--CESK-CDATPIS---------NGDECKDVNKVAYCPLV   [1099]
Ciona 6 (0100146470)                         NT------CSRNKKPKTTVS--CSSSSPSCVRS-----PVRAPAAGRCIDDPSVAYCPLV   [1120]
Homo ADAMTS10 (17432918)                     E-------CTEALRPPTTQQ--CEAK-CDS--------PTPGDGPEECKDVNKVAYCPLV   [1081]
Mus 10 (27502095)                            E-------CTEALRPSTMQQ--CEAK-CDSV-------VPPGDGPEECKDVNKVAYCPLV   [1048]
Fugu 10                                      ECLEHQRPASMQQCKSKCDLSLPVGTDNSE----------------ECKDVNTVAYCPLV   [1036]
Homo ADAMTS16 (21265061)                     RELASKK-CSHLPKPSLELERACAPLPCPRHPPFAAAGPSRGSWFASPWSQCTASCGGGV   [1147]
Mus 16 (21961374)                            ------------------------------------------------------------   [900]
Fugu 16                                      RELTDKK-CHHVPKPAVELQRPCVLKECPVR----------TTPALHRWSPYHHTFPQPL   [1081]
Ciona 16 (0100138085)                        RPVVARR-CRRARRPEVSLEKTCTLSTCPSPRPR---------WIVGTWQKCSVSCGGGL   [998]
Homo ADAMTS18 (21265067)                     ITFPERR-CRNIKKPNLDLEETCNRRACPAHPVYNMVAG----WYSLPWQQCTVTCGGGV   [1143]
Fugu 18                                      ------------------------------------------------------------   [742]
Homo ADAMTS12 (13569928)                     DLGGKQWQDSSTQPELSSRYLISTGSTSQPILTSQSLSIQPSEENVSSSDTGPTSEG---   [1116]
Mus 12 (27817773)                            ASQEDANGMGWQNNSTQAEEGSHFPTSSGSTSQVPVTSWSLSIQPDDENVSSSAIGPTSE   [1124]
Fugu 12                                      PQSTRDPRSAATSATPTTSVTVMKTTAEE-------------------------------   [1061]
Homo ADAMTS7 (11131377)                      ASSPKPG-TMGNAIEEEAPELDLPGPVFVDDFYYDYNFINFHEDLSYGPSEEPDLDLAGT   [1091]
Fugu 7                                       ------------------------------------------------------------   [930]
Ciona 7 (genewise.34.12.1)                   ------------------------------------------------------------   [965]
Homo ADAMTS17 (21265064)                     SE------CPALSKPAPYRQ--CYQEVCNDRINANTITSPRLAALTYKCTRDQWTVYCRV   [1087]
Homo ADAMTS19 (19525737)                     NE------CFSSEKPAAYRP--CHLQPCNEKINVN---------------------------   [1150]
Drosophila dCG4096 (22831757)                ------------------------------------------------------------   [1054]
Homo ADAMTS14 (29337086)                     QMEVLDRYCSIPGYHRL-----CCVS-CIKKASGPNPGPDPGPTSLPP------------   [1115]
Fugu 14                                      ------------------------------------------------------------   [991]
Homo ADAMTS3 (2224673)                       ---------------------------------------CQMEVLARYCSIPGYNKLCCE   [1048]
Fugu 3                                       SIPG---------------------------------------YNKLCCDSCSRRSGTLS   [818]
Ciona 3 (0100146117)                         ------------------------------------------------------------   [997]
Homo ADAMTS2 (7656867)                       --------CSIPGYNKL-----SCKS-CNLYNNLTNV-EGRIEPPPGKHNDIDVFMPTLP   [1124]
Mus 2 (28204840)                             --------CSIPSYNKL-----CCKS-CNPPRNLSNTEDGGVEPPPGKHNDIDVFMPTLP   [1126]
Fugu 2                                       ------------------------------------------------------------   [799]
Homo ADAMTS13 (16306598)                     QEEL----CGLASKPGSRREV-CQAVPCPA------------RWQYKLA-ACSVSCGRGV   [970]
Fugu 13                                      EDLYCDASTKPPPQSESCSTSPCPPTWRFKQG------------------ACSVTCGGGL   [893]
Homo ADAMTS1 (6525075)                        ------------------------------------------------------------   [967]
Mus 1 (1813340)                              ------------------------------------------------------------   [951]
Homo ADAMTS4 (12643637)                       ------------------------------------------------------------   [837]
Mus 4 (26350615)                             ------------------------------------------------------------   [845]
Fugu 4                                       ------------------------------------------------------------   [695]
Homo ADAMTS8 (21536392)                      ------------------------------------------------------------   [890]
Mus 8 (6708110)                              ------------------------------------------------------------   [905]
Fugu 8                                       ------------------------------------------------------------   [867]
Homo ADAMTS5 (7768707)                       ------------------------------------------------------------   [930]
Mus 5 (5923784)                              ------------------------------------------------------------   [930]
Fugu 5                                       ------------------------------------------------------------   [921]
Ciona 15 (0100137065)                        ------------------------------------------------------------   [987]
Homo ADAMTS15 (19171175)                     ------------------------------------------------------------   [950]
Fugu 15                                      ------------------------------------------------------------   [891]
Homo ADAMTS9 (27463365)                      RM------CDPETKPTSMQT--CQQPECAS-------------WQAGPWGQCSVTCGQGY   [1127]
Fugu 9                                       RLCGSPKPESVQTCQQQECAS----------------------WQVGPWGQCTTSCGPGY   [972]
Homo ADAMTS20 (28460690)                     GF------CNSSTKPESLSP--CELHTCAS-------------WQVGPWGPCTTTCGHGY   [1083]
Mus 20 (29500513)                            GF------CNASTKPESLRP--CELRACAS-------------WHVGPWGSCTATCGHGY   [1092]
Fugu 20                                      DR-------FCDPSRKPPTVGNCELPECAS-------------WKVGAWGECGVTCGHGF   [1064]
Ciona 9 (0100132719)                         NE------CSRQRKPPTTTL--CSNHDCPT-------------WSYDNWGPCSVTCGEGT   [1236]
Apis 9                                       NY------CGKPPLEITQA---CNSGPCEQ-------------WHKGDWSACSVTCGEGI   [980]
Drosophila CG6107 (20151419)                 --------CGSRKPKDVRK---CCHIKYTS-----------------DWTDCSVQCGEGV   [1302]
Caenorhabditis gon-1 CEt13h10 (3879882)      TSR-----CGPAQTQEH-----CNEHACTW-------------WQFGVWSDCSAKCGDGV   [1148]
introns                                      ------------------------------------------------------------   [2]
```

```
[                                                       1750      1760      1770      1780      1790      1800]
[                                                          .         .         .         .         .         .]

mask                                          ------------------------------------------------------------   [571]
Homo ADAMTS6 (11131380)                       LKFKFCSRAYFRQMCCKTCQGH--------------------------------------   [1117]
Fugu 6                                        LKFKFCSRAYFRQMCCKTCQGH--------------------------------------   [1121]
Ciona 6 (0100146470)                          LQFHYCNRAYFRKACCATCSK---------------------------------------   [1141]
Homo ADAMTS10 (17432918)                      LKFQFCSRAYFRQMCCKTCQGH--------------------------------------   [1103]
Mus 10 (27502095)                             LKFQFCSRAYFRQMCCKTCQGR--------------------------------------   [1070]
Fugu 10                                       LRFKFCSRPYFRQMCCKTCQGH--------------------------------------   [1058]
Homo ADAMTS16 (21265061)                       QTRSVQCLAGGRPASG--------CLLH-QK-PSASLACNTHF-CP--------------   [1182]
Mus 16 (21961374)                             ------------------------------------------------------------   [900]
Fugu 16                                       PHPPPPAEWRSSPWSQ--------------------------------------------   [1097]
Ciona 16 (0100138085)                         QMRAVRCLQFGRPSHSCSISSKAPNKRA-CN-TYKCRKKIQTA-CEDTFKWCYLVP---Q   [1052]
Homo ADAMTS18 (21265067)                       QTRSVHCVQQGRPSSS--------CLLH-QK-PPVLRACNTNF-CP--------------   [1178]
Fugu 18                                       ------------------------------------------------------------   [742]
Homo ADAMTS12 (13569928)                      -GLVATTTSGSGLSSSRNPITWPVTPFY-NT-LTKGPEMEIHS-GSGEEREQPEDK---D   [1169]
Mus 12 (27817773)                             GDFWATTTSDSGLSSSDA-MTWQVTPFY-ST-MTTDPEVEIHS-GSGEDSDQPLNK---D   [1177]
Fugu 12                                       ------------------------------------------------------------   [1061]
Homo ADAMTS7 (11131377)                       GDRTPPPHSRPAAPSTGSPVPATEPPAA-KE-EGVLGPWSPSP-WPSQAGRSPPPP---S   [1145]
Fugu 7                                        ------------------------------------------------------------   [930]
Ciona 7 (genewise.34.12.1)                    ------------------------------------------------------------   [965]
Homo ADAMTS17 (21265064)                      IREKNLCQDMRWYQRCCQTCRDFYANKM-RQ-PPPSS-----------------------   [1122]
Homo ADAMTS19 (19525737)                      ------------------------------------------------------------   [1150]
Drosophila dCG4096 (22831757)                 ------------------------------------------------------------   [1054]
Homo ADAMTS14 (29337086)                      ------------------------------------------------------------   [1115]
Fugu 14                                       ------------------------------------------------------------   [991]
Homo ADAMTS3 (2224673)                        SCSKRSSTLPPPYLLEAAETHDDVISNP-SD-LPRSLVMPTSL-VPYHSETPAKKM---S   [1102]
Fugu 3                                        MFAEAAETEEDLRFGSASQLLETLTAS---------------------------------   [845]
Ciona 3 (0100146117)                          ------------------------------------------------------------   [997]
Homo ADAMTS2 (7656867)                        VPTVAMEVRPSPSTPLEVPLNASSTNAT-ED-HPETNAVDEPY-KIHGLEDEVQPP---N   [1178]
Mus 2 (28204840)                              GPTVATQVQPSPGPPLEAPLNVSSTNAT-ED-HPETNAVDVPY-KIHGVDEEVPSP---N   [1180]
Fugu 2                                        ------------------------------------------------------------   [799]
Homo ADAMTS13 (16306598)                      VRRILYCARAHGEDDGEEILLDTQCQGL-PR-PEPQEACSLEP-CPPRWKVMSLGP---C   [1024]
Fugu 13                                       ANRVLYCGREAGGEEEEVLE-DSDCSHS-VK-PTAVVSCNGHS-CPARWKVSRTSP---C   [946]
Homo ADAMTS1 (6525075)                        ------------------------------------------------------------   [967]
Mus 1 (1813340)                               ------------------------------------------------------------   [951]
Homo ADAMTS4 (12643637)                       ------------------------------------------------------------   [837]
Mus 4 (26350615)                              ------------------------------------------------------------   [845]
Fugu 4                                        ------------------------------------------------------------   [695]
Homo ADAMTS8 (21536392)                       ------------------------------------------------------------   [890]
Mus 8 (6708110)                               ------------------------------------------------------------   [905]
Fugu 8                                        ------------------------------------------------------------   [867]
Homo ADAMTS5 (7768707)                        ------------------------------------------------------------   [930]
Mus 5 (5923784)                               ------------------------------------------------------------   [930]
Fugu 5                                        ------------------------------------------------------------   [921]
Ciona 15 (0100137065)                         ------------------------------------------------------------   [987]
Homo ADAMTS15 (19171175)                      ------------------------------------------------------------   [950]
Fugu 15                                       ------------------------------------------------------------   [891]
Homo ADAMTS9 (27463365)                       QLRAVKCIIGTYMSVVDDND----CNAA-TR-PTDTQ-CSAT--CGKGTRMRYVSCRDEN   [1178]
Fugu 9                                        QMRAVKCVVGPYGSVVDDTE----CNAA-TR-PTETQ-CSAS--CGKGTRMRYVSCRD-N   [1022]
Homo ADAMTS20 (28460690)                      QMRDVKCVNELASAVLEDTE----CHEA-SR-PSDRQ-CSVS--CGRGTQARYVSCRDAL   [1134]
Mus 20 (29500513)                             QMRAVKCISEIFGTMLDDRE----CPQA-SR-PSDRQDCILAP-CLAIPEVGATSL----   [1141]
Fugu 20                                       QMRAVRCVSGSYGDRVDDRE----CNAA-VR-PRDSQ-CSVS--CGKGKRARYVSCRDAR   [1115]
Ciona 9 (0100132719)                          SVRLYRCVDHASTAVDDGL-----CNTALLR-PDTRA-CYAQRACDVSRISRTYDNDGFP   [1289]
Apis 9                                        KRRKVICKTLDGITSNK-------CSA--SNKPDNITACILKS-CPTVNVTPIKYSSDPP   [1030]
Drosophila CG6107 (20151419)                  KRKKQSCTRVYKPDVPGTRKRRVYV----DE-S-YCISRKVHR-PKLRTTTKS-CR---I   [1351]
Caenorhabditis gon-1 CEt13h10 (3879882)       QYRDANCTDRHRSVLPEHR-----CLKM-EK-IIT-KPCHRES-CPKYKLGEWSQ----C   [1195]
introns                                       ------------------------------------------------------------   [2]
```


AC Nicholson, SB Malik, JM Logsdon Jr, E van Meir, 2004. Functional evolution of ADAMTS genes: evidence]
[from analyses of phylogeny and gene organization. Contact: john-logsdon@uiowa.edu]
[Intron phases marked 0, 1, 2]

```
[                                          1810      1820      1830      1840      1850      1860]
[                                             .         .         .         .         .         .]

mask                                       ------------------------------------------------------------   [571]
Homo ADAMTS6 (11131380)                    ------------------------------------------------------------   [1117]
Fugu 6                                     ------------------------------------------------------------   [1121]
Ciona 6 (0100146470)                       ------------------------------------------------------------   [1141]
Homo ADAMTS10 (17432918)                   ------------------------------------------------------------   [1103]
Mus 10 (27502095)                          ------------------------------------------------------------   [1070]
Fugu 10                                    ------------------------------------------------------------   [1058]
Homo ADAMTS16 (21265061)                   ------------------------------------------------------------   [1182]
Mus 16 (21961374)                          ------------------------------------------------------------   [900]
Fugu 16                                    ------------------------------------------------------------   [1097]
Ciona 16 (0100138085)                      HGQCSHKYFGVACCK--TCKA-SS---GNR------------------------------   [1076]
Homo ADAMTS18 (21265067)                   ------------------------------------------------------------   [1178]
Fugu 18                                    ------------------------------------------------------------   [742]
Homo ADAMTS12 (13569928)                   ESNPVIWTKIRVPGN--DAPV-E----STEMPLAPPLTPDLSRESWWPPFSTVMEGLLPS   [1222]
Mus 12 (27817773)                          KSNSVIWNKIGVPEH--DAPM-ET---DAELPLGPPPTSYMGEEPSWPPFSTKMEGSLPA   [1231]
Fugu 12                                    ------------------------------------------------------------   [1061]
Homo ADAMTS7 (11131377)                    EQTPGNPLINFLPEE--DTPI-GA---PDLGLPSLSWPRVSTDGLQTPATPESQNDFPVG   [1199]
Fugu 7                                     ------------------------------------------------------------   [930]
Ciona 7 (genewise.34.12.1)                 ------------------------------------------------------------   [965]
Homo ADAMTS17 (21265064)                   ------------------------------------------------------------   [1122]
Homo ADAMTS19 (19525737)                   ------------------------------------------------------------   [1150]
Drosophila dCG4096 (22831757)              ------------------------------------------------------------   [1054]
Homo ADAMTS14 (29337086)                   ------------------------------------------------------------   [1115]
Fugu 14                                    ------------------------------------------------------------   [991]
Homo ADAMTS3 (2224673)                     LSSISSVGGPNAYAA--FRPN-SK---PDGANLRQRSAQQAGSKTVRLVTVPSSPPTKRV   [1156]
Fugu 3                                     ------------------------------------------------------------   [845]
Ciona 3 (0100146117)                       ------------------------------------------------------------   [997]
Homo ADAMTS2 (7656867)                     LIPRRPSPYEKTRNQ--RIQE-LI---DEMRKKEMLGKF---------------------   [1211]
Mus 2 (28204840)                           LIPRRPSLYVKTRNQ--RIQE-LI---NAVQRKEKPGKF---------------------   [1213]
Fugu 2                                     ------------------------------------------------------------   [799]
Homo ADAMTS13 (16306598)                    SASCGLGTARRSVAC--VQLD-QG---QDVEVDEAACAALVRPEASVPCLIADCTYRWHV   [1078]
Fugu 13                                    SASCDLGIAQRAVSC--VRFI-NG---TESAVAEEKCHAGVKPATSVPCLVQVCTFRWEV   [1000]
Homo ADAMTS1 (6525075)                      ------------------------------------------------------------   [967]
Mus 1 (1813340)                            ------------------------------------------------------------   [951]
Homo ADAMTS4 (12643637)                     ------------------------------------------------------------   [837]
Mus 4 (26350615)                           ------------------------------------------------------------   [845]
Fugu 4                                     ------------------------------------------------------------   [695]
Homo ADAMTS8 (21536392)                    ------------------------------------------------------------   [890]
Mus 8 (6708110)                            ------------------------------------------------------------   [905]
Fugu 8                                     ------------------------------------------------------------   [867]
Homo ADAMTS5 (7768707)                     ------------------------------------------------------------   [930]
Mus 5 (5923784)                            ------------------------------------------------------------   [930]
Fugu 5                                     ------------------------------------------------------------   [921]
Ciona 15 (0100137065)                      ------------------------------------------------------------   [987]
Homo ADAMTS15 (19171175)                   ------------------------------------------------------------   [950]
Fugu 15                                    ------------------------------------------------------------   [891]
Homo ADAMTS9 (27463365)                    -GS---VADESAC-ATLPRPVAKEE-CSVTPCGQWKALDW--------------------   [1212]
Fugu 9                                     QGG---VADESAC-AHLPKPPAREV-CSVVACGQWKVLEW--------------------   [1057]
Homo ADAMTS20 (28460690)                    -DR---IADESYC-AHLPRPAEIWD-C-FTPCGEWQAGDW--------------------   [1167]
Mus 20 (29500513)                           -----------------PAIPLGRA-------AQWRHGSWTPCSVSCGRGSQARYVSCRD   [1177]
Fugu 20                                    GGVA-----DESQCTHLPRPPELST-C-FSPCGKWRAGEWSPCSVTCGVGKMTRHVDCSN   [1168]
Ciona 9 (0100132719)                       SI-----------------------------NARWRTGSWTPCSSSCGSGVRERYVSCRL   [1320]
Apis 9                                     YEIPSQQDNEVHDIIFHYGYKWHTETQKCSKPCIKGYMNTTVKCVSIETGIVAPDRYCDN   [1090]
Drosophila CG6107 (20151419)               NCK--WNASDWRRCPADCSEE-YQ---TRDVRCESFQGDGVEDKHCDAKKRPSKRRICNN   [1405]
Caenorhabditis gon-1 CEt13h10 (3879882)    SVSCEDGWSSRRVSC--VSGN-GT---EVDMSLC-GTASDRPASHQTCNLGTCPF--WRN   [1246]
introns                                    ------------------------------------------------------------   [2]
```

```
[                                                              1870      1880      1890      1900      1910      1920]
[                                                                 .         .         .         .         .         .]

mask                                      ------------------------------------------------------------   [571]
Homo ADAMTS6 (11131380)                   ------------------------------------------------------------   [1117]
Fugu 6                                    ------------------------------------------------------------   [1121]
Ciona 6 (0100146470)                      ------------------------------------------------------------   [1141]
Homo ADAMTS10 (17432918)                  ------------------------------------------------------------   [1103]
Mus 10 (27502095)                         ------------------------------------------------------------   [1070]
Fugu 10                                   ------------------------------------------------------------   [1058]
Homo ADAMTS16 (21265061)                  ------------------------------------------------------------   [1182]
Mus 16 (21961374)                         ------------------------------------------------------------   [900]
Fugu 16                                   ------------------------------------------------------------   [1097]
Ciona 16 (0100138085)                     ------------------------------------------------------------   [1076]
Homo ADAMTS18 (21265067)                  ------------------------------------------------------------   [1178]
Fugu 18                                   ------------------------------------------------------------   [742]
Homo ADAMTS12 (13569928)                  Q-RPTTSE-TGTPRVEGMVTEKPANTLLP-LGGDHQPEPSGKTANRNHLKLPNNMNQTKS   [1279]
Mus 12 (27817773)                         W-SFKNET-PRDDGMIAEKSRKIPLPLAG-DH---HPATSEKLENHDKLALPNTTNPTQG   [1285]
Fugu 12                                   ------------------------------------------------------------   [1061]
Homo ADAMTS7 (11131377)                   K-DSQSQL-PPPWRDRTNEVFKDDEEPKG-RGAPHLPPRPSSTLPPLSPVGSTHSSPSPD   [1256]
Fugu 7                                    ------------------------------------------------------------   [930]
Ciona 7 (genewise.34.12.1)                ------------------------------------------------------------   [965]
Homo ADAMTS17 (21265064)                  ------------------------------------------------------------   [1122]
Homo ADAMTS19 (19525737)                  ------------------------------------------------------------   [1150]
Drosophila dCG4096 (22831757)             ------------------------------------------------------------   [1054]
Homo ADAMTS14 (29337086)                  ------------------------------------------------------------   [1115]
Fugu 14                                   ------------------------------------------------------------   [991]
Homo ADAMTS3 (2224673)                    H-LSSASQ-MAAASFFAASDSIGASSQAR-TSKKDGKIIDNRRPTRSSTLER--------   [1205]
Fugu 3                                    ------------------------------------------------------------   [845]
Ciona 3 (0100146117)                      ------------------------------------------------------------   [997]
Homo ADAMTS2 (7656867)                    ------------------------------------------------------------   [1211]
Mus 2 (28204840)                          ------------------------------------------------------------   [1213]
Fugu 2                                    ------------------------------------------------------------   [799]
Homo ADAMTS13 (16306598)                  G-TWMECS-VSCGDGIQRRRDTCLGPQAQ-APVPADFCQHLPKPVTVRGCWAGPCVGQGT   [1135]
Fugu 13                                   K-PWSQCS-VSCGYGIQSRAVSCMGPSHP-QPLSPMLCMHMPKPITIQSCSTGSCSEERP   [1057]
Homo ADAMTS1 (6525075)                    ------------------------------------------------------------   [967]
Mus 1 (1813340)                           ------------------------------------------------------------   [951]
Homo ADAMTS4 (12643637)                   ------------------------------------------------------------   [837]
Mus 4 (26350615)                          ------------------------------------------------------------   [845]
Fugu 4                                    ------------------------------------------------------------   [695]
Homo ADAMTS8 (21536392)                   ------------------------------------------------------------   [890]
Mus 8 (6708110)                           ------------------------------------------------------------   [905]
Fugu 8                                    ------------------------------------------------------------   [867]
Homo ADAMTS5 (7768707)                    ------------------------------------------------------------   [930]
Mus 5 (5923784)                           ------------------------------------------------------------   [930]
Fugu 5                                    ------------------------------------------------------------   [921]
Ciona 15 (0100137065)                     ------------------------------------------------------------   [987]
Homo ADAMTS15 (19171175)                  ------------------------------------------------------------   [950]
Fugu 15                                   ------------------------------------------------------------   [891]
Homo ADAMTS9 (27463365)                   ----SSCS-VTCGQGRATRQVMCVNYSDH-VIDRSECDQDYIPETDQDCSMSPCPQRTPD   [1266]
Fugu 9                                    ----TACS-VTCGQGKTTRQVLCVNFSDQ-EVNASECDPDDRPATEQDCAMSQCPSRSSE   [1111]
Homo ADAMTS20 (28460690)                  ----SPCS-ASCGHGKTTRQVLCMNYHQP-IDENY-CDPEVRPLMEQECSLAACPPAHSH   [1220]
Mus 20 (29500513)                         AHDEVADESNCAHLPRPAAVSLCFSPCGEWQAGYWSPCSASCGHGKTTRRVLCVNYHQLV   [1237]
Fugu 20                                   YHQSVDPSFCDPDEKPATEQECIAAPCPSVYRRQRINDQPY-------------------   [1209]
Ciona 9 (0100132719)                      IRSDVVAPEEECGSSESRPAARETCEDSPCHGTWRTGVWTECSVSCDGGTRTRYVRCLYN   [1380]
Apis 9                                    EKKPSTEIPCNRFHCPIWITSDWGQFGSNENVLQCSKSCGTGLQRRRVECTMRRGNHGPE   [1150]
Drosophila CG6107 (20151419)              C-VRRQSR-VISQCNCEGVEKRRDFCFNS-HKGRIACPTRARVER-HSCTPPPHCRRRSA   [1461]
Caenorhabditis gon-1 CEt13h10 (3879882)   T-DWSACS-VSCGIGHRERTTECIYREQS-VDASF-CGDTKMPETSQTCHLLPCTS----   [1298]
introns                                   ------------------------------------------------------------   [2]
```

```
[                                                   1930      1940      1950      1960      1970      1980]
[                                                      .         .         .         .         .         .]

mask                                           ------------------------------------------------------------   [571]
Homo ADAMTS6 (11131380)                        ------------------------------------------------------------   [1117]
Fugu 6                                         ------------------------------------------------------------   [1121]
Ciona 6 (0100146470)                           ------------------------------------------------------------   [1141]
Homo ADAMTS10 (17432918)                       ------------------------------------------------------------   [1103]
Mus 10 (27502095)                              ------------------------------------------------------------   [1070]
Fugu 10                                        ------------------------------------------------------------   [1058]
Homo ADAMTS16 (21265061)                       ------------------------------------------------------------   [1182]
Mus 16 (21961374)                              ------------------------------------------------------------   [900]
Fugu 16                                        ------------------------------------------------------------   [1097]
Ciona 16 (0100138085)                          ------------------------------------------------------------   [1076]
Homo ADAMTS18 (21265067)                       ------------------------------------------------------------   [1178]
Fugu 18                                        ------------------------------------------------------------   [742]
Homo ADAMTS12 (13569928)                       SEPVLTEEDATSLITEGFLLNASN-------YKQLTNGHGSAHWI--VGNWSECSTTCGL   [1330]
Mus 12 (27817773)                              FGPVLTEEDASNLIAEGFLLNASD-------YKHLMKDHSPAYWI--VGNWSKCSTTCGL   [1336]
Fugu 12                                        ------------------------------------------------------------   [1061]
Homo ADAMTS7 (11131377)                        VAELWTGGTVAWEPALEGGLGPVD-------SELWPTVGVASLLP--PPIAPLPEMKVRD   [1307]
Fugu 7                                         ------------------------------------------------------------   [930]
Ciona 7 (genewise.34.12.1)                     ------------------------------------------------------------   [965]
Homo ADAMTS17 (21265064)                       ------------------------------------------------------------   [1122]
Homo ADAMTS19 (19525737)                       ------------------------------------------------------------   [1150]
Drosophila dCG4096 (22831757)                  ------------------------------------------------------------   [1054]
Homo ADAMTS14 (29337086)                       ------------------------------------------------------------   [1115]
Fugu 14                                        ------------------------------------------------------------   [991]
Homo ADAMTS3 (2224673)                         ------------------------------------------------------------   [1205]
Fugu 3                                         ------------------------------------------------------------   [845]
Ciona 3 (0100146117)                           ------------------------------------------------------------   [997]
Homo ADAMTS2 (7656867)                         ------------------------------------------------------------   [1211]
Mus 2 (28204840)                               ------------------------------------------------------------   [1213]
Fugu 2                                         ------------------------------------------------------------   [799]
Homo ADAMTS13 (16306598)                       PSLVPHEEAAAPGRTTATPAGASL-------EWSQARGLLFSPAP--QPRRLLPGPQENS   [1186]
Fugu 13                                        SHAGTPLQPTARTAPPGPTEASTV-------LQNLTTPIPVTKMS--QCPESTFGNRTVL   [1108]
Homo ADAMTS1 (6525075)                          ------------------------------------------------------------   [967]
Mus 1 (1813340)                                ------------------------------------------------------------   [951]
Homo ADAMTS4 (12643637)                         ------------------------------------------------------------   [837]
Mus 4 (26350615)                               ------------------------------------------------------------   [845]
Fugu 4                                         ------------------------------------------------------------   [695]
Homo ADAMTS8 (21536392)                        ------------------------------------------------------------   [890]
Mus 8 (6708110)                                ------------------------------------------------------------   [905]
Fugu 8                                         ------------------------------------------------------------   [867]
Homo ADAMTS5 (7768707)                         ------------------------------------------------------------   [930]
Mus 5 (5923784)                                ------------------------------------------------------------   [930]
Fugu 5                                         ------------------------------------------------------------   [921]
Ciona 15 (0100137065)                          ------------------------------------------------------------   [987]
Homo ADAMTS15 (19171175)                       ------------------------------------------------------------   [950]
Fugu 15                                        ------------------------------------------------------------   [891]
Homo ADAMTS9 (27463365)                        SGLAQHPFQNEDYRPRSAS---------------PSRTHVLGGNQ--WRTGPWGACSSTC   [1309]
Fugu 9                                         PRPFPSSPNTSARNNLPHN-----------------------------------------   [1130]
Homo ADAMTS20 (28460690)                       FPSSPVQPSYYLSTNLPLTQKLED-------NENQVVHPSVRGNQ--WRTGPWGSCSSSC   [1271]
Mus 20 (29500513)                              DESYCDPEGRPVTEQECSLAACPPLYSRAPSSSEQPSHVPSRNVPLTHKPGENQDQGAQL   [1297]
Fugu 20                                        ------------------------------------------------------------   [1209]
Ciona 9 (0100132719)                           GRYQDDGQHCLPQDKPSEDEICSSAPCPTTTTTTTSTTTTTSTTTSTTTSATTTTATTTT   [1440]
Apis 9                                         VTVRDEHCSRLGLRKPRSQRPCRRIACNYIWQEGPWSEKIVTHQQ---------------   [1195]
Drosophila CG6107 (20151419)                   IGSSISSRPRGTGVSSSRSLNSIG-------GSRNRGTPRSCADL--KEMHGYNKDGNYQ   [1512]
Caenorhabditis gon-1 CEt13h10 (3879882)        -----------------------------------------------WKPSHWSPCSVTC   [1311]
introns                                        ------------------------------------------------------------   [2]
```

```
[                                                 1990      2000      2010      2020      2030      2040]
[                                                    .         .         .         .         .         .]

mask                                          ------------------------------------------------------------   [571]
Homo ADAMTS6 (11131380)                       ------------------------------------------------------------   [1117]
Fugu 6                                        ------------------------------------------------------------   [1121]
Ciona 6 (0100146470)                          ------------------------------------------------------------   [1141]
Homo ADAMTS10 (17432918)                      ------------------------------------------------------------   [1103]
Mus 10 (27502095)                             ------------------------------------------------------------   [1070]
Fugu 10                                       ------------------------------------------------------------   [1058]
Homo ADAMTS16 (21265061)                      ------------------------------------------------------------   [1182]
Mus 16 (21961374)                             ------------------------------------------------------------   [900]
Fugu 16                                       ------------------------------------------------------------   [1097]
Ciona 16 (0100138085)                         ------------------------------------------------------------   [1076]
Homo ADAMTS18 (21265067)                      ------------------------------------------------------------   [1178]
Fugu 18                                       ------------------------------------------------------------   [742]
Homo ADAMTS12 (13569928)                      GAYWKRVECTTQMDSDCAAIQRPDPAKRCHLRPCAGWKVGNWSKCSRNCSGGFKIREIQC   [1390]
Mus 12 (27817773)                             GAYWRSVECSSGVDADCTTIQRPDPAKKCHLRPCAGWRVGNWSKCSRNCSGGFKIREVQC   [1396]
Fugu 12                                       ------------------------------------------------------------   [1061]
Homo ADAMTS7 (11131377)                       SSLEPGTPSFPAPGPGSWDLQTVAVWGTFLPTTLTGLGHMPEPALNPGPKGQPESLSPEV   [1367]
Fugu 7                                        ------------------------------------------------------------   [930]
Ciona 7 (genewise.34.12.1)                    ------------------------------------------------------------   [965]
Homo ADAMTS17 (21265064)                      ------------------------------------------------------------   [1122]
Homo ADAMTS19 (19525737)                      ------------------------------------------------------------   [1150]
Drosophila dCG4096 (22831757)                 ------------------------------------------------------------   [1054]
Homo ADAMTS14 (29337086)                      ------------------------------------------------------------   [1115]
Fugu 14                                       ------------------------------------------------------------   [991]
Homo ADAMTS3 (2224673)                        ------------------------------------------------------------   [1205]
Fugu 3                                        ------------------------------------------------------------   [845]
Ciona 3 (0100146117)                          ------------------------------------------------------------   [997]
Homo ADAMTS2 (7656867)                        ------------------------------------------------------------   [1211]
Mus 2 (28204840)                              ------------------------------------------------------------   [1213]
Fugu 2                                        ------------------------------------------------------------   [799]
Homo ADAMTS13 (16306598)                      VQSSACGRQHLEPTGTIDMRGPGQADCAVAIGRPLGEVVTLRVLESSLNCSAGDMLLLWG   [1246]
Fugu 13                                       LRQNRFILLALGLTRSMWAPSPAAIRHCGPEG----------------------------   [1140]
Homo ADAMTS1 (6525075)                        ------------------------------------------------------------   [967]
Mus 1 (1813340)                               ------------------------------------------------------------   [951]
Homo ADAMTS4 (12643637)                       ------------------------------------------------------------   [837]
Mus 4 (26350615)                              ------------------------------------------------------------   [845]
Fugu 4                                        ------------------------------------------------------------   [695]
Homo ADAMTS8 (21536392)                       ------------------------------------------------------------   [890]
Mus 8 (6708110)                               ------------------------------------------------------------   [905]
Fugu 8                                        ------------------------------------------------------------   [867]
Homo ADAMTS5 (7768707)                        ------------------------------------------------------------   [930]
Mus 5 (5923784)                               ------------------------------------------------------------   [930]
Fugu 5                                        ------------------------------------------------------------   [921]
Ciona 15 (0100137065)                         ------------------------------------------------------------   [987]
Homo ADAMTS15 (19171175)                      ------------------------------------------------------------   [950]
Fugu 15                                       ------------------------------------------------------------   [891]
Homo ADAMTS9 (27463365)                       AGGSQRRVVVCQDENGYTAND---CVERIKPDEQRACESGPCPQ---------------W   [1351]
Fugu 9                                        -------------------------------------------------------QSHQW   [1135]
Homo ADAMTS20 (28460690)                       SGGLQHRAVVCQDENGQSASY---CDAASKPPELQQCGPGPCPQ---------------W   [1313]
Mus 20 (29500513)                             SIRGNQWRTGPWGACSRSCAGGLQHRAVVCQDEDGRSATSCDGSSKPPESRHCGSGPCPH   [1357]
Fugu 20                                       ------------------------------------------------------------   [1209]
Ciona 9 (0100132719)                          ATTTNEPTTIYESNRTRLGHWRTGPWSQCSSTCGGGTQKRAVVCYSPHSAKADCDSLDPP   [1500]
Apis 9                                        ------------------------------------------------------------   [1195]
Drosophila CG6107 (20151419)                  LEVRSRMVHIYCHGMNSRTPQEYVNVDPQENYSIYYEYRTKQTNSCPPESRGHEYYNDQN   [1572]
Caenorhabditis gon-1 CEt13h10 (3879882)       GSGIQTRSVSCTRGSEGTIVDEYFCDRNTRPRLKKTCEKDTCDGPRVLQKLQADVPPIRW   [1371]
introns                                       ------------------------------------------------------------   [2]
```

```
[                                                   2050      2060      2070      2080      2090      2100]
[                                                   .         .         .         .         .         .]

mask                                          ------------------------------------------------------------   [571]
Homo ADAMTS6 (11131380)                       ------------------------------------------------------------   [1117]
Fugu 6                                        ------------------------------------------------------------   [1121]
Ciona 6 (0100146470)                          ------------------------------------------------------------   [1141]
Homo ADAMTS10 (17432918)                      ------------------------------------------------------------   [1103]
Mus 10 (27502095)                             ------------------------------------------------------------   [1070]
Fugu 10                                       ------------------------------------------------------------   [1058]
Homo ADAMTS16 (21265061)                      ------------------------------------------------------------   [1182]
Mus 16 (21961374)                             ------------------------------------------------------------   [900]
Fugu 16                                       ------------------------------------------------------------   [1097]
Ciona 16 (0100138085)                         ------------------------------------------------------------   [1076]
Homo ADAMTS18 (21265067)                      ------------------------------------------------------------   [1178]
Fugu 18                                       ------------------------------------------------------------   [742]
Homo ADAMTS12 (13569928)                      VDSRD-HRNLRPFHCQFLAGIPPPLSMSCNPEPCEAWQV-EPWSQCSRSCGGGVQERGVF   [1448]
Mus 12 (27817773)                             MDSLDHHRSLRPFHCQFLAGAPPPLSMSCNLEPCGEWQV-EPWSQCSRSCGGGVQERGVS   [1455]
Fugu 12                                       ------------------------------------------------------------   [1061]
Homo ADAMTS7 (11131377)                       PLSSRLLSTPAWDSPANSHRVPETQPLAPSLAEAGPPAD-PLVVRNASWQAGNWSECSTT   [1426]
Fugu 7                                        ------------------------------------------------------------   [930]
Ciona 7 (genewise.34.12.1)                    ------------------------------------------------------------   [965]
Homo ADAMTS17 (21265064)                      ------------------------------------------------------------   [1122]
Homo ADAMTS19 (19525737)                      ------------------------------------------------------------   [1150]
Drosophila dCG4096 (22831757)                 ------------------------------------------------------------   [1054]
Homo ADAMTS14 (29337086)                      ------------------------------------------------------------   [1115]
Fugu 14                                       ------------------------------------------------------------   [991]
Homo ADAMTS3 (2224673)                        ------------------------------------------------------------   [1205]
Fugu 3                                        ------------------------------------------------------------   [845]
Ciona 3 (0100146117)                          ------------------------------------------------------------   [997]
Homo ADAMTS2 (7656867)                        ------------------------------------------------------------   [1211]
Mus 2 (28204840)                              ------------------------------------------------------------   [1213]
Fugu 2                                        ------------------------------------------------------------   [799]
Homo ADAMTS13 (16306598)                      RLTWRKMCRKLLDMTFSSKTNTLVVRQRCGRPGGGVLLR-YGSQLAPETFYRECDMQLFG   [1305]
Fugu 13                                       ------------------------------------------------------------   [1140]
Homo ADAMTS1 (6525075)                        ------------------------------------------------------------   [967]
Mus 1 (1813340)                               ------------------------------------------------------------   [951]
Homo ADAMTS4 (12643637)                       ------------------------------------------------------------   [837]
Mus 4 (26350615)                              ------------------------------------------------------------   [845]
Fugu 4                                        ------------------------------------------------------------   [695]
Homo ADAMTS8 (21536392)                       ------------------------------------------------------------   [890]
Mus 8 (6708110)                               ------------------------------------------------------------   [905]
Fugu 8                                        ------------------------------------------------------------   [867]
Homo ADAMTS5 (7768707)                        ------------------------------------------------------------   [930]
Mus 5 (5923784)                               ------------------------------------------------------------   [930]
Fugu 5                                        ------------------------------------------------------------   [921]
Ciona 15 (0100137065)                         ------------------------------------------------------------   [987]
Homo ADAMTS15 (19171175)                      ------------------------------------------------------------   [950]
Fugu 15                                       ------------------------------------------------------------   [891]
Homo ADAMTS9 (27463365)                       AYGNWGECTKLCGGGIRTRLVVCQRSNGERFPDLSCEIL-DKPPDREQCNTHACPHDAAW   [1410]
Fugu 9                                        RTGPWGACTKPCGGGIKTRLVVCQRPNGERFNDLSCEIH-DKPPDREQCNTQPCPSNPHW   [1194]
Homo ADAMTS20 (28460690)                      NYGNWGECSQTCGGGIKSRLVICQFPNGQILEDHNCEIV-NKPPSVIQCHMHACPADVSW   [1372]
Mus 20 (29500513)                             WNYGDWGECTQTCGGGVKSRFVICQFPNGQMTQEHSCELPKPPSMMQCHLHACPEDVSWY   [1417]
Fugu 20                                       ------------------------------------------------------------   [1209]
Ciona 9 (0100132719)                          PSIQLCNADACPRWKRGRWGKCSKTCNGGRRLRNVYCVMSDGRSTYKHRCNVILKPVEIE   [1560]
Apis 9                                        ------------------------------------------------------------   [1195]
Drosophila CG6107 (20151419)                  SGRTHFRKLRLNITDLRIMDNDFKFADSRGLAQKLGSAG-DCYNRIGQCPQGDFSINMKD   [1631]
Caenorhabditis gon-1 CEt13h10 (3879882)       ATGPWTACSATCGNGTQRRLLKCRDHVRDLPDEY-CNHL-DKEVSTRNCRLRDCSY---W   [1426]
introns                                       ------------------------------------------------------------   [2]
```

```
[                                                   2110      2120      2130      2140      2150      2160]
[                                                      .         .         .         .         .         .]

mask                                          ------------------------------------------------------------   [571]
Homo ADAMTS6 (11131380)                       ------------------------------------------------------------   [1117]
Fugu 6                                        ------------------------------------------------------------   [1121]
Ciona 6 (0100146470)                          ------------------------------------------------------------   [1141]
Homo ADAMTS10 (17432918)                      ------------------------------------------------------------   [1103]
Mus 10 (27502095)                             ------------------------------------------------------------   [1070]
Fugu 10                                       ------------------------------------------------------------   [1058]
Homo ADAMTS16 (21265061)                      ------------------------------------------------------------   [1182]
Mus 16 (21961374)                             ------------------------------------------------------------   [900]
Fugu 16                                       ------------------------------------------------------------   [1097]
Ciona 16 (0100138085)                         ------------------------------------------------------------   [1076]
Homo ADAMTS18 (21265067)                      ------------------------------------------------------------   [1178]
Fugu 18                                       ------------------------------------------------------------   [742]
Homo ADAMTS12 (13569928)                      CPGGLCDWTKRPTSTMSCNEHLCCH-----------------------------------   [1473]
Mus 12 (27817773)                             CPGGLCDWTKRPATTVPCNRHLCCH-----------------------------------   [1480]
Fugu 12                                       ------------------------------------------------------------   [1061]
Homo ADAMTS7 (11131377)                       CGLGAVWRPVRCSSGRDEDCAPAGRPQPARRCHLRPCAT---------------------   [1465]
Fugu 7                                        ------------------------------------------------------------   [930]
Ciona 7 (genewise.34.12.1)                    ------------------------------------------------------------   [965]
Homo ADAMTS17 (21265064)                      ------------------------------------------------------------   [1122]
Homo ADAMTS19 (19525737)                      ------------------------------------------------------------   [1150]
Drosophila dCG4096 (22831757)                 ------------------------------------------------------------   [1054]
Homo ADAMTS14 (29337086)                      ------------------------------------------------------------   [1115]
Fugu 14                                       ------------------------------------------------------------   [991]
Homo ADAMTS3 (2224673)                        ------------------------------------------------------------   [1205]
Fugu 3                                        ------------------------------------------------------------   [845]
Ciona 3 (0100146117)                          ------------------------------------------------------------   [997]
Homo ADAMTS2 (7656867)                        ------------------------------------------------------------   [1211]
Mus 2 (28204840)                              ------------------------------------------------------------   [1213]
Fugu 2                                        ------------------------------------------------------------   [799]
Homo ADAMTS13 (16306598)                      PWGEIVSPSLSPATSNAGGCRLFINVAPHARIAIHALATNMGAGTEGANASY--ILIRDT   [1363]
Fugu 13                                       ------------------------------------------------------------   [1140]
Homo ADAMTS1 (6525075)                        ------------------------------------------------------------   [967]
Mus 1 (1813340)                               ------------------------------------------------------------   [951]
Homo ADAMTS4 (12643637)                       ------------------------------------------------------------   [837]
Mus 4 (26350615)                              ------------------------------------------------------------   [845]
Fugu 4                                        ------------------------------------------------------------   [695]
Homo ADAMTS8 (21536392)                       ------------------------------------------------------------   [890]
Mus 8 (6708110)                               ------------------------------------------------------------   [905]
Fugu 8                                        ------------------------------------------------------------   [867]
Homo ADAMTS5 (7768707)                        ------------------------------------------------------------   [930]
Mus 5 (5923784)                               ------------------------------------------------------------   [930]
Fugu 5                                        ------------------------------------------------------------   [921]
Ciona 15 (0100137065)                         ------------------------------------------------------------   [987]
Homo ADAMTS15 (19171175)                      ------------------------------------------------------------   [950]
Fugu 15                                       ------------------------------------------------------------   [891]
Homo ADAMTS9 (27463365)                       STGPWSSCSVSCGRGHKQRNVY-CMAKDGSHLESDY--CKHLA-KPHGHRKC--RGGRCP   [1464]
Fugu 9                                        SADTWSSCSASCGRGFRSRTVS-CVAASGLVISEEN--CQGLSPKPSRQRRC--RGGRCP   [1249]
Homo ADAMTS20 (28460690)                      HQEPWTSCSASCGKGRKYREVF-CIDQFQRKLEDTN--CSQVQ-KPPTHKAC--RSVRCP   [1426]
Mus 20 (29500513)                             RGPWKSCSASCGKGVKYREVLCIDQFQRKLEEKYCSHLHKPRTHKACRSGRCPSWKANKW   [1477]
Fugu 20                                       ------------------------------------------------------------   [1209]
Ciona 9 (0100132719)                          PCRTMTCPGAHRWRKSAWSACSASCGRGTKNRNVNCLNANGETVADAKCLLHRTKPRVVR   [1620]
Apis 9                                        ------------------------------------------------------------   [1195]
Drosophila CG6107 (20151419)                  TDFSIRPGTVWRMHGQYSVMKRISEFDTTTQMRRGFCGGYCGGCYIAPDSGL--YLDVL-   [1688]
Caenorhabditis gon-1 CEt13h10 (3879882)       KMAEWEECPATCGTHVQQSRNVTCVSAEDGGRTILKDVDCDVQKRPTSARNC--RLEPCP   [1484]
introns                                       ------------------------------------------------------------   [2]
```

```
[                                                  2170      2180      2190      2200      2210      2220]
[                                                     .         .         .         .         .         .]

mask                                          ------------------------------------------------------------  [571]
Homo ADAMTS6 (11131380)                       ------------------------------------------------------------  [1117]
Fugu 6                                        ------------------------------------------------------------  [1121]
Ciona 6 (0100146470)                          ------------------------------------------------------------  [1141]
Homo ADAMTS10 (17432918)                      ------------------------------------------------------------  [1103]
Mus 10 (27502095)                             ------------------------------------------------------------  [1070]
Fugu 10                                       ------------------------------------------------------------  [1058]
Homo ADAMTS16 (21265061)                      ------------------------------------------------------------  [1182]
Mus 16 (21961374)                             ------------------------------------------------------------  [900]
Fugu 16                                       ------------------------------------------------------------  [1097]
Ciona 16 (0100138085)                         ------------------------------------------------------------  [1076]
Homo ADAMTS18 (21265067)                      ------------------------------------------------------------  [1178]
Fugu 18                                       ------------------------------------------------------------  [742]
Homo ADAMTS12 (13569928)                      --------WATGNWDLCSTSCGGGFQKRIVQCVPS---EGNK---TEDQDQCLCDHKPRP  [1519]
Mus 12 (27817773)                             --------WATGNWELCNTSCGGGSQKRTIHCIPS---ENST---TEDQDQCLCDHQVKP  [1526]
Fugu 12                                       ------------------------------------------------------------  [1061]
Homo ADAMTS7 (11131377)                       --------WHSGNWSKCSRSCGGGSSVRDVQCVDT---RDLR---PLRPFHCQPG-PAKP  [1510]
Fugu 7                                        ------------------------------------------------------------  [930]
Ciona 7 (genewise.34.12.1)                    ------------------------------------------------------------  [965]
Homo ADAMTS17 (21265064)                      ------------------------------------------------------------  [1122]
Homo ADAMTS19 (19525737)                      ------------------------------------------------------------  [1150]
Drosophila dCG4096 (22831757)                 ------------------------------------------------------------  [1054]
Homo ADAMTS14 (29337086)                      ------------------------------------------------------------  [1115]
Fugu 14                                       ------------------------------------------------------------  [991]
Homo ADAMTS3 (2224673)                        ------------------------------------------------------------  [1205]
Fugu 3                                        ------------------------------------------------------------  [845]
Ciona 3 (0100146117)                          ------------------------------------------------------------  [997]
Homo ADAMTS2 (7656867)                        ------------------------------------------------------------  [1211]
Mus 2 (28204840)                              ------------------------------------------------------------  [1213]
Fugu 2                                        ------------------------------------------------------------  [799]
Homo ADAMTS13 (16306598)                      HSLRTTAFHGQQVLYWESESSQAEMEFSEGFLKAQ---ASLR---GQYWTLQSWVPEMQD  [1417]
Fugu 13                                       ------------------------------------------------------------  [1140]
Homo ADAMTS1 (6525075)                        ------------------------------------------------------------  [967]
Mus 1 (1813340)                               ------------------------------------------------------------  [951]
Homo ADAMTS4 (12643637)                       ------------------------------------------------------------  [837]
Mus 4 (26350615)                              ------------------------------------------------------------  [845]
Fugu 4                                        ------------------------------------------------------------  [695]
Homo ADAMTS8 (21536392)                       ------------------------------------------------------------  [890]
Mus 8 (6708110)                               ------------------------------------------------------------  [905]
Fugu 8                                        ------------------------------------------------------------  [867]
Homo ADAMTS5 (7768707)                        ------------------------------------------------------------  [930]
Mus 5 (5923784)                               ------------------------------------------------------------  [930]
Fugu 5                                        ------------------------------------------------------------  [921]
Ciona 15 (0100137065)                         ------------------------------------------------------------  [987]
Homo ADAMTS15 (19171175)                      ------------------------------------------------------------  [950]
Fugu 15                                       ------------------------------------------------------------  [891]
Homo ADAMTS9 (27463365)                       K-------WKAGAWSQCSVSCGRGVQQRHVGCQIG---THKI---ARETECNPYTRPESE  [1511]
Fugu 9                                        -------KWKTGNWGECSASCGDGVQQREVFCQVG---DQRI---PEESGCSRRSRPPSS  [1296]
Homo ADAMTS20 (28460690)                       S-------WKANSWNECSVTCGSGVQQRDVYCRLK---GVGQ---VVEEMCDQSTRPCSQ  [1473]
Mus 20 (29500513)                             KECSVTCGSGVQQREVYCRLRGTGRVSEDMCDPSTRP-QGQR---QCWRQDCMRYQWTTG  [1533]
Fugu 20                                       ------------------------------------------------------------  [1209]
Ciona 9 (0100132719)                          RCVKQRRCPRWRTGRWNQCSASCGVGIQLRRVNCRYK-RKVI---VDEL-CDVNSRPDDV  [1675]
Apis 9                                        ------------------------------------------------------------  [1195]
Drosophila CG6107 (20151419)                  ------------------------------------------------------------  [1688]
Caenorhabditis gon-1 CEt13h10 (3879882)       KGEEHIGSWIIGDWSKCSASCGGGWRRRSVSCTSS---SCDE---TRKPKMFDKCNEELC  [1538]
introns                                       ------------------------------------------------------------  [2]
```

```
[                                                      2230      2240      2250      2260      2270      2280]
[                                                         .         .         .         .         .         .]

mask                                          ------------------------------------------------------------   [571]
Homo ADAMTS6 (11131380)                       ------------------------------------------------------------   [1117]
Fugu 6                                        ------------------------------------------------------------   [1121]
Ciona 6 (0100146470)                          ------------------------------------------------------------   [1141]
Homo ADAMTS10 (17432918)                      ------------------------------------------------------------   [1103]
Mus 10 (27502095)                             ------------------------------------------------------------   [1070]
Fugu 10                                       ------------------------------------------------------------   [1058]
Homo ADAMTS16 (21265061)                      ------------------------------------------------------------   [1182]
Mus 16 (21961374)                             ------------------------------------------------------------   [900]
Fugu 16                                       ------------------------------------------------------------   [1097]
Ciona 16 (0100138085)                         ------------------------------------------------------------   [1076]
Homo ADAMTS18 (21265067)                      ------------------------------------------------------------   [1178]
Fugu 18                                       ------------------------------------------------------------   [742]
Homo ADAMTS12 (13569928)                      PEFKKC-NQQACKKSADLLCTKDKLSASFCQTLKAMKKCSVPTVRAECCFSCPQTHITHT   [1578]
Mus 12 (27817773)                             PEFQTC-NQQACRKSADLTCLKDRLSISFCQTLKSMRKCSVPSVRAQCCLSCPQAPSIHT   [1585]
Fugu 12                                       ------------------------------------------------------------   [1061]
Homo ADAMTS7 (11131377)                       PAHRPC-GAQPCLSWYTSSWRECSEACGGGEQQRLVTCPEPGLCEEALRPNTTRPCNTHP   [1569]
Fugu 7                                        ------------------------------------------------------------   [930]
Ciona 7 (genewise.34.12.1)                    ------------------------------------------------------------   [965]
Homo ADAMTS17 (21265064)                      ------------------------------------------------------------   [1122]
Homo ADAMTS19 (19525737)                      ------------------------------------------------------------   [1150]
Drosophila dCG4096 (22831757)                 ------------------------------------------------------------   [1054]
Homo ADAMTS14 (29337086)                      ------------------------------------------------------------   [1115]
Fugu 14                                       ------------------------------------------------------------   [991]
Homo ADAMTS3 (2224673)                        ------------------------------------------------------------   [1205]
Fugu 3                                        ------------------------------------------------------------   [845]
Ciona 3 (0100146117)                          ------------------------------------------------------------   [997]
Homo ADAMTS2 (7656867)                        ------------------------------------------------------------   [1211]
Mus 2 (28204840)                              ------------------------------------------------------------   [1213]
Fugu 2                                        ------------------------------------------------------------   [799]
Homo ADAMTS13 (16306598)                      PQSWKG-KEGT-------------------------------------------------   [1427]
Fugu 13                                       ------------------------------------------------------------   [1140]
Homo ADAMTS1 (6525075)                        ------------------------------------------------------------   [967]
Mus 1 (1813340)                               ------------------------------------------------------------   [951]
Homo ADAMTS4 (12643637)                       ------------------------------------------------------------   [837]
Mus 4 (26350615)                              ------------------------------------------------------------   [845]
Fugu 4                                        ------------------------------------------------------------   [695]
Homo ADAMTS8 (21536392)                       ------------------------------------------------------------   [890]
Mus 8 (6708110)                               ------------------------------------------------------------   [905]
Fugu 8                                        ------------------------------------------------------------   [867]
Homo ADAMTS5 (7768707)                        ------------------------------------------------------------   [930]
Mus 5 (5923784)                               ------------------------------------------------------------   [930]
Fugu 5                                        ------------------------------------------------------------   [921]
Ciona 15 (0100137065)                         ------------------------------------------------------------   [987]
Homo ADAMTS15 (19171175)                      ------------------------------------------------------------   [950]
Fugu 15                                       ------------------------------------------------------------   [891]
Homo ADAMTS9 (27463365)                       RDCQGP-RCPLYTWRAEEWQECTKTCGEGSRYRKVVCVDDNKNEVHGARCDVSKRPVDRE   [1570]
Fugu 9                                        QSCRVAGCPSRYRWREADWQRCSKTCGGGHRRRALKCEDHNRQEIHEMYCANLIRPPDVE   [1356]
Homo ADAMTS20 (28460690)                      RRC----------WSQDCVQHKGMERGRLNCANNCGFSYRQRITYCTEIPSTKKHKLHRL   [1523]
Mus 20 (29500513)                             DWLDCSTSCKKKETYRLVKCVNEQNVQANESLCDPLTKPLSIKKCRNPHCKYSVVTGDSS   [1593]
Fugu 20                                       ------------------------------------------------------------   [1209]
Ciona 9 (0100132719)                          TRCNVT----------DCPSHKWHAERWRPCSVSCGEGTMTRVVTCRSLPHNIVVDPSLC   [1725]
Apis 9                                        ------------------------------------------------------------   [1195]
Drosophila CG6107 (20151419)                  ------------------------------------------------------------   [1688]
Caenorhabditis gon-1 CEt13h10 (3879882)       PPLTNN-S-----WQISPWTHCSVSCGGGVQRRKIWCEDVLSGRKQDDIECSEIKPREQR   [1592]
introns                                       ------------------------------------------------------------   [2]
```

```
[                                                 2290      2300      2310      2320      2330      2340]
[                                                 .         .         .         .         .         .]

mask                                          ------------------------------------------------------------   [571]
Homo ADAMTS6 (11131380)                       ------------------------------------------------------------   [1117]
Fugu 6                                        ------------------------------------------------------------   [1121]
Ciona 6 (0100146470)                          ------------------------------------------------------------   [1141]
Homo ADAMTS10 (17432918)                      ------------------------------------------------------------   [1103]
Mus 10 (27502095)                             ------------------------------------------------------------   [1070]
Fugu 10                                       ------------------------------------------------------------   [1058]
Homo ADAMTS16 (21265061)                      ------------------------------------------------------------   [1182]
Mus 16 (21961374)                             ------------------------------------------------------------   [900]
Fugu 16                                       ------------------------------------------------------------   [1097]
Ciona 16 (0100138085)                         ------------------------------------------------------------   [1076]
Homo ADAMTS18 (21265067)                      ------------------------------------------------------------   [1178]
Fugu 18                                       ------------------------------------------------------------   [742]
Homo ADAMTS12 (13569928)                      QRQRRQRLLQKSKEL---------------------------------------------   [1593]
Mus 12 (27817773)                             QRQRKQQLLQNHDML---------------------------------------------   [1600]
Fugu 12                                       ------------------------------------------------------------   [1061]
Homo ADAMTS7 (11131377)                       CTQ-------WVVGPWGQCSAPCGGGVQRRLVKCVNTQTGLPEEDSDQCGHEAWPESSRP   [1622]
Fugu 7                                        ------------------------------------------------------------   [930]
Ciona 7 (genewise.34.12.1)                    ------------------------------------------------------------   [965]
Homo ADAMTS17 (21265064)                      ------------------------------------------------------------   [1122]
Homo ADAMTS19 (19525737)                      ------------------------------------------------------------   [1150]
Drosophila dCG4096 (22831757)                 ------------------------------------------------------------   [1054]
Homo ADAMTS14 (29337086)                      ------------------------------------------------------------   [1115]
Fugu 14                                       ------------------------------------------------------------   [991]
Homo ADAMTS3 (2224673)                        ------------------------------------------------------------   [1205]
Fugu 3                                        ------------------------------------------------------------   [845]
Ciona 3 (0100146117)                          ------------------------------------------------------------   [997]
Homo ADAMTS2 (7656867)                        ------------------------------------------------------------   [1211]
Mus 2 (28204840)                              ------------------------------------------------------------   [1213]
Fugu 2                                        ------------------------------------------------------------   [799]
Homo ADAMTS13 (16306598)                      ------------------------------------------------------------   [1427]
Fugu 13                                       ------------------------------------------------------------   [1140]
Homo ADAMTS1 (6525075)                        ------------------------------------------------------------   [967]
Mus 1 (1813340)                               ------------------------------------------------------------   [951]
Homo ADAMTS4 (12643637)                       ------------------------------------------------------------   [837]
Mus 4 (26350615)                              ------------------------------------------------------------   [845]
Fugu 4                                        ------------------------------------------------------------   [695]
Homo ADAMTS8 (21536392)                       ------------------------------------------------------------   [890]
Mus 8 (6708110)                               ------------------------------------------------------------   [905]
Fugu 8                                        ------------------------------------------------------------   [867]
Homo ADAMTS5 (7768707)                        ------------------------------------------------------------   [930]
Mus 5 (5923784)                               ------------------------------------------------------------   [930]
Fugu 5                                        ------------------------------------------------------------   [921]
Ciona 15 (0100137065)                         ------------------------------------------------------------   [987]
Homo ADAMTS15 (19171175)                      ------------------------------------------------------------   [950]
Fugu 15                                       ------------------------------------------------------------   [891]
Homo ADAMTS9 (27463365)                       SCSLHPCEYVWITGEWSECSVTCGKGYKQRLVSCSEIYTGKENYEYSSQTTINCPGTQPP   [1630]
Fugu 9                                        RCNDHACEVVWITGEWTECSASCGQGYRQRLISCSEVPVEGENYQYGHQSLSNCPGTPPE   [1416]
Homo ADAMTS20 (28460690)                      RPIVYQECPVVPSSQVYQCINSCLHLATWKVGKWSKCSVTCGIGIMKRQVKCITKHGLSS   [1583]
Mus 20 (29500513)                             QCAGNCGFTSPQKITYCTKIQSSKKHTFHQLRPVVYGECPVIPSPQAYKCDLRSCLHVAT   [1653]
Fugu 20                                       ------------------------------------------------------------   [1209]
Ciona 9 (0100132719)                          NTELVTERPLA--SQ--QCNNGPCN-AFWRAGAWSECTKSCGPGIQRRPVKCSYVDPMTY   [1780]
Apis 9                                        ------------------------------------------------------------   [1195]
Drosophila CG6107 (20151419)                  ------------------------------------------------------------   [1688]
Caenorhabditis gon-1 CEt13h10 (3879882)       DCEMPPCRSHYHNKTSSASMTSLSSSNSNTTSSASASSLPILPPVVSWQTSAWSACSAKC   [1652]
introns                                       ------------------------------------------------------------   [2]
```

[	2350 2360 2370 2380 2390 2400]	
[	. . . . . .]	
mask	--	[571]
Homo ADAMTS6 (11131380)	--	[1117]
Fugu 6	--	[1121]
Ciona 6 (0100146470)	--	[1141]
Homo ADAMTS10 (17432918)	--	[1103]
Mus 10 (27502095)	--	[1070]
Fugu 10	--	[1058]
Homo ADAMTS16 (21265061)	---IAEKK-D	[1188]
Mus 16 (21961374)	--	[900]
Fugu 16	--	[1097]
Ciona 16 (0100138085)	--	[1076]
Homo ADAMTS18 (21265067)	---APEKRED	[1185]
Fugu 18	--	[742]
Homo ADAMTS12 (13569928)	--	[1593]
Mus 12 (27817773)	--	[1600]
Fugu 12	--	[1061]
Homo ADAMTS7 (11131377)	CGTEDCEPVEPPRCERDRLSFGFCETLRLLGRCQLPTIRTQCCRSCSPPSHGAPSRGHQR	[1682]
Fugu 7	--	[930]
Ciona 7 (genewise.34.12.1)	--	[965]
Homo ADAMTS17 (21265064)	--	[1122]
Homo ADAMTS19 (19525737)	--TITSPRLAAL	[1160]
Drosophila dCG4096 (22831757)	--	[1054]
Homo ADAMTS14 (29337086)	--	[1115]
Fugu 14	--	[991]
Homo ADAMTS3 (2224673)	--	[1205]
Fugu 3	--	[845]
Ciona 3 (0100146117)	--	[997]
Homo ADAMTS2 (7656867)	--	[1211]
Mus 2 (28204840)	--	[1213]
Fugu 2	--	[799]
Homo ADAMTS13 (16306598)	--	[1427]
Fugu 13	--	[1140]
Homo ADAMTS1 (6525075)	--	[967]
Mus 1 (1813340)	--	[951]
Homo ADAMTS4 (12643637)	--	[837]
Mus 4 (26350615)	--	[845]
Fugu 4	--	[695]
Homo ADAMTS8 (21536392)	--	[890]
Mus 8 (6708110)	--	[905]
Fugu 8	--	[867]
Homo ADAMTS5 (7768707)	--	[930]
Mus 5 (5923784)	--	[930]
Fugu 5	--	[921]
Ciona 15 (0100137065)	--	[987]
Homo ADAMTS15 (19171175)	--	[950]
Fugu 15	--	[891]
Homo ADAMTS9 (27463365)	SVHPCYLRDCPVSATWRVGNWGSCSVSCGVGVMQRSVQCLNNAVQPSHLCRSDLKPEERK	[1690]
Fugu 9	SYMPCHLDPCPSPQAWRVGIWGPCSASCGEGVMERLVQCLAHGQESHGCSLDDKPEARKV	[1476]
Homo ADAMTS20 (28460690)	DLCLNHLKPGAQKKCYANDCKS-------------------------------FTTCKEI	[1612]
Mus 20 (29500513)	WKVGKWSKCSVTCGIGIMERRVACRTENGWPSDLCLKRLKPDAQKKCYANDCKLLTTCKE	[1713]
Fugu 20	--	[1209]
Ciona 9 (0100132719)	KEKTVTSCRLTTRPYAVRDCDRGRCDSEVYWRVGSWSEVS--------------------	[1820]
Apis 9	--	[1195]
Drosophila CG6107 (20151419)	--	[1688]
Caenorhabditis gon-1 CEt13h10 (3879882)	GRGTKRRVVECVNPSLNVTVASTECDQTKKPVEEVRCRTKHCPRWKTTTWSSCSVTCGRG	[1712]
introns	--	[2]

```
[                                                                  2410      2420      2430      2440      2450      2460]
[                                                                     .         .         .         .         .         .]

mask                                            ------------------------------------------------------------  [571]
Homo ADAMTS6 (11131380)                         ------------------------------------------------------------  [1117]
Fugu 6                                          ------------------------------------------------------------  [1121]
Ciona 6 (0100146470)                            ------------------------------------------------------------  [1141]
Homo ADAMTS10 (17432918)                        ------------------------------------------------------------  [1103]
Mus 10 (27502095)                               ------------------------------------------------------------  [1070]
Fugu 10                                         ------------------------------------------------------------  [1058]
Homo ADAMTS16 (21265061)                        AFCKDYFHWCYLVPQHGMCSHKFYGKQCCKTCSKSNL-----------------------  [1225]
Mus 16 (21961374)                               ------------------------------------------------------------  [900]
Fugu 16                                         ------------------------------------------------------------  [1097]
Ciona 16 (0100138085)                           ------------------------------------------------------------  [1076]
Homo ADAMTS18 (21265067)                        PSCVDFFNWCHLVPQHGVCNHKFYGKQCCKSCTRKI------------------------  [1221]
Fugu 18                                         ------------------------------------------------------------  [742]
Homo ADAMTS12 (13569928)                        ------------------------------------------------------------  [1593]
Mus 12 (27817773)                               ------------------------------------------------------------  [1600]
Fugu 12                                         ------------------------------------------------------------  [1061]
Homo ADAMTS7 (11131377)                         VARR--------------------------------------------------------  [1686]
Fugu 7                                          ------------------------------------------------------------  [930]
Ciona 7 (genewise.34.12.1)                      ------------------------------------------------------------  [965]
Homo ADAMTS17 (21265064)                        ------------------------------------------------------------  [1122]
Homo ADAMTS19 (19525737)                        TFKCLGDQWPVYCRVIREKNLCQDMRWYQRCCETCRDFYAQKLQQKS-------------  [1207]
Drosophila dCG4096 (22831757)                   ------------------------------------------------------------  [1054]
Homo ADAMTS14 (29337086)                        ------------------------------------------------------------  [1115]
Fugu 14                                         ------------------------------------------------------------  [991]
Homo ADAMTS3 (2224673)                          ------------------------------------------------------------  [1205]
Fugu 3                                          ------------------------------------------------------------  [845]
Ciona 3 (0100146117)                            ------------------------------------------------------------  [997]
Homo ADAMTS2 (7656867)                          ------------------------------------------------------------  [1211]
Mus 2 (28204840)                                ------------------------------------------------------------  [1213]
Fugu 2                                          ------------------------------------------------------------  [799]
Homo ADAMTS13 (16306598)                        ------------------------------------------------------------  [1427]
Fugu 13                                         ------------------------------------------------------------  [1140]
Homo ADAMTS1 (6525075)                          ------------------------------------------------------------  [967]
Mus 1 (1813340)                                 ------------------------------------------------------------  [951]
Homo ADAMTS4 (12643637)                         ------------------------------------------------------------  [837]
Mus 4 (26350615)                                ------------------------------------------------------------  [845]
Fugu 4                                          ------------------------------------------------------------  [695]
Homo ADAMTS8 (21536392)                         ------------------------------------------------------------  [890]
Mus 8 (6708110)                                 ------------------------------------------------------------  [905]
Fugu 8                                          ------------------------------------------------------------  [867]
Homo ADAMTS5 (7768707)                          ------------------------------------------------------------  [930]
Mus 5 (5923784)                                 ------------------------------------------------------------  [930]
Fugu 5                                          ------------------------------------------------------------  [921]
Ciona 15 (0100137065)                           ------------------------------------------------------------  [987]
Homo ADAMTS15 (19171175)                        ------------------------------------------------------------  [950]
Fugu 15                                         ------------------------------------------------------------  [891]
Homo ADAMTS9 (27463365)                         TCRNVYN-----------------------------------------------------  [1697]
Fugu 9                                          CRNPSCEFRHCFAPDVCFPTCLCFVATAARNHFLLSRPFIFRSFAS--------------  [1522]
Homo ADAMTS20 (28460690)                        QVKN-HIRKDGDYYLNIKGRIIKVL-----------------------------------  [1636]
Mus 20 (29500513)                               LQVTNNVTKDGDYDLNVRGRILKIHCSGMQLENPREYLPLVKSEDNFSEIYGLRLQNPYE  [1773]
Fugu 20                                         ------------------------------------------------------------  [1209]
Ciona 9 (0100132719)                            ------------------------------------------------------------  [1820]
Apis 9                                          ------------------------------------------------------------  [1195]
Drosophila CG6107 (20151419)                    ------------------------------------------------------------  [1688]
Caenorhabditis gon-1 CEt13h10 (3879882)         IRRREVQCYRGRKNLVSDSECNPKTKLNSVANCFPVACPAYRWNVTPWSKCKDECARGQK  [1772]
introns                                         ------------------------------------------------------------  [2]
```

```
[                                                      2470      2480      2490      2500      2510      2520]
[                                                         .         .         .         .         .         .]

mask                                          ------------------------------------------------------------   [571]
Homo ADAMTS6 (11131380)                       ------------------------------------------------------------   [1117]
Fugu 6                                        ------------------------------------------------------------   [1121]
Ciona 6 (0100146470)                          ------------------------------------------------------------   [1141]
Homo ADAMTS10 (17432918)                      ------------------------------------------------------------   [1103]
Mus 10 (27502095)                             ------------------------------------------------------------   [1070]
Fugu 10                                       ------------------------------------------------------------   [1058]
Homo ADAMTS16 (21265061)                      ------------------------------------------------------------   [1225]
Mus 16 (21961374)                             ------------------------------------------------------------   [900]
Fugu 16                                       ------------------------------------------------------------   [1097]
Ciona 16 (0100138085)                         ------------------------------------------------------------   [1076]
Homo ADAMTS18 (21265067)                      ------------------------------------------------------------   [1221]
Fugu 18                                       ------------------------------------------------------------   [742]
Homo ADAMTS12 (13569928)                      ------------------------------------------------------------   [1593]
Mus 12 (27817773)                             ------------------------------------------------------------   [1600]
Fugu 12                                       ------------------------------------------------------------   [1061]
Homo ADAMTS7 (11131377)                       ------------------------------------------------------------   [1686]
Fugu 7                                        ------------------------------------------------------------   [930]
Ciona 7 (genewise.34.12.1)                    ------------------------------------------------------------   [965]
Homo ADAMTS17 (21265064)                      ------------------------------------------------------------   [1122]
Homo ADAMTS19 (19525737)                      ------------------------------------------------------------   [1207]
Drosophila dCG4096 (22831757)                 ------------------------------------------------------------   [1054]
Homo ADAMTS14 (29337086)                      ------------------------------------------------------------   [1115]
Fugu 14                                       ------------------------------------------------------------   [991]
Homo ADAMTS3 (2224673)                        ------------------------------------------------------------   [1205]
Fugu 3                                        ------------------------------------------------------------   [845]
Ciona 3 (0100146117)                          ------------------------------------------------------------   [997]
Homo ADAMTS2 (7656867)                        ------------------------------------------------------------   [1211]
Mus 2 (28204840)                              ------------------------------------------------------------   [1213]
Fugu 2                                        ------------------------------------------------------------   [799]
Homo ADAMTS13 (16306598)                      ------------------------------------------------------------   [1427]
Fugu 13                                       ------------------------------------------------------------   [1140]
Homo ADAMTS1 (6525075)                        ------------------------------------------------------------   [967]
Mus 1 (1813340)                               ------------------------------------------------------------   [951]
Homo ADAMTS4 (12643637)                       ------------------------------------------------------------   [837]
Mus 4 (26350615)                              ------------------------------------------------------------   [845]
Fugu 4                                        ------------------------------------------------------------   [695]
Homo ADAMTS8 (21536392)                       ------------------------------------------------------------   [890]
Mus 8 (6708110)                               ------------------------------------------------------------   [905]
Fugu 8                                        ------------------------------------------------------------   [867]
Homo ADAMTS5 (7768707)                        ------------------------------------------------------------   [930]
Mus 5 (5923784)                               ------------------------------------------------------------   [930]
Fugu 5                                        ------------------------------------------------------------   [921]
Ciona 15 (0100137065)                         ------------------------------------------------------------   [987]
Homo ADAMTS15 (19171175)                      ------------------------------------------------------------   [950]
Fugu 15                                       ------------------------------------------------------------   [891]
Homo ADAMTS9 (27463365)                       ------------------------------------------------------------   [1697]
Fugu 9                                        ------------------------------------------------------------   [1522]
Homo ADAMTS20 (28460690)                      ------------------------------------------------------------   [1636]
Mus 20 (29500513)                             CPFNGSRRPDCACENDYLPAGYTVFSKVRVDLESMQIKTADLLFSQTLSGKAVPFATAGD   [1833]
Fugu 20                                       ------------------------------------------------------------   [1209]
Ciona 9 (0100132719)                          ------------------------------------------------------------   [1820]
Apis 9                                        ------------------------------------------------------------   [1195]
Drosophila CG6107 (20151419)                  ------------------------------------------------------------   [1688]
Caenorhabditis gon-1 CEt13h10 (3879882)       QTRRVHCISTSGKRAAPRMCELARAPTSIRECDTSNCPYEWVPGDWQTCSKSCGEGVQTR   [1832]
introns                                       ------------------------------------------------------------   [2]
```

```
[                                                    2530      2540      2550      2560      2570      2580]
[                                                       .         .         .         .         .         .]

mask                                         ------------------------------------------------------------   [571]
Homo ADAMTS6 (11131380)                      ------------------------------------------------------------   [1117]
Fugu 6                                       ------------------------------------------------------------   [1121]
Ciona 6 (0100146470)                         ------------------------------------------------------------   [1141]
Homo ADAMTS10 (17432918)                     ------------------------------------------------------------   [1103]
Mus 10 (27502095)                            ------------------------------------------------------------   [1070]
Fugu 10                                      ------------------------------------------------------------   [1058]
Homo ADAMTS16 (21265061)                     ------------------------------------------------------------   [1225]
Mus 16 (21961374)                            ------------------------------------------------------------   [900]
Fugu 16                                      ------------------------------------------------------------   [1097]
Ciona 16 (0100138085)                        ------------------------------------------------------------   [1076]
Homo ADAMTS18 (21265067)                     ------------------------------------------------------------   [1221]
Fugu 18                                      ------------------------------------------------------------   [742]
Homo ADAMTS12 (13569928)                     ------------------------------------------------------------   [1593]
Mus 12 (27817773)                            ------------------------------------------------------------   [1600]
Fugu 12                                      ------------------------------------------------------------   [1061]
Homo ADAMTS7 (11131377)                      ------------------------------------------------------------   [1686]
Fugu 7                                       ------------------------------------------------------------   [930]
Ciona 7 (genewise.34.12.1)                   ------------------------------------------------------------   [965]
Homo ADAMTS17 (21265064)                     ------------------------------------------------------------   [1122]
Homo ADAMTS19 (19525737)                     ------------------------------------------------------------   [1207]
Drosophila dCG4096 (22831757)                ------------------------------------------------------------   [1054]
Homo ADAMTS14 (29337086)                     ------------------------------------------------------------   [1115]
Fugu 14                                      ------------------------------------------------------------   [991]
Homo ADAMTS3 (2224673)                       ------------------------------------------------------------   [1205]
Fugu 3                                       ------------------------------------------------------------   [845]
Ciona 3 (0100146117)                         ------------------------------------------------------------   [997]
Homo ADAMTS2 (7656867)                       ------------------------------------------------------------   [1211]
Mus 2 (28204840)                             ------------------------------------------------------------   [1213]
Fugu 2                                       ------------------------------------------------------------   [799]
Homo ADAMTS13 (16306598)                     ------------------------------------------------------------   [1427]
Fugu 13                                      ------------------------------------------------------------   [1140]
Homo ADAMTS1 (6525075)                       ------------------------------------------------------------   [967]
Mus 1 (1813340)                              ------------------------------------------------------------   [951]
Homo ADAMTS4 (12643637)                      ------------------------------------------------------------   [837]
Mus 4 (26350615)                             ------------------------------------------------------------   [845]
Fugu 4                                       ------------------------------------------------------------   [695]
Homo ADAMTS8 (21536392)                      ------------------------------------------------------------   [890]
Mus 8 (6708110)                              ------------------------------------------------------------   [905]
Fugu 8                                       ------------------------------------------------------------   [867]
Homo ADAMTS5 (7768707)                       ------------------------------------------------------------   [930]
Mus 5 (5923784)                              ------------------------------------------------------------   [930]
Fugu 5                                       ------------------------------------------------------------   [921]
Ciona 15 (0100137065)                        ------------------------------------------------------------   [987]
Homo ADAMTS15 (19171175)                     ------------------------------------------------------------   [950]
Fugu 15                                      ------------------------------------------------------------   [891]
Homo ADAMTS9 (27463365)                      ------------------------------------------------------------   [1697]
Fugu 9                                       ------------------------------------------------------------   [1522]
Homo ADAMTS20 (28460690)                     ------------------------------------------------------------   [1636]
Mus 20 (29500513)                            CYSAARCPQGQFSINLAGTGMKISNTAKWLAQGRYASVIIHRSQDGTKVYGRCGGFCGKC   [1893]
Fugu 20                                      ------------------------------------------------------------   [1209]
Ciona 9 (0100132719)                         ------------------------------------------------------------   [1820]
Apis 9                                       ------------------------------------------------------------   [1195]
Drosophila CG6107 (20151419)                 ------------------------------------------------------------   [1688]
Caenorhabditis gon-1 CEt13h10 (3879882)      EVRCRRKINFNSTIPIIFMLEDEPAVPKEKCELFPKPNESQTCELNPCDSEFKWSFGPWG   [1892]
introns                                      ------------------------------------------------------------   [2]
```

```
[                                                                   2590      2600      2610      2620      2630      2640]
[                                                                      .         .         .         .         .         .]

mask                                            ------------------------------------------------------------   [571]
Homo ADAMTS6 (11131380)                         ------------------------------------------------------------   [1117]
Fugu 6                                          ------------------------------------------------------------   [1121]
Ciona 6 (0100146470)                            ------------------------------------------------------------   [1141]
Homo ADAMTS10 (17432918)                        ------------------------------------------------------------   [1103]
Mus 10 (27502095)                               ------------------------------------------------------------   [1070]
Fugu 10                                         ------------------------------------------------------------   [1058]
Homo ADAMTS16 (21265061)                        ------------------------------------------------------------   [1225]
Mus 16 (21961374)                               ------------------------------------------------------------   [900]
Fugu 16                                         ------------------------------------------------------------   [1097]
Ciona 16 (0100138085)                           ------------------------------------------------------------   [1076]
Homo ADAMTS18 (21265067)                        ------------------------------------------------------------   [1221]
Fugu 18                                         ------------------------------------------------------------   [742]
Homo ADAMTS12 (13569928)                        ------------------------------------------------------------   [1593]
Mus 12 (27817773)                               ------------------------------------------------------------   [1600]
Fugu 12                                         ------------------------------------------------------------   [1061]
Homo ADAMTS7 (11131377)                         ------------------------------------------------------------   [1686]
Fugu 7                                          ------------------------------------------------------------   [930]
Ciona 7 (genewise.34.12.1)                      ------------------------------------------------------------   [965]
Homo ADAMTS17 (21265064)                        ------------------------------------------------------------   [1122]
Homo ADAMTS19 (19525737)                        ------------------------------------------------------------   [1207]
Drosophila dCG4096 (22831757)                   ------------------------------------------------------------   [1054]
Homo ADAMTS14 (29337086)                        ------------------------------------------------------------   [1115]
Fugu 14                                         ------------------------------------------------------------   [991]
Homo ADAMTS3 (2224673)                          ------------------------------------------------------------   [1205]
Fugu 3                                          ------------------------------------------------------------   [845]
Ciona 3 (0100146117)                            ------------------------------------------------------------   [997]
Homo ADAMTS2 (7656867)                          ------------------------------------------------------------   [1211]
Mus 2 (28204840)                                ------------------------------------------------------------   [1213]
Fugu 2                                          ------------------------------------------------------------   [799]
Homo ADAMTS13 (16306598)                        ------------------------------------------------------------   [1427]
Fugu 13                                         ------------------------------------------------------------   [1140]
Homo ADAMTS1 (6525075)                          ------------------------------------------------------------   [967]
Mus 1 (1813340)                                 ------------------------------------------------------------   [951]
Homo ADAMTS4 (12643637)                         ------------------------------------------------------------   [837]
Mus 4 (26350615)                                ------------------------------------------------------------   [845]
Fugu 4                                          ------------------------------------------------------------   [695]
Homo ADAMTS8 (21536392)                         ------------------------------------------------------------   [890]
Mus 8 (6708110)                                 ------------------------------------------------------------   [905]
Fugu 8                                          ------------------------------------------------------------   [867]
Homo ADAMTS5 (7768707)                          ------------------------------------------------------------   [930]
Mus 5 (5923784)                                 ------------------------------------------------------------   [930]
Fugu 5                                          ------------------------------------------------------------   [921]
Ciona 15 (0100137065)                           ------------------------------------------------------------   [987]
Homo ADAMTS15 (19171175)                        ------------------------------------------------------------   [950]
Fugu 15                                         ------------------------------------------------------------   [891]
Homo ADAMTS9 (27463365)                         ------------------------------------------------------------   [1697]
Fugu 9                                          ------------------------------------------------------------   [1522]
Homo ADAMTS20 (28460690)                        ------------------------------------------------------------   [1636]
Mus 20 (29500513)                               IPHMATGLSIQVL-----------------------------------------------   [1906]
Fugu 20                                         ------------------------------------------------------------   [1209]
Ciona 9 (0100132719)                            ------------------------------------------------------------   [1820]
Apis 9                                          ------------------------------------------------------------   [1195]
Drosophila CG6107 (20151419)                    ------------------------------------------------------------   [1688]
Caenorhabditis gon-1 CEt13h10 (3879882)         ECSKNCGQGIRRRRVKCVANDGRRVERVKCTTKKPRRTQYCFERNCLPSTCQELKSQNVK   [1952]
introns                                         ------------------------------------------------------------   [2]
```

[	2650 2660 2670 2680 2690 2700]	
[	. . . . . .]	
mask	--	[571]
Homo ADAMTS6 (11131380)	--	[1117]
Fugu 6	--	[1121]
Ciona 6 (0100146470)	--	[1141]
Homo ADAMTS10 (17432918)	--	[1103]
Mus 10 (27502095)	--	[1070]
Fugu 10	--	[1058]
Homo ADAMTS16 (21265061)	--	[1225]
Mus 16 (21961374)	--	[900]
Fugu 16	--	[1097]
Ciona 16 (0100138085)	--	[1076]
Homo ADAMTS18 (21265067)	--	[1221]
Fugu 18	--	[742]
Homo ADAMTS12 (13569928)	--	[1593]
Mus 12 (27817773)	--	[1600]
Fugu 12	--	[1061]
Homo ADAMTS7 (11131377)	--	[1686]
Fugu 7	--	[930]
Ciona 7 (genewise.34.12.1)	--	[965]
Homo ADAMTS17 (21265064)	--	[1122]
Homo ADAMTS19 (19525737)	--	[1207]
Drosophila dCG4096 (22831757)	--	[1054]
Homo ADAMTS14 (29337086)	--	[1115]
Fugu 14	--	[991]
Homo ADAMTS3 (2224673)	--	[1205]
Fugu 3	--	[845]
Ciona 3 (0100146117)	--	[997]
Homo ADAMTS2 (7656867)	--	[1211]
Mus 2 (28204840)	--	[1213]
Fugu 2	--	[799]
Homo ADAMTS13 (16306598)	--	[1427]
Fugu 13	--	[1140]
Homo ADAMTS1 (6525075)	--	[967]
Mus 1 (1813340)	--	[951]
Homo ADAMTS4 (12643637)	--	[837]
Mus 4 (26350615)	--	[845]
Fugu 4	--	[695]
Homo ADAMTS8 (21536392)	--	[890]
Mus 8 (6708110)	--	[905]
Fugu 8	--	[867]
Homo ADAMTS5 (7768707)	--	[930]
Mus 5 (5923784)	--	[930]
Fugu 5	--	[921]
Ciona 15 (0100137065)	--	[987]
Homo ADAMTS15 (19171175)	--	[950]
Fugu 15	--	[891]
Homo ADAMTS9 (27463365)	--	[1697]
Fugu 9	--	[1522]
Homo ADAMTS20 (28460690)	--	[1636]
Mus 20 (29500513)	--	[1906]
Fugu 20	--	[1209]
Ciona 9 (0100132719)	--	[1820]
Apis 9	--	[1195]
Drosophila CG6107 (20151419)	--	[1688]
Caenorhabditis gon-1 CEt13h10 (3879882)	AKDGNYTILLDGFTIEIYCHRMNSTIPKAYLNVNPRTNFAEVYGKKLIYPHTCPFNGDRN	[2012]
introns	--	[2]

[	2710 2720 2730 2740 2750 2760]	
[	. . . . . .]	
mask	--	[571]
Homo ADAMTS6 (11131380)	--	[1117]
Fugu 6	--	[1121]
Ciona 6 (0100146470)	--	[1141]
Homo ADAMTS10 (17432918)	--	[1103]
Mus 10 (27502095)	--	[1070]
Fugu 10	--	[1058]
Homo ADAMTS16 (21265061)	--	[1225]
Mus 16 (21961374)	--	[900]
Fugu 16	--	[1097]
Ciona 16 (0100138085)	--	[1076]
Homo ADAMTS18 (21265067)	--	[1221]
Fugu 18	--	[742]
Homo ADAMTS12 (13569928)	--	[1593]
Mus 12 (27817773)	--	[1600]
Fugu 12	--	[1061]
Homo ADAMTS7 (11131377)	--	[1686]
Fugu 7	--	[930]
Ciona 7 (genewise.34.12.1)	--	[965]
Homo ADAMTS17 (21265064)	--	[1122]
Homo ADAMTS19 (19525737)	--	[1207]
Drosophila dCG4096 (22831757)	--	[1054]
Homo ADAMTS14 (29337086)	--	[1115]
Fugu 14	--	[991]
Homo ADAMTS3 (2224673)	--	[1205]
Fugu 3	--	[845]
Ciona 3 (0100146117)	--	[997]
Homo ADAMTS2 (7656867)	--	[1211]
Mus 2 (28204840)	--	[1213]
Fugu 2	--	[799]
Homo ADAMTS13 (16306598)	--	[1427]
Fugu 13	--	[1140]
Homo ADAMTS1 (6525075)	--	[967]
Mus 1 (1813340)	--	[951]
Homo ADAMTS4 (12643637)	--	[837]
Mus 4 (26350615)	--	[845]
Fugu 4	--	[695]
Homo ADAMTS8 (21536392)	--	[890]
Mus 8 (6708110)	--	[905]
Fugu 8	--	[867]
Homo ADAMTS5 (7768707)	--	[930]
Mus 5 (5923784)	--	[930]
Fugu 5	--	[921]
Ciona 15 (0100137065)	--	[987]
Homo ADAMTS15 (19171175)	--	[950]
Fugu 15	--	[891]
Homo ADAMTS9 (27463365)	--	[1697]
Fugu 9	--	[1522]
Homo ADAMTS20 (28460690)	--	[1636]
Mus 20 (29500513)	--	[1906]
Fugu 20	--	[1209]
Ciona 9 (0100132719)	--	[1820]
Apis 9	--	[1195]
Drosophila CG6107 (20151419)	--	[1688]
Caenorhabditis gon-1 CEt13h10 (3879882)	DSCHCSEDGDASAGLTRFNKVRIDLLNRKFHLADYTFAKREYGVHVPYGTAGDCYSMKDC	[2072]
introns	--	[2]

```
[                                                    2770      2780      2790      2800      2810      2820]
[                                                    .         .         .         .         .         .]

mask                                          ------------------------------------------------------------   [571]
Homo ADAMTS6 (11131380)                       ------------------------------------------------------------   [1117]
Fugu 6                                        ------------------------------------------------------------   [1121]
Ciona 6 (0100146470)                          ------------------------------------------------------------   [1141]
Homo ADAMTS10 (17432918)                      ------------------------------------------------------------   [1103]
Mus 10 (27502095)                             ------------------------------------------------------------   [1070]
Fugu 10                                       ------------------------------------------------------------   [1058]
Homo ADAMTS16 (21265061)                      ------------------------------------------------------------   [1225]
Mus 16 (21961374)                             ------------------------------------------------------------   [900]
Fugu 16                                       ------------------------------------------------------------   [1097]
Ciona 16 (0100138085)                         ------------------------------------------------------------   [1076]
Homo ADAMTS18 (21265067)                      ------------------------------------------------------------   [1221]
Fugu 18                                       ------------------------------------------------------------   [742]
Homo ADAMTS12 (13569928)                      ------------------------------------------------------------   [1593]
Mus 12 (27817773)                             ------------------------------------------------------------   [1600]
Fugu 12                                       ------------------------------------------------------------   [1061]
Homo ADAMTS7 (11131377)                       ------------------------------------------------------------   [1686]
Fugu 7                                        ------------------------------------------------------------   [930]
Ciona 7 (genewise.34.12.1)                    ------------------------------------------------------------   [965]
Homo ADAMTS17 (21265064)                      ------------------------------------------------------------   [1122]
Homo ADAMTS19 (19525737)                      ------------------------------------------------------------   [1207]
Drosophila dCG4096 (22831757)                 ------------------------------------------------------------   [1054]
Homo ADAMTS14 (29337086)                      ------------------------------------------------------------   [1115]
Fugu 14                                       ------------------------------------------------------------   [991]
Homo ADAMTS3 (2224673)                        ------------------------------------------------------------   [1205]
Fugu 3                                        ------------------------------------------------------------   [845]
Ciona 3 (0100146117)                          ------------------------------------------------------------   [997]
Homo ADAMTS2 (7656867)                        ------------------------------------------------------------   [1211]
Mus 2 (28204840)                              ------------------------------------------------------------   [1213]
Fugu 2                                        ------------------------------------------------------------   [799]
Homo ADAMTS13 (16306598)                      ------------------------------------------------------------   [1427]
Fugu 13                                       ------------------------------------------------------------   [1140]
Homo ADAMTS1 (6525075)                        ------------------------------------------------------------   [967]
Mus 1 (1813340)                               ------------------------------------------------------------   [951]
Homo ADAMTS4 (12643637)                       ------------------------------------------------------------   [837]
Mus 4 (26350615)                              ------------------------------------------------------------   [845]
Fugu 4                                        ------------------------------------------------------------   [695]
Homo ADAMTS8 (21536392)                       ------------------------------------------------------------   [890]
Mus 8 (6708110)                               ------------------------------------------------------------   [905]
Fugu 8                                        ------------------------------------------------------------   [867]
Homo ADAMTS5 (7768707)                        ------------------------------------------------------------   [930]
Mus 5 (5923784)                               ------------------------------------------------------------   [930]
Fugu 5                                        ------------------------------------------------------------   [921]
Ciona 15 (0100137065)                         ------------------------------------------------------------   [987]
Homo ADAMTS15 (19171175)                      ------------------------------------------------------------   [950]
Fugu 15                                       ------------------------------------------------------------   [891]
Homo ADAMTS9 (27463365)                       ------------------------------------------------------------   [1697]
Fugu 9                                        ------------------------------------------------------------   [1522]
Homo ADAMTS20 (28460690)                      ------------------------------------------------------------   [1636]
Mus 20 (29500513)                             ------------------------------------------------------------   [1906]
Fugu 20                                       ------------------------------------------------------------   [1209]
Ciona 9 (0100132719)                          ------------------------------------------------------------   [1820]
Apis 9                                        ------------------------------------------------------------   [1195]
Drosophila CG6107 (20151419)                  ------------------------------------------------------------   [1688]
Caenorhabditis gon-1 CEt13h10 (3879882)       PQGIFSIDLKSAGLKLVDDLNWEDQGHRTSSRIDRFYNNAKVIGHCGGFCGKCSPERYKG   [2132]
introns                                       ------------------------------------------------------------   [2]
```

```
[                                                     2830      2840      2850      2860      2870   ]
[                                                     .         .         .         .         .      ]

mask                                       -------------------------------------------------------   [571]
Homo ADAMTS6 (11131380)                    -------------------------------------------------------   [1117]
Fugu 6                                     -------------------------------------------------------   [1121]
Ciona 6 (0100146470)                       -------------------------------------------------------   [1141]
Homo ADAMTS10 (17432918)                   -------------------------------------------------------   [1103]
Mus 10 (27502095)                          -------------------------------------------------------   [1070]
Fugu 10                                    -------------------------------------------------------   [1058]
Homo ADAMTS16 (21265061)                   -------------------------------------------------------   [1225]
Mus 16 (21961374)                          -------------------------------------------------------   [900]
Fugu 16                                    -------------------------------------------------------   [1097]
Ciona 16 (0100138085)                      -------------------------------------------------------   [1076]
Homo ADAMTS18 (21265067)                   -------------------------------------------------------   [1221]
Fugu 18                                    -------------------------------------------------------   [742]
Homo ADAMTS12 (13569928)                   -------------------------------------------------------   [1593]
Mus 12 (27817773)                          -------------------------------------------------------   [1600]
Fugu 12                                    -------------------------------------------------------   [1061]
Homo ADAMTS7 (11131377)                    -------------------------------------------------------   [1686]
Fugu 7                                     -------------------------------------------------------   [930]
Ciona 7 (genewise.34.12.1)                 -------------------------------------------------------   [965]
Homo ADAMTS17 (21265064)                   -------------------------------------------------------   [1122]
Homo ADAMTS19 (19525737)                   -------------------------------------------------------   [1207]
Drosophila dCG4096 (22831757)              -------------------------------------------------------   [1054]
Homo ADAMTS14 (29337086)                   -------------------------------------------------------   [1115]
Fugu 14                                    -------------------------------------------------------   [991]
Homo ADAMTS3 (2224673)                     -------------------------------------------------------   [1205]
Fugu 3                                     -------------------------------------------------------   [845]
Ciona 3 (0100146117)                       -------------------------------------------------------   [997]
Homo ADAMTS2 (7656867)                     -------------------------------------------------------   [1211]
Mus 2 (28204840)                           -------------------------------------------------------   [1213]
Fugu 2                                     -------------------------------------------------------   [799]
Homo ADAMTS13 (16306598)                   -------------------------------------------------------   [1427]
Fugu 13                                    -------------------------------------------------------   [1140]
Homo ADAMTS1 (6525075)                     -------------------------------------------------------   [967]
Mus 1 (1813340)                            -------------------------------------------------------   [951]
Homo ADAMTS4 (12643637)                    -------------------------------------------------------   [837]
Mus 4 (26350615)                           -------------------------------------------------------   [845]
Fugu 4                                     -------------------------------------------------------   [695]
Homo ADAMTS8 (21536392)                    -------------------------------------------------------   [890]
Mus 8 (6708110)                            -------------------------------------------------------   [905]
Fugu 8                                     -------------------------------------------------------   [867]
Homo ADAMTS5 (7768707)                     -------------------------------------------------------   [930]
Mus 5 (5923784)                            -------------------------------------------------------   [930]
Fugu 5                                     -------------------------------------------------------   [921]
Ciona 15 (0100137065)                      -------------------------------------------------------   [987]
Homo ADAMTS15 (19171175)                   -------------------------------------------------------   [950]
Fugu 15                                    -------------------------------------------------------   [891]
Homo ADAMTS9 (27463365)                    -------------------------------------------------------   [1697]
Fugu 9                                     -------------------------------------------------------   [1522]
Homo ADAMTS20 (28460690)                   -------------------------------------------------------   [1636]
Mus 20 (29500513)                          -------------------------------------------------------   [1906]
Fugu 20                                    -------------------------------------------------------   [1209]
Ciona 9 (0100132719)                       -------------------------------------------------------   [1820]
Apis 9                                     -------------------------------------------------------   [1195]
Drosophila CG6107 (20151419)               -------------------------------------------------------   [1688]
Caenorhabditis gon-1 CEt13h10 (3879882)    LIFEVNTKLLNHVKNGGHIDDELDDDGFSGDMD----------------------   [2165]
introns                                    -------------------------------------------------------   [2]
;
END;
```
