# Supplementary material for: Functional evolution of ADAMTS genes: Evidence from analyses of phylogeny and gene organization
Source: BMC Evol Biol. 2005 Feb 4;5:11. doi: 10.1186/1471-2148-5-11 (PMC549037; doi:10.1186/1471-2148-5-11)
Supplement: Additional File 1 — Alignment used for phylogenetic analyses of human ADAMTS proteins and invertebrate homologs. Unambiguously aligned amino acid sites, indicated by the "mask" line in the alignment, were used for phylogenetic analyses (figure 1). Intron positions in the corresponding genomic sequence are indicated, with color-coding for intron phases (Figures 3 and 4). [file 1471-2148-5-11-S1.pdf]

Additional file #1 for:

Research Article

Functional evolution of ADAMTS genes: Evidence from analyses of phylogeny and gene organization

Ainsley C. Nicholson[1,3*], Shehre-Banoo Malik[2*], John M. Logsdon Jr.[2] and Erwin G. Van Meir[1§]

[1]Laboratory of Molecular Neuro-Oncology, Winship Cancer Institute, 1365-C Clifton Road, Room C5078, Emory University, Atlanta GA 30322 USA. Phone: 404-778-5563, Fax: 404-778-5240.
[2]Roy J. Carver Center for Comparative Genomics, Department of Biological Sciences, 300 Old Biology Building, University of Iowa, Iowa City IA 52242-1324 USA. Phone: 319-335-1083, Fax: 319-335-1069
[3]Present address: Viral Exanthems and Herpesvirus Branch, Centers for Disease Control and Prevention, 1600 Clifton Rd, Mailstop A14, Atlanta, GA 30333 USA.

* These authors contributed equally to this work

§ Corresponding author

Email addresses:
ACN: agn0@cdc.gov
SBM: banoo-malik@uiowa.edu
JML: john-logsdon@uiowa.edu
EGVM: evanmei@emory.edu

Additional File 1 **– (NMLVadamtsalign1.pdf) Alignment used for phylogenetic analyses of human ADAMTS proteins and invertebrate homologs .** Unambiguously aligned amino acid sites, indicated by the “mask” line in the alignment, were used for phylogenetic analyses (figure 1). Intron positions in the corresponding genomic sequence are indicated, with color-coding for intron phases (table 1 and figure 3).

```
#NEXUS
[AC Nicholson, SB Malik, JM Logsdon Jr, E van Meir, 2004. Functional evolution of ADAMTS genes:]
[evidence from analyses of phylogeny and gene organization. Contact: john-logsdon@uiowa.edu]
[Intron phases marked 0, 1, 2]
BEGIN DATA;
        DIMENSIONS  NTAX=23 NCHAR=2611;
        FORMAT DATATYPE=PROTEIN  SYMBOLS = " 1 2 3 4"  MISSING=? GAP=-  INTERLEAVE ;
MATRIX
[                    10        20        30        40        50        60]
[                    .         .         .         .         .         .]
ADAMTS6     --------------------MEILWKTL-TWILSLIMAS--------------------S    [19]
ADAMTS10    --------------------MAPACQIL-RWALALGLG---------------------L    [18]
ADAMTS18    --------MECALLLACAFPAAGSGPPRGLAGLGRVAKA------LQLCCLCCASVAAAL    [46]
ADAMTS16    -------------MKPRARGWRGLAALW-MLLAQVAEQAPACAM-GPAAAAPGSPSVPRP    [45]
ADAMTS7     --------------------MPGGPSPR-SPAPLLRPLLLLLCA-LAPG-----------    [27]
ADAMTS12    --------------------MPCAQRSW-LANLSVVAQLLNFGA-LCYG-----------    [27]
ADAMTS17    -----------------------------------------------MCDGALLPPLVLP    [13]
ADAMTS19    MRLTHICCCCLLYQLGFLSNGIVSELQFAPDREEWEVVFPALWRREPVDPAGGSGGSADP    [60]
ADAMTS2     ------------MDPPAGAARRLLCPAL-LLLLLLLPPPLLPPP-PP----------PAN    [36]
ADAMTS3     -----------------------MVLLS-LWLIAAALVEVRTSA-DG-------------    [22]
ADAMTS14    -------------------------MAP-LRALLSYLLPLHCAL-CA-------------    [20]
ADAMTS13    ------------------------------------------------------------    [0]
dCG4096     ------------------------MWIR-SRWLGLLLHYWLVVV-TQGGGVRPLYGLHSD    [34]
ADAMTS4     -------------------MSQTGSHPG-RGLAGRWLWGAQPCL-LLP-----------I    [28]
ADAMTS5     ----------------MLLGWASLLLCA-FRLPLAAVGPAATPA-QDK-----------A    [31]
ADAMTS8     ------------------------------------MFPAPAAP----------------    [8]
ADAMTS1     ------------------MQRAVPEGFG-RRKLGSDMGNAERAP-GS-------------    [27]
ADAMTS15    ------------------------------------------------------------    [0]
dCG6107     ---------------------------MPDGAANLHAVAHNLRSKRCANRIPHFSLSIFQ    [33]
ADAMTS9     ---------------MQFVSWATLLTLL-VRDLAEMGSPDAAAA-VRKDR----------    [33]
ADAMTS20    -----------------------------MRNAWELTGPVAVVA-EA-------------    [17]
mask        ------------------------------------------------------------    [0]
gon-1       -----------------------------------------MRS-IGGSFHLLQPVVAAL    [18]
[Intron                              1            2                          ]

[                    70        80        90        100       110       120]
[                    .         .         .         .         .         .]
ADAMTS6     EFHSDHRLSYSSQEEFLTYLEHYQLTIPIRVDQNGAFLSFTVKN---DKHSRRRRSMDPI    [76]
ADAMTS10    MFEVTH--AFRSQDEFLSSLESYEIAFPTRVDHNGALLAFSPPP---PRRQRRG------    [67]
ADAMTS18    ASDSSSGASGLNDDYVFVTPVEVDSAGSYISHDILH---------NGRKKRS--------    [89]
ADAMTS16    PPPAERPGWMEKGEYDLVSAYEVDHRGDYVSHEIMH-----------HQRRRRAV-----    [89]
ADAMTS7     -------APGPAPGRATEGRAALDIVHPVRVDAGGSFLSYELWP---RALRKRD------    [71]
ADAMTS12    -------RQPQPGPVRFPDRRQEHFIKGLPEYHVVGPVRVDASGHFLSYGLHYPITSSRR    [80]
ADAMTS17    VLLLLVWGLDPGTAVGDAAADVEVVLPWRVRPDDVHLPPLPAAPGPRRRRRPRTPPAAPR    [73]
ADAMTS19    GWVRGVGGGGSARAQAAGSSREVRSVAPVPLEEPVEGRSESRLRPPPPPSEGEEDEELES    [120]
ADAMTS2     ARLAAAADPPG---GPLGHGAERILAVPVRTDAQGRLVSHVVSAATSRAGVRARRAAP--    [91]
ADAMTS3     QAGNEEMVQID---LPIKRYREYELVTPVSTNLEGRYLSHTLSASHKKRSARDVSSNP--    [77]
ADAMTS14    ---AAGSRTPE---LHLSGKLSDYGVTVPCSTDFRGRFLSHVVSGPAAASAGSMVVDTPP    [74]
ADAMTS13    ------------------------------------------------------------    [0]
dCG4096     ELVAGEGQLVVPRRVHPDGAFMTHQLEYAHE---------LDHRRHRQRRSLNSEHDTQA    [85]
ADAMTS4     VPLSWLVWLLLLLLASLLPSARLASPLPREEEIVFPEKLNGSV--------LPG------    [74]
ADAMTS5     GQPPTAAAAAQPRRRQGEEVQERAEPPGHPHPLAQRRRSKGLVQN-----IDQL------    [80]
ADAMTS8     RWLPFLLLLLLLLLPLARGAPARPAAGGQASELVVPTRLP--------------------    [48]
ADAMTS1     RSFGPVPTLLLLAAALLAVSDALGRPSEEDEELVVPELER-----------APG------    [70]
ADAMTS15    --------MLLLGILTLAFAGRTAGGSEPEREVVVPIRLDPDINGRRYYWRGPE------    [46]
dCG6107     LEVKKRCEWWCHKYIQKMSTHWRQNACLYACCIAFLLGMLIMFHLGLRSAHKGQEELPQS    [93]
ADAMTS9     --------LHPRQVKLLETLGEYEIVSPIRVNALGEPFPTNVHFKRTRRSINSATDPWPA    [85]
ADAMTS20    ----LVRTLTSYEVVIPERVNEFGEVFPQSHHFSRQKRSS-EALEPMP------------    [60]
mask        ------------------------------------------------------------    [0]
Ce gon-1    ILLVVCLVYALQSGSGTISEFSSDVLFSRAKYSG---VPVHHSRWRQDAGIHVIDSHHIV    [75]
[Intron                   3                     4                             ]
```

```
[                    130       140       150       160       170       180]
[                    .         .         .         .         .         .]
[Modal, ≥80%, full maj.                                              L]
ADAMTS6     ------------------------------------DPQQAVSKLFFKLSAYGKHFHLNL   [100]
ADAMTS10    ------------------------------------TGATAESRLFYKVASPSTHFLLNL   [91]
ADAMTS18    -------------------------------------AQNARSSLHYRFSAFGQELHLEL   [112]
ADAMTS16    -------------------------------------AVSEVESLHLRLKGPRHDFHMDL   [112]
ADAMTS7     ------------------------------------VSVRRDAPAFYELQYRGRELRFNL   [95]
ADAMTS12    KRD---------------------------------LDGS-EDWVYYRISHEEKDLFFNL   [106]
ADAMTS17    A-RPG------------------------------------ERALLLHLPAFGRDLYLQL   [96]
ADAMTS19    QELPRGSSGAAALSPGAPASWQPPPPPQPPSPPPAQHAEPDGDEVLLRIPAFSRDLYLLL   [180]
ADAMTS2     ------------------------VRTPSFPGGN--E-EEPGSHLFYNVTVFGRDLHLRL   [124]
ADAMTS3     ------------------------------------------EQLFFNITAFGKDFHLRL   [95]
ADAMTS14    T-LPRHS------------SHLRVARSPLHPGGTLWPGRVGRHSLYFNVTVFGKELHLRL   [121]
ADAMTS13    ----------------------------------------------------MHQRHPRA   [8]
dCG4096     A-DLHLL------------------------------------------LPLANETLHLEL   [103]
ADAMTS4     -------------------------------------SGAPAR-LLCRLQAFGETLLLEL   [96]
ADAMTS5     -------------------------------------YSGGGK-VGYLVYAGGRRFLLDL   [102]
ADAMTS8     --------------------------------------GSAGE-LALHLSAFGKGFVLRL   [69]
ADAMTS1     -------------------------------------HGTTR----LRLHAFDQQLDLEL   [89]
ADAMTS15    -------------------------------------DSGDQG-LIFQITAFQEDFYLHL   [68]
dCG6107     THPLANSPPATPATLHPRRLDNDTSTDHEPPDGLDDLDEEEHSAFVMPTKVYNYSLSEAD   [153]
ADAMTS9     F-ASSS-------------------------------SSSTSSQAHYRLSAFGQQFLFNL   [113]
ADAMTS20    ----------------------------------------FR--THYRFTAYGQLFQLNL   [78]
mask        ------------------------------------------------------------   [0]
Ce gon-1    RRDSYGR-----------------------RGKRDVTSTDRRRRLQGVARDCGHACHLRL   [112]

[                    190       200       210       220       230       240]
[                    .         .         .         .         .         .]
[Modal, ≥80%, full maj.                                                              ]
ADAMTS6     TLNTDFVSKHFTVEYW--------------------------------------------   [116]
ADAMTS10    TRSSRLLAGHVSVEYW--------------------------------------------   [107]
ADAMTS18    KP-SAILSSHFIVQVL--------------------------------------------   [127]
ADAMTS16    RTSSSLVAPGFIVQTL--------------------------------------------   [128]
ADAMTS7     TANQHLLAPGFVSETR--------------------------------------------   [111]
ADAMTS12    TVNQGFLSNSYIMEKR--------------------------------------------   [122]
ADAMTS17    RRDLRFLSRGFEVEE---------------------------------------------   [111]
ADAMTS19    RRDGRFLAPRFAVEQRPNPGPGPT------------------------------------   [204]
ADAMTS2     RPNARLVAPGATMEWQG---------E---------------------------------   [142]
ADAMTS3     KPNTQLVAPGAVVEWHETSLVPGNITDPINNHQ---------------------------   [128]
ADAMTS14    RPNRRLVVPGSSVEWQE---------D---------------------------------   [139]
ADAMTS13    RCPPLCVAGILACGFLLGCW----------------------------------------   [28]
dCG4096     MAHSYFLAPNLVVERHRRDLRTRSPLTTRHL-----------------------------   [134]
ADAMTS4     EQDSGVQVEGLTVQYLG-------------------------------------------   [113]
ADAMTS5     ERDGSVGIAGFVPAGGG-------------------------------------------   [119]
ADAMTS8     APDDSFLAPEFKIERLG-------------------------------------------   [86]
ADAMTS1     RPDSSFLAPGFTLQNVGRK-----------------------------------------   [108]
ADAMTS15    TPDAQFLAPAFSTEHLG-V-----------------------------------------   [86]
dCG6107     LIYESKRNSDINSFLKESASAFAMTGTYRNMSNEIWDPHPQYNLNVFGRQLHLVLRQDAS   [213]
ADAMTS9     TANAGFIAPLFTVTLLGTPGV---------------------------------------   [134]
ADAMTS20    TADASFLAAGYTEVHLGTP-----------------------------------------   [97]
mask        ------------------------------------------------------------   [0]
gon-1       RSDDAVYIVHLHRWNQIPDSHNKSVPHFSNSNFAPMVLYLDSE-----------------   [155]
[Intron                      5     6                                                 ]
```

```
[                    250       260       270       280       290       300]
[                    .         .         .         .         .         .]
[Modal, ≥80%, full maj.                            C   G                 G ]
ADAMTS6     -------------------GKDGPQWKH--DFLDNCHYTGYLQDQRSTT-KVALSNCVG-   [153]
ADAMTS10    -------------------TREGLAWQR--AARPHCLYAGHLQGQASSS-HVAISTCGG-   [144]
ADAMTS18    -------------------GKDGASETQ-KPEVQQCFYQGFIRNDSSSS--VAVSTCAG-   [164]
ADAMTS16    -------------------GKTGTKSVQTLPPEDFCFYQGSLRSHRNSS--VALSTCQG-   [166]
ADAMTS7     -------------------RRGGLGRAHIRAHTPACHLLGEVQDPELEGGLAAISACDG-   [151]
ADAMTS12    -------------------YGNLSHVKMMASSAPLCHLSGTVLQQGTRVGTAALSACHG-   [162]
ADAMTS17    ----------------------AGAARRRGRPAELCFYSGRVLGHPGSL--VSLSACGAA   [147]
ADAMTS19    --------------------GAASAPQPPAPPDAGCFYTGAVLRHPGSL--ASFSTCGG-   [241]
ADAMTS2     -----------------------KGTTRVEPLLGSCLYVGDVAGLAEASS-VALSNCDG-   [177]
ADAMTS3     ------------------PGSATYRIRKTEPLQTNCAYVGDIVDIPGTS--VAISNCDG-   [167]
ADAMTS14    -----------------------FRELFRQPLRQECVYTGGVTGMPGAA--VAISNCDG-   [173]
ADAMTS13    ---------------------------GPSHFQQSCLQALEPQ--------AVSS-----   [48]
dCG4096     -----------------NCHFHGKVRGQPATNVAISTCAGLVSCPNFC-VLPELITLCQ-   [175]
ADAMTS4     -----------------------QAPELLGGAEPGTYLTGTINGDPESV--ASLHWDGGA   [148]
ADAMTS5     -------------------------TSAPWRHRSHCFYRGTVDASPRSL--AVFDLCGGL   [152]
ADAMTS8     ---------------------GSGRATGGERGLRGCFFSGTVNGEPESL--AAVSLCRG-   [122]
ADAMTS1     ---------------------SGSETPLPETDLAHCFYSGTVNGDPSSA--AALSLCEG-   [144]
ADAMTS15    ---------------------PLQGLTGGSSDLRRCFYSGDVNAEPDSF--AAVSLCGG-   [122]
dCG6107     F-VHNHSMTHIRILKEGEEHPGPETEAEAEQRHLGCFYSGYVEDDPHSM--VSVSLCGG-   [269]
ADAMTS9     -------------------NQTKFYSEE-EAELKHCFYKGYVNTNSEHT--AVISLCSG-   [171]
ADAMTS20    -------------------ERGAWESDAGPSDLRHCFYRGQVNSQEDYK--AVVSLCGG-   [135]
mask        ----------------------------------111111111--------1111-----   [13]
Ce gon-1    ----------------------EEVRGGMSRTDPDCIYRAHVKGVHQHSIVNLCDSEDG-   [192]
[Intron                                   7                                ]
[                                                Prodomain                          ]

[                    310       320       330       340       350       360]
[                    .         .         .         .         .         .]
[≥80%, full maj.G            P                   H                       ]
ADAMTS6     --LHGVIATEDEEYFIEPLKN-TTEDSKHFSYENG-HPHVIYKKSALQQRHLYDHSHCGV   [209]
ADAMTS10    --LHGLIVADEEEYLIEPLHG--GPKGSR-SPEE-SGPHVVYKRSSLRHPHLDTACGVRD   [198]
ADAMTS18    --LSGLIRTRKNEFLISPLPQL-LAQEHNYSSPAGHHPHVLYKRTAEEKIQRYRGYPGSG   [221]
ADAMTS16    --LSGMIRTEEADYFLRPLPSH-LSWKLGRAAQGSSPSHVLYKRSTEPHAPGASEVLVTS   [223]
ADAMTS7     --LKGVFQLSNEDYFIEPLDS--APARPG-----HAQPHVVYKRQAPERLAQRGDSSAPS   [202]
ADAMTS12    --LTGFFQLPHGDFFIEPVKK--HPLVEG-----GYHPHIVYRRQKVPETKEPTCGLKD-   [212]
ADAMTS17    GGLVGLIQLGQEQVLIQPLNN--SQGPFS-----G-REHLIRRKWSLTPSPSAEAQRPEQ   [199]
ADAMTS19    -GLMGFIQLNEDFIFIEPLND--------TMAITG-HPHRVYRQKRSMEEKVTEKSALHS   [291]
ADAMTS2     --LAGLIRMEEEEFFIEPLEK--GLAAQEAEQ--G-RVHVVYRRPPTSPPLGGPQALDTG   [230]
ADAMTS3     --LAGMIKSDNEEYFIEPLER--GKQMEEE-K--G-RIHVVYKRSAVEQAPI--------   [211]
ADAMTS14    --LAGLIRTDSTDFFIEPLER--GQQEKEA-S--G-RTHVVYRREAVQQEWAEPDGDLHN   [225]
ADAMTS13    -----------YLSPGAPLK--------------GRPPSPGFQRQRQ-------------   [70]
dCG4096     ---VGHIRTAGNEYFIEPSKEHEPHP------VNG-HPHVVFQRSSVKPKHSLRKRNKRK   [225]
ADAMTS4     --LLGVLQYRGAELHLQPLEGGTPNSAGGP------GAHILRRKSP--------------   [186]
ADAMTS5     DGFFAVKHARYTLKPLLRGPWAEEEKGRVYGDGSARILHVYTREGFSFEALPPRASCE--   [210]
ADAMTS8     --LSGSFLLDGEEFTIQPQGA--GGSLA--------QPHRLQRWGPAGARPLPR------   [164]
ADAMTS1     --VRGAFYLLGEAYFIQPLPAASERLATAAPGEKPPAPLQFHLLRRNRQGDVGG--TCGV   [200]
ADAMTS15    --LRGAFGYRGAEYVISPLPN--ASA--PAAQRNSQGAHLLQRRGVPG------------   [164]
dCG6107     --MTGYIKTSFGALLIQPVNR--TSSDE--------VLHRVFRKSQRNARHAVSKFEL--   [315]
ADAMTS9     --MLGTFRSHDGDYFIEPLQS--MDEQEDEEEQN--KPHIIYRRSAPQREPSTGR-----   [220]
ADAMTS20    --LTGTFKGQNGEYFLEPIMK--ADGNEYED--GHNKPHLIYRQDLNNSFLQT-------   [182]
mask        -----------11111111------------------111111111--------------   [30]
Ce gon-1    --LYGMLALPSGIHTVEPIIS--GNGTEHD----GASRHRQHLVRKFDPMHFKSF-----   [239]
[Intron       8                        9       10             11           ]
```

```
[                    370       380       390       400       410       420]
[                     .         .         .         .         .         .]
[Modal, ≥80%, full maj.                                                                ]
ADAMTS6      SD-------------------------------------FTRSGKPWWLNDTSTVSYSLP   [232]
ADAMTS10     ---------------------------------------EKPWKGRPWWLRTLKPPPARP   [219]
ADAMTS18     RNYPGYSPSHIPHASQSRETEYHHRRLQKQHFCGRRKKYAPKPPTEDTYLRFDEYGS---   [278]
ADAMTS16     R-------TWELAHQPLHSSDLRLGLPQKQHFCGRRKKYMPQPPKEDLFILPDEYKS---   [273]
ADAMTS7      TCGVQV---------------------------------YPELESRR----ERWEQRQQW   [225]
ADAMTS12     ---------------------------------------SVNISQKQELWREKWERHNLP   [233]
ADAMTS17     LCKVLT-----E---------------------------KKKPTW--------GRPSRDW   [219]
ADAMTS19     HYCGIISD-------------------------------KGRPRSRKIA-----------   [309]
ADAMTS2      ---------------------------------------ASLDSLDSLSRALGVLEEHAN   [251]
ADAMTS3      --DMSKDFHYRE---------------------------SDLEGLDDLGTVYGNIHQQLN   [242]
ADAMTS14     EA-------------------------------------FGLGDLPNLLGLVGDQLG---   [245]
ADAMTS13     ------------------------------------------------------------   [70]
dCG4096      RGGKSGSGAEVSNCGTREPRRRMETRLEWQARGKVKVQGGRQIRRHHHHHHHHHHKHKYR   [285]
ADAMTS4      ------------------------------ASGQGPMCN-----VKAP----LGSPS--P   [205]
ADAMTS5      -TPAS-TPEAHEHAPAHSNP-------SGRAALASQL-----LDQSALSPAGGSGP-QTW   [255]
ADAMTS8      -----GP-EWEVETGEGQRQER-------GDHQEDSEEE----SQEEEAEGASEPPPPLG   [207]
ADAMTS1      VDDEPRP-TGKAETEDEDEGTE--------GEDEGPQWS-----PQDPALQGVGQPTG-T   [245]
ADAMTS15     -----GP-SGDPTSRCGVASGW--------NPAILRALD-----PYKPRRAGFGESRSRR   [205]
dCG6107      ---GLDDFMSKLEQVQEEEQKSKSRKLN--------------------------------   [340]
ADAMTS9      --------------------------------HACDTSEHKNRHSKDKKKTRARKWGERI   [248]
ADAMTS20     -------------------------------LKYCSVSESQIKETSLPFHTYSNMNEDLN   [211]
mask         ------------------------------------------------------------   [30]
Ce gon-1     ------------DHLNSTSVNETETTVATWQDQWEDVIERKA------------------   [269]
[Intron                                             12                 13]

[                    430       440       450       460       470       480]
[                     .         .         .         .         .         .]
[Modal, ≥80%, full maj.                       R  R                E L    D]
ADAMTS6      INNTHI--HH-------------------------RQKR------SVSIERFVETLVVAD   [259]
ADAMTS10     LGNETE--RGQP-----------------------GLKR------SVSRERYVETLVVAD   [248]
ADAMTS18     -SG--------------------------------RPRRSAG---KSQKGLNVETLVVAD   [302]
ADAMTS16     -CL--------------------------------RHKRSLLRS-HRNEELNVETLVVVD   [299]
ADAMTS7      RRP--------------------------------RLRRLHQR--SVSKEKWVETLVVAD   [251]
ADAMTS12     SRS--------------------------------LSRR------SISKERWVETLVVAD   [255]
ADAMTS17     -----------------------------------RERRNAI---RLTSEHTVETLVVAD   [241]
ADAMTS19     --------------------------------ESGRGKRYSY---KLPQEYNIETVVVAD   [334]
ADAMTS2      SS---------------------------------RRRARRH---AADDDYNIEVLLGVD   [275]
ADAMTS3      ETM--------------------------------RRRRH-----AGENDYNIEVLLGVD   [265]
ADAMTS14     DTE--------------------------------RKRRH-----AKPGSYSIEVLLVVD   [268]
ADAMTS13     -----------------------------------RQRR------AAGGILHLELLVAVG   [89]
dCG4096      HHQQKISRVPHTKFKYETQFQTE-----PDHAEIPRRRR------SISSPRHVETLIVAD   [334]
ADAMTS4      RPR--------------------------------RAKR------FASLSRFVETLVVAD   [227]
ADAMTS5      WR---------------------------------RRRR------SISRARQVELLLVAD   [276]
ADAMTS8      ATS--------------------------------RTKR------FVSEARFVETLLVAD   [229]
ADAMTS1      GSI--------------------------------RKKR------FVSSHRYVETMLVAD   [267]
ADAMTS15     RSG--------------------------------RAKR------FVSIPRYVETLVVAD   [227]
dCG6107      -----------------------------------RKKR--HYADVDNQVYTLEVLIAVD   [363]
ADAMTS9      NLAGDVAALNSGLATEAFSAYGNKTDNTREKRTHRRTKR------FLSYPRFVEVLVVAD   [302]
ADAMTS20     VMKERVLGHTSKNV------------PLKDERRHSRKKR------LISYPRYIEIMVTAD   [253]
mask         -----------------------------------1111------111111111111111   [49]
Ce gon-1     -----------------------------------RSRR-----AANSWDHYEVEVLVVAD   [289]
[Intron                                     ▲ 14                                ]
[                                furin prodomain cleavage motif               ]
```

```
[                    490       500       510       520       530       540]
[                    .         .         .         .         .         .]
[≥80%, full maj. HG      Y LT          S                              ]
ADAMTS6     KMMVGYHGRKDIEHYILSVMNIVAKLYRDSSLGNVVNIIVARLIVLTEDQPNLE-INH-H   [317]
ADAMTS10    KMMVAYHGRRDVEQYVLAIMNIVAKLFQDSSLGSTVNILVTRLILLTEDQPTLE-ITH-H   [306]
ADAMTS18    KKMVEKHGKGNVTTYILTVMNMVSGLFKDGTIGSDINVVVVSLILLEQEPGGLL-INH-H   [360]
ADAMTS16    KKMMQNHGHENITTYVLTILNMVSALFKDGTIGGNINIAIVGLILLEDEQPGLV-ISH-H   [357]
ADAMTS7     AKMVEYHGQPQVESYVLTIMNMVAGLFHDPSIGNPIHITIVRLVLLEDEEEDLK-ITH-H   [309]
ADAMTS12    TKMIEYHGSENVESYILTIMNMVTGLFHNPSIGNAIHIVVVRLILLEEEEQGLK-IVH-H   [313]
ADAMTS17    ADMVQYHGAEAAQRFILTVMNMVYNMFQHQSLGIKINIQVTKLVLLRQRPAKLS-IGH-H   [299]
ADAMTS19    PAMVSYHGADAARRFILTILNMVFNLFQHKSLGVQVNLRVIKLILLHETPPELY-IGH-H   [392]
ADAMTS2     DSVVQFHGKEHVQKYLLTLMNIVNEIYHDESLGAHINVVLVRIILLSYGKSMSL-IEIGN   [334]
ADAMTS3     DSVVRFHGKEHVQNYLLTLMNIVNEIYHDESLGVHINVVLVRMIMLGYAKSISL-IERGN   [324]
ADAMTS14    DSVVRFHGKEHVQNYVLTLMNIVDEIYHDESLGVHINIALVRLIMVGYRQSLSL-IERGN   [327]
ADAMTS13    PDVFQAHQE-DTERYVLTNLNIGAELLRDPSLGAQFRVHLVKMVILTEPEGAPN-ITA-N   [146]
dCG4096     ATMSAFHR--DLNGYLLTIMNMVSALYKDPSIGNSIEIVVVRIIQLDEEESQLQLNLTQN   [392]
ADAMTS4     DKMAAFHGA-GLKRYLLTVMAAAAKAFKHPSIRNPVSLVVTRLVILGSGEEGPQ-VGP-S   [284]
ADAMTS5     ASMARLYGR-GLQHYLLTLASIANRLYSHASIENHIRLAVVKVVVLGDKDKSLE-VSK-N   [333]
ADAMTS8     ASMAAFYGA-DLQNHILTLMSVAARIYKHPSIKNSINLMVVKVLIVEDEKWGPE-VSD-N   [286]
ADAMTS1     QSMAEFHGS-GLKHYLLTLFSVAARLYKHPSIRNSVSLVVVKILVIHDEQKGPE-VTS-N   [324]
ADAMTS15    ESMVKFHGA-DLEHYLLTLLATAARLYRHPSILNPINIVVVKVLLLRDRDSGPK-VTG-N   [284]
dCG6107     NSMKQFHGE-DLQPYILILMSIVSSIFADASIGNSIRILLVRLISLPNIN-----DQTHS   [417]
ADAMTS9     NRMVSYHGE-NLQHYILTLMSIVASIYKDPSIGNLINIVIVNLIVIHNEQDGPS-ISF-N   [359]
ADAMTS20    AKVVSAHGS-NLQNYILTLMSIVATIYKDPSIGNLIHIVVVKLVMIHREEEGPV-INF-D   [310]
mask        11111111--11111111111111111111111111111111111111-----------1   [96]
Ce gon-1    TKMYEYHGR-SLEDYVLTLFSTVASIYRHQSLRASINVVVVKLIVLKTENAGPR-ITQ-N   [346]
[Intron                 15        16                           17                ]
```

```
[                    550       560       570       580       590       600]
[                    .         .         .         .         .         .]
[≥80%, full maj.L   C W                          D A   TR D              ]
ADAMTS6     ADKSLDSFCKWQKSILSHQSDGNTIPENG----IAHHDNAVLITRYDICTYKNKP-CGTL   [372]
ADAMTS10    AGKSLDSFCKWQKSIVNHSGHGNAIPENG----VANHDTAVLITRYDICIYKNKP-CGTL   [361]
ADAMTS18    ADQSLNSFCQWQSALIGKNG--------------KRHDHAILLTGFDICSWKNEP-CDTL   [405]
ADAMTS16    ADHTLSSFCQWQSGLMGKDG--------------TRHDHAILLTGLDICSWKNEP-CDTL   [402]
ADAMTS7     ADNTLKSFCKWQKSINMKGDAHP-----------LHHDTAILLTRKDLCAAMNRP-CETL   [357]
ADAMTS12    AEKTLSSFCKWQKSINPKSDLNP-----------VHHDVAVLLTRKDICAGFNRP-CETL   [361]
ADAMTS17    GERSLESFCHWQNEEYGGARYLGNNQVPGGKDDPPLVDAAVFVTRTDFCVHKDEP-CDTV   [358]
ADAMTS19    GEKMLESFCKWQHEEFGKKNDIHLEMSTNWGEDMTSVDAAILITRKDFCVHKDEP-CDTV   [451]
ADAMTS2     PSQSLENVCRWAYLQQKPDTGHD-----------EYHDHAIFLTRQDFGP------SGMQ   [377]
ADAMTS3     PSRSLENVCRWASQQQRSDLNHS-----------EHHDHAIFLTRQDFGP------AGMQ   [367]
ADAMTS14    PSRSLEQVCRWAHSQQRQDPSHA-----------EHHDHVVFLTRQDFGP------SGMQ   [370]
ADAMTS13    LTSSLLSVCGWSQTINPEDDTDP-----------GHADLVLYITRFDLELPDGN--RQVR   [193]
dCG4096     AQKNLDRFCSWQHKLNKGSEKDP-----------HHHDVAILITRKNICANN----CMTL   [437]
ADAMTS4     AAQTLRSFCAWQRGLNTPEDSDP-----------DHFDTAILFTRQDLC-GVST--CDTL   [330]
ADAMTS5     AATTLKNFCKWQHQHNQLGDDHE-----------EHYDAAILFTREDLC-GHHS--CDTL   [379]
ADAMTS8     GGLTLRNFCNWQRRFNQPSDRHP-----------EHYDTAILLTRQNFC-GQEGL-CDTL   [333]
ADAMTS1     AALTLRNFCNWQKQHNPPSDRDA-----------EHYDTAILFTRQDLC-GSQT--CDTL   [370]
ADAMTS15    AALTLRNFCAWQKKLNKVSDKHP-----------EYWDTAILFTRQDLC-GATT--CDTL   [330]
dCG6107     SNEMLKHFCQFINQ------------------SGYERDTAMLITREPICGSVPGKICHML   [459]
ADAMTS9     AQTTLKNLCQWQHSKNSPGGI--------------HHDTAVLLTRQDIC-RAHDK-CDTL   [403]
ADAMTS20    GATTLKNFCSWQQTQNDLDDVHP-----------SHHDTAVLITREDIC-SSKEK-CNML   [357]
mask        11111111111111---------------------11111111111111-------1111   [128]
Ce gon-1    AQQTLQDFCRWQQYYNDPDDSSV-----------QHHDVAILLTRKDIC-RSQGK-CDTL   [393]
[Intron              18                                19,20         21          ]
```

```
[                   610       620       630       640       650       660]
[                    .         .         .         .         .         .]
[≥80%,maj.  G       C    SC      G   AFT AHE GH      HD       C          ]
ADAMTS6     GLASVAGMCEPERSCSINEDIGLGSAFTIAHEIGHNFGMNHDGIGNS-C-GTKG------   [424]
ADAMTS10    GLAPVGGMCERERSCSVNEDIGLPQAFTIAHEIGHTFGMNHDGVGNS-C-GARG------   [413]
ADAMTS18    GFAPISGMCSKYRSCTINEDTGLGLAFTIAHESGHNFGMIHDGEGNP-CRKAEG------   [458]
ADAMTS16    GFAPISGMCSKYRSCTINEDTGLGLAFTIAHESGHNFGMIHDGEGNM-CKKSEG------   [455]
ADAMTS7     GLSHVAGMCQPHRSCSINEDTGLPLAFTVAHELGHSFGIQHDGSGND-C-EPVG------   [409]
ADAMTS12    GLSHLSGMCQPHRSCNINEDSGLPLAFTIAHELGHSFGIQHDGKEND-C-EPVG------   [413]
ADAMTS17    GIAYLGGVCSAKRKCVLAEDNGLNLAFTIAHELGHNLGMNHDDDHSS-C-----------   [406]
ADAMTS19    GIAYLSGMCSEKRKCIIAEDNGLNLAFTIAHEMGHNMGINHDNDHPS-C-----------   [499]
ADAMTS2     GYAPVTGMCHPVRSCTLNHEDGFSSAFVVAHETGHVLGMEHDGQGNR-C----GD-----   [427]
ADAMTS3     GYAPVTGMCHPVRSCTLNHEDGFSSAFVVAHETGHVLGMEHDGQGNR-C----GD-----   [417]
ADAMTS14    GYAPVTGMCHPLRSCALNHEDGFSSAFVIAHETGHVLGMEHDGQGNG-C-----D-----   [419]
ADAMTS13    GVTQLGGACSPTWSCLITEDTGFDLGVTIAHEIGHSFGLEHDGAPGSGC-----------   [242]
dCG4096     GLANVGGMCKPKQSCSVNEDNGIMLSHTITHELGHNFGMFHDTAKIG-CHPRVGPIV---   [493]
ADAMTS4     GMADVGTVCDPARSCAIVEDDGLQSAFTAAHELGHVFNMLHDNSKP--CISLNGP-----   [383]
ADAMTS5     GMADVGTICSPERSCAVIEDDGLHAAFTVAHEIGHLLGLSHDDSKF--CEETFG------   [431]
ADAMTS8     GVADIGTICDPNKSCSVIEDEGLQAAHTLAHELGHVLSMPHDDSKP--CTRLFG------   [385]
ADAMTS1     GMADVGTVCDPSRSCSVIEDDGLQAAFTTAHELGHVFNMPHDDAKQ--CASLNG------   [422]
ADAMTS15    GMADVGTMCDPKRSCSVIEDDGLPSAFTTAHELGHVFNMPHDNVKV--CEEVFG------   [382]
dCG6107     GLAELGTVCS-SSSCSIVQDTGLPTAFTMAHELGHILNMNHDDDDK--CMPYVTRQN---   [513]
ADAMTS9     GLAELGTICDPYRSCSISEDSGLSTAFTIAHELGHVFNMPHDDNNK--C-KEEG------   [454]
ADAMTS20    GLSYLGTICDPLQSCFINEEKGLISAFTIAHELGHTLGVQHDDNPR--C-KEM-------   [407]
mask        111111111111111111111111111111111111111111111--1-----------   [175]
Ce gon-1    GLAELGTMCDMQKSCAIIEDNGLSAAFTIAHELGHVFSIPHDDERK--CSTYMPVNKVCK   [451]
[Intron     22                               23,24                         ]
[                                            catalytic Zn++ binding motif    ]

[                   670       680       690       700       710       720]
[                    .         .         .         .         .         .]
[Modal, ≥80%, full maj.      M              WS CS        L        CL    P]
ADAMTS6     -----------HEAAKLMAAHITA--NTNPFSWSACSRDYITSFLDSGRGT--CLDNE-P   [468]
ADAMTS10    -----------QDPAKLMAAHITM--KTNPFVWSSCNRDYITSFLDSGLGL--CLNNR-P   [457]
ADAMTS18    ---------------NIMSPTLTG--NNGVFSWSSCSRQYLKKFLSTPQAG--CLVDE-P   [498]
ADAMTS16    ---------------NIMSPTLAG--RNGVFSWSPCSRQYLHKFLSTAQAI--CLADQ-P   [495]
ADAMTS7     ------------KRPFIMSPQLLY--DAAPLTWSRCSRQYITRFLDRGWGL--CLDDP-P   [452]
ADAMTS12    ------------RHPYIMSRQLQY--DPTPLTWSKCSEEYITRFLDRGWGF--CLDDI-P   [456]
ADAMTS17    -----------AGRSHIMSGEWVKGRNPSDLSWSSCSRDDLENFLKSKVST--CLLVTDP   [453]
ADAMTS19    -----------ADGLHIMSGEWIKGQNLGDVSWSRCSKEDLERFLRSKASN--CLLQTNP   [546]
ADAMTS2     ----------EVRLGSIMAPLVQA--AFHRFHWSRCSQQELSRYLHS--YD--CLLDD-P   [470]
ADAMTS3     ----------ETAMGSVMAPLVQA--AFHRYHWSRCSGQELKRYIHS--YD--CLLDD-P   [460]
ADAMTS14    ----------ETSLGSVMAPLVQA--AFHRFHWSRCSKLELSRYLPS--YD--CLLDD-P   [462]
ADAMTS13    -----------GPSGHVMASDGAA--PRAGLAWSPCSRRQLLSLLSAGRAR--CVWDP-P   [286]
dCG4096     ---------------HIMTPTFGA--DTLQVCWSNCSRKYITHFLDQGLGE--CLDDP-P   [533]
ADAMTS4     ----------LSTSRHVMAPVMAH--VDPEEPWSPCSARFITDFLDNGYGH--CLLDK-P   [428]
ADAMTS5     ----------STEDKRLMSSILTS--IDASKPWSKCTSATITEFLDDGHGN--CLLDL-P   [476]
ADAMTS8     ----------PMGKHHVMAPLFVH--LNQTLPWSPCSAMYLTELLDGGHGD--CLLDA-P   [430]
ADAMTS1     ----------VNQDSHMMASMLSN--LDHSQPWSPCSAYMITSFLDNGHGE--CLMDK-P   [467]
ADAMTS15    ----------KLRANHMMSPTLIQ--IDRANPWSACSAAIITDFLDSGHGD--CLLDQ-P   [427]
dCG6107     ----------NNKVLHIMSSVMGI--HMHPWSWSKCSRHFVSEFLEKTDKS--CLETS-V   [558]
ADAMTS9     ----------VKSPQHVMAPLNF--YTNPWMWSKCSRKYITEFLDTGYGE--CLLNE-P   [499]
ADAMTS20    ----------KVTKYHVMAPALSF--HMSPWSWSNCSRKYVTEFLDTGYGE--CLLDK-P   [452]
mask        ---------------111111-----------111111111111111---11--1111--1   [203]
Ce gon-1    FQSTKFDKTQFQNNFHIMAPTLEY--NTHPWSWSPCSAGMLERFLENNRGQTQCLFDQ-P   [508]
[Intron                    25                          26,27,28            ]
```

```
[                    730       740       750       760       770       780]
[                     .         .         .         .         .         .]
[Modal, ≥80%, full maj.  PG     Y    QC    G     C           C  LWC      ]
ADAMTS6      ---PKRDFLYPAVAPG--QVYDADEQCRFQYGATSRQCKYG-----EVCRELWCLSK--S   [516]
ADAMTS10     ---PRQDFVYPTVAPG--QAYDADEQCRFQHGVKSRQCKYG-----EVCSELWCLSK--S   [505]
ADAMTS18     --KQAGQYKYPDKLPG--QIYDADTQCKWQFGAKAKLCSLG--FVKDICKSLWCHRV--G   [550]
ADAMTS16     --KPVKEYKYPEKLPG--ELYDANTQCKWQFGEKAKLCMLD-FKKADICKALWCHRI--G   [548]
ADAMTS7      ---AKDIIDFPSVPPG--VLYDVSHQCRLQYGAYSAFCEDM----DNVCHTLWCSV---G   [500]
ADAMTS12     ---KKKGLKSKVIAPG--VIYDVHHQCQLQYGPNATFCQEVE----NVCQTLWCSV---K   [504]
ADAMTS17     --RSQHTVRLPHKLPG--MHYSANEQCQILFGMNATFCRNME---HLMCAGLWCLVE--G   [504]
ADAMTS19     --QSVNSVMVPSKLPG--MTYTADEQCQILFGPLASFCQEMQ---HVICTGLWCKVE--G   [597]
ADAMTS2      --FAHDWPALP-QLPG--LHYSMNEQCRFDFGLGYMMCTA--FRTFDPCKQLWCSHPD-N   [522]
ADAMTS3      --FDHDWPKLP-ELPG--INYSMDEQCRFDFGVGYKMCTA--FRTFDPCKQLWCSHPD-N   [512]
ADAMTS14     --FDPAWPQPP-ELPG--INYSMDEQCRFDFGSGYQTCLA--FRTFEPCKQLWCSHPD-N   [514]
ADAMTS13     RPQPGSAGHPPDAQPG--LYYSANEQCRVAFGPKAVACTFA-REHLDMCQALSCHTDPLD   [343]
dCG4096      T--PLDEYNYTGELPG--MRYNARGQCRLQFNLTTDSEVGACSAPHEFCSTLWCKVNGE-   [588]
ADAMTS4      ----EAPLHLPVTFPG--KDYDADRQCQLTFGPDSRHCPQL----PPPCAALWCSGHLNG   [478]
ADAMTS5      ----RKQILGPEELPG--QTYDATQQCNLTFGPEYSVCPGM-----DVCARLWCAVVRQG   [525]
ADAMTS8      ----GAALPLPTGLPGRMALYQLDQQCRQIFGPDFRHCPNT--SAQDVCAQLWCHTDGAE   [484]
ADAMTS1      ----HNPIQLPGDLPG--TSYDANRQCQFTFGEDSKHCPDA----ASTCSTLWCTGTSGG   [517]
ADAMTS15     ----SKPISLPEDLPG--ASYTLSQQCELAFGVGSKPCPYM-----QYCTKLWCTGKAKG   [476]
dCG6107      ---GAHIPYGTERLPG--EIYSLDAQCQLSFGNDFGYCPTD-----EECKRLWCNRTSGN   [608]
ADAMTS9      ---ESRPYPLPVQLPG--ILYNVNKQCELIFGPGSQVCPY-----MMQCRRLWCNNVNGV   [549]
ADAMTS20     ---DEEIYNLPSELPG--SRYDGNKQCELAFGPGSQMCPHI----ENICMHLWCTSTEKL   [503]
mask         ----1111111-1111--111111111111111111111111------11111111111----   [246]
Ce gon-1     VERRYYEDVFVRDEPG--KKYDAHQQCKFVFGPASELCPYM-----PTCRRLWCATFYGS   [561]
[Intron                                    29,30,31                        ]

[                    790       800       810       820       830       840]
[                     .         .         .         .         .         .]
[≥80%, full maj.C       P    GT C          C  G C               G W   W     ]
ADAMTS6      NR--CVTNS--IPAAEGTLCQTGNIEKGWCYQGDCVPFGTW-PQSI--DGGWGPWSLWGE   [569]
ADAMTS10     NR--CITNS--IPAAEGTLCQTHTIDKGWCYKRVCVPFGSR-PEGV--DGAWGPWTPWGD   [558]
ADAMTS18     HR--CETKF--MPAAEGTVCGL----SMWCRQGQCVKFGELGPRPI--HGQWSAWSKWSE   [600]
ADAMTS16     RK--CETKF--MPAAEGTICGH----DMWCRGGQCVKYGDEGPKPT--HGHWSDWSSWSP   [598]
ADAMTS7      TT--CHSKL--DAAVDGTRCGE----NKWCLSGECVPVGFR-PEAV--DGGWSGWSAWSI   [549]
ADAMTS12     GF--CRSKL--DAAADGTQCGE----KKWCMAGKCITVGKK-PESI--PGGWGRWSPWSH   [553]
ADAMTS17     DTS-CKTKL--DPPLDGTECGA----DKWCRAGECVSKTPI-PEHV--DGDWSPWGAWSM   [554]
ADAMTS19     EKE-CRTKL--DPPMDGTDCDL----GKWCKAGECTSRTSA------PEHLAGEWSLWSP   [644]
ADAMTS2      PYF-CKTKK--GPPLDGTMCAP----GKHCFKGHCIWLT---PDILKRDGSWGAWSPFGS   [572]
ADAMTS3      PYF-CKTKK--GPPLDGTECAA----GKWCYKGHCMWK---NANQQKQDGNWGSWTKFGS   [562]
ADAMTS14     PYF-CKTKK--GPPLDGTECAP----GKWCFKGHCIWKS--PEQTYGQDGGWSSWTKFGS   [565]
ADAMTS13     QSS-CSRLL--VPLLDGTECGV----EKWCSKGRCRSLVELTP-IAAVHGRWSSWGPRSP   [395]
dCG4096      ----CVTHM--RPTAPGTLCGR----NKWCQNGKCVRREELAAV----NGGWGDWSEWSE   [634]
ADAMTS4      HAM-CQTKH--SPWADGTPCGP----AQACMGGRCLHMDQLQDFNIPQAGGWGPWGPWGD   [531]
ADAMTS5      QMV-CLTKK--LPAVEGTPCGK----GRICLQGKCVDKTKKKYYSTSSHGNWGSWGSWGQ   [578]
ADAMTS8      PL--CHTKNGSLPWADGTPCGP----GHLCSEGSCLPEEEVERPKPVVDGGWAPWGPWGE   [538]
ADAMTS1      VLV-CQTKH--FPWADGTSCGE----GKWCINGKCVNKTDRKHFDTPFHGNWGMWGPWGD   [570]
ADAMTS15     QMV-CQTRH--FPWADGTSCGE----GKLCLKGACVERHNLNKHRV--DGSWAKWDPYGP   [527]
dCG6107      SNEQCASSN--LPWADGTPCGSS---GHWCQRGKCVSNKHGY--GRQVNGGWGPWTPFTP   [661]
ADAMTS9      HKG-CRTQH--TPWADGTECEP----GKHCKYGFCVPKEMDVPV-T--DGSWGSWSPFGT   [599]
ADAMTS20     HKG-CFTQH--VPPADGTDCGP---GMVHCRHGLCVNKETE---TRPVNGEWGPWEPYSS   [554]
mask         ----11111--1111111111-----1111111111111---------111111111111   [285]
Ce gon-1     QMG-CRTQH--MPWADGTPCDESR--SMFCHHGACVRLAPESL--TKIDGQWGDWRSWGE   [614]
[Intron                           32                 33      ▲             ]
[                                                           Thrombospondin→    ]
```

```
[                     850       860       870       880       890       900]
[                       .         .         .         .         .         .]
[≥80%, maj. CSR CG G      R C   P P   GG   C G        C    C          FR  QC  ]
ADAMTS6     CSRTCGGGVSSSLRHCDSPAPSGGGKYCLGERKRYRSCNTDPCPL---GSRDFREKQCAD    [626]
ADAMTS10    CSRTCGGGVSSSSRHCDSPRPTIGGKYCLGERRRHRSCNTDDCPP---GSQDFREVQCSE    [615]
ADAMTS18    CSRTCGGGVKFQERHCNNPKPQYGGLFCPGSSRIYQLCNINPCNE---NSLDFRAQQCAE    [657]
ADAMTS16    CSRTCGGGVSHRSRLCTNPKPSHGGKFCEGSTRTLKLCNSQKCPR---DSVDFRAAQCAE    [655]
ADAMTS7     CSRSCGMGVQSAERQCTQPTPKYKGRYCVGERKRFRLCNLQACPA---GRPSFRHVQCSH    [606]
ADAMTS12    CSRTCGAGVQSAERLCNNPEPKFGGKYCTGERKRYRLCNVHPCRS---EAPTFRQMQCSE    [610]
ADAMTS17    CSRTCGTGARFRQRKCDNPPPGPGGTHCPGASVEHAVCENLPCPK---GLPSFRDQQCQA    [611]
ADAMTS19    CSRTCSAGISSRERKC--PGLDSEARDCNGPRKQYRICENPPCPA---GLPGFRDWQCQA    [699]
ADAMTS2     CSRTCGTGVKFRTRQCDNPHPANGGRTCSGLAYDFQLCSRQDCPD---SLADFREEQCRQ    [629]
ADAMTS3     CSRTCGTGVRFRTRQCNNPMPINGGQDCPGVNFEYQLCNTEECQK---HFEDFRAQQCQQ    [619]
ADAMTS14    CSRSCGGGVRSRSRSCNNPSPAYGGRLCLGPMFEYQVCNSEECPG---TYEDFRAQQCAK    [622]
ADAMTS13    CSRSCGGGVVTRRRQCNNPRPAFGGRACVGADLQAEMCNTQACEK---TQLEFMSQQCAR    [452]
dCG4096     CSRSCGGGVSTQQRECDNPVPANGGVFCIGERKRYKICRKRPCPA---EEPSFRAQQCAR    [691]
ADAMTS4     CSRTCGGGVQFSSRDCTRPVPRNGGKYCEGRRTRFRSCNTEDCPT--GSALTFREEQCAA    [589]
ADAMTS5     CSRSCGGGVQFAYRHCNNPAPRNNGRYCTGKRAIYRSCSLMPCPP---NGKSFRHEQCEA    [635]
ADAMTS8     CSRTCGGGVQFSHRECKDPEPQNGGRYCLGRRAKYQSCHTEECPP---DGKSFREQQCEK    [595]
ADAMTS1     CSRTCGGGVQYTMRECDNPVPKNGGKYCEGKRVRYRSCNLEDCPD--NNGKTFREEQCEA    [628]
ADAMTS15    CSRTCGGGVQLARRQCTNPTPANGGKYCEGVRVKYRSCNLEPCPSS-ASGKSFREEQCEA    [586]
dCG6107     CSLTCGGGVQESRRECNQPVPENGGKYCTGSRKKYRSCNTHQCPP---GSMDPREQQCYA    [718]
ADAMTS9     CSRTCGGGIKTAIRECNRPEPKNGGKYCVGRRMKFKSCNTEPCLK---QKRDFRDEQCAH    [656]
ADAMTS20    CSRTCGGGIESATRRCNRPEPRNGGNYCVGRRMKFRSCNTDSCPK---GTQDFREKQCSD    [611]
mask        111111111111111111111111111111111111111111111----111111111111    [341]
Ce gon-1    CSRTCGGGVQKGLRDCDSPKPRNGGKYCVGQRERYRSCNTQECPW---DTQPYREVQCSE    [671]
[Intron                       34                    35     36,37,38              ]

[                     910       920       930       940       950       960]
[                       .         .         .         .         .         .]
[Modal, ≥80%, full maj.      W              C L C                V DGT C    ]
ADAMTS6     FDNM--PFRGKYY---NWKPYTGG---G-VKPCALNCLAEGYNFYTERAPAVIDGTQCNA    [677]
ADAMTS10    FDSI--PFRGKFY---KWKTYRGG---G-VKACSLTSLAEGFNFYTERAAAVVDGTPCRP    [666]
ADAMTS18    YNSK--PFRGWFY---QWKPYTKVEE-E--DRCKLYCKAENFEFFFAMSGKVKDGTPCSP    [709]
ADAMTS16    HNSR--RFRGRHY---KWKPYTQVED-Q--DLCKLYCIAEGFDFFFSLSNKVKDGTPCSE    [707]
ADAMTS7     FDAM--LYKGQLH---TWVPVVND-----VNPCELHCRPANEYFAKKLRDAVVDGTPCYQ    [656]
ADAMTS12    FDTV--PYKNELY---HWFPIFNP-----AHPCELYCRPIDGQFSEKMLDAVIDGTPCFE    [660]
ADAMTS17    HDR---LSPKKKG---LLTAVVVDD--K---PCELYCSPLGKESPLLVADRVLDGTPCGP    [660]
ADAMTS19    YSVR-TSSPKHIL---QWQAVLDEE--K---PCALFCSPVGKEQPILLSEKVMDGTSCGY    [750]
ADAMTS2     WDLY-FEHGDAQH---HWLPHEHRDA-K--ERCHLYCESRETGEVVSMKRMVHDGTRCSY    [682]
ADAMTS3     RNSH-FEYQNTKH---HWLPYEHPDP-K--KRCHLYCQSKETGDVAYMKQLVHDGTHCSY    [672]
ADAMTS14    RNSY-YVHQNAKH---SWVPYEPDDD-A--QKCELICQSADTGDVVFMNQVVHDGTRCSY    [675]
ADAMTS13    TDGQPLRSSPGGASFYHWGAAVPHSQGD--ALCRHMCRAIGESFIMKRGDSFLDGTRCMP    [510]
dCG4096     FDNV--SYQGATYK---WLPFFDKN--N---PCKLFCSDVDDTIIANWGATVLDGTPCTL    [741]
ADAMTS4     YNHR-TDLFKSFPGPMDWVPRYTGVA-P-QDQCKLTCQARALGYYYVLEPRVVDGTPCSP    [646]
ADAMTS5     KNGY-QSDAKGVKTFVEWVPKYAGVL-P-ADVCKLTCRAKGTGYYVVFSPKVTDGTECRP    [692]
ADAMTS8     YNAY-NYTDMDGN-LLQWVPKYAGVS-P-RDRCKLFCRARGRSEFKVFEAKVIDGTLCGP    [651]
ADAMTS1     HNEF-SKASFGSGPAVEWIPKYAGVS-P-KDRCKLICQAKGIGYFFVLQPKVVDGTPCSP    [685]
ADAMTS15    FNGY-NHSTNRLTLAVAWVPKYSGVS-P-RDKCKLICRANGTGYFYVLAPKVVDGTLCSP    [643]
dCG6107     MNGRN-MNIPGVNPDTKWVPKYEK---D---ACKLFCRMDMKVTYFMLKSMVTDGTSCAV    [771]
ADAMTS9     FDGK-HFNINGLLPNVRWVPKYSGI--LMKDRCKLFCRVAGNTAYYQLRDRVIDGTPCGQ    [713]
ADAMTS20    FNGK-HLDISGIPSNVRWLPRYSGI--GTKDRCKLYCQVAGTNYFYLLKDMVEDGTPCGT    [668]
mask        11--------------11111111-------1111111111111111111111111111111    [380]
Ce gon-1    FNNKDIGIQGVASTNTHWVPKYANVAPN--ERCKLYCRLSGSAAFYLLRDKVVDGTPCDR    [729]
[Intron                                   39                      40             ]
```


AC Nicholson, SB Malik, JM Logsdon Jr, E van Meir, 2004. Functional evolution of ADAMTS genes: evidence]
[from analyses of phylogeny and gene organization. Contact: john-logsdon@uiowa.edu]
[Intron phases marked 0, 1, 2]

```
[                    970       980       990       1000      1010      1020]
[                    .         .         .         .         .         .]
[≥80%, full maj.       C  G C  GCD    S    D C VC G    C    G           ]
ADAMTS6     ------DSLDICINGECKHVGCDNILGSDAREDRCRVCGGDGSTCDAIEGFFNDSLPR--   [729]
ADAMTS10    ------DTVDICVSGECKHVGCDRVLGSDLREDKCRVCGGDGSACETIEGVFSPASPG--   [718]
ADAMTS18    ------NKNDVCIDGVCELVGCDHELGSKAVSDACGVCKGDNSTCKFYKGLYLNQHKA--   [761]
ADAMTS16    ------DSRNVCIDGICERVGCDNVLGSDAVEDVCGVCNGNNSACTIHRGLYTKHHHT--   [759]
ADAMTS7     VR----ASRDLCINGICKNVGCDFEIDSGAMEDRCGVCHGNGSTCHTVSGTFEEAE-G--   [709]
ADAMTS12    GG----NSRNVCINGICKMVGCDYEIDSNATEDRCGVCLGDGSSCQTVRKMFKQKE-G--   [713]
ADAMTS17    ------YETDLCVHGKCQKIGCDGIIGSAAKEDRCGVCSGDGKTCHLVKGDFSHAR-G--   [711]
ADAMTS19    ------QGLDICANGRCQKVGCDGLLGSLAREDHCGVCNGNGKSCKIIKGDFNHTR-G--   [801]
ADAMTS2     K-----DAFSLCVRGDCRKVGCDGVIGSSKQEDKCGVCGGDNSHCKVVKGTFTRSPKK--   [735]
ADAMTS3     K-----DPYSICVRGECVKVGCDKEIGSNKVEDKCGVCGGDNSHCRTVKGTFTRTPRK--   [725]
ADAMTS14    R-----DPYSVCARGECVPVGCDKEVGSMKADDKCGVCGGDNSHCRTVKGTLGKASKQ--   [728]
ADAMTS13    SGPREDGTLSLCVSGSCRTFGCDGRMDSQQVWDRCQVCGGDNSTCSPRKGSFTAGRA---   [567]
dCG4096     ------GTNNMCIDGICKKVGCDWIVDSEVQDDRCGVCGGSGDQCQPVRETYTDPFAA--   [793]
ADAMTS4     ------DSSSVCVQGRCIHAGCDRIIGSKKKFDKCMVCGGDGSGCSKQSGSFRKFR----   [696]
ADAMTS5     ------YSNSVCVRGKCVRTGCDGIIGSKLQYDKCGVCGGDNSSCTKIVGTFNKKS----   [742]
ADAMTS8     ------ETLAICVRGQCVKAGCDHVVDSPRKLDKCGVCGGKGNSCRKVSGSLTPTN----   [701]
ADAMTS1     ------DSTSVCVQGQCVKAGCDRIIDSKKKFDKCGVCGGNGSTCKKISGSVTSAK----   [735]
ADAMTS15    ------DSTSVCVQGKCIKAGCDGNLGSKKRFDKCGVCGGDNKSCKKVTGLFTKPM----   [693]
dCG6107     ------DSFDKCVNGICRPAGCDNELNSIAKLDKCGVCEGRNDTCHEVTGNLLVSNLLGL   [825]
ADAMTS9     ------DTNDICVQGLCRQAGCDHVLNSKARRDKCGVCGGDNSSCKTVAGTFNTVH----   [763]
ADAMTS20    ------ETHDICVQGQCMAAGCDHVLNSSAKIDKCGVCGGDNSSCKTITGVFNSSH----   [718]
mask        -------111111111111111111111111111111111111111111111111-----   [428]
Ce gon-1    ------NGDDICVAGACMPAGCDHQLHSTLRRDKCGVCGGDDSSCKVVKGTFNEQG-T--   [780]
[Intron                      41,42          43                                 ]

[                    1030      1040      1050      1060      1070      1080]
[                    .         .         .         .         .         .]
[≥80%, full maj.     Y     IP G                                  N      ]
ADAMTS6     -------GGYMEVVQIPRGSVHIEVREVAMSKN-----YIALKSE--GDDYYINGAWTID   [775]
ADAMTS10    -------AGYEDVVWIPKGSVHIFIQDLNLSLS-----HLALKGD--QESLLLEGLPGTP   [764]
ADAMTS18    -------NEYYPVVLIPAGARSIEIQELQVSSS-----YLAVRSL--SQKYYLTGGWSID   [807]
ADAMTS16    -------NQYYHMVTIPSGARSIRIYEMNVSTS-----YISVRNA--LRRYYLNGHWTVD   [805]
ADAMTS7     -------LGYVDVGLIPAGAREIRIQEVAEAAN-----FLALRSED-PEKYFLNGGWTIQ   [756]
ADAMTS12    -------SGYVDIGLIPKGARDIRVMEIEGAGN-----FLAIRSED-PEKYYLNGGFIIQ   [760]
ADAMTS17    -------TGYIEAAVIPAGARRIRVVEDKPAHS-----FLALKDS---GKGSINSDWKIE   [756]
ADAMTS19    -------AGYVEVLVIPAGARRIKVVEEKPAHS-----YLALRDA---GKQSINSDWKIE   [846]
ADAMTS2     -------HGYIKMFEIPAGARHLLIQEVDATSH-----HLAVKNLE-TGKFILNEENDVD   [782]
ADAMTS3     -------LGYLKMFDIPPGARHVLIQEDEASPH-----ILAIKNQA-TGHYILNGKG-EE   [771]
ADAMTS14    -------AGALKLVQIPAGARHIQIEALEKSPH-----RIVVKNQV-TGSFILNPKG-KE   [774]
ADAMTS13    -------REYVTFLTVTPNLTSVYIANHRPLFT-----HLAVRI---GGRYVVAGKMSIS   [612]
dCG4096     -----KDGAYVEIVTIPARARHILIRELANSPH-----FLAIATGDGGDRFYLNGDSLIS   [843]
ADAMTS4     -------YGYNNVVTIPAGATHILVRQQGNPGHRS--IYLALKLP--DGSYALNGEYTLM   [745]
ADAMTS5     -------KGYTDVVRIPEGATHIKVRQFKAKDQTRFTAYLALKKK--NGEYLINGKYMIS   [793]
ADAMTS8     -------YGYNDIVTIPAGATNIDVKQRSHPGVQNDGNYLALKTA--DGQYLLNGNLAIS   [752]
ADAMTS1     -------PGYHDIITIPTGATNIEVKQRNQRGSRNNGSFLAIKAA--DGTYILNGDYTLS   [786]
ADAMTS15    -------HGYNFVVAIPAGASSIDIRQRGYKGLIGDDNYLALKNS--QGKYLLNGHFVVS   [744]
dCG6107     NDGNEPNKTLYYVTRIPKGASNIIITQRGYPDQ-----NFIVLTDDR-DNELLNGKFLKT   [879]
ADAMTS9     -------YGYNTVVRIPAGATNIDVRQHSFSGETDDDNYLALSSS--KGEFLLNGNFVVT   [814]
ADAMTS20    -------YGYNVVVKIPAGATNVDIRQYSYSGQPDDS-YLALSDA--EGNFLFNGNFLLS   [768]
mask        -------11111111111111111111111111-----111111----111111111-11   [471]
Ce gon-1    -------FGYNEVMKIPAGSANIDIRQKGYNNMKEDDNYLSLRAA--NGEFLLNGHFQVS   [831]
[Intron           44,45       46                    47                         ]
```

AC Nicholson, SB Malik, JM Logsdon Jr, E van Meir, 2004. Functional evolution of ADAMTS genes: evidence]
[from analyses of phylogeny and gene organization. Contact: john-logsdon@uiowa.edu]
[Intron phases marked 0, 1, 2]

```
[                     1090      1100      1110      1120      1130      1140]
[                     .         .         .         .         .         .]
[≥80%, full maj.        G       Y       E                                  ]
ADAMTS6     WPRK---FDVAG--TAFHYKRPTDEPESLE-----ALGPTSENLIVMVLLQ---------   [816]
ADAMTS10    QPHR---LPLAG--TTFQLRQGPDQVQSLE-----ALGPINASLIVMVLAR---------   [805]
ADAMTS18    WPGE---FPFAG--TTFEYQRSFNRPERLY-----APGPTNETLVFEILMQ---------   [848]
ADAMTS16    WPGR---YKFSG--TTFDYRRSYNEPENLI-----ATGPTNETLIVELLFQ---------   [846]
ADAMTS7     WNGD---YQVAG--TTFTYARR-GNWENLT-----SPGPTKEPVWIQLLFQ---------   [796]
ADAMTS12    WNGN---YKLAG--TVFQYDRK-GDLEKLM-----ATGPTNESVWIQLLFQ---------   [800]
ADAMTS17    LPGE---FQIAG--TTVRYVRR-GLWEKIS-----AKGPTKLPLHLMVLLFH--------   [797]
ADAMTS19    HSGA---FNLAG--TTVHYVRR-GLWEKIS-----AKGPTTAPLHLLVLLFQ--------   [887]
ADAMTS2     ASSK--TFIAMG--VEWEYRDE-DGRETLQ-----TMGPLHGTITVLVIPVG--------   [824]
ADAMTS3     AKSR--TFIDLG--VEWDYNIE-DDIESLH-----TDGPLHDPVIVLIIPQEN-------   [814]
ADAMTS14    ATSR--TFTAMG--LEWEDAVE-DAKESLK-----TSGPLPEAIAILALPPTEG------   [818]
ADAMTS13    PNTTYPSLLEDG---RVEYRVALTEDRLPRLEEIRIWGPLQEDADIQVYRRYGEEYGN--   [667]
dCG4096     MPGE---FEIAG--AESLYDRV-DEQETIT-----IPQPIQHSISLYAIVRGN-------   [885]
ADAMTS4     PSPT--DVVLPGA-VSLRYSGATAASETLS-----GHGPLAQPLTLQVLVAGN-------   [790]
ADAMTS5     TSET--IIDING--TVMNYSGWSHRDDFLHGM---GYSATKEILIVQILATDP-------   [839]
ADAMTS8     AIEQ--DILVKG--TILKYSGSIATLERLQ-----SFRPLPEPLTVQLLTVPGE------   [797]
ADAMTS1     TLEQ--DIMYKG--VVLRYSGSSAALERIR-----SFSPLKEPLTIQVLTVGN-------   [830]
ADAMTS15    AVER--DLVVKG--SLLRYSGTGTAVESLQ-----ASRPILEPLTVEVLSVGK-------   [788]
dCG6107     YPLK---FVYAG--VTMQYTGSSSVVEQVN---TTYSWKLSRDLIVQIISLDV-------   [931]
ADAMTS9     MAKR--E-IRIG-NAVVEYSGSETAVERIN-----STDRIEQELLLQVLSVGK-------   [858]
ADAMTS20    TSKK--EINVQGTRTVIEYSGSNNAVERIN-----STNRQEKELILQVLCVGN-------   [814]
mask        1111-----111----111111-1111111-----11111111111111-----------   [505]
Ce gon-1    LARQ---QIAFQD-TVLEYSGSDAIIERIN-----GTGPIRSDIYVHVLSVG--------   [874]
[Intron                                               48                  ]
```

```
[                     1150      1160      1170      1180      1190      1200]
[                     .         .         .         .         .         .]
[Modal, ≥80%, full maj.                Y                                   ]
ADAMTS6     ------------------EQNLGIRYKFNVPITRTGSGDNEVGFT---------------   [843]
ADAMTS10    ------------------TELPALRYRFNAPIARDSLPPYS-------------------   [828]
ADAMTS18    ------------------GKNPGIAWKYALPKVMNGTPPATKRPAYT-------------   [877]
ADAMTS16    ------------------GRNPGVAWEYSMPRLGTEKQPPAQPSYT--------------   [874]
ADAMTS7     ------------------ESNPGVHYEYTIHREAGGHDEVPPPVFS--------------   [824]
ADAMTS12    ------------------VTNPGIKYEYTIQKDGLDNDVEQMYF----------------   [826]
ADAMTS17    ------------------DQDYGIHYEYTVPVNRTAENQSEPEKPQDSLFI---------   [830]
ADAMTS19    ------------------DQNYGLHYEYTIPSDPLPENQSSKAPEPLFM-----------   [918]
ADAMTS2     ------------------DTRVSLTYKYMIHEDSLN-VDDNNVLEEDSVVYE--------   [857]
ADAMTS3     ------------------DTRSSLTYKYIIHEDSVPTINSNNVIQEELDTFE--------   [848]
ADAMTS14    ------------------GPRSSLAYKYVIHEDLLPLIGSNNVLLEEMDTYE--------   [852]
ADAMTS13    ------------------LTRPDITFTYFQPKPRQAWV----------------------   [687]
dCG4096     ------------------ESNAGIFYEFTLPALNVTAGRQFQ------------------   [909]
ADAMTS4     ------------------PQDTRLRYSFFVPRPTPSTPRPTPQDWLHRRAQILEILRRRP   [832]
ADAMTS5     ------------------TKPLDVRYSFFVPKKSTPKVNS-VTSHGSNKVGSHTSQPQ--   [878]
ADAMTS8     ------------------VFPPKVKYTFFVPNDVDFSMQS-SKERATTNIIQPLLH-AQ-   [836]
ADAMTS1     ------------------ALRPKIKYTYFVKKKKESFNAIPTFSA---------------   [857]
ADAMTS15    ------------------MTPPRVRYSFYLPKEPREDKSSHPKDPRGPSVLHNSVLSLSN   [830]
dCG6107     -------------SPSKRQDTVLLSYSYTIDKPPDYEAEVEIYR----------------   [975]
ADAMTS9     ------------------LYNPDVRYSFNIPIEDKPQQFY--------------------   [880]
ADAMTS20    ------------------LYNPDVHYSFNIPLEERSDMFT--------------------   [836]
mask        ------------------111111111111111111------------------------   [523]
Ce gon-1    ------------------SHPPDISYEYMTAAVPNAVIRPISSALYL-------------   [903]
```

```
[                    1210      1220      1230      1240      1250      1260]
[                    .         .         .         .         .         .]
[≥80%, full maj.        W         C   C  G       C               C      P]

ADAMTS6      ------------WNHQP-WSECSATCAGGVQRQEVVCKRLDDNSIVQNNY--CDPDSK-P   [887]
ADAMTS10     ------------WHYAP-WTKCSAQCAGGSQVQAVECRNQLDSSAVAPHY--CSAHSK-L   [872]
ADAMTS18     ------------WSIVQ--SECSVSCGGGYINVKAICLRDQNTQVNSSF---CSAKTK-P   [919]
ADAMTS16     ------------WAIVR--SECSVSCGGGQMTVREGCYRDLKFQVNMSF---CNPKTR-P   [916]
ADAMTS7      ------------WHYGP-WTKCTVTCGRGVQRQNVYCLERQAGPVDEEH---CDPLGR-P   [867]
ADAMTS12     ------------WQYGH-WTECSVTCGTGIRRQTAHCIKKGRGMVKATF---CDPETQ-P   [869]
ADAMTS17     ------------WTHSG-WEGCSVQCGGGERRTIVSCTRIVNKTTTLVNDSDCPQASR-P   [876]
ADAMTS19     ------------WTHTS-WEDCDATCGGGERKTTVSCTKIMSKNISIVDNEKCKYLTK-P   [964]
ADAMTS2      ------------WALKK-WSPCSKPCGGGSQFTKYGCRRRLDHKMVHRGF--CAALSK-P   [901]
ADAMTS3      ------------WALKS-WSQVSKPCGGGFQYTKYGCRRKSDNKMVHRSF--CEANKK-P   [892]
ADAMTS14     ------------WALKS-WAPCSKACGGGIQFTKYGCRRRRDHHMVQRHL--CDHKKR-P   [896]
ADAMTS13     ------------WAAVR--GPCSVSCGAGLRWVNYSCLDQARKELVETVQ--CQGSQQ-P   [730]
dCG4096      ------------WRLSN-WTACSASCGGGVQHREPICQENGKGESNEPFQ--RIVSIK-R   [953]
ADAMTS4      ------------WAGRK-------------------------------------------   [837]
ADAMTS5      ------------WVTGP-WLACSRTCDTGWHTRTVQCQDGNRKLAKG-----CPLSQR-P   [919]
ADAMTS8      ------------WVLGD-WSECSSTCGAGWQRRTVECRDPSGQASAT-----CNKALK-P   [877]
ADAMTS1      ------------WVIEE-WGECSKSCELGWQRRLVECRDINGQPASE-----CAKEVK-P   [898]
ADAMTS15     QVEQPDDRPPARWVAGS-WGPCSASCGSGLQKRAVDCRGSAGQRTVPA----CDAAHR-P   [884]
dCG6107      ------------WEMQA-PSNCDSLCE-GRSHRLPACISTTQGVKVAPQF--CDKSAM-P   [1018]
ADAMTS9      ------------WNSHGPWQACSKPCQ-GERKRKLVCTRESDQLTVSDQR--CDRLPQ-P   [924]
ADAMTS20     ------------WDPYGPWEGCTKMCQ-GLQRRNITCIHKSDHSVVSDKE--CDHLPL-P   [880]
mask         ------------11111-------------------------------------------   [528]
Ce gon-1     ------------WRVTDTWTECDRACR-GQQSQKLMCLDMSTHRQSHDRN--CQNVLK-P   [947]
[Intron                          49                                         ]

[                    1270      1280      1290      1300      1310      1320]
[                    .         .         .         .         .         .]
[≥80%, full maj.  C    C                                                   ]
ADAMTS6      PENQRACNTEPCPPE-WFIGDWLECSKTCDGGMRTRAVL-CIRKIGPSEEETLDYSG-CL   [944]
ADAMTS10     PKRQRACNTEPCPPD-WVVGNWSLCSRSCDAGVRSRSVV-CQRRVSAAEEKALDDSA-CP   [929]
ADAMTS18     VTEPKICNAFSCPAY-WMPGEWSTCSKACAGGQQSRKIQ-CVQKKPFQKEEAVLHSL-CP   [976]
ADAMTS16     VTGLVPCKVSACPPS-WSVGNWSACSRTCGGGAQSRPVQ-C-TRRVHYDSEPVPASL-CP   [972]
ADAMTS7      DDQQRKCSEQPCPAR-WWAGEWQLCSSSCGPGGLSRRAVLCIRSVGLDEQSALEPPA-CE   [925]
ADAMTS12     NGRQKKCHEKACPPR-WWAGEWEACSATCGPHGEKKRTVLCIQTMVSDEQ-ALPPTD-CQ   [926]
ADAMTS17     EPQVRRCNLHPCQSR-WVAGPWSPCSATCEKGFQHREVT-CVYQLQNGTHVATRPLY-CP   [933]
ADAMTS19     EPQIRKCNEQPCQTR-WMMTEWTPCSRTCGKGMQSRQVA-CTQQLSNGTLIRARERD-CI   [1021]
ADAMTS2      KAIRRACNPQECSQPVWVTGEWEPCSQTCGRTGMQVRSVRCIQPLHDNTTRSVHAKH-CN   [960]
ADAMTS3      KPIRRMCNIQECTHPLWVAEEWEHCTKTCGSSGYQLRTVRCLQPLLDGTNRSVHSKY-CM   [951]
ADAMTS14     KPIRRRCNQHPCSQPVWVTEEWGACSRSCGKLGVQTRGIQCLLPLSNGTHKVMPAKA-CA   [955]
ADAMTS13     PAWPEACVLEPCPPY-WAVGDFGPCSASCGGGLRERPV-RCVEAQGSLLKTLPPAR--CR   [786]
dCG4096      NLLIALGDTLPC----WTHAKN-KR-PARQSRG---------------------------   [980]
ADAMTS4      ------------------------------------------------------------   [837]
ADAMTS5      SAFKQ-CLLKKC------------------------------------------------   [930]
ADAMTS8      EDAKP-CESQLCPL----------------------------------------------   [890]
ADAMTS1      ASTRP-CADHPCPQ--WQLGEWSSCSKTCGKGYKKRSLK-CLSHDGGVLSHES-----CD   [949]
ADAMTS15     VETQA-CGEP-CPT--WELSAWSPCSKSCGRGFQRRSLK-CVGHGGRLLARDQ-----CN   [934]
dCG6107      KIDDRACNTD-CRLN-LTVTSISECSAACGELGTREKTYACVQTFTNMQRSNIVDMSYCK   [1076]
ADAMTS9      GHITEPCGTD-CDLR-WHVASRSECSAQCGLGYRTLDIY-CAKYSRLDGKTEKVDDGFCS   [981]
ADAMTS20     SFVTQSCNTD-CELR-WHVIGKSECSSQCGQGYRTLDIH-CMKYSIHEGQTVQVDDHYCG   [937]
mask         ------------------------------------------------------------   [528]
Ce gon-1     KQATRMCNID-CSTR-WITEDVSSCSAKCGSGQKRQRVS-CVKMEGDRQTPASEHL--CD   [1002]
[Intron                  50                                                  ]
```

```
[                    1330      1340      1350      1360      1370      1380]
[                    .         .         .         .         .         .]
[≥80%, full maj.       C     C                                         ]
ADAMTS6      THRPVEKEP---CNNQS-CPPQ-WVALDWSECTPKCGP-GFKHRIVLCKSSDLSKTFPAA   [998]
ADAMTS10     QPRPPVLEA---CHGPT-CPPE-WAALDWSECTPSCGP-GLRHRVVLCKSADHRATLPPA   [983]
ADAMTS18     VSTPTQVQA---CNSHA-CPPQ-WSLGPWSQCSKTCGR-GVRKRELLCKGSAAETLPESQ   [1030]
ADAMTS16     QPAPSSRQA---CNSQS-CPPA-WSAGPWAECSHTCGK-GWRKRAVACKSTNPSARAQLL   [1026]
ADAMTS7      HLPRPPTETP--CNRHVPCPAT-WAVGNWSQCSVTCGE-GTQRRNVLCTNDTGVP-----   [976]
ADAMTS12     HLLKPKTLLS--CNRDILCPSD-WTVGNWSECSVSCGG-GVRIRSVTCAKNHDEP-----   [977]
ADAMTS17     GPRPAAVQS---CEGQD-CLSI-WEASEWSQCSASCGK-GVWKRTVACTNSQGK------   [981]
ADAMTS19     GPKPASAQR---CEGQD-CMTV-WEAGVWSECSVKCGK-GIRHRTVRCTNPRKK------   [1069]
ADAMTS2      DARPESRRA---CSREL-CPGR-WRAGPWSQCSVTCGN-GTQERPVPCRTADDSFGI---   [1011]
ADAMTS3      GDRPESRRP---CNRVP-CPAQ-WKTGPWSECSVTCGE-GTEVRQVLCRAGDH-------   [1005]
ADAMTS14     GDRPEARRP---CLRVP-CPAQ-WRLGAWSQCSATCGE-GIQQRQVVCRTNANSLGH---   [1006]
ADAMTS13     AGAQQPAVALETCNPQP-CPAR-WEVSEPSSCTSAGGA-GLALENETCVPGADGLEAPVT   [843]
dCG4096      ------------CGDQP-CPAH-WWPGPWQFCPVTCRPVGFVAPPQRRRSVVCLDEHDVV   [1026]
ADAMTS4      ------------------------------------------------------------   [837]
ADAMTS5      ------------------------------------------------------------   [930]
ADAMTS8      ------------------------------------------------------------   [890]
ADAMTS1      PLKKPKHFIDF-CTMAE-CS----------------------------------------   [967]
ADAMTS15     LHRKPQELDF--CVLRP-C-----------------------------------------   [950]
dCG6107      LKFDVAYHEE--CREG--C----WVLSEWSTCSKSCGT-GSQQREAHCYLHNSRVSDDL-   [1126]
ADAMTS9      SHPKPSNREK--CSGE--CNTGGWRYSAWTECSKSCDG-GTQRRRAICVNTRNDVLDDSK   [1036]
ADAMTS20     DQLKPPTQEL--CHGN--CVFTRWHYSEWSQCSRSCGG-GERSRESYCMNNFGHRLADNE   [992]
mask         ------------------------------------------------------------   [528]
Ce gon-1     RNSKPSDIAS--CYID--CSGRKWNYGEWTSCSETCGSNGKMHRKSYCVDDSNRRVDESL   [1058]
[Intron                           51       52                                   ]
```

```
[                    1390      1400      1410      1420      1430      1440]
[                    .         .         .         .         .         .]
[Modal, ≥80%, full maj.                                                            ]
ADAMTS6      Q----CPEESKPPVRIR---CSLGR--CPPPR-----------------------WVTGD   [1026]
ADAMTS10     H----CSPAAKPPATMR---CNLRR--CPPAR-----------------------WVAGE   [1011]
ADAMTS18     -----CTSLPRPELQEG---CVLGR--CPKNSRLQ--------------------WVASS   [1060]
ADAMTS16     PDAV-CTSEPKPRMHEA---CLLQR--CHKPKKLQ--------------------WLVSA   [1060]
ADAMTS7      -----CDEAQQPASEVT---CSLPL--CR--------------------------WPLGT   [1000]
ADAMTS12     -----CDVTRKPNSRAL---CGLQQ--CP----------------------------SS   [998]
ADAMTS17     -----CDASTRPRAEEA---CEDYSG-CYE-------------------------WKTGD   [1007]
ADAMTS19     -----CVLSTRPREAED---CEDYSK-CYV-------------------------WRMGD   [1095]
ADAMTS2      -----CQEERPETART-CRLGPCPRNISDPSKKSYVV-----------------QWLSRP   [1048]
ADAMTS3      -----CDGEKPESVRA-CQLPPCNDEP- --------------------------------   [1027]
ADAMTS14     -----CEGDRPDTVQV-CSLPACGGNHQNSTVRADVWELVTPEG----------QWVPQS   [1041]
ADAMTS13     EGPG-SVDEKLPAPEP----CVGMS--CPPGWGHLDATSAGEKAPSPWGSIRTGAQAAHV   [896]
dCG4096      VADAECGHLQKPAEME---PCESSLPICRTK-----------------------------   [1054]
ADAMTS4      ------------------------------------------------------------   [837]
ADAMTS5      ------------------------------------------------------------   [930]
ADAMTS8      ------------------------------------------------------------   [890]
ADAMTS1      ------------------------------------------------------------   [967]
ADAMTS15     ------------------------------------------------------------   [950]
dCG6107      -----CNPRTKPHLNTLIGICNTES--CPTYTKSPNALAVSN-------------WVIGE   [1166]
ADAMTS9      -----CTHQEKVTIQR----CSEFP--CPQ-------------------------WKSGD   [1060]
ADAMTS20     -----CQELSRVTREN----CNEFS--CPS-------------------------WAASE   [1016]
mask         ------------------------------------------------------------   [528]
Ce gon-1     -----CGREQKEATERE---CNRIP--CPR-------------------------WVYGH   [1083]
[Intron                               53      54,55                             ]
```

```
[                    1450      1460      1470      1480      1490      1500]
[                    .         .         .         .         .         .]
[Modal, ≥80%, full maj.                                                           ]
ADAMTS6      WGQ----CSAQCGLGQQMRTVQCLSYTGQASSD-------CLETVRPPSMQQ--CESK-C   [1072]
ADAMTS10     WGE----CSAQCGVGQRQRSVRCTSHTGQASHE-------CTEALRPPTTQQ--CEAK-C   [1057]
ADAMTS18     WSE----CSATCGLGVRKREMKCSEKGFQGKLITFPERR-CRNIKKPNLDLEETCNRRAC   [1115]
ADAMTS16     WSQ----CSVTCERGTQKRFLKCAEKYVSGKYRELASKK-CSHLPKPSLELERACAPLPC   [1115]
ADAMTS7      LGPEGSGSGSSSHELFNEADFIPHHLAPRPSPASSPKPG-TMGNAIEEEAPELDLPGPVF   [1059]
ADAMTS12     RRVLKPNKGTISNGKNPPTLKPVPPPTSRPRMLTTPTGP--------ESMSTSTPAISSP   [1050]
ADAMTS17     WST----CSSTCGKGLQSRVVQCMHKVTGRHGSE------CPALSKPAPYRQ--CYQEVC   [1055]
ADAMTS19     WSK----CSITCGKGMQSRVIQCMHKITGRHGNE------CFSSEKPAAYRP--CHLQPC   [1143]
ADAMTS2      ---------DPDSPIRKISSKGHCQGDKSIFCRMEVLSRYCSIPGYNKLS----CKS--C   [1093]
ADAMTS3      ----- -----------------CLGDKSIFCQMEVLARYCSIPGYNKLC----CES--C   [1027]
ADAMTS14     ----------PLHPIYKISSTEPCTGDRSVFCQMEVLDRYCSIPGYHRL-----CCVS-C   [1095]
ADAMTS13     WTPAAGSCSVSCGRGLMELRFLCMDSALRVPVQEEL----CGLASKPGSRREV-CQAVPC   [951]
dCG4096      ------------------------------------------------------------   [1054]
ADAMTS4      ------------------------------------------------------------   [837]
ADAMTS5      ------------------------------------------------------------   [930]
ADAMTS8      ------------------------------------------------------------   [890]
ADAMTS1      ------------------------------------------------------------   [967]
ADAMTS15     ------------------------------------------------------------   [950]
dCG6107      WGE----CNEWCEKTRSVS---CSHPYGIG----------CGSRKPKDVRK---CCHIKY   [1206]
ADAMTS9      WSE----CLVTCGKGHKHRQVWCQFGEDRLNDRM------CDPETKPTSMQT--CQQPEC   [1108]
ADAMTS20     WSE----CLVTCGKGTKQRQVWCQLNVDHLSDGF------CNSSTKPESLSP--CELHTC   [1064]
mask         ------------------------------------------------------------   [528]
Ce gon-1     WSE----CSRSCDGGVKMRHAQCLDAADRETHTSR-----CGPAQTQEH-----CNEHAC   [1129]
[Intron                 56       57     58                                  ]

[                    1510      1520      1530      1540      1550      1560]
[                    .         .         .         .         .         .]
[Modal, ≥80%, full maj.                                                           ]
ADAMTS6      D--------S-TPISNTEECKDVNKVAYCPLVLKFKFCSRAYFRQMCCKTCQGH------   [1117]
ADAMTS10     D--------SPTPGDGPEECKDVNKVAYCPLVLKFQFCSRAYFRQMCCKTCQGH------   [1103]
ADAMTS18     PAHPVYNMVAG----WYSLPWQQCTVTCGGGVQTRSVHCVQQGRPSSS--------CLLH   [1163]
ADAMTS16     PRHPPFAAAGPSRGSWFASPWSQCTASCGGGVQTRSVQCLAGGRPASG--------CLLH   [1167]
ADAMTS7      VDDFYYDYNFINFHEDLSYGPSEEPDLDLAGTGDRTPPPHSRPAAPSTGSPVPATEPPAA   [1119]
ADAMTS12     SPTTASKEGDLGGKQWQDSSTQPELSSRYLISTGSTSQPILTSQSLSIQPSEENVSSSDT   [1110]
ADAMTS17     NDRINANTITSPRLAALTYKCTRDQWTVYCRVIREKNLCQDMRWYQRCCQTCRDFYANKM   [1115]
ADAMTS19     NEKINVNTITSPRLAALTFKCLGDQWPVYCRVIREKNLCQDMRWYQRCCETCRDFYAQKL   [1150]
ADAMTS2      NLYNNLTNVEGRIEPPPGKHNDIDVF----------------------------------   [1119]
ADAMTS3      SKRSSTLPPPYLLEAAETHDDVISNP----------------------------------   [1076]
ADAMTS14     IKKASGPNPGPDPGPTSLPP----------------------------------------   [1115]
ADAMTS13     PA-------------RWQYKLAACSVSCGRGVVRRILYCARAHGEDDGEEILLDTQCQGL   [998]
dCG4096      ------------------------------------------------------------   [1054]
ADAMTS4      ------------------------------------------------------------   [837]
ADAMTS5      ------------------------------------------------------------   [930]
ADAMTS8      ------------------------------------------------------------   [890]
ADAMTS1      ------------------------------------------------------------   [967]
ADAMTS15     ------------------------------------------------------------   [950]
dCG6107      TSD-----------------WTDCSVQCGEGVKRKKQSCTRVYKPDVPGTR---KRRVYD   [1246]
ADAMTS9      AS-------------WQAGPWGQCSVTCGQGYQLRAVKCIIGTYMSVVDDND----CNAA   [1151]
ADAMTS20     AS-------------WQVGPWGPCTTTCGHGYQMRDVKCVNELASAVLEDTE----CHEA   [1107]
mask         ------------------------------------------------------------   [528]
Ce gon-1     TW-------------WQFGVWSDCSAKCGDGVQYRDANCTDRHRSVLPEHR-----CLKM   [1171]
[Intron                       59,60,61,62                                63   ]
```

```
[                    1570      1580      1590      1600      1610      1620]
[                       .         .         .         .         .         .]
[Modal, ≥80%, full maj.                                                     ]
ADAMTS6      ------------------------------------------------------------   [1117]
ADAMTS10     ------------------------------------------------------------   [1103]
ADAMTS18     QKPPVLRACNTNFCP---------------------------------------------   [1178]
ADAMTS16     QKPSASLACNTHFCP---------------------------------------------   [1182]
ADAMTS7      KEEGVLGPWSPSPWPSQAGRSPPPPSEQTPGNPLINFLPEEDTPIGAPDLGLPSLSWPRV   [1179]
ADAMTS12     GPTSEGGLVATTTSGSGLSSSRNPITWPVTPFYNTLTKGPEMEIHSGSGEEREQPEDKDE   [1170]
ADAMTS17     RQPPPSS-----------------------------------------------------   [1122]
ADAMTS19     QQKS--------------------------------------------------------   [1150]
ADAMTS2      ------------------------------------------------------------   [1119]
ADAMTS3      SDLPRSLVMPTSLVPYHSETPAKKMSLSSISSVGGPNAYAAFRPNSKPDGANLRQRSAQQ   [1136]
ADAMTS14     ------------------------------------------------------------   [1115]
ADAMTS13     PRPEPQEACSLEPCPPRWKVMSLGPCSASCGLGTARRSVACVQLDQGQDVEVDEAACAAL   [1058]
dCG4096      ------------------------------------------------------------   [1054]
ADAMTS4      ------------------------------------------------------------   [837]
ADAMTS5      ------------------------------------------------------------   [930]
ADAMTS8      ------------------------------------------------------------   [890]
ADAMTS1      ------------------------------------------------------------   [967]
ADAMTS15     ------------------------------------------------------------   [950]
dCG6107      QLQVECLGLEALSC----------------------------------------------   [1260]
ADAMTS9      TRPTDTQ------------------CSATCGKGTRMRYVSCRDENGSVADESAC-ATLPR   [1192]
ADAMTS20     SRPSDRQ------------------CSVSCGRGTQARYVSCRDALDRIADESYC-AHLPR   [1148]
mask         ------------------------------------------------------------   [528]
Ce gon-1     EKIIT-KPCHRESCPKYKLGEWSQ-CSVSCEDGWSSRRVSCVSGNGTEVDMSLC-GTASD   [1228]
[Intron                     64        65                                  ]

[                    1630      1640      1650      1660      1670      1680]
[                       .         .         .         .         .         .]
[Modal, ≥80%, full maj.                                                     ]
ADAMTS6      ------------------------------------------------------------   [1117]
ADAMTS10     ------------------------------------------------------------   [1103]
ADAMTS18     ------------------------------------------------------------   [1178]
ADAMTS16     ------------------------------------------------------------   [1182]
ADAMTS7      STDGLQTPATPESQNDFPVGKDSQSQLPPPWRDRTNEVFKDDEEPKGRGAPHLPPRPSST   [1239]
ADAMTS12     SNPVIWTKIRVPGNDAPVESTEMPLAPPLTPDLSRESWWPPFSTVMEGLLPSQRPTTSET   [1230]
ADAMTS17     ------------------------------------------------------------   [1122]
ADAMTS19     ------------------------------------------------------------   [1150]
ADAMTS2      -MPTLPVPTVAMEVRPSPSTPLEVPLNASSTNATEDHPETNAVDEPYKIHGLEDEVQPPN   [1178]
ADAMTS3      AGSKTVRLVTVPSSPPTKRVHLSSASQMAAASFFAASDSIGASSQARTSKKDGKIIDNRR   [1196]
ADAMTS14     ------------------------------------------------------------   [1115]
ADAMTS13     VRPEASVPCLIADCTYRWHVGTWMECSVSCGDGIQRRRDTCLGPQAQAPVPADFCQHLPK   [1118]
dCG4096      ------------------------------------------------------------   [1054]
ADAMTS4      ------------------------------------------------------------   [837]
ADAMTS5      ------------------------------------------------------------   [930]
ADAMTS8      ------------------------------------------------------------   [890]
ADAMTS1      ------------------------------------------------------------   [967]
ADAMTS15     ------------------------------------------------------------   [950]
dCG6107      ------------------------------------------------------------   [1260]
ADAMTS9      PVAKEE-CSVTPCGQ--WKALDWSSCSVTCGQGRATRQVMCVNYSDHVIDRSECDQDYIP   [1249]
ADAMTS20     PAEIWD-CFTP-CGE--WQAGDWSPCSASCGHGKTTRQVLCMNYHQPIDENY-CDPEVRP   [1203]
mask         ------------------------------------------------------------   [528]
Ce gon-1     RPASHQTCNLGTCPF--WRNTDWSACSVSCGIGHRERTTECIYREQSVDASF-CGDTKMP   [1285]
[Intron                               66                                 ]
```

```
[                    1690      1700      1710      1720      1730      1740]
[                    .         .         .         .         .         .]
[Modal, ≥80%, full maj.                                                              ]
ADAMTS6     ------------------------------------------------------------   [1117]
ADAMTS10    ------------------------------------------------------------   [1103]
ADAMTS18    ------------------------------------------------------------   [1178]
ADAMTS16    ------------------------------------------------------------   [1182]
ADAMTS7     LPPLSPVGSTHSSPSPDVAELWTGGTVAWEPALEGGLGPVDSELWPTVGVASLLPPPIAP   [1299]
ADAMTS12    GTPRVEGMVTEKPANTLLPLGGDHQPEPSGKTANRNHLKLPNNMNQTKSSEPVLTEEDAT   [1290]
ADAMTS17    ------------------------------------------------------------   [1122]
ADAMTS19    ------------------------------------------------------------   [1150]
ADAMTS2     LIPRRPSPYEKTRNQRIQELIDEMRKKEMLGKF---------------------------   [1211]
ADAMTS3     PTRSSTLER---------------------------------------------------   [1205]
ADAMTS14    ------------------------------------------------------------   [1115]
ADAMTS13    PVTVRGCWAGPCVGQGTPSLVPHEEAAAPGRTTATPAGASLEWSQARGLLFSPAPQPRRL   [1178]
dCG4096     ------------------------------------------------------------   [1054]
ADAMTS4     ------------------------------------------------------------   [837]
ADAMTS5     ------------------------------------------------------------   [930]
ADAMTS8     ------------------------------------------------------------   [890]
ADAMTS1     ------------------------------------------------------------   [967]
ADAMTS15    ------------------------------------------------------------   [950]
dCG6107     ------------------------------------------------------------   [1260]
ADAMTS9     ETDQDCSMSPCPQRTPDSGLAQHPFQNEDYRPRSAS--------PSRTHVLGGNQWRTGP   [1301]
ADAMTS20    LMEQECSLAACPPAHSHFPSSPVQPSYYLSTNLPLTQKLEDNENQVVHPSVRGNQWRTGP   [1263]
mask        ------------------------------------------------------------   [528]
Ce gon-1    ETSQTCHLLPCTS------------------------------------------WKPSH   [1303]
[Intron                                                                         ]

[                    1750      1760      1770      1780      1790      1800]
[                    .         .         .         .         .         .]
[Modal, ≥80%, full maj.                                                              ]
ADAMTS6     ------------------------------------------------------------   [1117]
ADAMTS10    ------------------------------------------------------------   [1103]
ADAMTS18    ------------------------------------------------------------   [1178]
ADAMTS16    ------------------------------------------------------------   [1182]
ADAMTS7     LPEMKVRDSSLEPGTPSFPAPGPGSWDLQTVAVWGTFLPTTLTGLGHMPEPALNPGPKGQ   [1359]
ADAMTS12    SLITEGFLLNASNYKQLTNGHGSAHWIVGNWSECSTTCGLGAYWKRVECTTQMDSDCAAI   [1350]
ADAMTS17    ------------------------------------------------------------   [1122]
ADAMTS19    ------------------------------------------------------------   [1150]
ADAMTS2     ------------------------------------------------------------   [1211]
ADAMTS3     ------------------------------------------------------------   [1205]
ADAMTS14    ------------------------------------------------------------   [1115]
ADAMTS13    LPGPQENSVQSSACGRQHLEPTGTIDMRGPGQADCAVAIGRPLGEVVTLRVLESSLNCSA   [1238]
dCG4096     ------------------------------------------------------------   [1054]
ADAMTS4     ------------------------------------------------------------   [837]
ADAMTS5     ------------------------------------------------------------   [930]
ADAMTS8     ------------------------------------------------------------   [890]
ADAMTS1     ------------------------------------------------------------   [967]
ADAMTS15    ------------------------------------------------------------   [950]
dCG6107     ------------------------------------------------------------   [1260]
ADAMTS9     WGACSSTCAGGSQRRVVVCQDENGYTAND---CVERIKPDEQRACESGPCPQ--------   [1350]
ADAMTS20    WGSCSSSCSGGLQHRAVVCQDENGQSASY---CDAASKPPELQQCGPGPCPQ--------   [1312]
mask        ------------------------------------------------------------   [528]
Ce gon-1    WSPCSVTCGSGIQTRSVSCTRGSEGTIVDEYFCDRNTRPRLKKTCEKDTCDGPRVLQKLQ   [1363]
[Intron         67      68                     69                               ]
```

```
[                    1810      1820      1830      1840      1850      1860]
[                    .         .         .         .         .         .]
[Modal, ≥80%, full maj.                                                     ]
ADAMTS6       ------------------------------------------------------------   [1117]
ADAMTS10      ------------------------------------------------------------   [1103]
ADAMTS18      ------------------------------------------------------------   [1178]
ADAMTS16      ------------------------------------------------------------   [1182]
ADAMTS7       PESLSPEVPLSSRLLSTPAWDSPANSHRVPETQPLAPSLAEAGPPADPLVVRNASWQAGN   [1419]
ADAMTS12      QRPDPAKRCHLRPCAGWKVGNWSKCSRNCSGGFKIREIQCVDSRDHRNLRPFHCQFLAGI   [1410]
ADAMTS17      ------------------------------------------------------------   [1122]
ADAMTS19      ------------------------------------------------------------   [1150]
ADAMTS2       ------------------------------------------------------------   [1211]
ADAMTS3       ------------------------------------------------------------   [1205]
ADAMTS14      ------------------------------------------------------------   [1115]
ADAMTS13      GDMLLLWGRLTWRKMCRKLLDMTFSSKTNTLVVRQRCGRPGGGVLLRYGSQLAPETFYRE   [1298]
dCG4096       ------------------------------------------------------------   [1054]
ADAMTS4       ------------------------------------------------------------   [837]
ADAMTS5       ------------------------------------------------------------   [930]
ADAMTS8       ------------------------------------------------------------   [890]
ADAMTS1       ------------------------------------------------------------   [967]
ADAMTS15      ------------------------------------------------------------   [950]
dCG6107       ------------------------------------------------------------   [1260]
ADAMTS9       -------WAYGNWGECTKLCGGGIRTRLVVCQRSNGERFPDLSCEILDKPPDREQCNTHA   [1403]
ADAMTS20      -------WNYGNWGECSQTCGGGIKSRLVICQFPNGQILEDHNCEIVNKPPSVIQCHMHA   [1365]
mask          ------------------------------------------------------------   [528]
Ce gon-1      ADVPPIRWATGPWTACSATCGNGTQRRLLKCRDHVRDLPDEY-CNHLDKEVSTRNCRLRD   [1422]
[Intron        70             71                                                ]

[                    1870      1880      1890      1900      1910      1920]
[                    .         .         .         .         .         .]
[Modal, ≥80%, full maj.                                                     ]
ADAMTS6       ------------------------------------------------------------   [1117]
ADAMTS10      ------------------------------------------------------------   [1103]
ADAMTS18      ------------------------------------------------------------   [1178]
ADAMTS16      ------------------------------------------------------------   [1182]
ADAMTS7       WSECSTTCGLGAVWRPVRCSSGRDEDCAPAGRPQPARRCHLRPCAT--------------   [1465]
ADAMTS12      PPPLSMSCNPEPCEAWQVEPWSQCSRSCGGGVQERGVFCPGGLCDWTKRPTSTMSCNEHL   [1470]
ADAMTS17      ------------------------------------------------------------   [1122]
ADAMTS19      ------------------------------------------------------------   [1150]
ADAMTS2       ------------------------------------------------------------   [1211]
ADAMTS3       ------------------------------------------------------------   [1205]
ADAMTS14      ------------------------------------------------------------   [1115]
ADAMTS13      CDMQLFGPWGEIVSPSLSPATSNAGGCRLFINVAPHARIAIHALATNMGAGTEGANASYI   [1358]
dCG4096       ------------------------------------------------------------   [1054]
ADAMTS4       ------------------------------------------------------------   [837]
ADAMTS5       ------------------------------------------------------------   [930]
ADAMTS8       ------------------------------------------------------------   [890]
ADAMTS1       ------------------------------------------------------------   [967]
ADAMTS15      ------------------------------------------------------------   [950]
dCG6107       ------------------------------------------------------------   [1260]
ADAMTS9       CPHDAAWSTGPWSSCSVSCGRGHKQRNVY-CMAKDGSHLESDY---CKHLAKPHGHRKCR   [1459]
ADAMTS20      CPADVSWHQEPWTSCSASCGKGRKYREVF-CIDQFQRKLEDTN---CSQVQKPPTHKACR   [1421]
mask          ------------------------------------------------------------   [528]
Ce gon-1      CSY---WKMAEWEECPATCGTHVQQSRNVTCVSAEDGGRTILKDVDCDVQKRPTSARNCR   [1479]
[Intron       72                                                                ]
```

```
[                    1930      1940      1950      1960      1970      1980]
[                    .         .         .         .         .         .]
[Modal, ≥80%, full maj.                                                           ]
ADAMTS6     ------------------------------------------------------------   [1117]
ADAMTS10    ------------------------------------------------------------   [1103]
ADAMTS18    ------------------------------------------------------------   [1178]
ADAMTS16    ------------------------------------------------------------   [1182]
ADAMTS7     -------------WHSGNWSKCSRSCGGGSSVRDVQCVDTRDLRPLRPFHCQPGPAKPPA   [1512]
ADAMTS12    CCH----------WATGNWDLCSTSCGGGFQKRIVQCVPSEGNKTEDQDQCLCDHKPRPP   [1520]
ADAMTS17    ------------------------------------------------------------   [1122]
ADAMTS19    ------------------------------------------------------------   [1150]
ADAMTS2     ------------------------------------------------------------   [1211]
ADAMTS3     ------------------------------------------------------------   [1205]
ADAMTS14    ------------------------------------------------------------   [1115]
ADAMTS13    LIRDTHSLRTTAFHGQQVLYWESESSQAEMEFSEGFLKAQASLRGQYWTLQSWVPEMQDP   [1418]
dCG4096     ------------------------------------------------------------   [1054]
ADAMTS4     ------------------------------------------------------------   [837]
ADAMTS5     ------------------------------------------------------------   [930]
ADAMTS8     ------------------------------------------------------------   [890]
ADAMTS1     ------------------------------------------------------------   [967]
ADAMTS15    ------------------------------------------------------------   [950]
dCG6107     ------------------------------------------------------------   [1260]
ADAMTS9     GGRCPK-------WKAGAWSQCSVSCGRGVQQRHVGCQIGTHKIARETECNPYTRPESER   [1512]
ADAMTS20    SVRCPS-------WKANSWNECSVTCGSGVQQRDVYCRLKGVGQVVEEMCDQSTRPCSQR   [1474]
mask        ------------------------------------------------------------   [528]
Ce gon-1    LEPCPKGEEHIGSWIIGDWSKCSASCGGGWRRRSVSCTSSSCDETRKPKMFDKCNEELCP   [1539]
[Intron      73                  74                                            ]
[                    1990      2000      2010      2020      2030      2040]
[                    .         .         .         .         .         .]
[Modal, ≥80%, full maj.                                                           ]
ADAMTS6     ------------------------------------------------------------   [1117]
ADAMTS10    ------------------------------------------------------------   [1103]
ADAMTS18    ------------------------------------------------------------   [1178]
ADAMTS16    ------------------------------------------------------------   [1182]
ADAMTS7     HRPCGAQPCLSWYTSSWRECSEACGGGEQQRLVTCPEPGL-------CEEALRPNTTRPC   [1565]
ADAMTS12    EFKKCNQQACKKSADLLCTKDKLSASFCQTLKAMKKCSVPTVRAECCFSCPQTHITHTQR   [1580]
ADAMTS17    ------------------------------------------------------------   [1122]
ADAMTS19    ------------------------------------------------------------   [1150]
ADAMTS2     ------------------------------------------------------------   [1211]
ADAMTS3     ------------------------------------------------------------   [1205]
ADAMTS14    ------------------------------------------------------------   [1115]
ADAMTS13    QSWKGKEGT---------------------------------------------------   [1427]
dCG4096     ------------------------------------------------------------   [1054]
ADAMTS4     ------------------------------------------------------------   [837]
ADAMTS5     ------------------------------------------------------------   [930]
ADAMTS8     ------------------------------------------------------------   [890]
ADAMTS1     ------------------------------------------------------------   [967]
ADAMTS15    ------------------------------------------------------------   [950]
dCG6107     ------------------------------------------------------------   [1260]
ADAMTS9     DCQGPRCPLYTWRAEEWQECTKTCGEGSRYRKVVCVDDNKNEVHGARCDVSKRPVDRESC   [1572]
ADAMTS20    RC---------WSQDCVQHKGMERGRLNCANNCGFSYRQRITYCTEIPSTKKHKLHRLRP   [1525]
mask        ------------------------------------------------------------   [528]
Ce gon-1    PLTNNS-----WQISPWTHCSVSCGGGVQRRKIWCEDVLSGRKQDDIECSEIKPREQRDC   [1594]
[Intron                        75                                         ]
```

```
[                    2050      2060      2070      2080      2090      2100]
[                    .         .         .         .         .         .]
[Modal, ≥80%, full maj.                                                          ]
ADAMTS6     ------------------------------------------------------------   [1117]
ADAMTS10    ------------------------------------------------------------   [1103]
ADAMTS18    ------------------------------------------------------------   [1178]
ADAMTS16    ------------------------------------------------------------   [1182]
ADAMTS7     NTHPCTQ-WVVGPWGQCSAPCGGGVQRRLVKCVNTQTGLPEEDSDQCGHEAWPESSRPCG   [1624]
ADAMTS12    QRRQRLLQKSKEL-----------------------------------------------   [1593]
ADAMTS17    ------------------------------------------------------------   [1122]
ADAMTS19    ------------------------------------------------------------   [1150]
ADAMTS2     ------------------------------------------------------------   [1211]
ADAMTS3     ------------------------------------------------------------   [1205]
ADAMTS14    ------------------------------------------------------------   [1115]
ADAMTS13    ------------------------------------------------------------   [1427]
dCG4096     ------------------------------------------------------------   [1054]
ADAMTS4     ------------------------------------------------------------   [837]
ADAMTS5     ------------------------------------------------------------   [930]
ADAMTS8     ------------------------------------------------------------   [890]
ADAMTS1     ------------------------------------------------------------   [967]
ADAMTS15    ------------------------------------------------------------   [950]
dCG6107     ------------------------------------------------------------   [1260]
ADAMTS9     SLHPCEYVWITGEWSECSVTCGKGYKQRLVSCSEIYTGKENYEYSSQTTINCPGTQPPSV   [1632]
ADAMTS20    IVYQECPVVPSSQVYQCINSCLHLATWKVGKWSKCSVTCGIGIMKRQVKCITKHGLSSDL   [1585]
mask        ------------------------------------------------------------   [528]
Ce gon-1    EMPPCRSHYHNKTSSASMTSLSSSNSNTTSSASASSLPILPPVVSWQTSAWSACSAKCGR   [1654]
[Intron                                   76                             ]
```

```
[                    2110      2120      2130      2140      2150      2160]
[                    .         .         .         .         .         .]
[Modal, ≥80%, full maj.                                                          ]
ADAMTS6     ------------------------------------------------------------   [1117]
ADAMTS10    ------------------------------------------------------------   [1103]
ADAMTS18    ---------------------------------------------------APEKREDPS   [1187]
ADAMTS16    ---------------------------------------------------IAEKKDAF-   [1190]
ADAMTS7     TEDCEPVEPPRCERDRLSFGFCETLRLLGRCQLPTIRTQCCRSCSPPSHGAPSRGHQRVA   [1684]
ADAMTS12    ------------------------------------------------------------   [1593]
ADAMTS17    ------------------------------------------------------------   [1122]
ADAMTS19    ------------------------------------------------------------   [1162]
ADAMTS2     ------------------------------------------------------------   [1211]
ADAMTS3     ------------------------------------------------------------   [1205]
ADAMTS14    ------------------------------------------------------------   [1115]
ADAMTS13    ------------------------------------------------------------   [1427]
dCG4096     ------------------------------------------------------------   [1054]
ADAMTS4     ------------------------------------------------------------   [837]
ADAMTS5     ------------------------------------------------------------   [930]
ADAMTS8     ------------------------------------------------------------   [890]
ADAMTS1     ------------------------------------------------------------   [967]
ADAMTS15    ------------------------------------------------------------   [950]
dCG6107     ------------------------------------------------------------   [1260]
ADAMTS9     HPCYLRDCPVSATWRVGNWGSCSVSCGVGVMQRSVQCLNNAVQPSHLCRSDLKPEERKTC   [1692]
ADAMTS20    CLNHLKPGAQKKCYANDCKS-------------------------------FTTCKEIQV   [1614]
mask        ------------------------------------------------------------   [528]
Ce gon-1    GTKRRVVECVNPSLNVTVASTECDQTKKPVEEVRCRTKHCPRWKTTTWSSCSVTCGRGIR   [1714]
[Intron                         77   78                               79        ]
```

```
[                    2170      2180      2190      2200      2210      2220]
[                       .         .         .         .         .         .]
[Modal, ≥80%, full maj.                                                             ]
ADAMTS6      ------------------------------------------------------------   [1117]
ADAMTS10     ------------------------------------------------------------   [1103]
ADAMTS18     CVDFFNWCHLVPQHGVCNHKFYGKQCCKSCTRKI--------------------------   [1221]
ADAMTS16     CKDYFHWCYLVPQHGMCSHKFYGKQCCKTCSKSNL-------------------------   [1225]
ADAMTS7      RR----------------------------------------------------------   [1686]
ADAMTS12     ------------------------------------------------------------   [1593]
ADAMTS17     ------------------------------------------------------------   [1122]
ADAMTS19     ------------------------------------------------------------   [1207]
ADAMTS2      ------------------------------------------------------------   [1211]
ADAMTS3      ------------------------------------------------------------   [1205]
ADAMTS14     ------------------------------------------------------------   [1115]
ADAMTS13     ------------------------------------------------------------   [1427]
dCG4096      ------------------------------------------------------------   [1054]
ADAMTS4      ------------------------------------------------------------   [837]
ADAMTS5      ------------------------------------------------------------   [930]
ADAMTS8      ------------------------------------------------------------   [890]
ADAMTS1      ------------------------------------------------------------   [967]
ADAMTS15     ------------------------------------------------------------   [950]
dCG6107      ------------------------------------------------------------   [1260]
ADAMTS9      RNVYN-------------------------------------------------------   [1697]
ADAMTS20     KN-HIRKDGDYYLNIKGRIIKVL-------------------------------------   [1636]
mask         ------------------------------------------------------------   [528]
Ce gon-1     RREVQCYRGRKNLVSDSECNPKTKLNSVANCFPVACPAYRWNVTPWSKCKDECARGQKQT   [1774]

[                    2230      2240      2250      2260      2270      2280]
[                       .         .         .         .         .         .]
[Modal, ≥80%, full maj.                                                             ]
ADAMTS6      ------------------------------------------------------------   [1117]
ADAMTS10     ------------------------------------------------------------   [1103]
ADAMTS18     ------------------------------------------------------------   [1221]
ADAMTS16     ------------------------------------------------------------   [1225]
ADAMTS7      ------------------------------------------------------------   [1686]
ADAMTS12     ------------------------------------------------------------   [1593]
ADAMTS17     ------------------------------------------------------------   [1122]
ADAMTS19     ------------------------------------------------------------   [1207]
ADAMTS2      ------------------------------------------------------------   [1211]
ADAMTS3      ------------------------------------------------------------   [1205]
ADAMTS14     ------------------------------------------------------------   [1115]
ADAMTS13     ------------------------------------------------------------   [1427]
dCG4096      ------------------------------------------------------------   [1054]
ADAMTS4      ------------------------------------------------------------   [837]
ADAMTS5      ------------------------------------------------------------   [930]
ADAMTS8      ------------------------------------------------------------   [890]
ADAMTS1      ------------------------------------------------------------   [967]
ADAMTS15     ------------------------------------------------------------   [950]
dCG6107      ------------------------------------------------------------   [1260]
ADAMTS9      ------------------------------------------------------------   [1697]
ADAMTS20     ------------------------------------------------------------   [1636]
mask         ------------------------------------------------------------   [528]
Ce gon-1     RRVHCISTSGKRAAPRMCELARAPTSIRECDTSNCPYEWVPGDWQTCSKSCGEGVQTREV   [1834]
```

```
[                  2290      2300      2310      2320      2330      2340]
[                     .         .         .         .         .         .]
[Modal, ≥80%, full maj.                                                            ]
ADAMTS6     ------------------------------------------------------------   [1117]
ADAMTS10    ------------------------------------------------------------   [1103]
ADAMTS18    ------------------------------------------------------------   [1221]
ADAMTS16    ------------------------------------------------------------   [1225]
ADAMTS7     ------------------------------------------------------------   [1686]
ADAMTS12    ------------------------------------------------------------   [1593]
ADAMTS17    ------------------------------------------------------------   [1122]
ADAMTS19    ------------------------------------------------------------   [1207]
ADAMTS2     ------------------------------------------------------------   [1211]
ADAMTS3     ------------------------------------------------------------   [1205]
ADAMTS14    ------------------------------------------------------------   [1115]
ADAMTS13    ------------------------------------------------------------   [1427]
dCG4096     ------------------------------------------------------------   [1054]
ADAMTS4     ------------------------------------------------------------   [837]
ADAMTS5     ------------------------------------------------------------   [930]
ADAMTS8     ------------------------------------------------------------   [890]
ADAMTS1     ------------------------------------------------------------   [967]
ADAMTS15    ------------------------------------------------------------   [950]
dCG6107     ------------------------------------------------------------   [1260]
ADAMTS9     ------------------------------------------------------------   [1697]
ADAMTS20    ------------------------------------------------------------   [1636]
mask        ------------------------------------------------------------   [528]
Ce gon-1    RCRRKINFNSTIPIIFMLEDEPAVPKEKCELFPKPNESQTCELNPCDSEFKWSFGPWGEC   [1894]

[                  2350      2360      2370      2380      2390      2400]
[                     .         .         .         .         .         .]
[Modal, ≥80%, full maj.                                                            ]
ADAMTS6     ------------------------------------------------------------   [1117]
ADAMTS10    ------------------------------------------------------------   [1103]
ADAMTS18    ------------------------------------------------------------   [1221]
ADAMTS16    ------------------------------------------------------------   [1225]
ADAMTS7     ------------------------------------------------------------   [1686]
ADAMTS12    ------------------------------------------------------------   [1593]
ADAMTS17    ------------------------------------------------------------   [1122]
ADAMTS19    ------------------------------------------------------------   [1207]
ADAMTS2     ------------------------------------------------------------   [1211]
ADAMTS3     ------------------------------------------------------------   [1205]
ADAMTS14    ------------------------------------------------------------   [1115]
ADAMTS13    ------------------------------------------------------------   [1427]
dCG4096     ------------------------------------------------------------   [1054]
ADAMTS4     ------------------------------------------------------------   [837]
ADAMTS5     ------------------------------------------------------------   [930]
ADAMTS8     ------------------------------------------------------------   [890]
ADAMTS1     ------------------------------------------------------------   [967]
ADAMTS15    ------------------------------------------------------------   [950]
dCG6107     ------------------------------------------------------------   [1260]
ADAMTS9     ------------------------------------------------------------   [1697]
ADAMTS20    ------------------------------------------------------------   [1636]
mask        ------------------------------------------------------------   [528]
Ce gon-1    SKNCGQGIRRRRVKCVANDGRRVERVKCTTKKPRRTQYCFERNCLPSTCQELKSQNVKAK   [1954]
```

```
[                   2410      2420      2430      2440      2450      2460]
[                      .         .         .         .         .         .]
[Modal, ≥80%, full maj.                                                      ]
ADAMTS6     ------------------------------------------------------------   [1117]
ADAMTS10    ------------------------------------------------------------   [1103]
ADAMTS18    ------------------------------------------------------------   [1221]
ADAMTS16    ------------------------------------------------------------   [1225]
ADAMTS7     ------------------------------------------------------------   [1686]
ADAMTS12    ------------------------------------------------------------   [1593]
ADAMTS17    ------------------------------------------------------------   [1122]
ADAMTS19    ------------------------------------------------------------   [1207]
ADAMTS2     ------------------------------------------------------------   [1211]
ADAMTS3     ------------------------------------------------------------   [1205]
ADAMTS14    ------------------------------------------------------------   [1115]
ADAMTS13    ------------------------------------------------------------   [1427]
dCG4096     ------------------------------------------------------------   [1054]
ADAMTS4     ------------------------------------------------------------   [837]
ADAMTS5     ------------------------------------------------------------   [930]
ADAMTS8     ------------------------------------------------------------   [890]
ADAMTS1     ------------------------------------------------------------   [967]
ADAMTS15    ------------------------------------------------------------   [950]
dCG6107     ------------------------------------------------------------   [1260]
ADAMTS9     ------------------------------------------------------------   [1697]
ADAMTS20    ------------------------------------------------------------   [1636]
mask        ------------------------------------------------------------   [528]
Ce gon-1    DGNYTILLDGFTIEIYCHRMNSTIPKAYLNVNPRTNFAEVYGKKLIYPHTCPFNGDRNDS   [2014]

[                   2470      2480      2490      2500      2510      2520]
[                      .         .         .         .         .         .]
[Modal, ≥80%, full maj.                                                      ]
ADAMTS6     ------------------------------------------------------------   [1117]
ADAMTS10    ------------------------------------------------------------   [1103]
ADAMTS18    ------------------------------------------------------------   [1221]
ADAMTS16    ------------------------------------------------------------   [1225]
ADAMTS7     ------------------------------------------------------------   [1686]
ADAMTS12    ------------------------------------------------------------   [1593]
ADAMTS17    ------------------------------------------------------------   [1122]
ADAMTS19    ------------------------------------------------------------   [1207]
ADAMTS2     ------------------------------------------------------------   [1211]
ADAMTS3     ------------------------------------------------------------   [1205]
ADAMTS14    ------------------------------------------------------------   [1115]
ADAMTS13    ------------------------------------------------------------   [1427]
dCG4096     ------------------------------------------------------------   [1054]
ADAMTS4     ------------------------------------------------------------   [837]
ADAMTS5     ------------------------------------------------------------   [930]
ADAMTS8     ------------------------------------------------------------   [890]
ADAMTS1     ------------------------------------------------------------   [967]
ADAMTS15    ------------------------------------------------------------   [950]
dCG6107     ------------------------------------------------------------   [1260]
ADAMTS9     ------------------------------------------------------------   [1697]
ADAMTS20    ------------------------------------------------------------   [1636]
mask        ------------------------------------------------------------   [528]
Ce gon-1    CHCSEDGDASAGLTRFNKVRIDLLNRKFHLADYTFAKREYGVHVPYGTAGDCYSMKDCPQ   [2074]
```

```
[                     2530      2540      2550      2560      2570      2580]
[                     .         .         .         .         .         .]
[Modal, ≥80%, full maj.                                                              ]
ADAMTS6      ------------------------------------------------------------   [1117]
ADAMTS10     ------------------------------------------------------------   [1103]
ADAMTS18     ------------------------------------------------------------   [1221]
ADAMTS16     ------------------------------------------------------------   [1225]
ADAMTS7      ------------------------------------------------------------   [1686]
ADAMTS12     ------------------------------------------------------------   [1593]
ADAMTS17     ------------------------------------------------------------   [1122]
ADAMTS19     ------------------------------------------------------------   [1207]
ADAMTS2      ------------------------------------------------------------   [1211]
ADAMTS3      ------------------------------------------------------------   [1205]
ADAMTS14     ------------------------------------------------------------   [1115]
ADAMTS13     ------------------------------------------------------------   [1427]
dCG4096      ------------------------------------------------------------   [1054]
ADAMTS4      ------------------------------------------------------------   [837]
ADAMTS5      ------------------------------------------------------------   [930]
ADAMTS8      ------------------------------------------------------------   [890]
ADAMTS1      ------------------------------------------------------------   [967]
ADAMTS15     ------------------------------------------------------------   [950]
dCG6107      ------------------------------------------------------------   [1260]
ADAMTS9      ------------------------------------------------------------   [1697]
ADAMTS20     ------------------------------------------------------------   [1636]
mask         ------------------------------------------------------------   [528]
Ce gon-1     GIFSIDLKSAGLKLVDDLNWEDQGHRTSSRIDRFYNNAKVIGHCGGFCGKCSPERYKGLI   [2134]

[                     2590      2600      2610]
[                     .         .         . ]
[Modal, ≥80%, full maj.                            ]
ADAMTS6      -------------------------------   [1117]
ADAMTS10     -------------------------------   [1103]
ADAMTS18     -------------------------------   [1221]
ADAMTS16     -------------------------------   [1225]
ADAMTS7      -------------------------------   [1686]
ADAMTS12     -------------------------------   [1593]
ADAMTS17     -------------------------------   [1122]
ADAMTS19     -------------------------------   [1207]
ADAMTS2      -------------------------------   [1211]
ADAMTS3      -------------------------------   [1205]
ADAMTS14     -------------------------------   [1115]
ADAMTS13     -------------------------------   [1427]
dCG4096      -------------------------------   [1054]
ADAMTS4      -------------------------------   [837]
ADAMTS5      -------------------------------   [930]
ADAMTS8      -------------------------------   [890]
ADAMTS1      -------------------------------   [967]
ADAMTS15     -------------------------------   [950]
dCG6107      -------------------------------   [1260]
ADAMTS9      -------------------------------   [1697]
ADAMTS20     -------------------------------   [1636]
mask         -------------------------------   [528]
Ce gon-1     FEVNTKLLNHVKNGGHIDDELDDDGFSGDMD   [2165]
;
END;
```
